# Supplementary material for: DiPRO1 distinctly reprograms muscle and mesenchymal cancer cells
Source: EMBO Mol Med. 2024 Jul 15;16(8):4. doi: 10.1038/s44321-024-00097-z (PMC11319797; doi:10.1038/s44321-024-00097-z)
Supplement: Supplementary file 1 — Appendix [file 44321_2024_97_MOESM1_ESM.pdf]

Appendix Supplemental Data

DiPRO1 dependent transcriptional and epigenetic regulation distinctly controls the fate of muscle and mesenchymal cancer cells

Table of Contents

Appendix Figure S1 – DiPRO1 direct targets and their functions…p.2-3

Appendix Figure S2 – The codon-optimized DiPRO1 expressing vector…p.4

Appendix Figure S3 – Depletion of DiPRO1 expression affects the survival of rhabdomyosarcoma and Ewing sarcoma cells...p.5

Appendix Figure S4 – Overlap of differentially expressed genes (DEGs) in mesenchymal cancer cells and myoblasts following DiPRO1 KD…p.6

Appendix Figure S5 – Impact of DiPRO1 knockdown on CpG island methylation in RMS and myoblast cells...p.7

Appendix Table S1 – Characteristics of the patients used in the study...p.8

Appendix Table S2 – Disease groups and tumor types in the study cohort…p.8

Appendix Table S3 – List of primers used for transcription analysis...p.8

Appendix Figure S1

A DiPRO1/ZNF555 protein

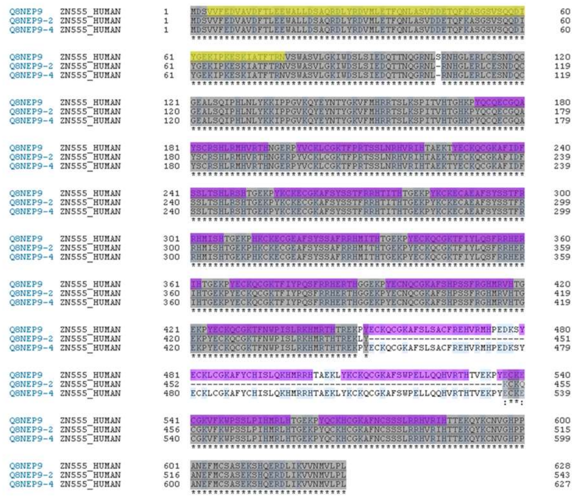

- KRAB A
- Zn-fingers (12-15)
- Identical for 3 isoforms

Predicted size

Isoforme 1 : 73.1 kDa (Q8NEP9-1) 628AA

Isoforme 2 : 63.0 kDa (Q8NEP9-2) 543AA

Isoforme 3 : 73.0 kDa (Q8NEP9-4) 627AA

C

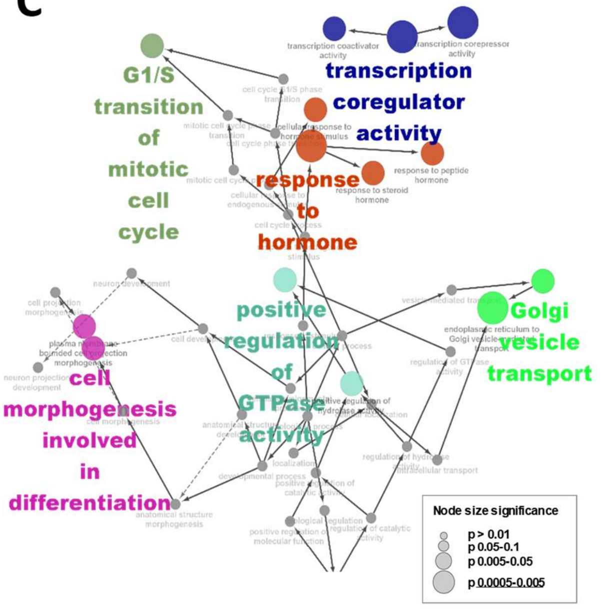

B

Chromatin annotation	%
Transcriptional elongation	6.34
Transcriptional transition	2.38
Weak Transcription	15.45
Weak Promoter	0.59
Active Promoter	0.40
Strong Enhancer	2.18
Weak Enhancer	3.37
Polycomb -repressed	3.17
Heterochrom low signal	60.00
Repetitive/Copy Number Variation	0.79
Poised Promoter	0.40
Insulator	0.40
NA	4.55

D

Rank	Motif	P-value	% of targets	% of BG	Best match
1	GGGTTAGGTTCC	1^{e}-204	16.04	0.01	**Six1** (GSE20150)
2	GTCACAACATTT	1^{e}-84	6.93	0.05	**Foxj1** (PB0120.1)
3	TGTGACCAGA	1^{e}-76	8.71	0.17	**Nr2f2** (PB0153.1)
4	AACACTGAAC	1^{e}-69	12.28	0.85	**Nkx3.1** (GSE28264)
5	TTAAAGACAC	1^{e}-62	5.35	0.16	**Hox9** (PH0073.1)
6	TTGCTCCTAGGG	1^{e}-58	7.92	0.03	**MZF1** (MA0057.1)
7	TCAATACATAAC	1^{e}-49	6.34	0.01	**HNF1B** (MA0153.1)
8	CAGAGGCGCACA	1^{e}-46	37.82	8.85	**Gm397/ZSCAN4** (PB0130.1)

E

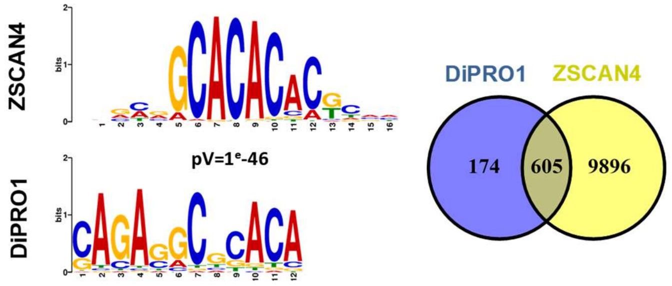

F

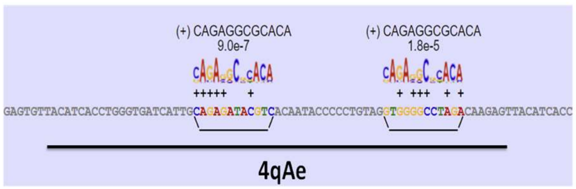

Appendix Figure S1. **DiPRO1 direct targets and their functions.**

A Amino-acid sequence alignment of three DiPRO1/ZNF555 isoforms. Residues shaded in blue correspond to charged amino acids.

B Chromatin annotation of DiPRO1 binding regions. **C,** A network of functional groups of enriched categories was created for the DiPRO1 target genes. GO biological process (BP) groups of enriched terms are represented by colored nodes, and node size reflects the significance of term enrichment, p-value group corrected with Bonferroni stepwise reduction, kappa score = 0.4. Gray nodes correspond to parent nodes connected by flashes to root nodes.

D Top 8 *de novo* motifs for DiPRO1 and the best matches. Motives from the ChIP-seq experimental data were prioritized.

E The most abundant DiPRO1 binding motif 8 and its best match ZSCAN4 (left). The motif alignment was shown for the ZSCAN4 human PB0130_1/Jaspar using the Tomtom tool. Gene overlap between the *cis*-regulatory regions of DiPRO1 and ZSCAN4 (right).

F The DiPRO1 motif 8 matches the beta satellite DNA repeat of the 4qAe sequence.

B-F ChIP-seq data for DiPRO1 (GSM2466593) performed in HEK 293T cells.

A

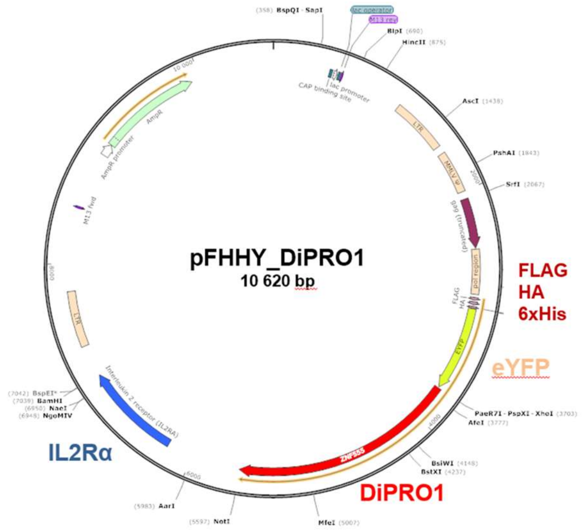

B

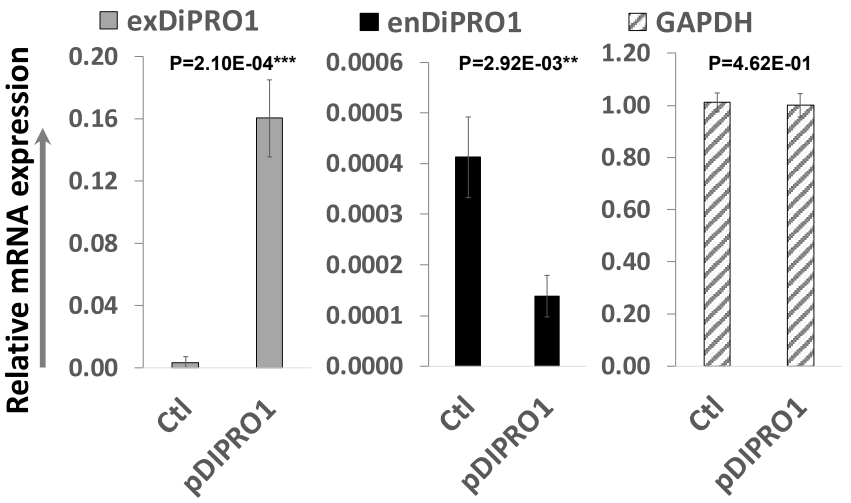

Appendix Figure S2. The codon-optimized DiPRO1 expressing vector.

A Map of the pDiPRO1 retroviral vector showing its major components. The DiPRO1 ORF was inserted into the pOZ vector backbone between the XhoI and NotI sites, with the epitope tags FHHY: FLAG-HA-6xHis-YFP at the amino terminal position. The EMC IRES directs the expression of the IL2Rα ORF, a surface selection marker.

B The codon-optimized DiPRO1 ORF cloned into the pOZ vector was amplified. The primers distinguish between the exogenous (exDiPRO1) and endogenous (enDiPRO1) DiPRO1 gene. Total RNA was extracted from TE671 cells transiently transfected with exogenous DiPRO1 (pDiPRO1) and parental homologs (Ctl) 24 h post-transfection. Results represent two independent extractions of total RNA and RT-qPCR analysis performed in triplicate and normalized to GAPDH, mean ± SD. The t -test was used for statistical analyses.

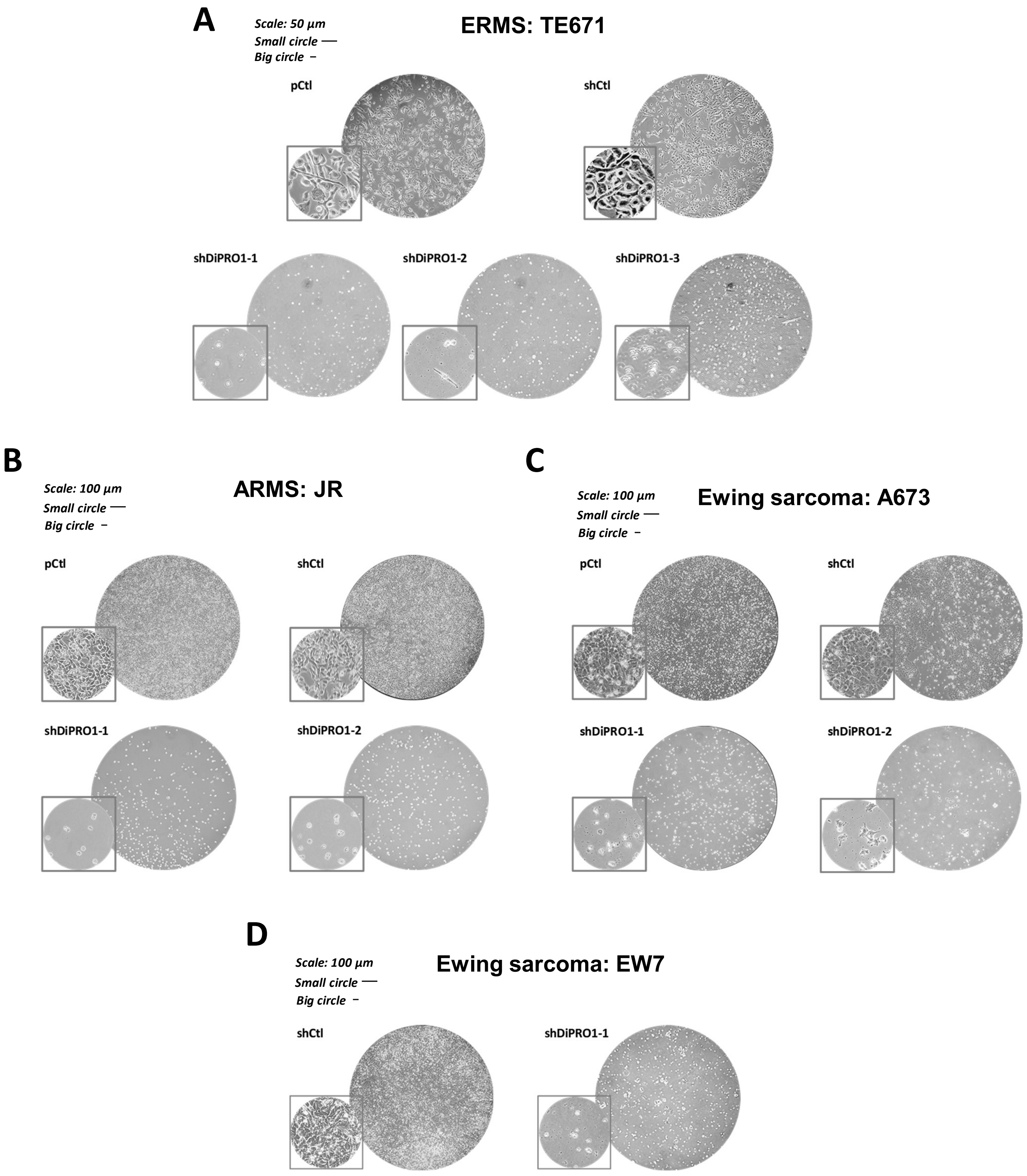

Appendix Figure S3. Depletion of DiPRO1 expression affects the survival of rhabdomyosarcoma and Ewing sarcoma cells.

Stable knockdown was achieved by transduction of lentiviral vectors producing non-targeting shRNA (shCtl) or DiPRO1-targeting shRNAs (shDiPRO1-1/2/3). DiPRO1 knockdown induces a dramatic cell death of RMS and Ewing's sarcoma cells 5-7 days after infection. Representative images of RMS TE671 (**A**), JR (**B**) and Ewing sarcoma A673 (**C**), EW7 (**D**) cells. The cell images with shDiPRO1-1 are represented in Fig. 3C. pCtl: untranfected parental cells, ARMS: alveolar rhabdomyosarcoma, ERMS: embryonal rhabdomyosarcoma.

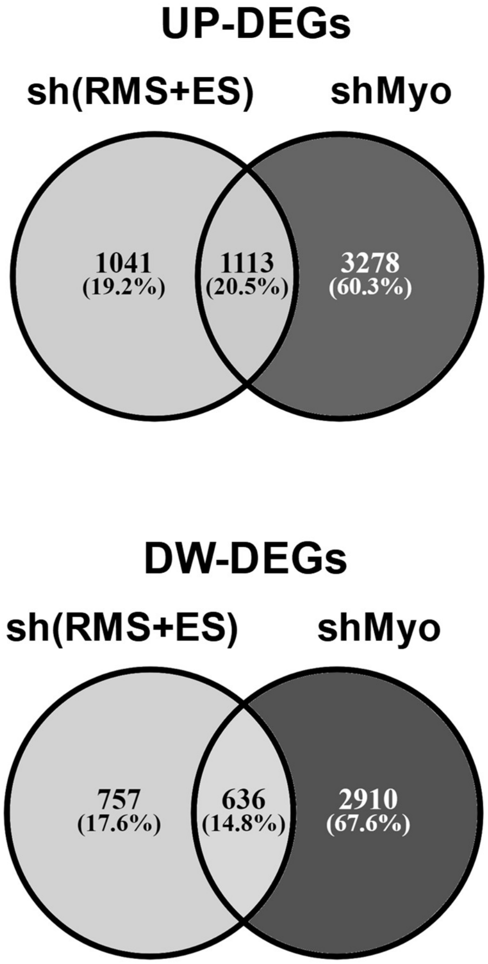

Appendix Figure S4. Overlap of differentially expressed genes (DEGs) in mesenchymal cancer cells and myoblasts following DiPRO1 KD.

DiPRO1 KD in RMS, Ewing sarcoma (ES) and myoblasts (Myo) cells was achieved by transduction of lentiviral vectors producing non-targeting shRNA (shCtl) or DiPRO1-targeting shRNAs (shDiPRO1). The resultes represent DEGs versus shCtl. UP: upregulated, DW: downregulated

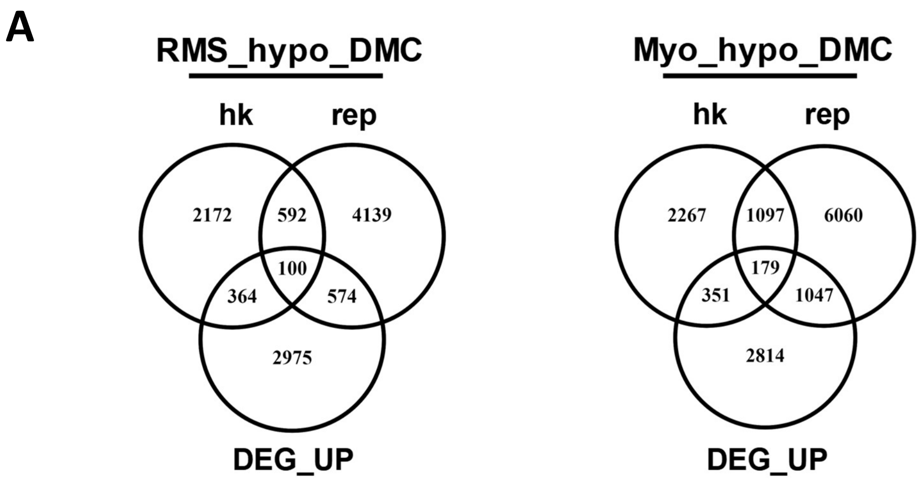

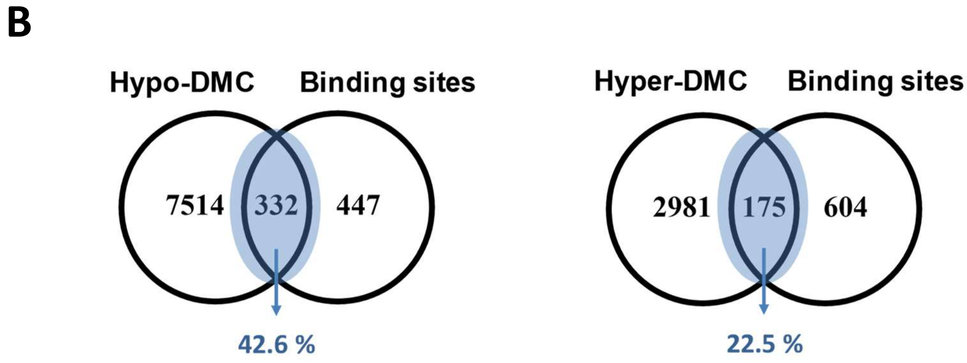

Appendix Figure S5. Impact of DiPRO1 knockdown on CpG island methylation in RMS and myoblast cells.

A, Venn diagram of overlapping genes at hypomethylated DMC associated with upregulated DEGs in RMS and myoblast (Myo) cells compared to control, P<0.05.

B, Venn diagrams overlapping genes associated with *cis*-regulatory binding sites for DiPRO1 and genes linked to the vicinity hypo-or hyper-DMC in RMS cells with DiPRO1 knockdown.

DMC, differentially methylated CGI; hk-DMC, housekeeping DMC w/o repetitive elements; rep-DMC, DMC with repetitive elements.

Appendix Table S1. Characteristics of the patients used in the study.

	All samples n=340	RMS n=37	ES n=25
Age (years)*	12.7±0.59	11.7±1.62	15.6±2.0
Gender			
Female	40%	43%	32%
Male	60%	57%	68%
Treatment outcome			
Refractory	31%	32%	24%
Relapse	68%	68%	76%

* median ± CI (95%)

Appendix Table S2. Disease groups and tumor types in the study cohort.

DISEASE GROUP	CNS TUMORS	LEUKEMIAS	LYMPHOMAS	SARCOMAS	OTHER SOLID TUMORS
TUMOR TYPE	High-grade glioma	Acute lymphoblastic leukemia	Anaplastic large-cell lymphoma	Osteosarcoma	Neuroblastoma
	Low-grade glioma	Acute myeloid leukemia	Other Non-Hodgkin Lymphoma	Non-rhabdmyosarcoma soft tissue sarcoma	Wilms' tumor
	Medulloblastoma			Ewing sarcoma	Hepatoblastoma
	Ependymoma			Rhabdomyosarcoma	Carcinoma
	Other CNS tumor				Other solid tumor
PATIENT NUMBER	104	27	9	129	58

Appendix Table S3. List of primers used for transcription analysis.

Sequence ID	Forward	Reverse
DiPRO1endo	TGAGCAGAATCCAGCAAAGCCCA	CCGCCTGCCCCTAGCGGTCC
DiPRO1exo	TCAATTTAAGGCCAGCGGGA	CCCAGCTCACGTTCCTTGTA
MYF5	ATGCCATCCGCTACATCGAG	TGCCATCAGAGCAGTTGGAG
MYOD1	CTCTCTGCTCCTTTGCCACA	AGTGCTCTTCGGGTTTCAGG
MYOG	GTGCCATCCAGTACATCGAGC	GCAGATGATCCCCTGGGTTG
MYH1	GAGTCCATGCAGAGCACACT	GCTGTTCCTTCAGGTCCTCC
GAPDH	CCAGGTGGTCTCCTCTGACT	AAGTGGTCGTTGAGGGCAAT
