# Supplementary material for: DiPRO1 distinctly reprograms muscle and mesenchymal cancer cells
Source: EMBO Mol Med. 2024 Jul 15;16(8):4. doi: 10.1038/s44321-024-00097-z (PMC11319797; doi:10.1038/s44321-024-00097-z)
Supplement: Supplementary file 9 — Movie EV1 [file 44321_2024_97_MOESM9_ESM.zip › Movie EV1.docx]

**Movie EV1. Uptake of siDiPRO1/jetPEI®/Cy5 nanocomposites by tumor cells**

A representative movie depicted a nude mouse that received subcutaneous inoculations of A673 Ewing sarcoma cells and was subsequently treated with a single dose (0.5 mg/kg) of Cy5-coupled siDiPRO1/jetPEI® nanocomposite via intratumoral administration. Internalization was monitored in the live mouse 72 hours after treatment.
