# Supplementary figures and images for: DiPRO1 distinctly reprograms muscle and mesenchymal cancer cells (part 1 of 2)
Source: EMBO Mol Med. 2024 Jul 15;16(8):4. doi: 10.1038/s44321-024-00097-z (PMC11319797; doi:10.1038/s44321-024-00097-z)

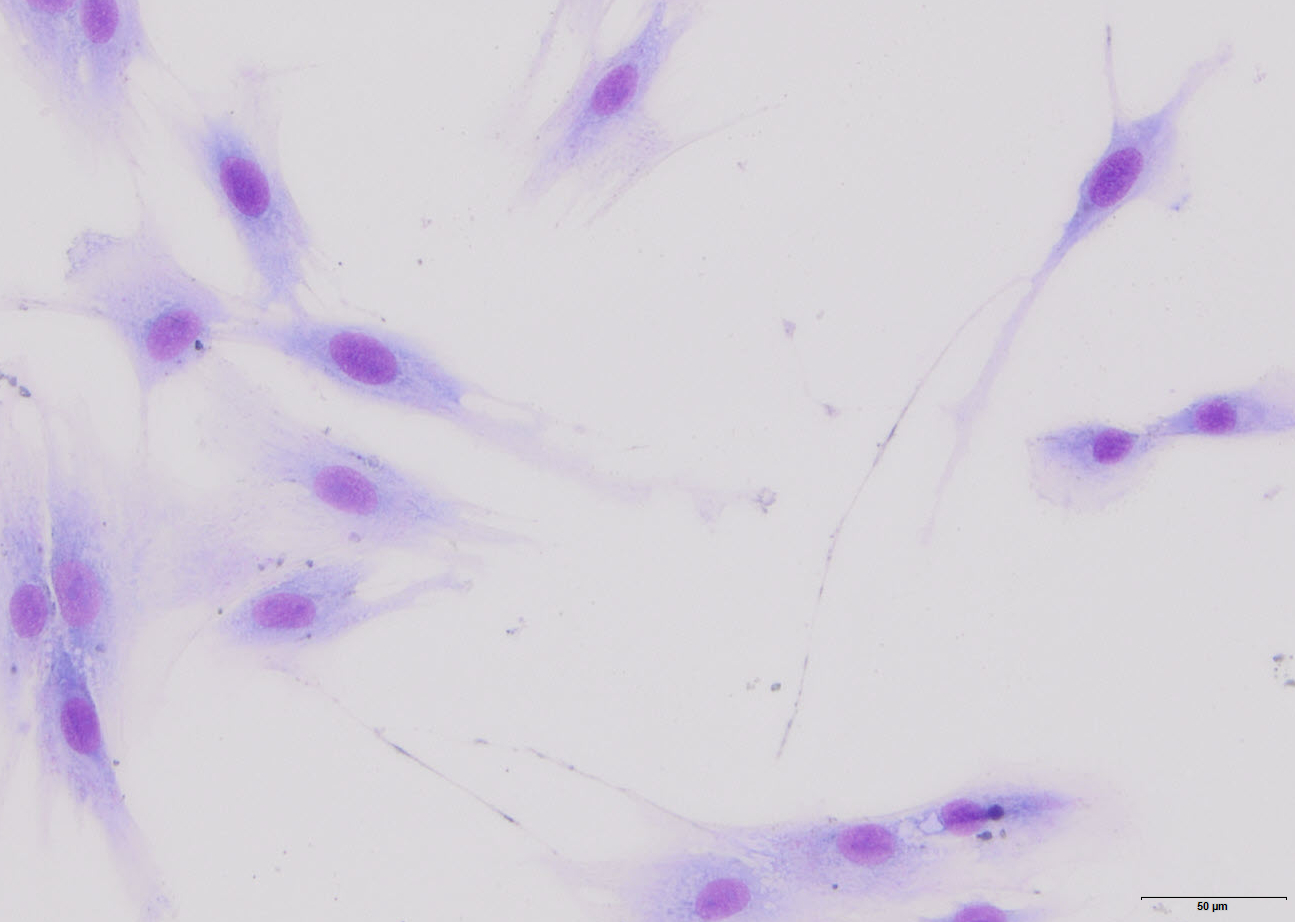

Supplement: Supplementary file 10 — Source data Fig. 2 [file 44321_2024_97_MOESM10_ESM.zip › Fig.2/Fig_2C/MGG_Myo_pCtl-50μm-1.jpg]

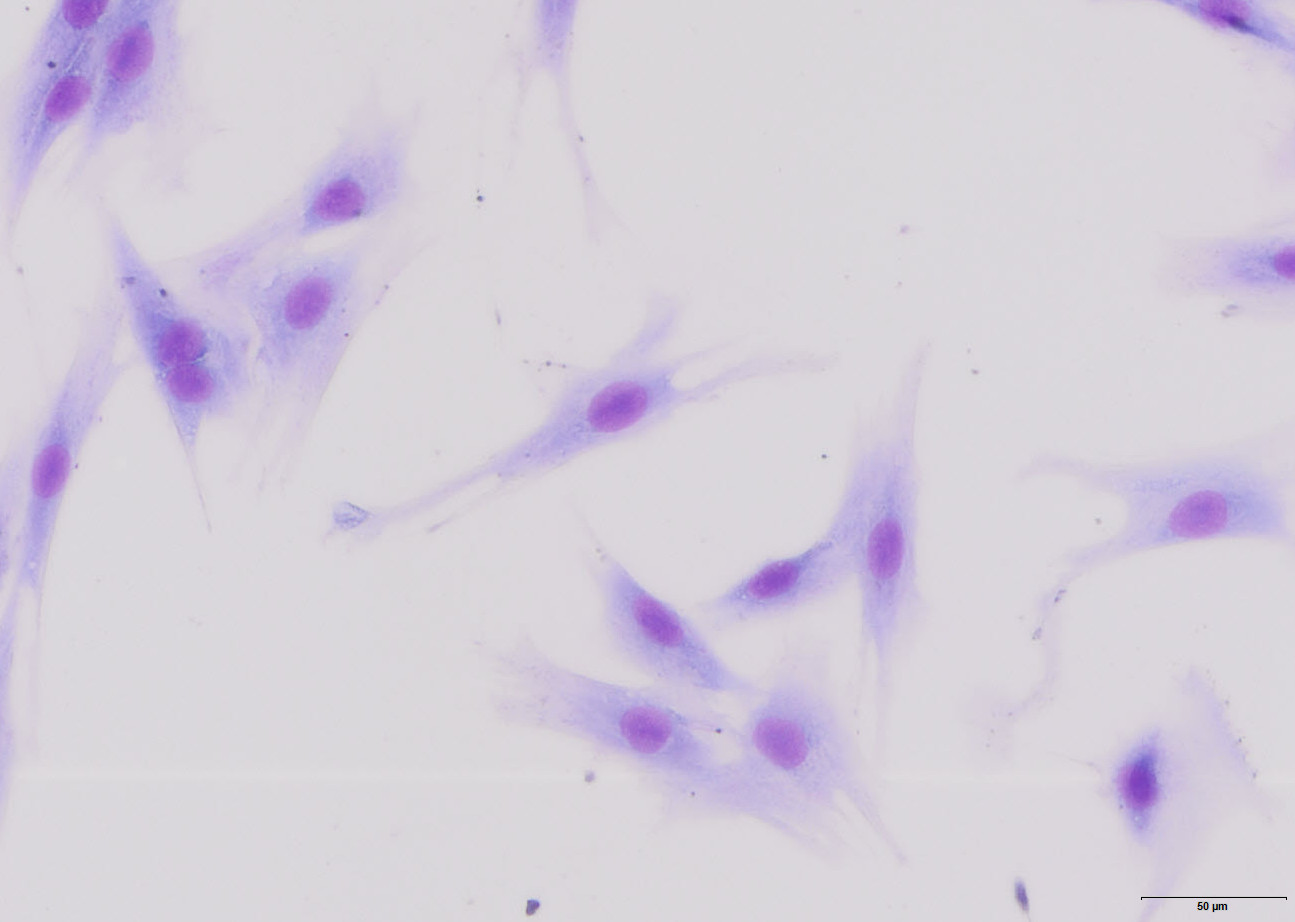

Supplement: Supplementary file 10 — Source data Fig. 2 [file 44321_2024_97_MOESM10_ESM.zip › Fig.2/Fig_2C/MGG_Myo_pCtl-50μm-2.jpg]

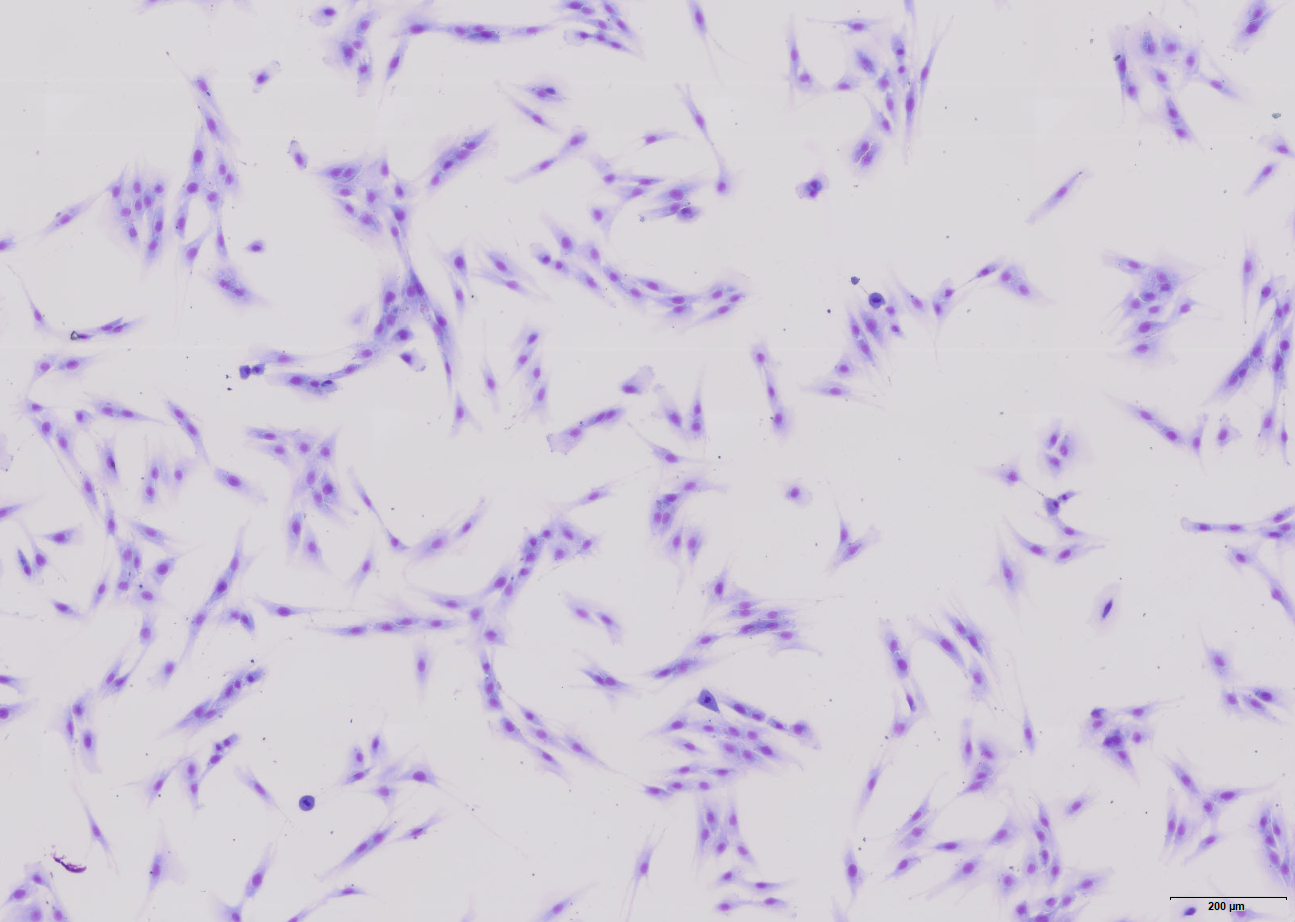

Supplement: Supplementary file 10 — Source data Fig. 2 [file 44321_2024_97_MOESM10_ESM.zip › Fig.2/Fig_2C/MGG_Myo_pCtl_200μm.jpg]

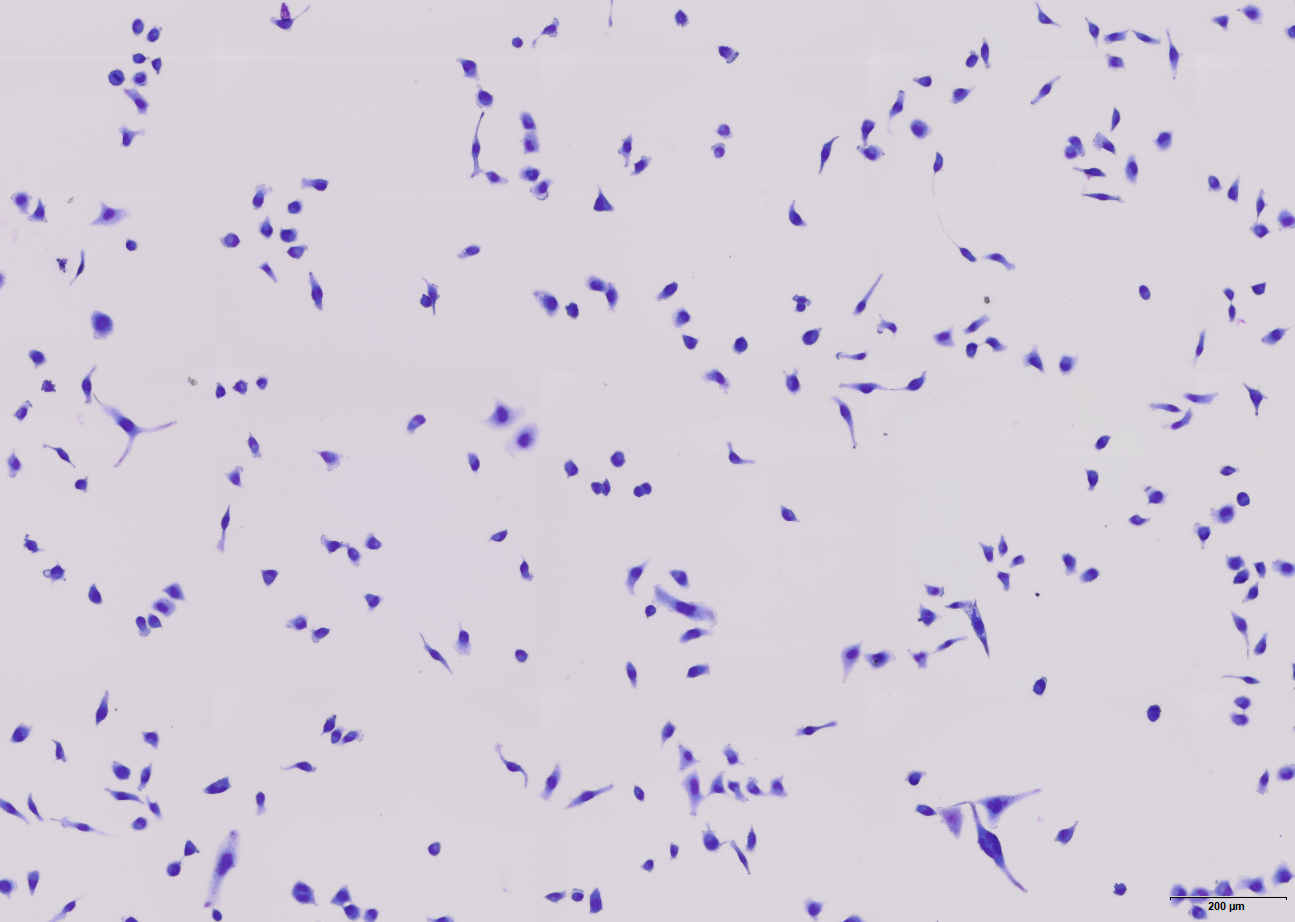

Supplement: Supplementary file 10 — Source data Fig. 2 [file 44321_2024_97_MOESM10_ESM.zip › Fig.2/Fig_2C/MGG_Myo_pDiPRO1_200μm.jpg]

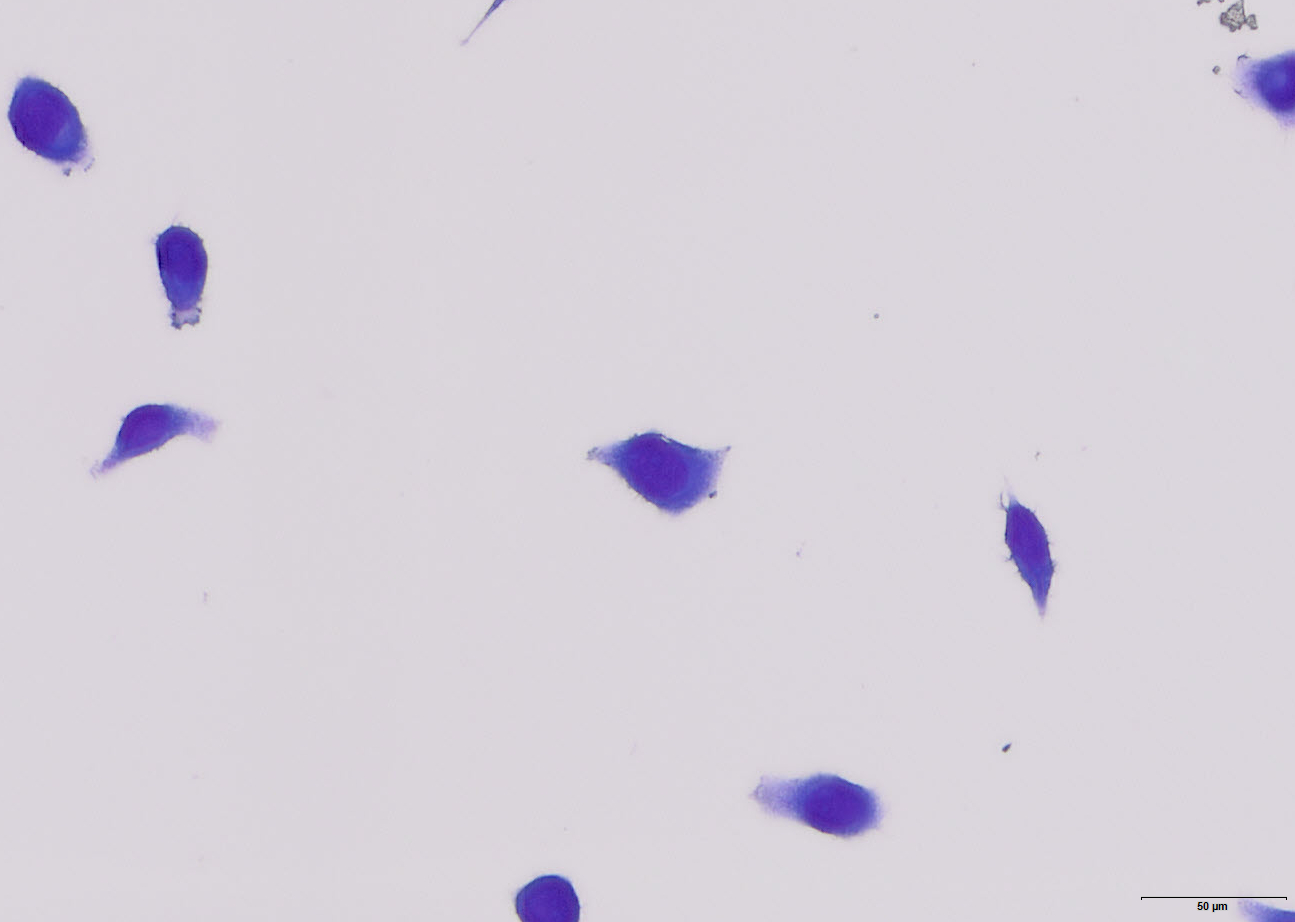

Supplement: Supplementary file 10 — Source data Fig. 2 [file 44321_2024_97_MOESM10_ESM.zip › Fig.2/Fig_2C/MGG_Myo_pDiPRO1_50μm-1.jpg]

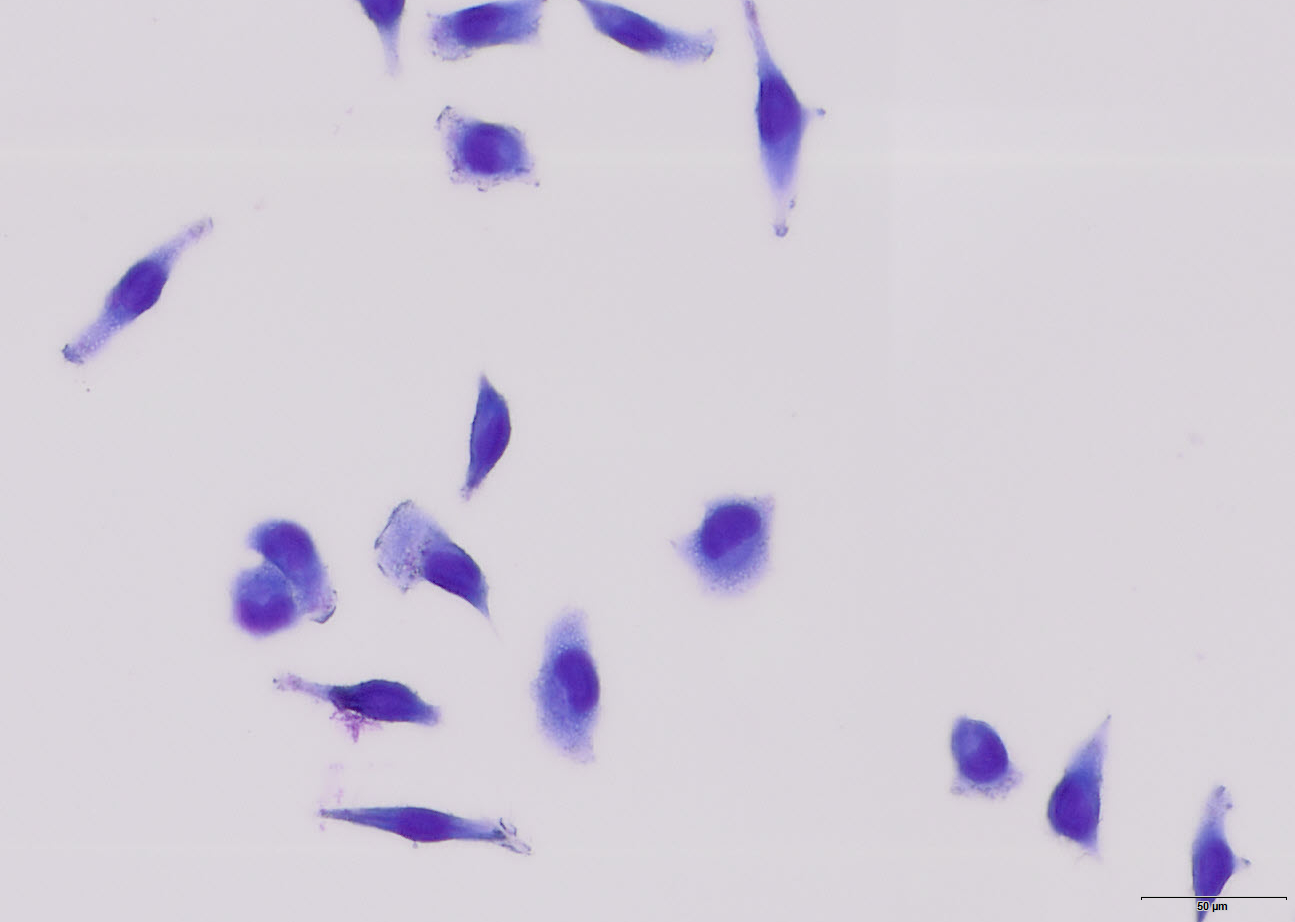

Supplement: Supplementary file 10 — Source data Fig. 2 [file 44321_2024_97_MOESM10_ESM.zip › Fig.2/Fig_2C/MGG_Myo_pDiPRO1_50μm-2.jpg]

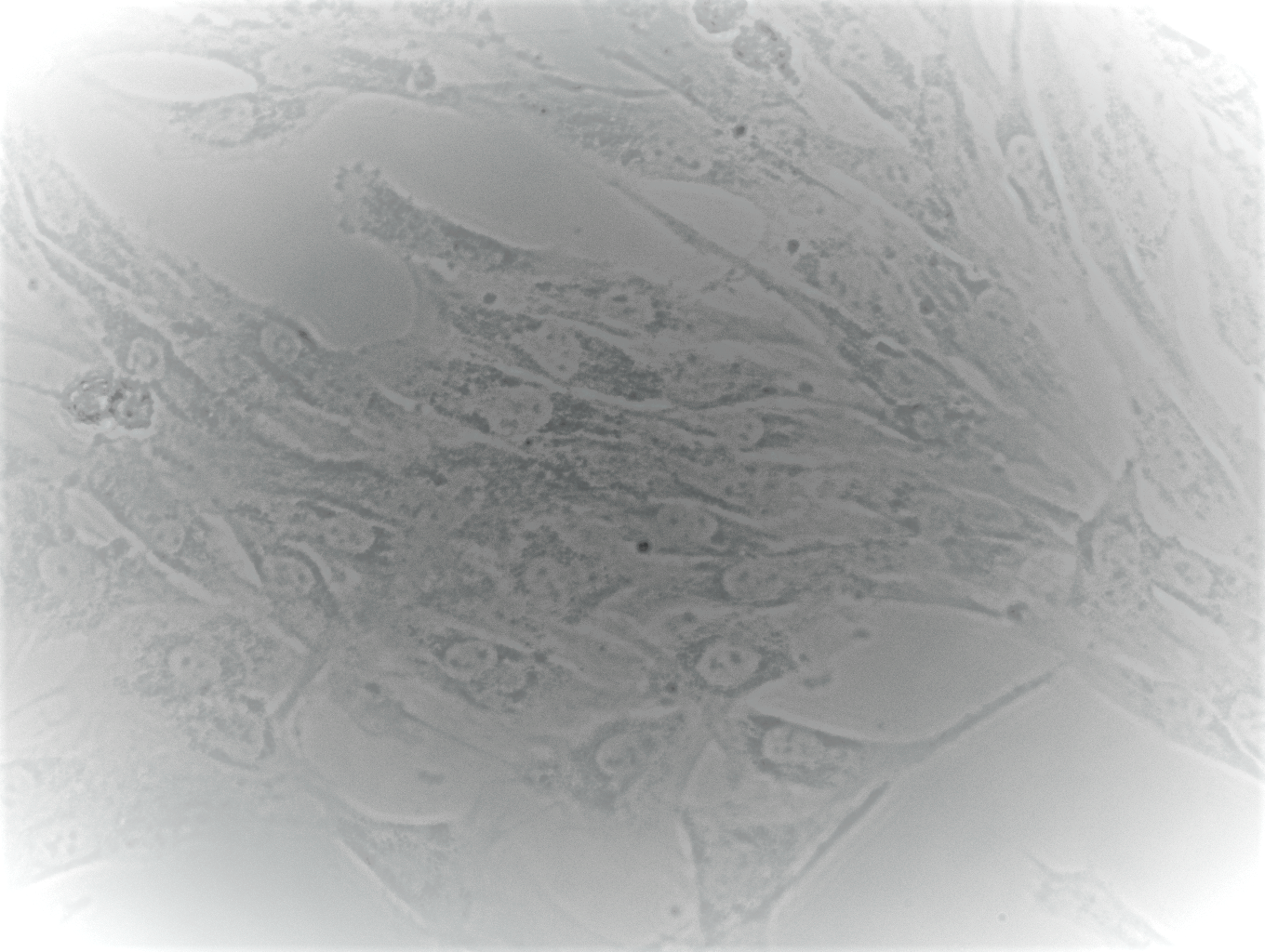

Supplement: Supplementary file 10 — Source data Fig. 2 [file 44321_2024_97_MOESM10_ESM.zip › Fig.2/Fig_2C/Real_Myo_pCtl-1.tif]

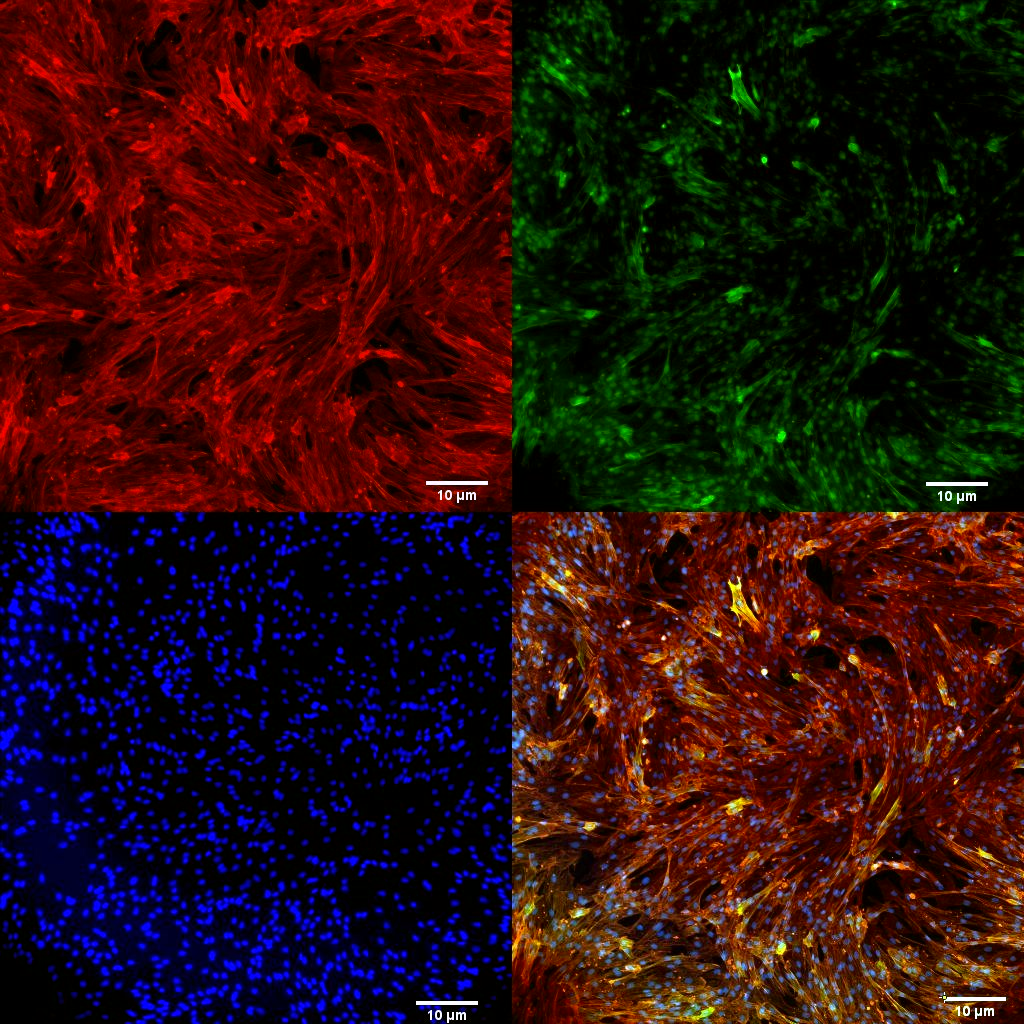

Supplement: Supplementary file 10 — Source data Fig. 2 [file 44321_2024_97_MOESM10_ESM.zip › Fig.2/Fig_2F/Dif_Myo_Ctl.tif]

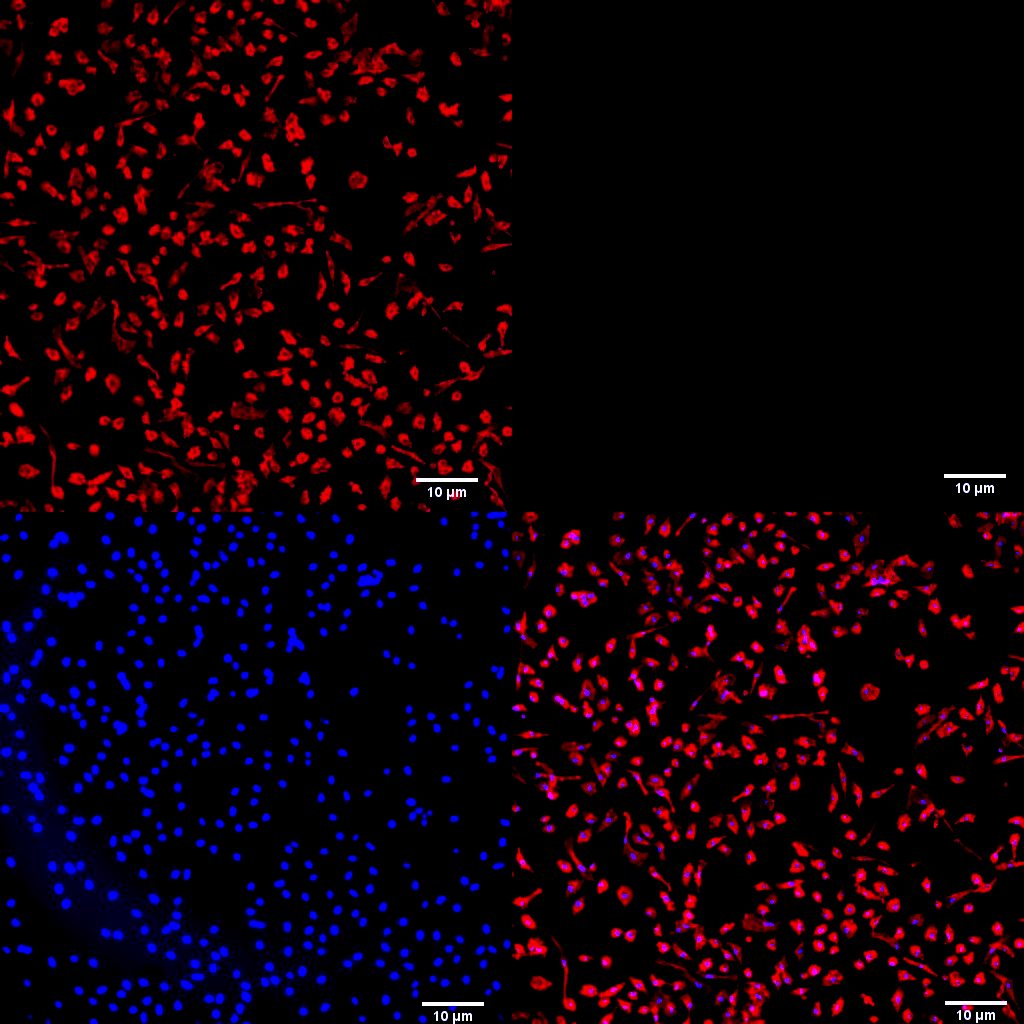

Supplement: Supplementary file 10 — Source data Fig. 2 [file 44321_2024_97_MOESM10_ESM.zip › Fig.2/Fig_2F/Dif_Myo_pDIPRO1.tif]

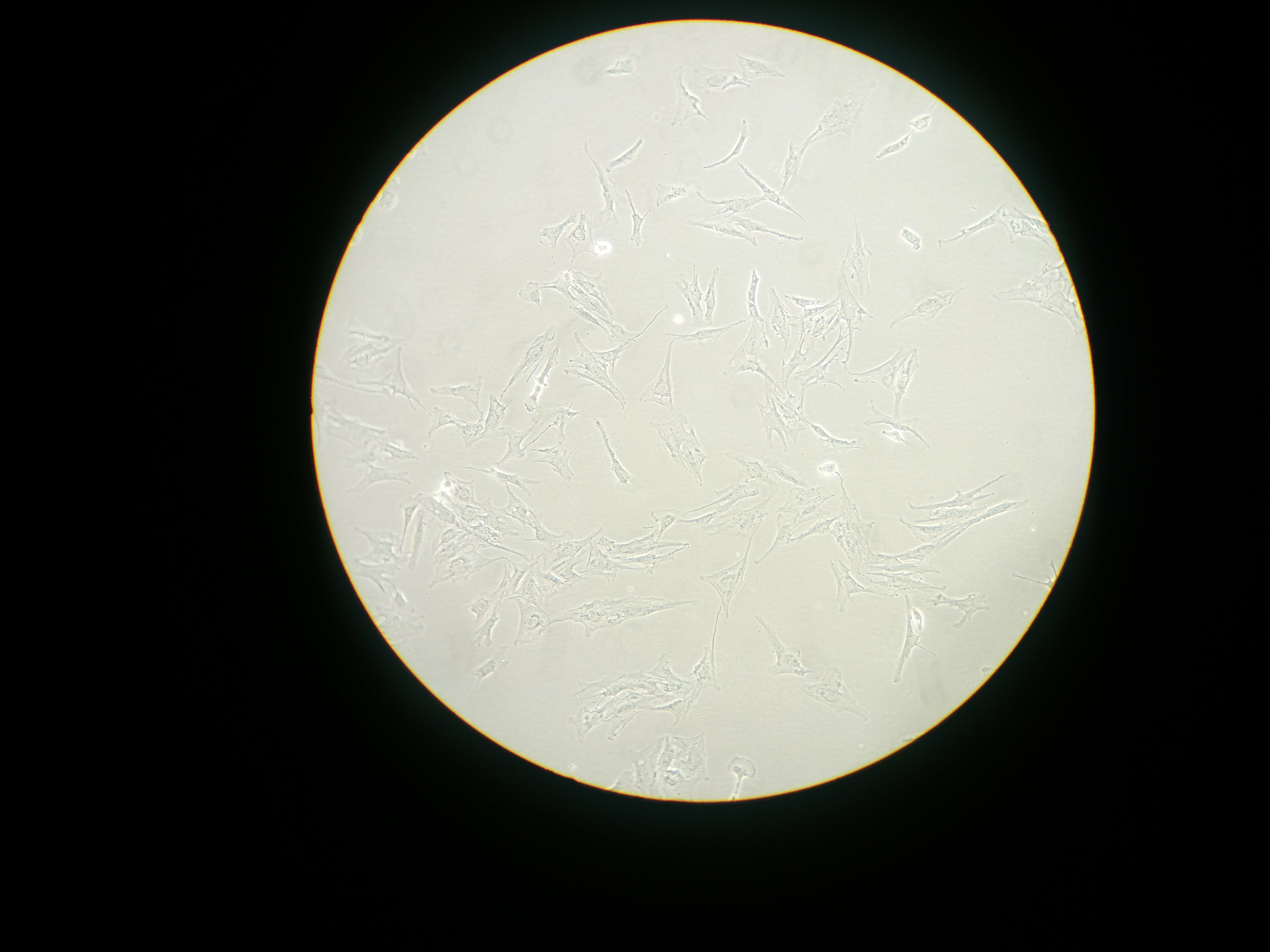

Supplement: Supplementary file 10 — Source data Fig. 2 [file 44321_2024_97_MOESM10_ESM.zip › Fig.2/Fig_2I/Myo_shCtl-1.jpg]

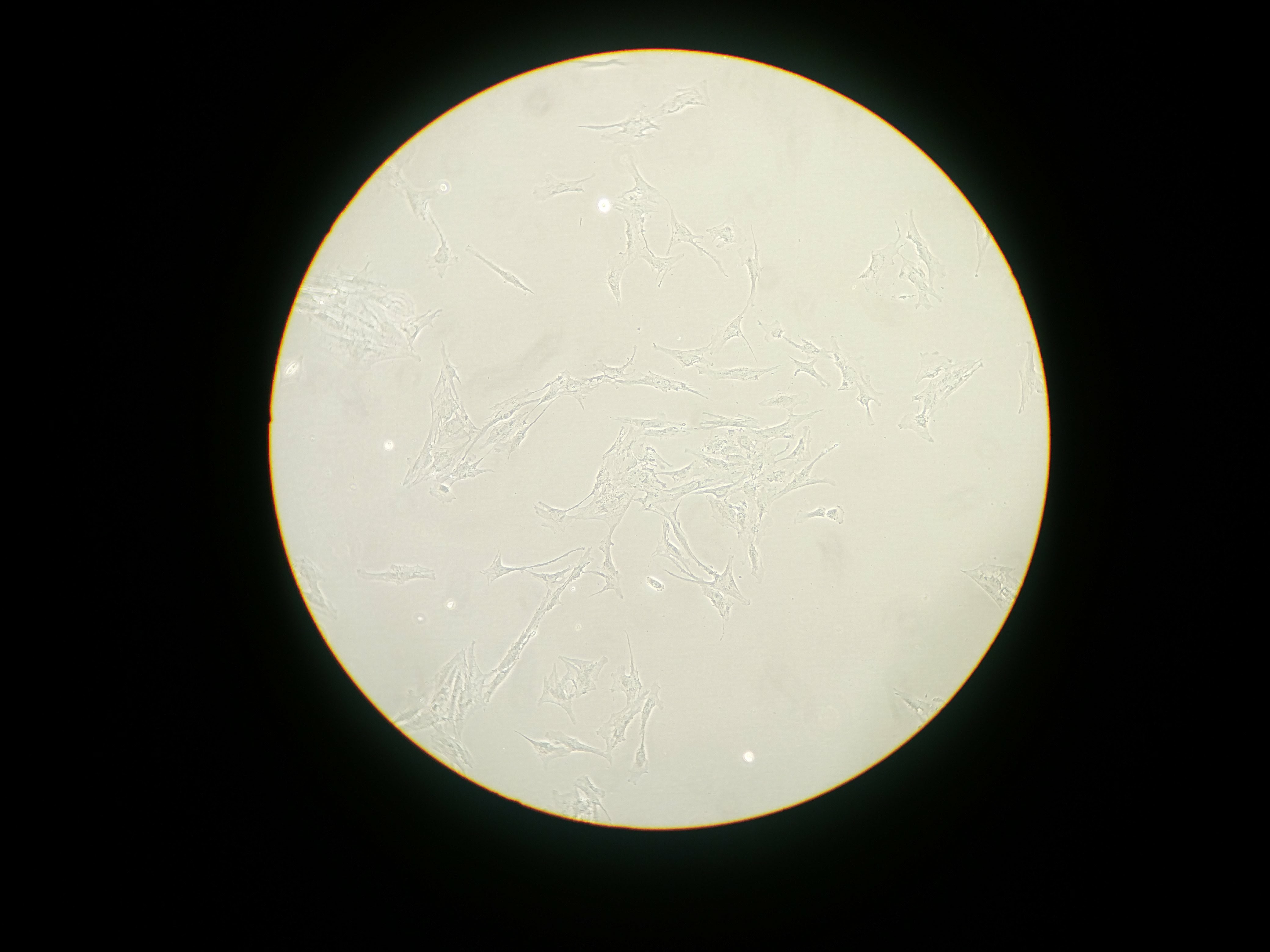

Supplement: Supplementary file 10 — Source data Fig. 2 [file 44321_2024_97_MOESM10_ESM.zip › Fig.2/Fig_2I/Myo_shCtl-2.jpg]

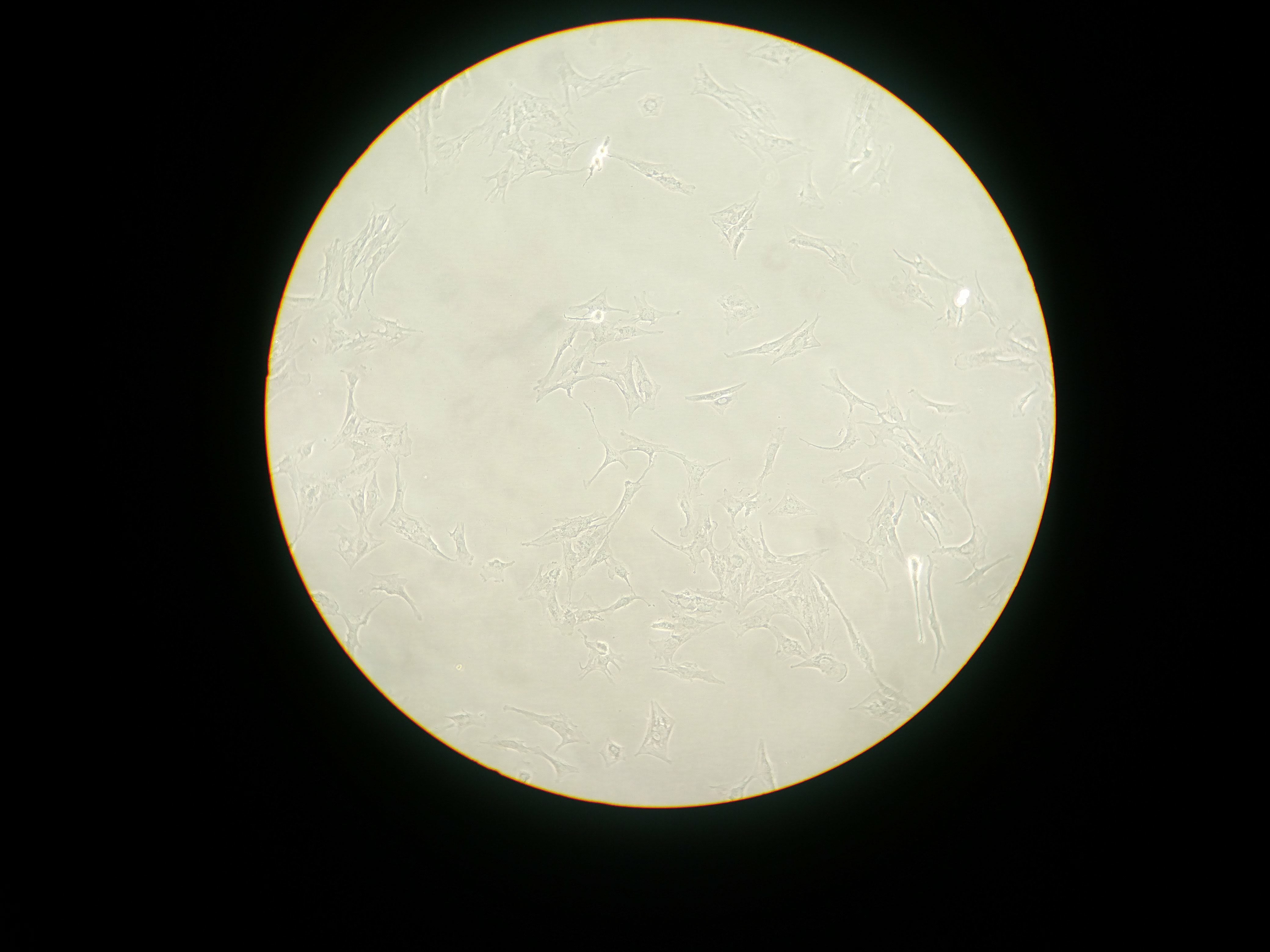

Supplement: Supplementary file 10 — Source data Fig. 2 [file 44321_2024_97_MOESM10_ESM.zip › Fig.2/Fig_2I/Myo_shCtl-3.jpg]

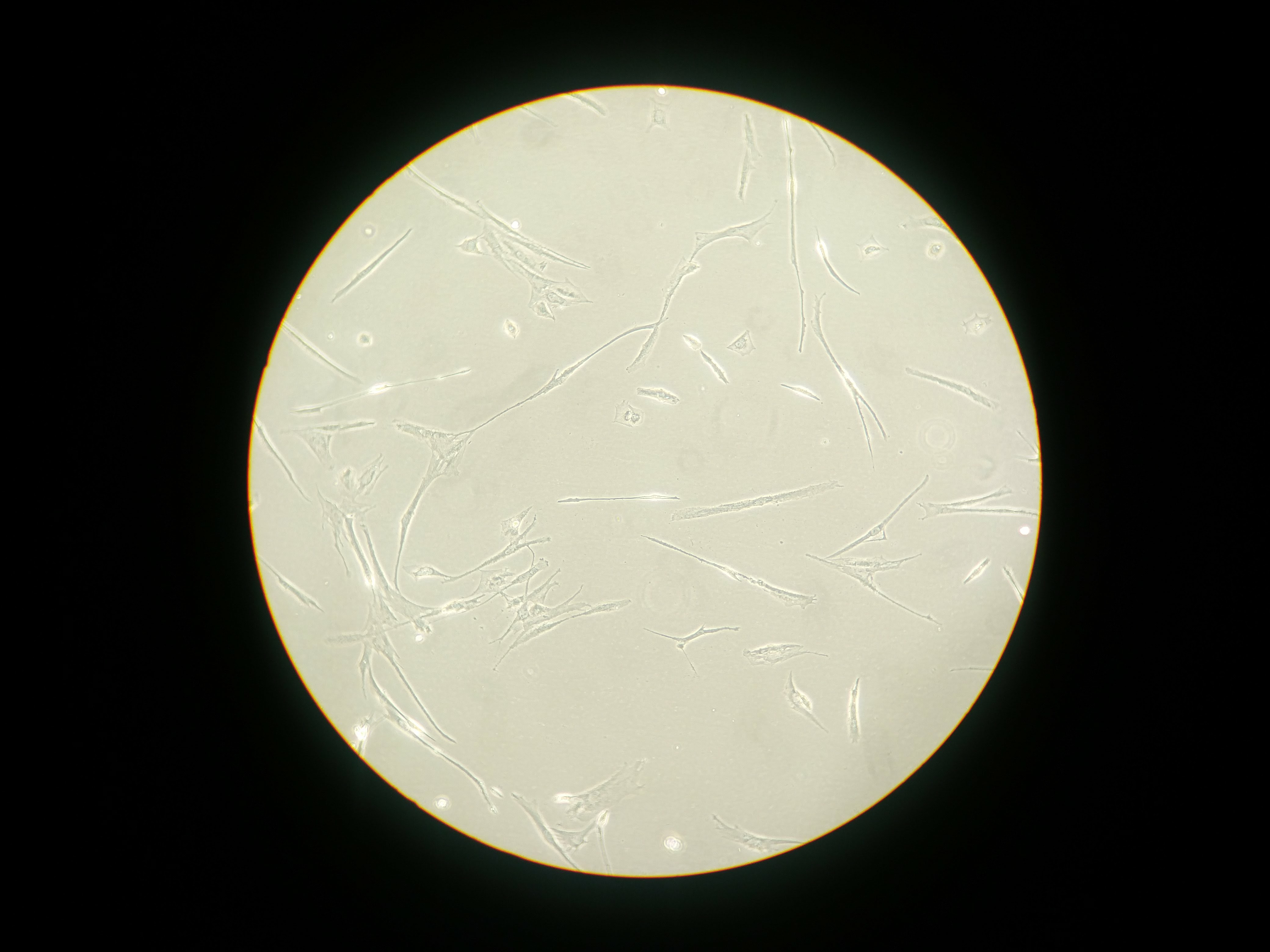

Supplement: Supplementary file 10 — Source data Fig. 2 [file 44321_2024_97_MOESM10_ESM.zip › Fig.2/Fig_2I/Myo_shDiPRO-1.jpg]

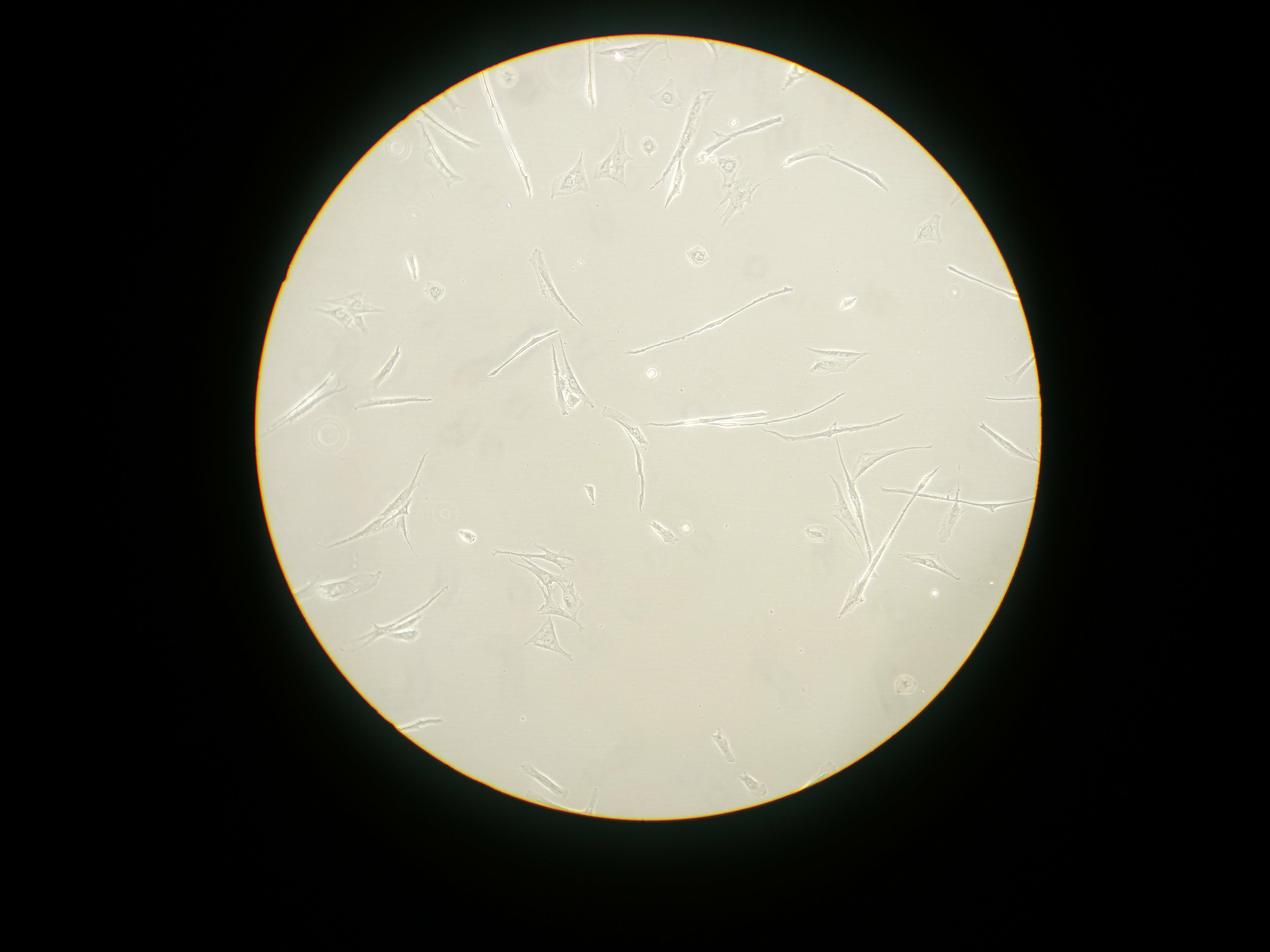

Supplement: Supplementary file 10 — Source data Fig. 2 [file 44321_2024_97_MOESM10_ESM.zip › Fig.2/Fig_2I/Myo_shDiPRO-2.jpg]

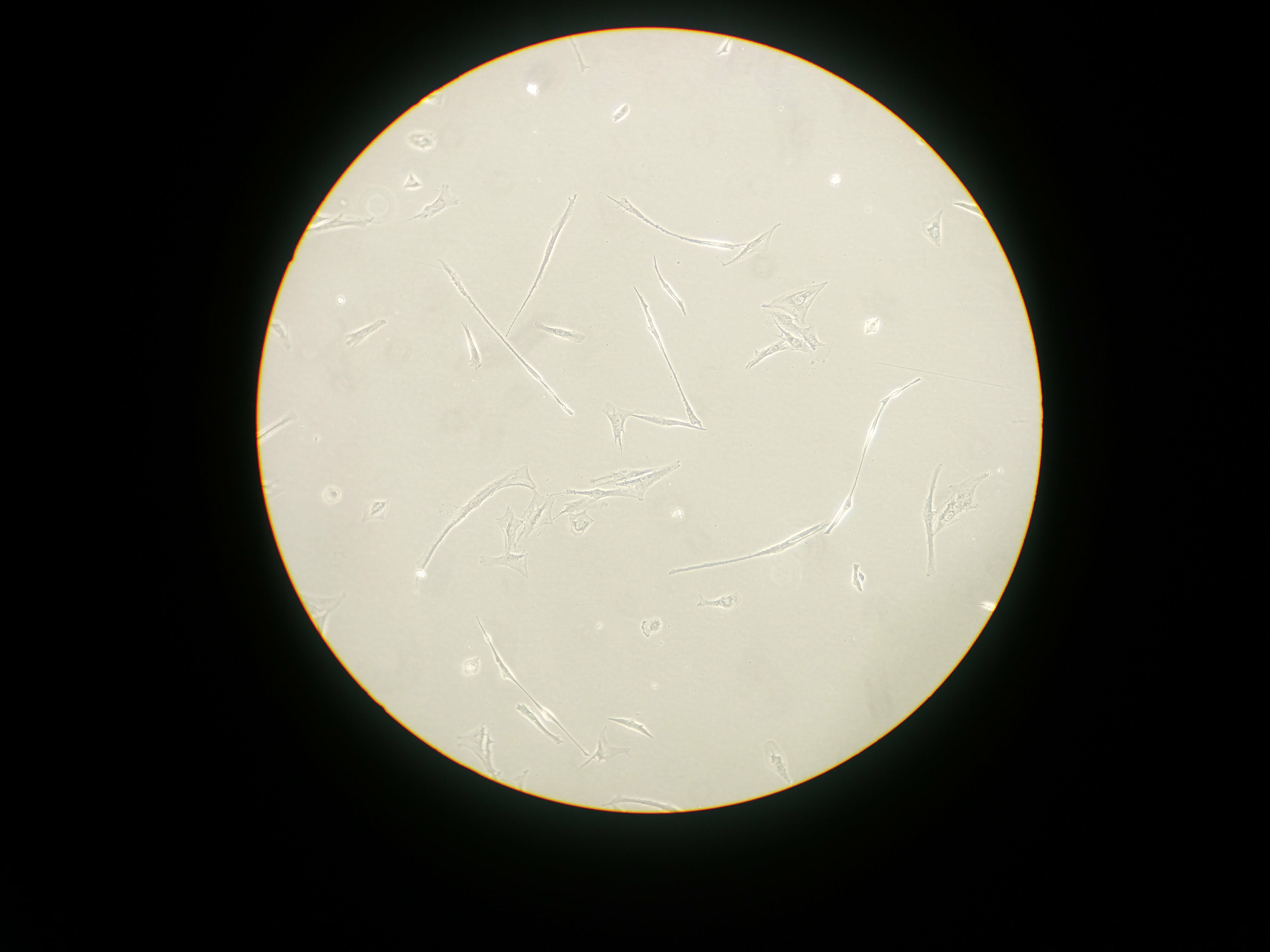

Supplement: Supplementary file 10 — Source data Fig. 2 [file 44321_2024_97_MOESM10_ESM.zip › Fig.2/Fig_2I/Myo_shDiPRO-3.jpg]

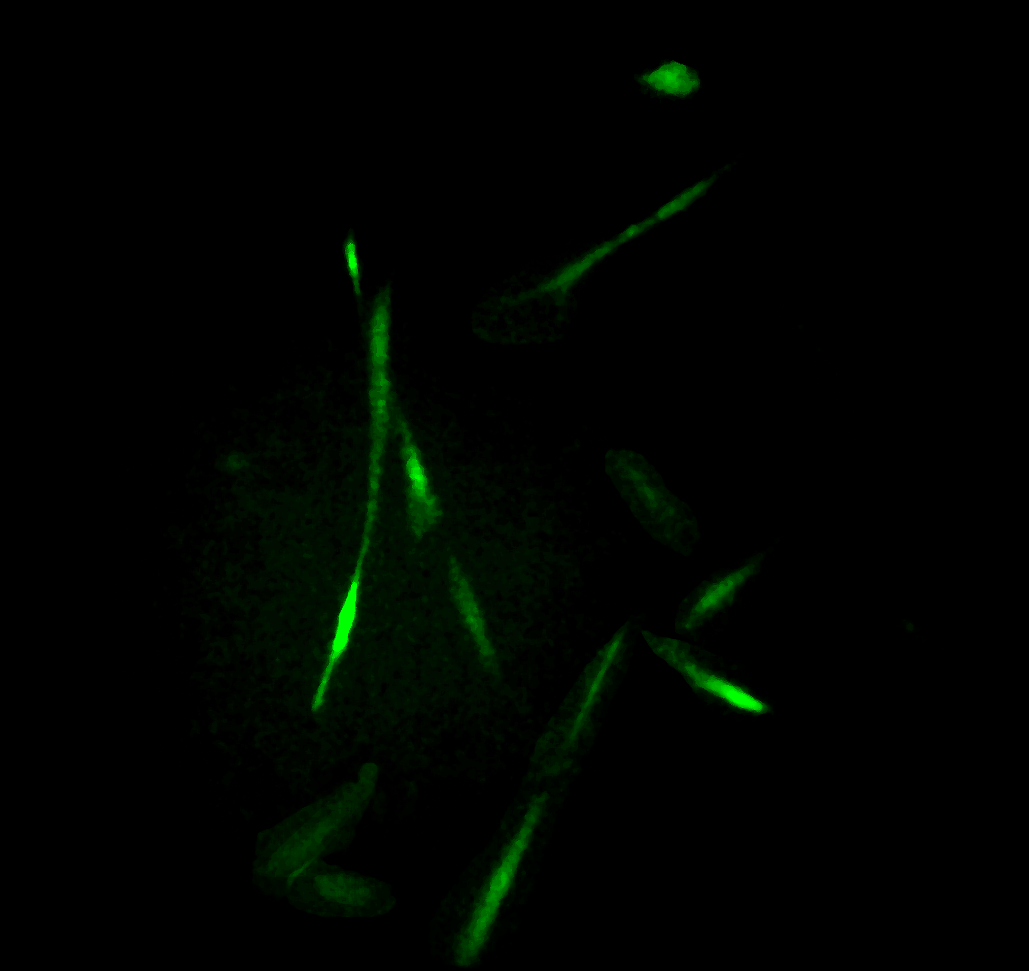

Supplement: Supplementary file 10 — Source data Fig. 2 [file 44321_2024_97_MOESM10_ESM.zip › Fig.2/Fig_2I/Myo_shDiPRO1_GFP.png]

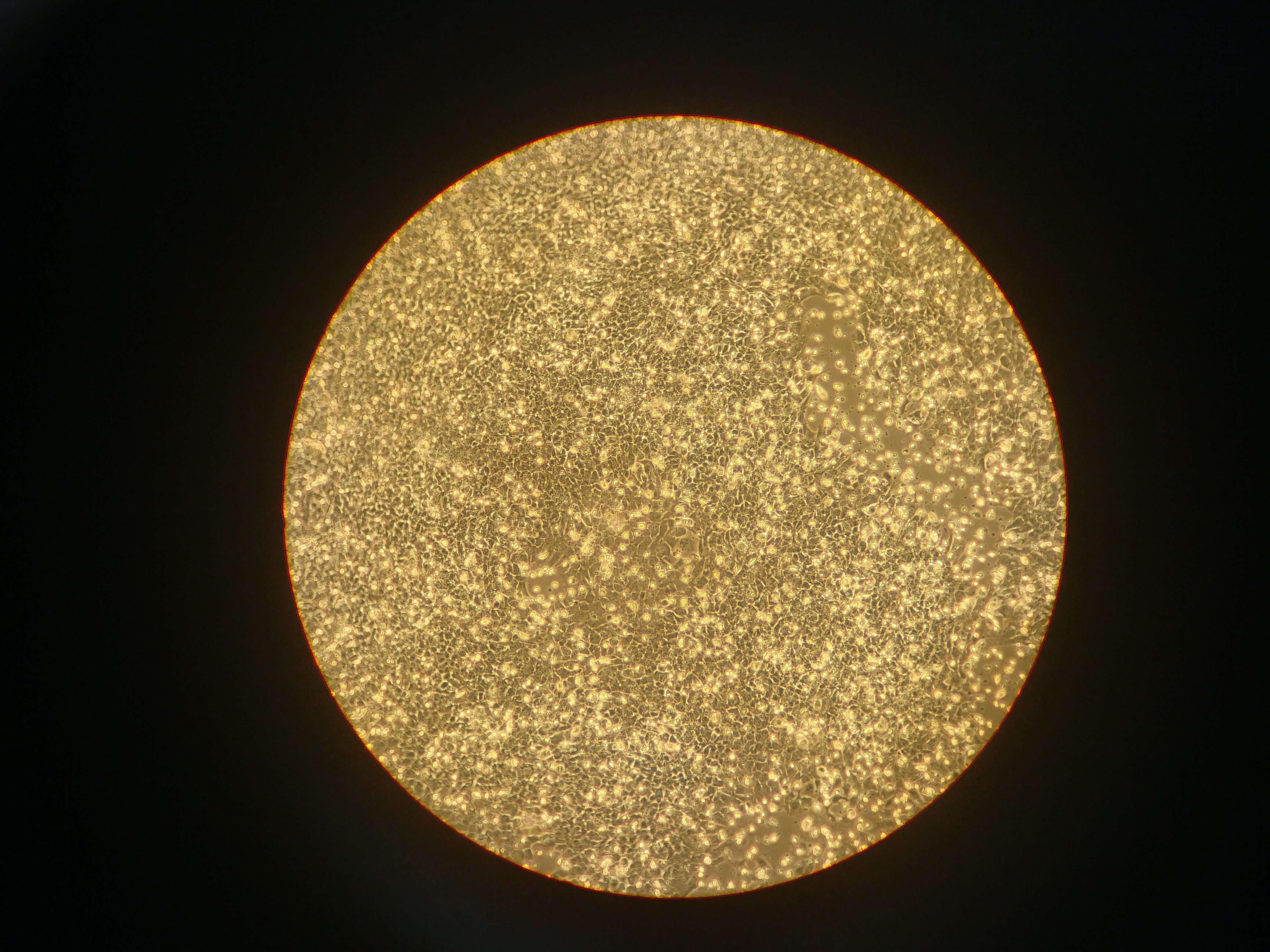

Supplement: Supplementary file 11 — Source data Fig. 3 [file 44321_2024_97_MOESM11_ESM.zip › Fig 3/Fig_3C/A673-shCTL/A673_pCTL.jpg]

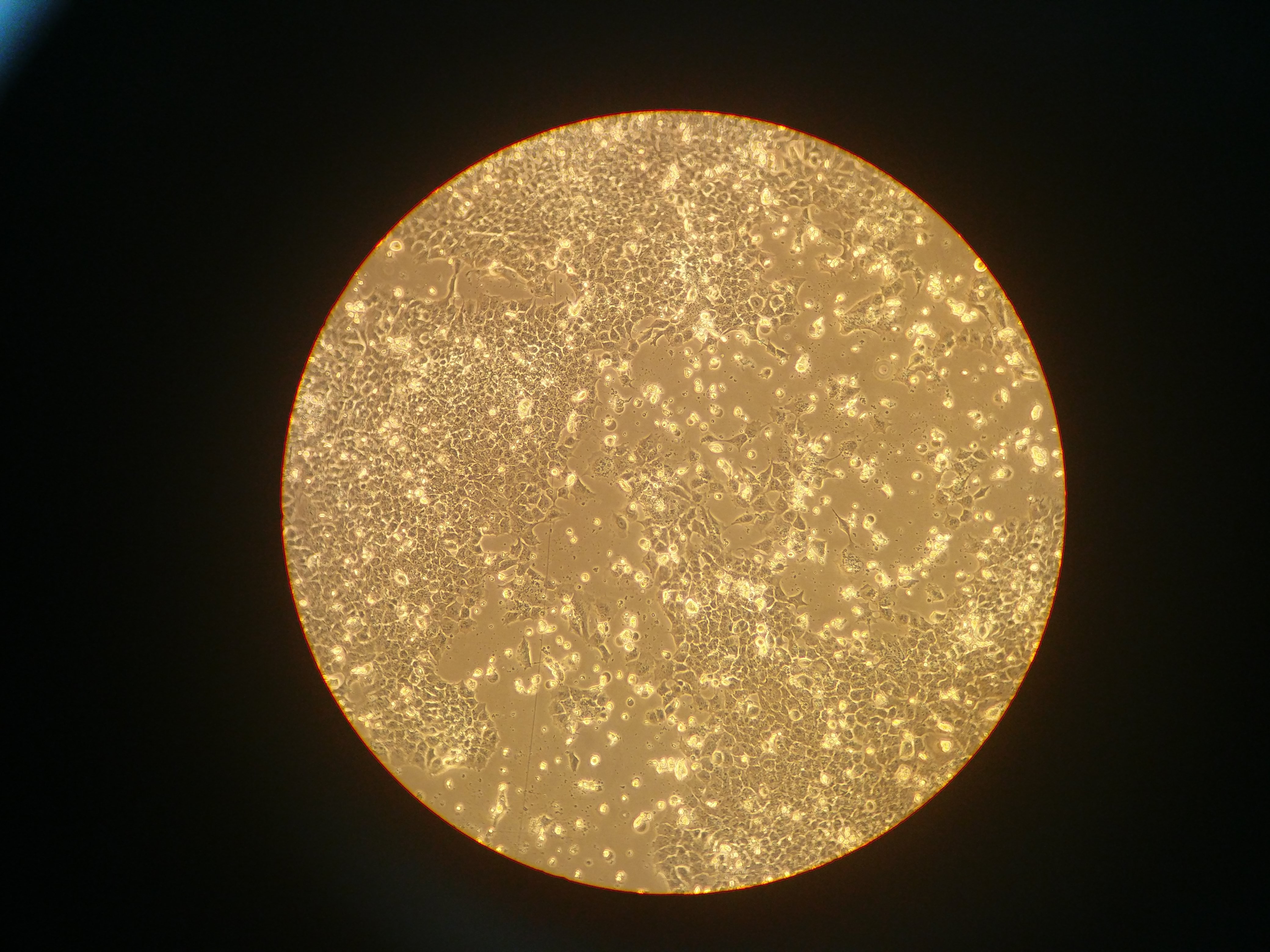

Supplement: Supplementary file 11 — Source data Fig. 3 [file 44321_2024_97_MOESM11_ESM.zip › Fig 3/Fig_3C/A673-shCTL/A673_shCTL-1.jpg]

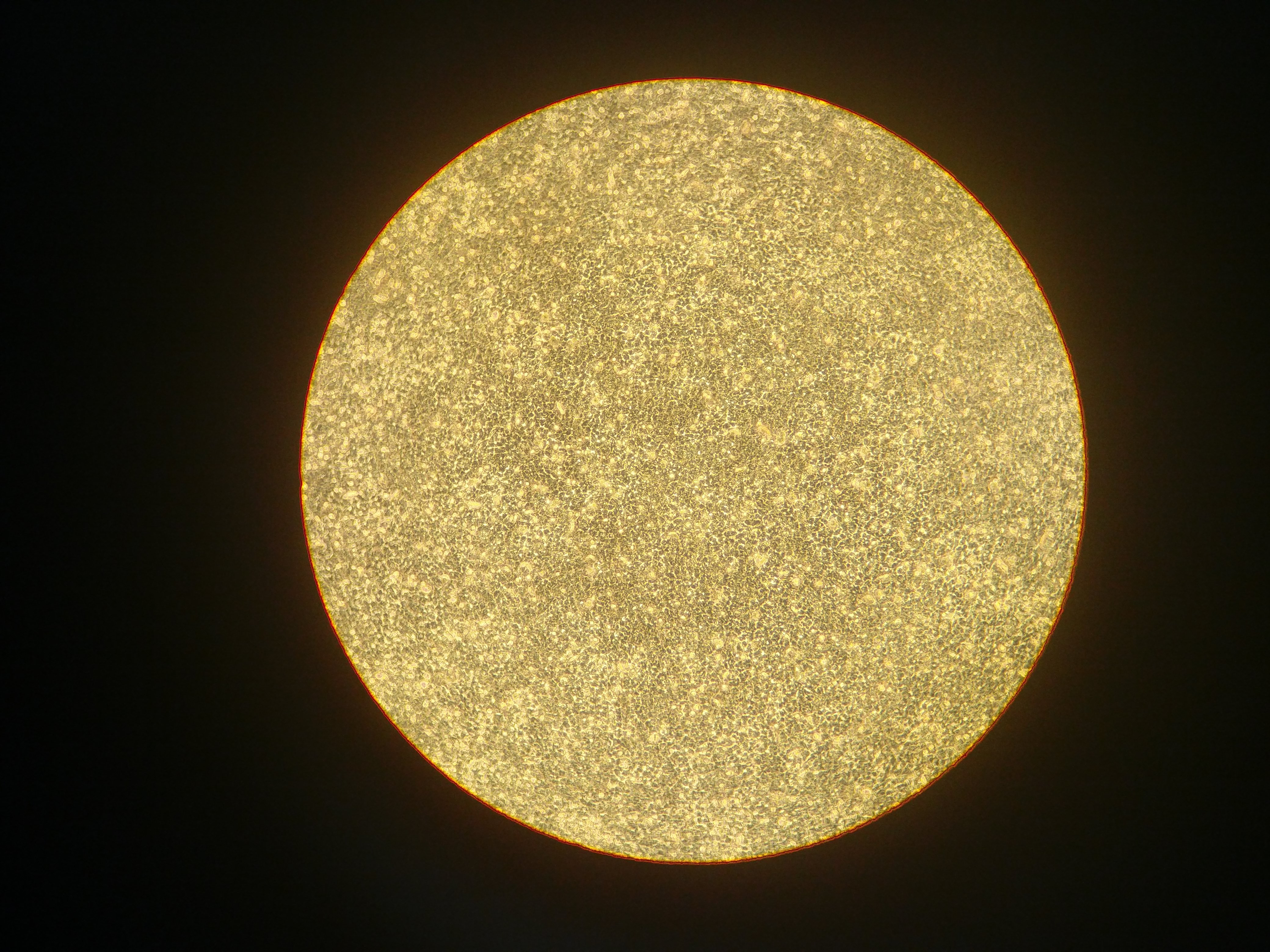

Supplement: Supplementary file 11 — Source data Fig. 3 [file 44321_2024_97_MOESM11_ESM.zip › Fig 3/Fig_3C/A673-shCTL/A673_shCTL-2.jpg]

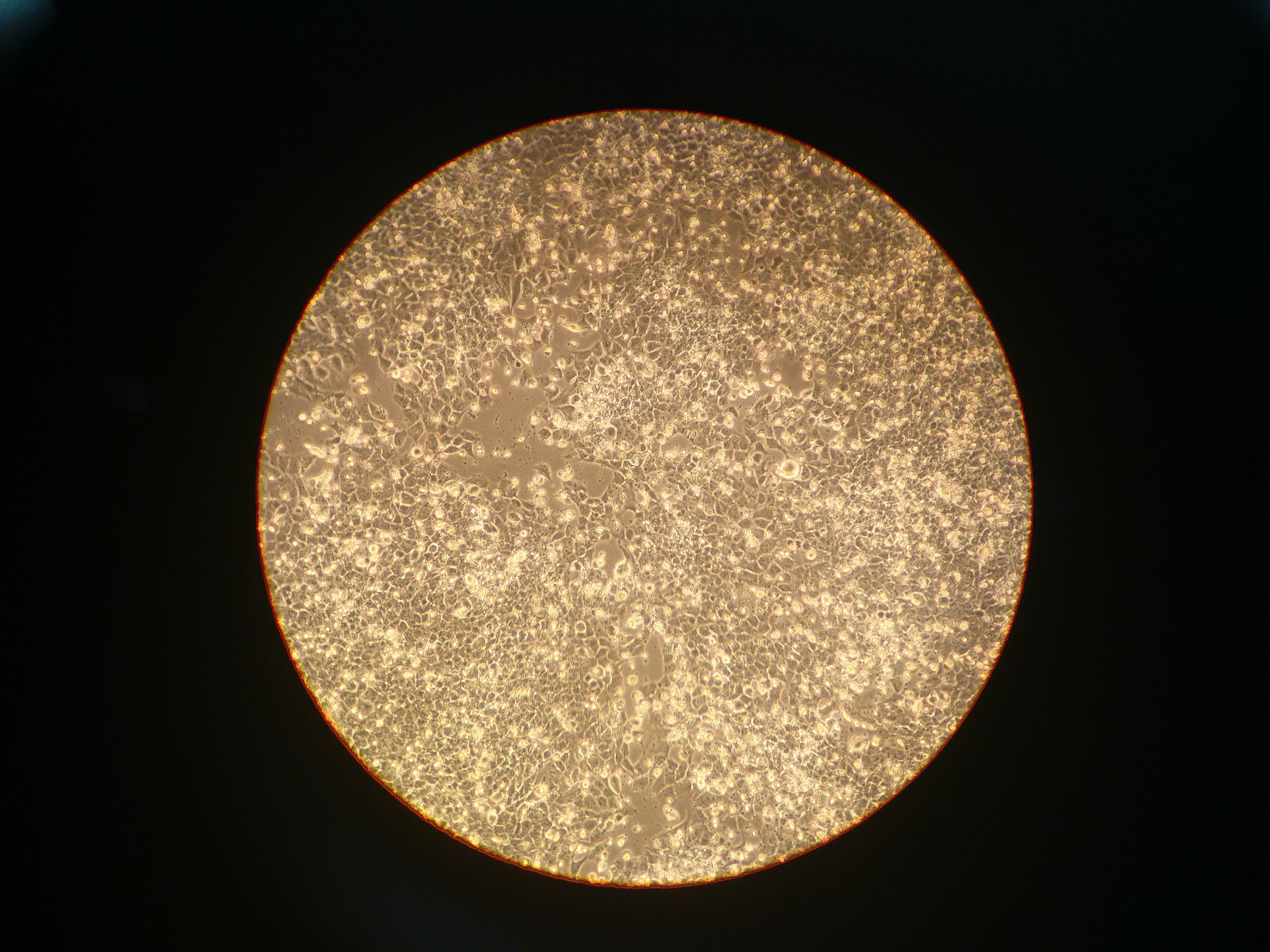

Supplement: Supplementary file 11 — Source data Fig. 3 [file 44321_2024_97_MOESM11_ESM.zip › Fig 3/Fig_3C/A673-shCTL/A673_shCTL-3.jpg]

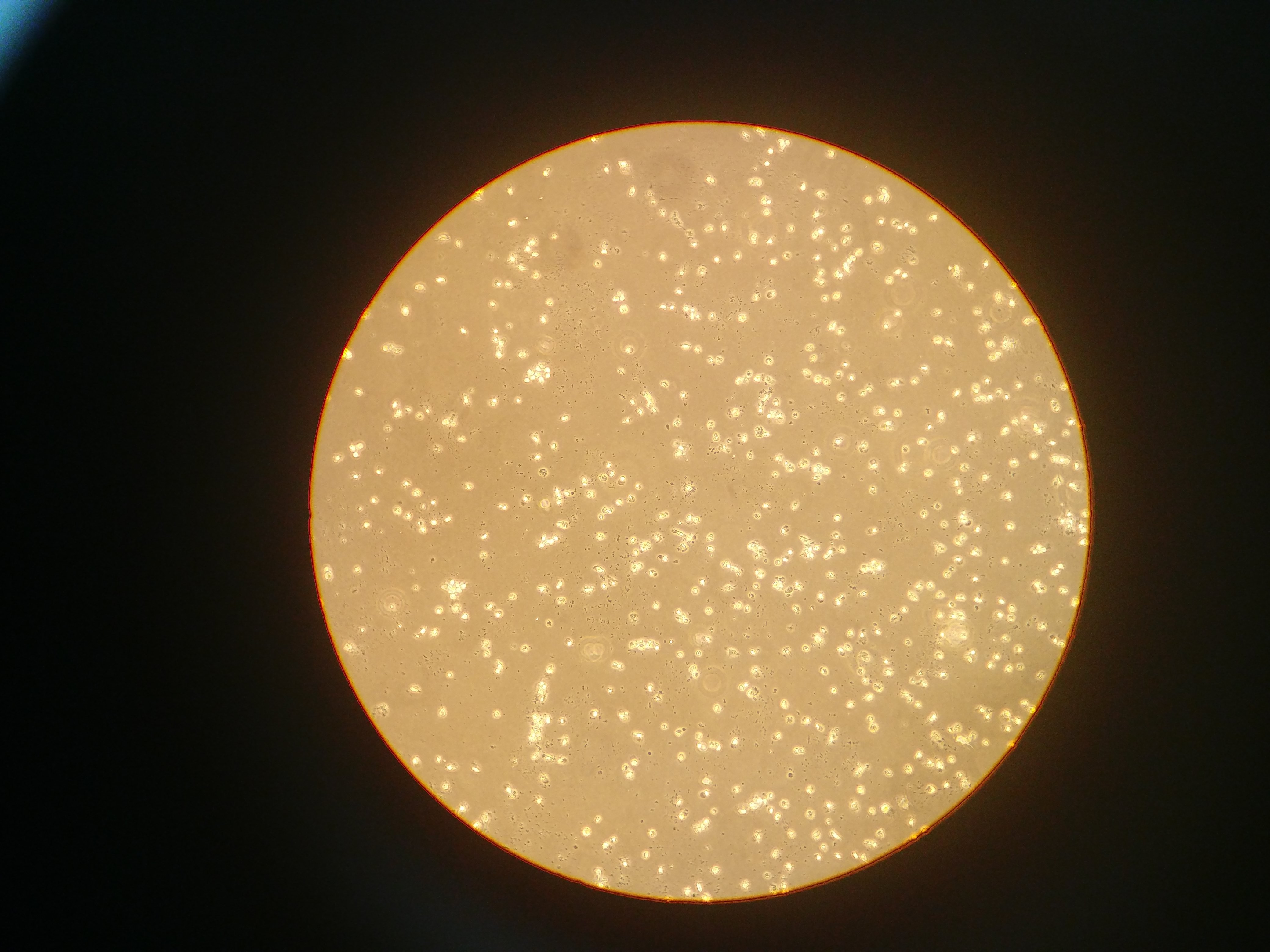

Supplement: Supplementary file 11 — Source data Fig. 3 [file 44321_2024_97_MOESM11_ESM.zip › Fig 3/Fig_3C/A673_shDIPRO1/A673-sh1/A673_sh1-1.jpg]

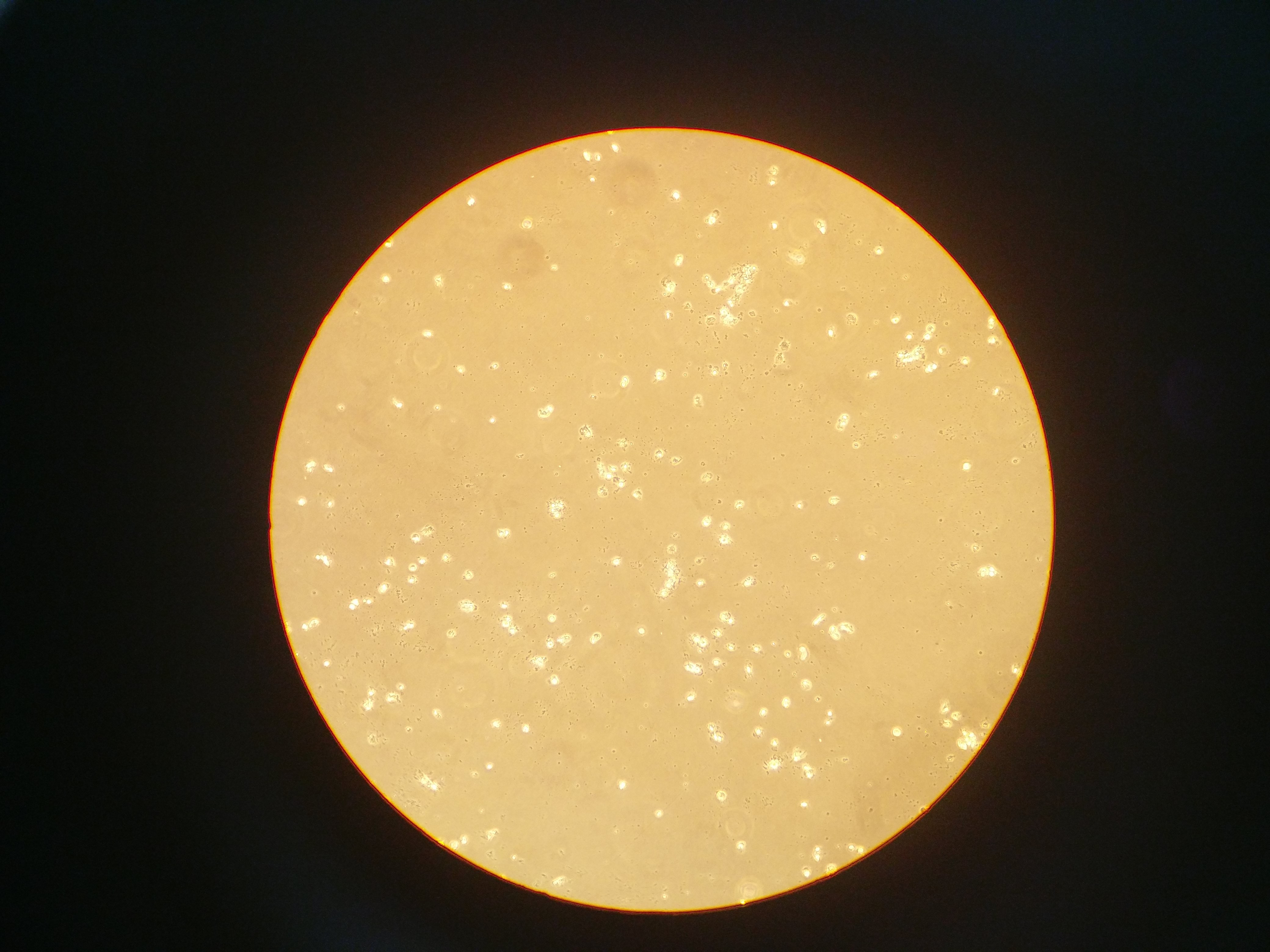

Supplement: Supplementary file 11 — Source data Fig. 3 [file 44321_2024_97_MOESM11_ESM.zip › Fig 3/Fig_3C/A673_shDIPRO1/A673-sh1/A673_sh1-2.jpg]

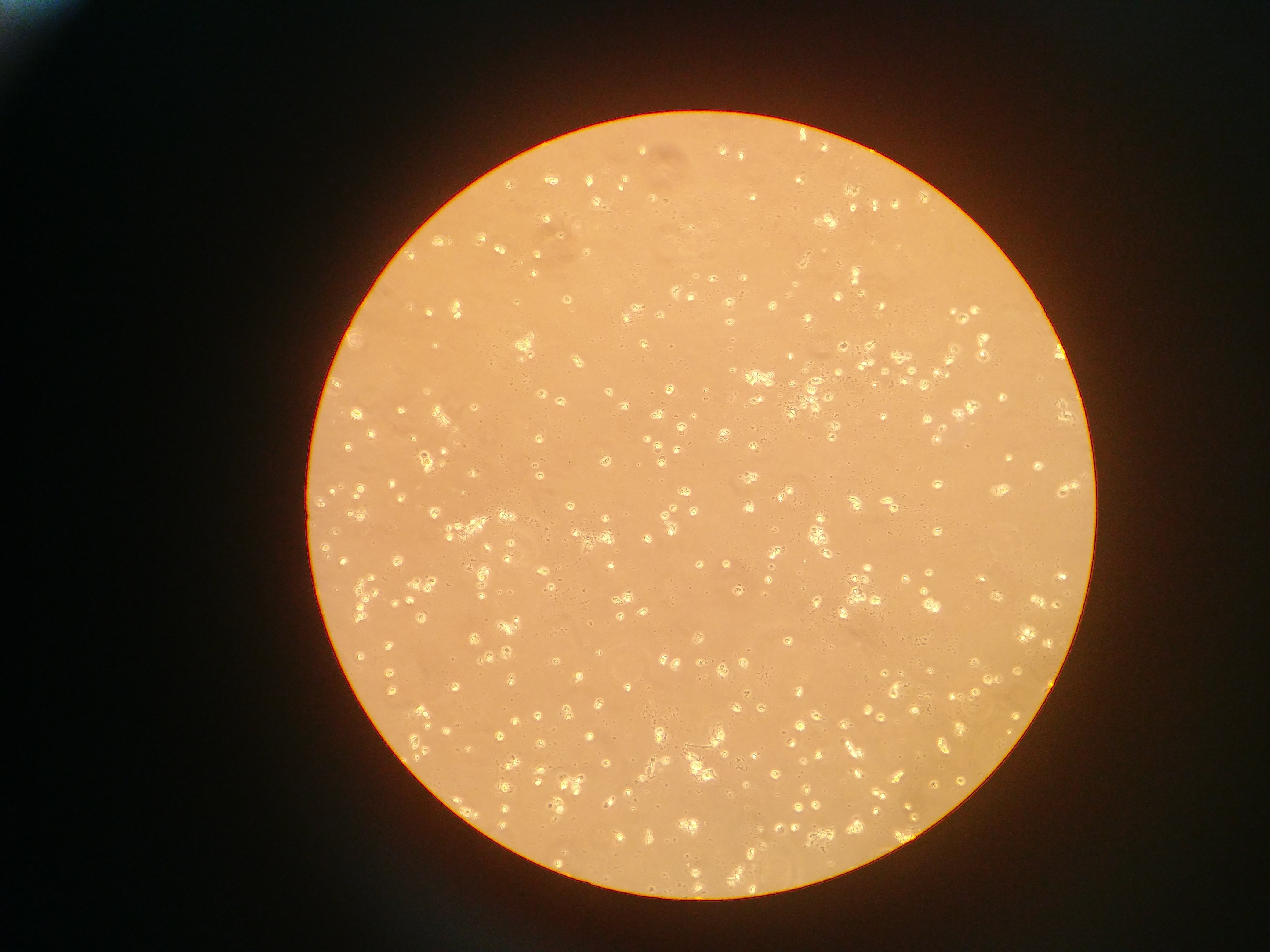

Supplement: Supplementary file 11 — Source data Fig. 3 [file 44321_2024_97_MOESM11_ESM.zip › Fig 3/Fig_3C/A673_shDIPRO1/A673-sh1/A673_sh1-4.jpg]

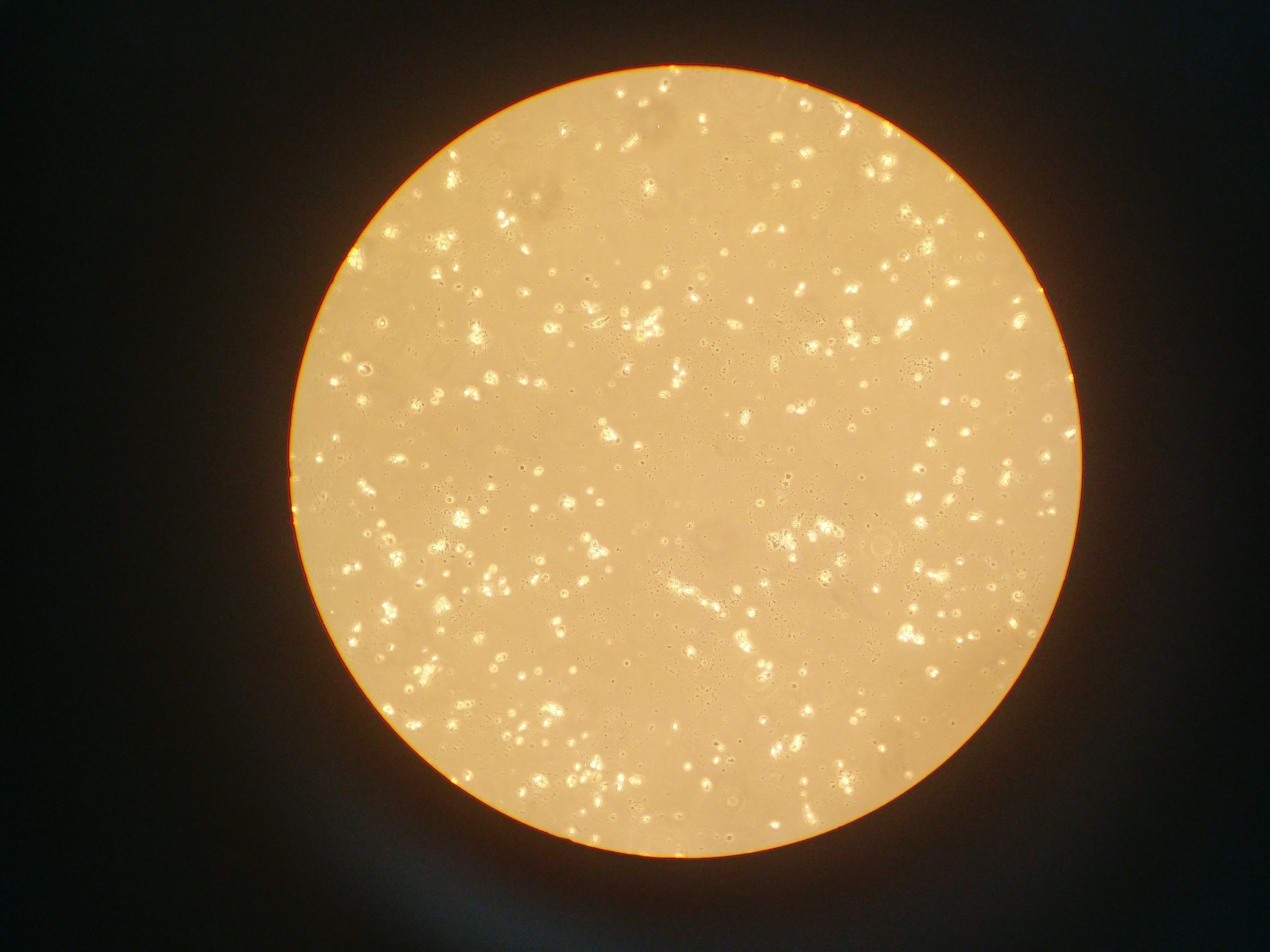

Supplement: Supplementary file 11 — Source data Fig. 3 [file 44321_2024_97_MOESM11_ESM.zip › Fig 3/Fig_3C/A673_shDIPRO1/A673-sh1/A673_sh1_3.jpg]

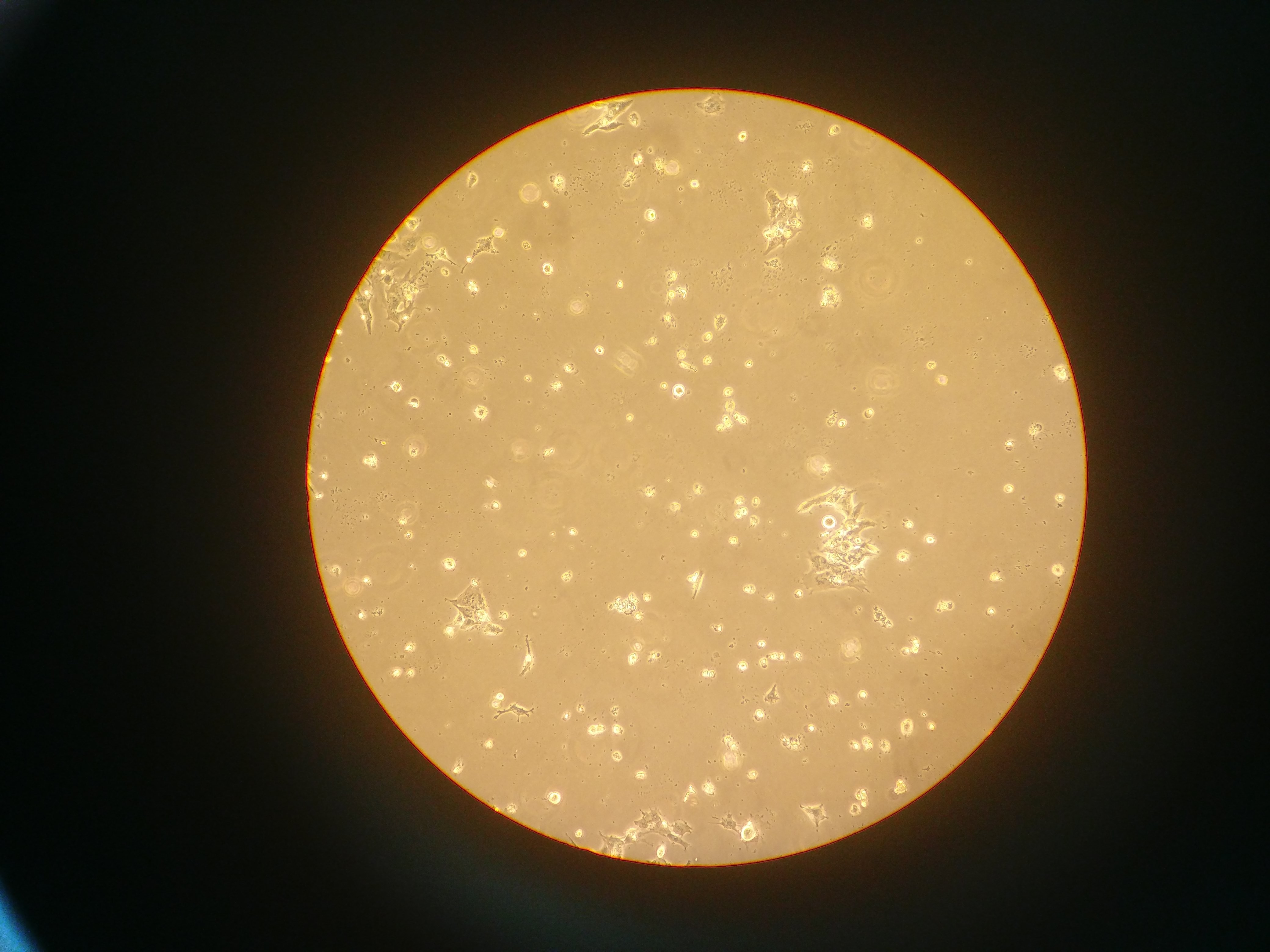

Supplement: Supplementary file 11 — Source data Fig. 3 [file 44321_2024_97_MOESM11_ESM.zip › Fig 3/Fig_3C/A673_shDIPRO1/A673_sh2/A673_sh2-1.jpg]

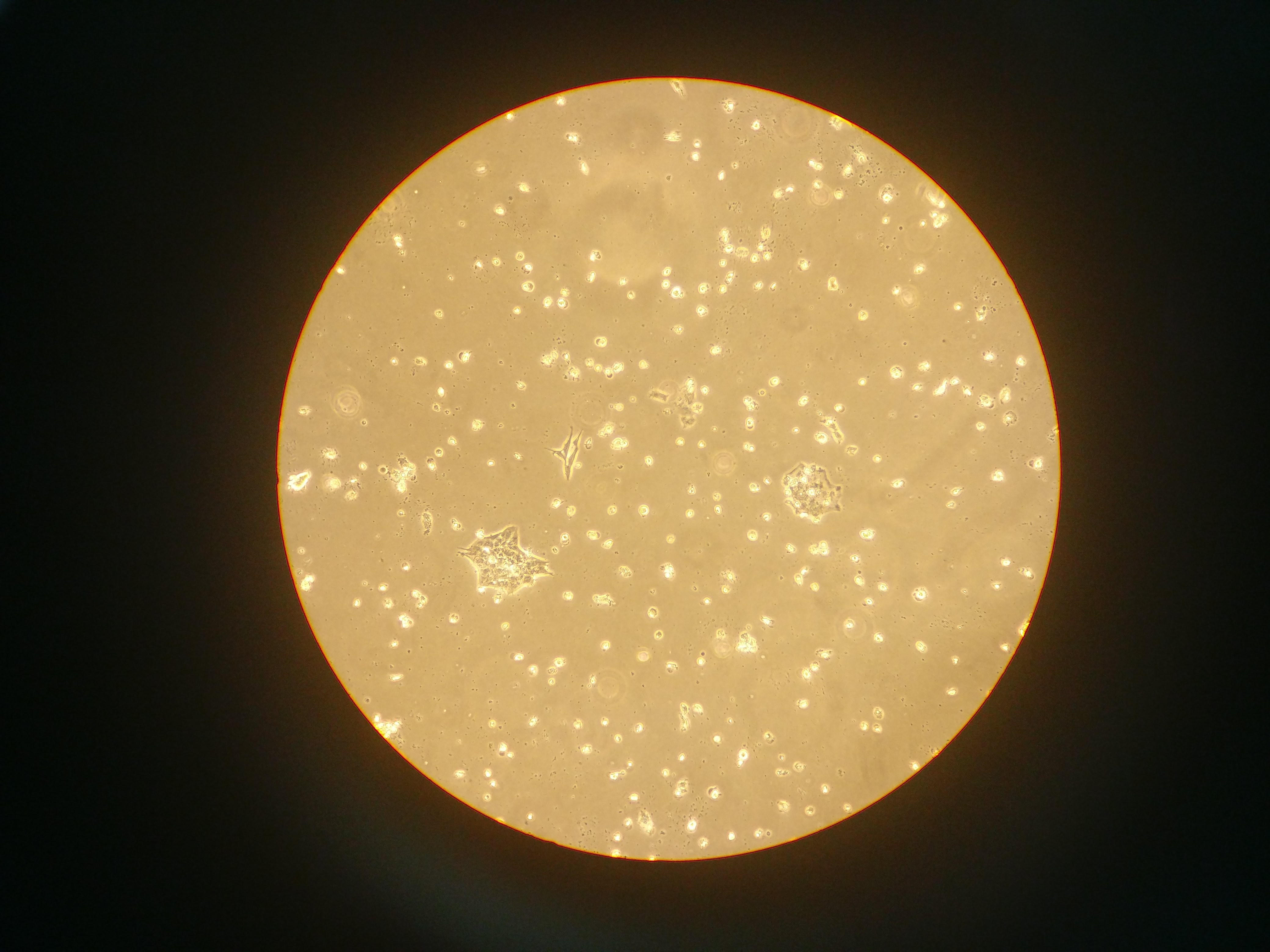

Supplement: Supplementary file 11 — Source data Fig. 3 [file 44321_2024_97_MOESM11_ESM.zip › Fig 3/Fig_3C/A673_shDIPRO1/A673_sh2/A673_sh2-2.jpg]

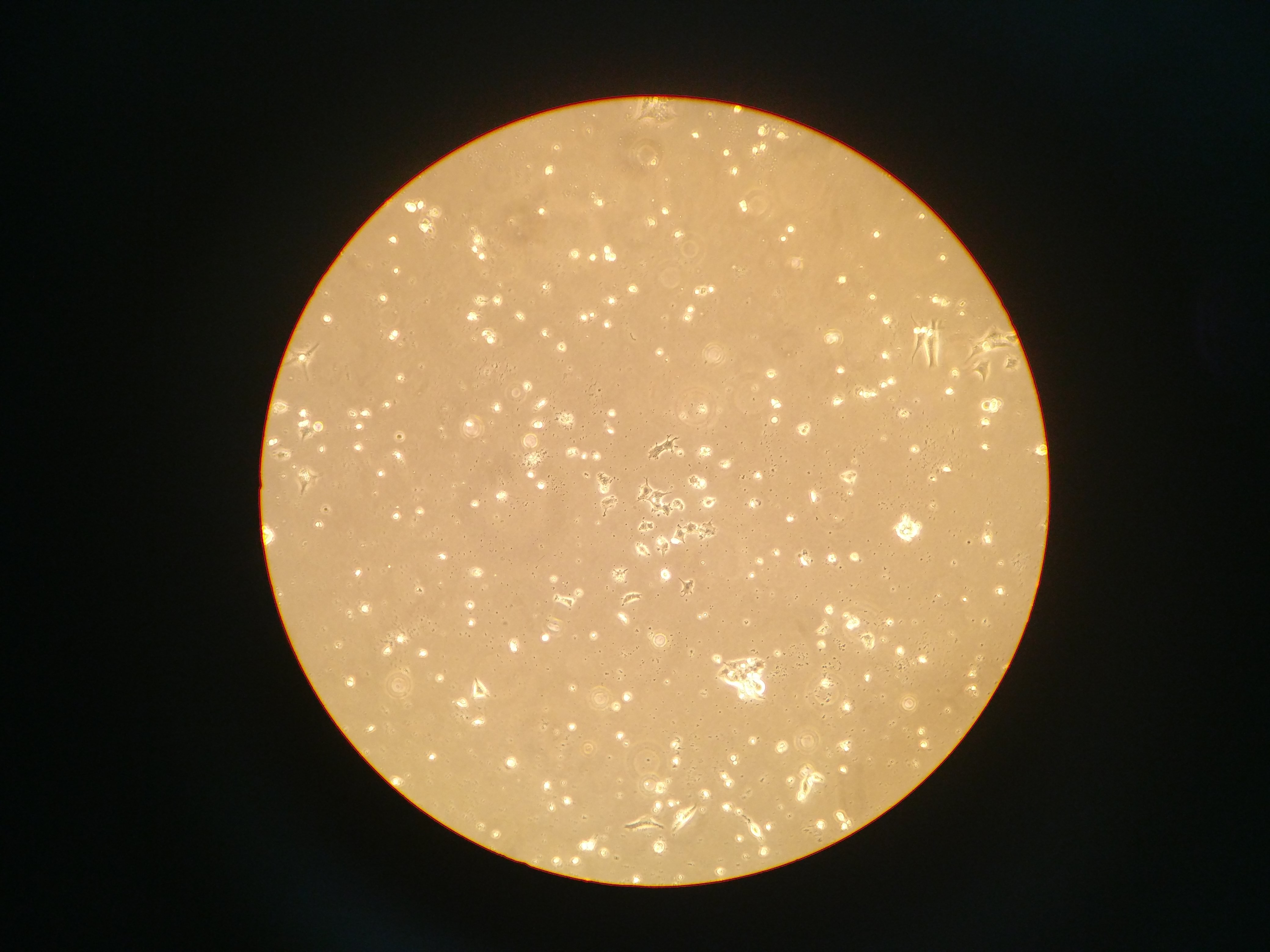

Supplement: Supplementary file 11 — Source data Fig. 3 [file 44321_2024_97_MOESM11_ESM.zip › Fig 3/Fig_3C/A673_shDIPRO1/A673_sh2/A673_sh2-3.jpg]

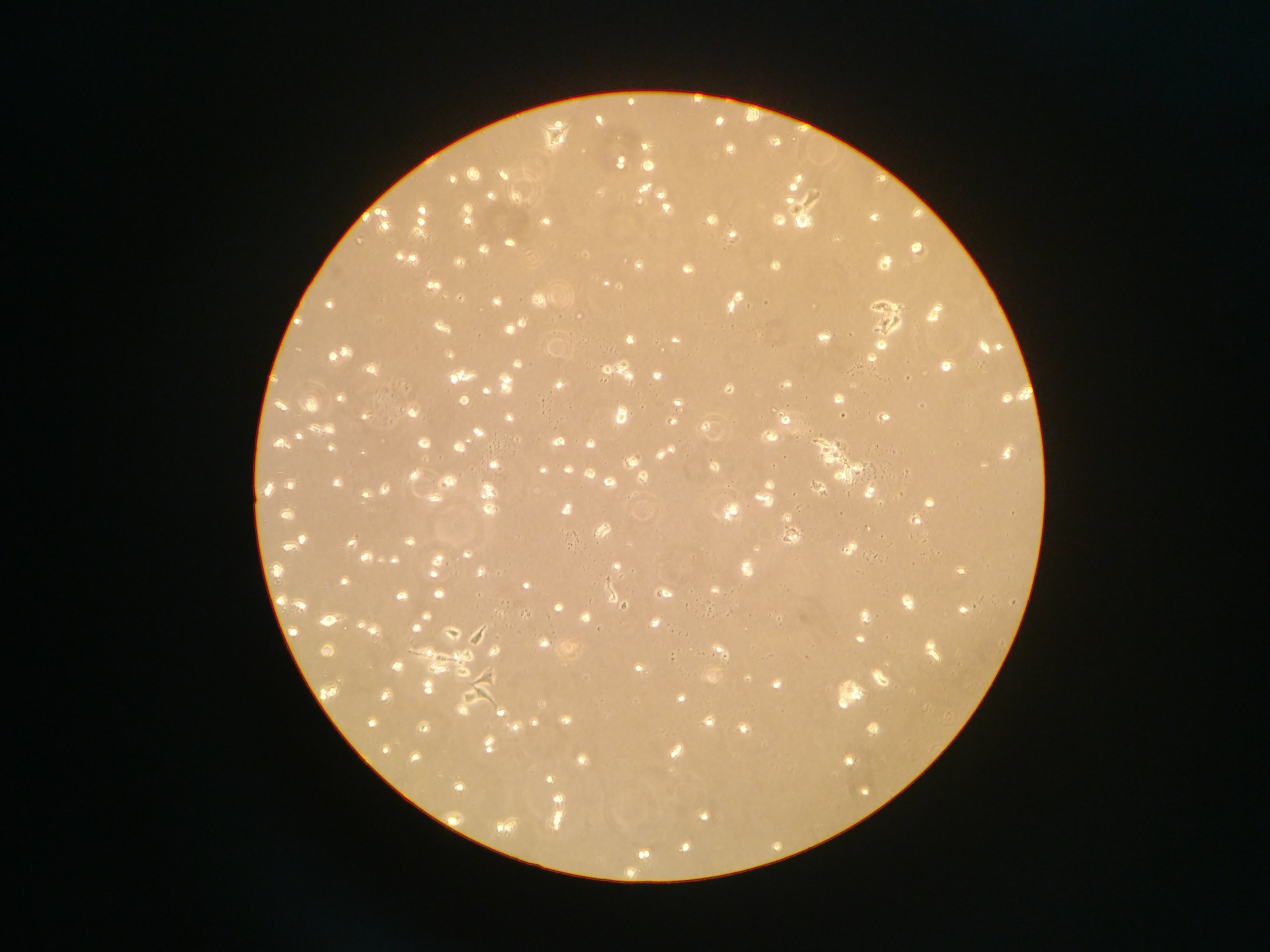

Supplement: Supplementary file 11 — Source data Fig. 3 [file 44321_2024_97_MOESM11_ESM.zip › Fig 3/Fig_3C/A673_shDIPRO1/A673_sh2/A673_sh2-4.jpg]

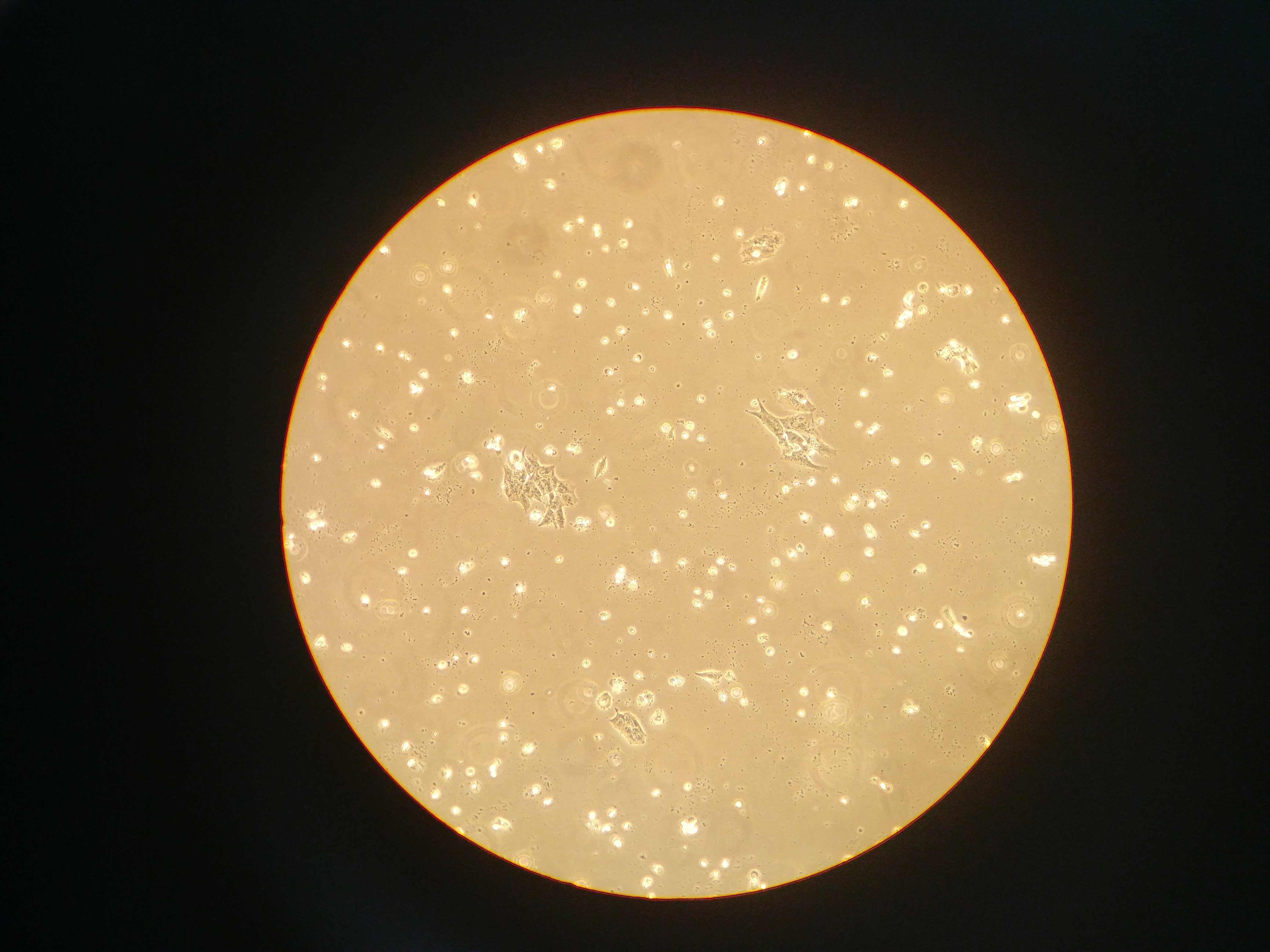

Supplement: Supplementary file 11 — Source data Fig. 3 [file 44321_2024_97_MOESM11_ESM.zip › Fig 3/Fig_3C/A673_shDIPRO1/A673_sh2/A673_sh2-5.jpg]

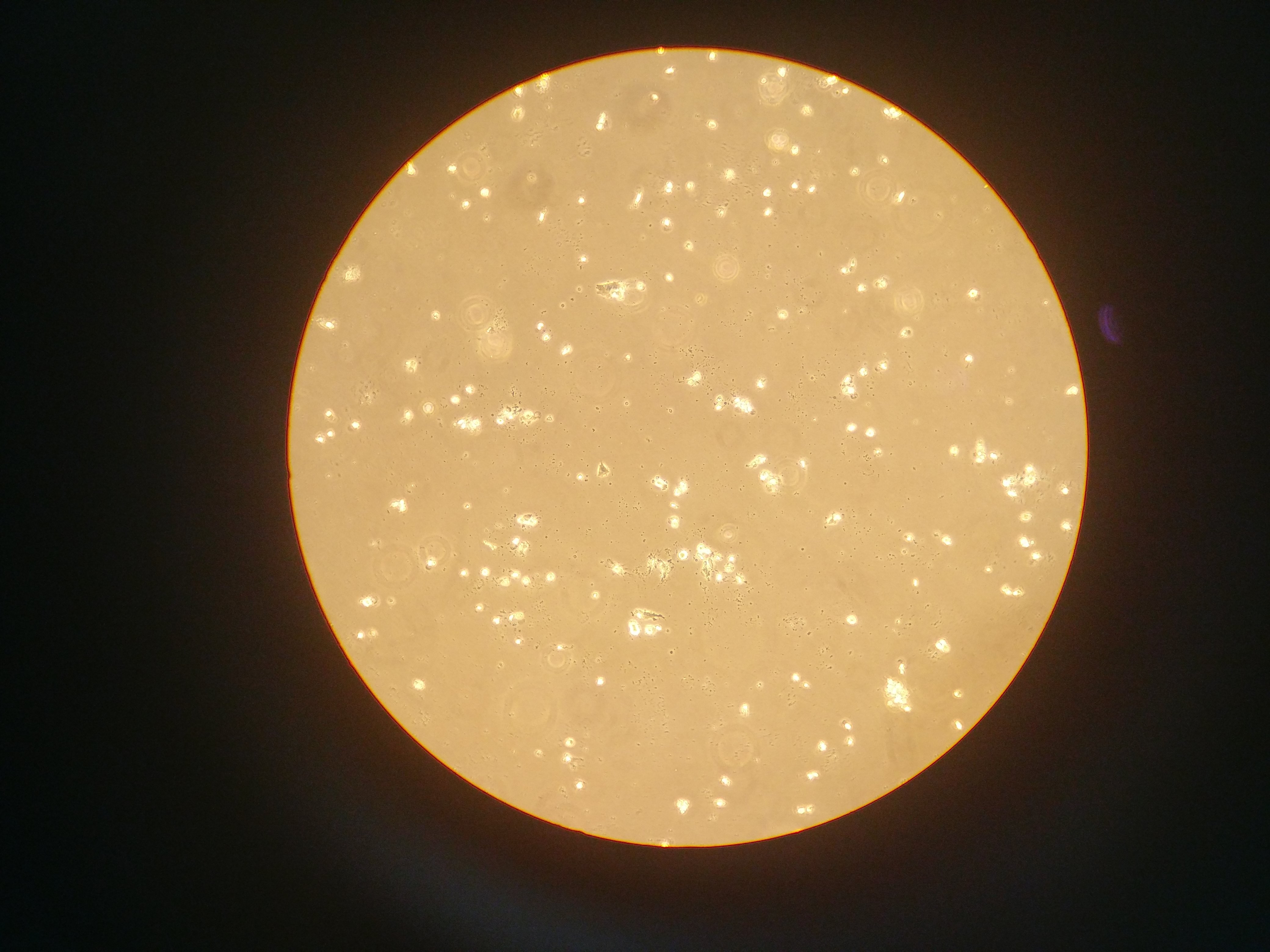

Supplement: Supplementary file 11 — Source data Fig. 3 [file 44321_2024_97_MOESM11_ESM.zip › Fig 3/Fig_3C/A673_shDIPRO1/A673_sh2/A673_sh2-6.jpg]

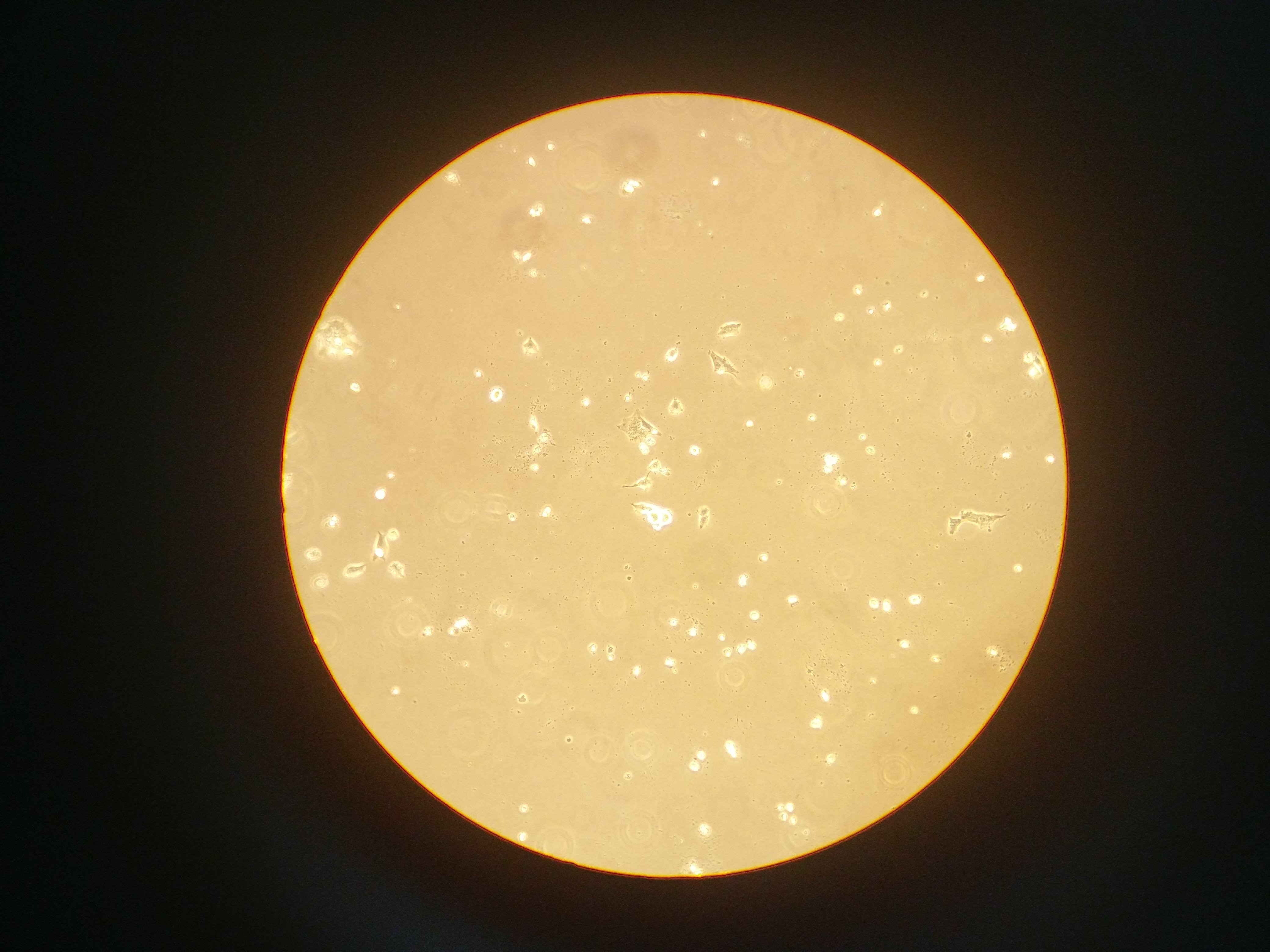

Supplement: Supplementary file 11 — Source data Fig. 3 [file 44321_2024_97_MOESM11_ESM.zip › Fig 3/Fig_3C/A673_shDIPRO1/A673_sh2/A673_sh2-7.jpg]

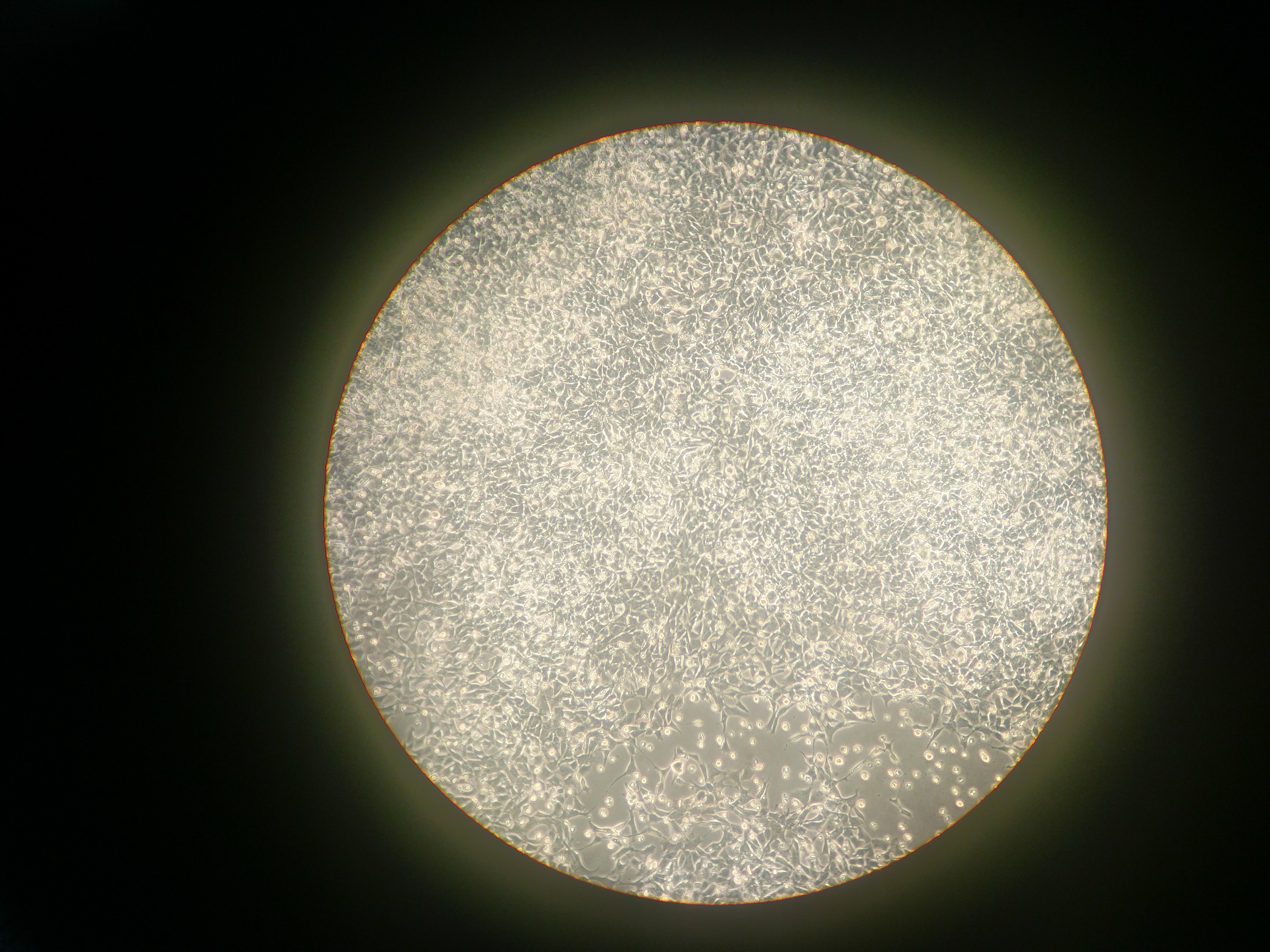

Supplement: Supplementary file 11 — Source data Fig. 3 [file 44321_2024_97_MOESM11_ESM.zip › Fig 3/Fig_3C/EW7-shCTL/EW7-shCTL-1.jpg]

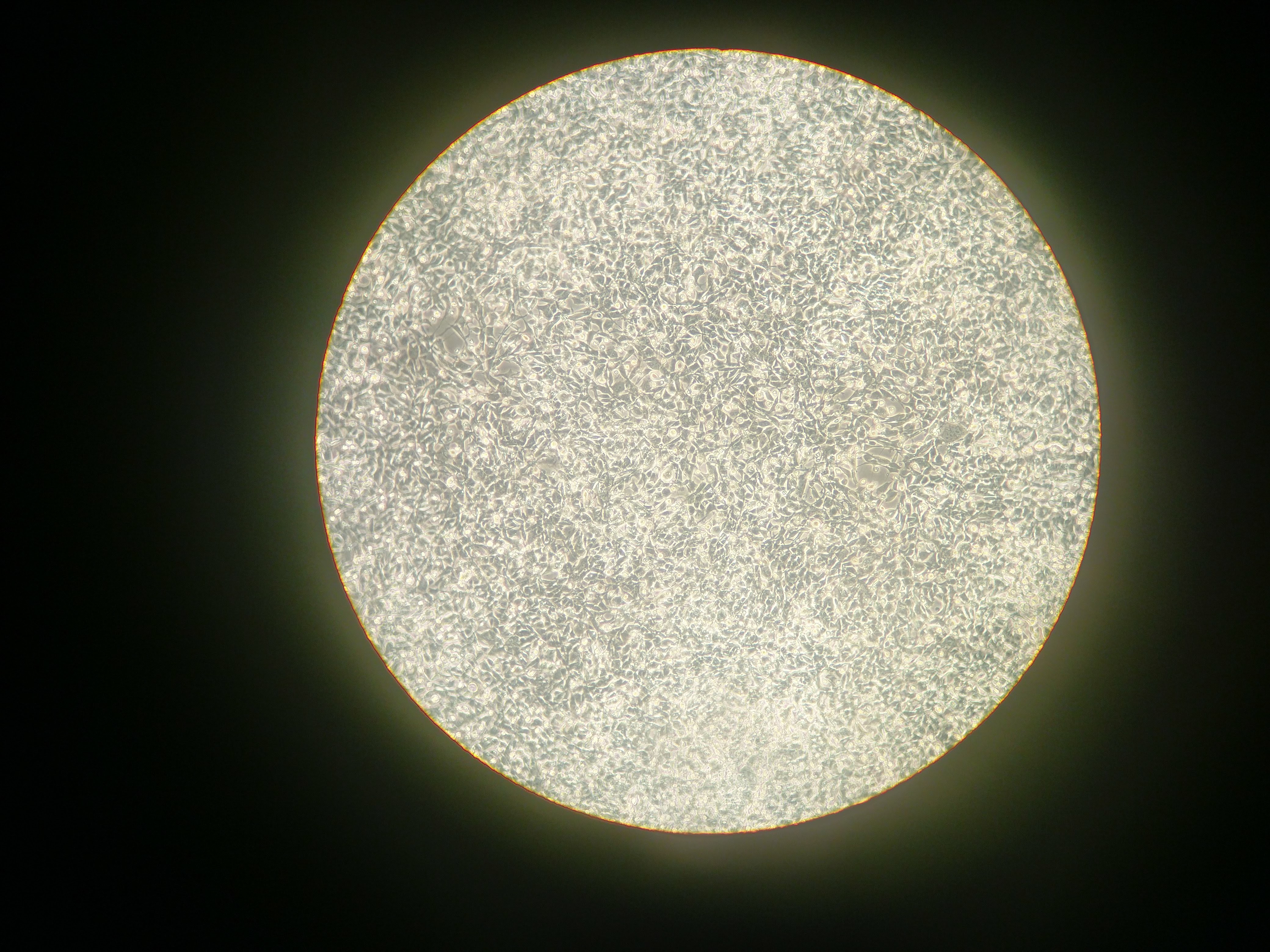

Supplement: Supplementary file 11 — Source data Fig. 3 [file 44321_2024_97_MOESM11_ESM.zip › Fig 3/Fig_3C/EW7-shCTL/EW7-shCTL-2.jpg]

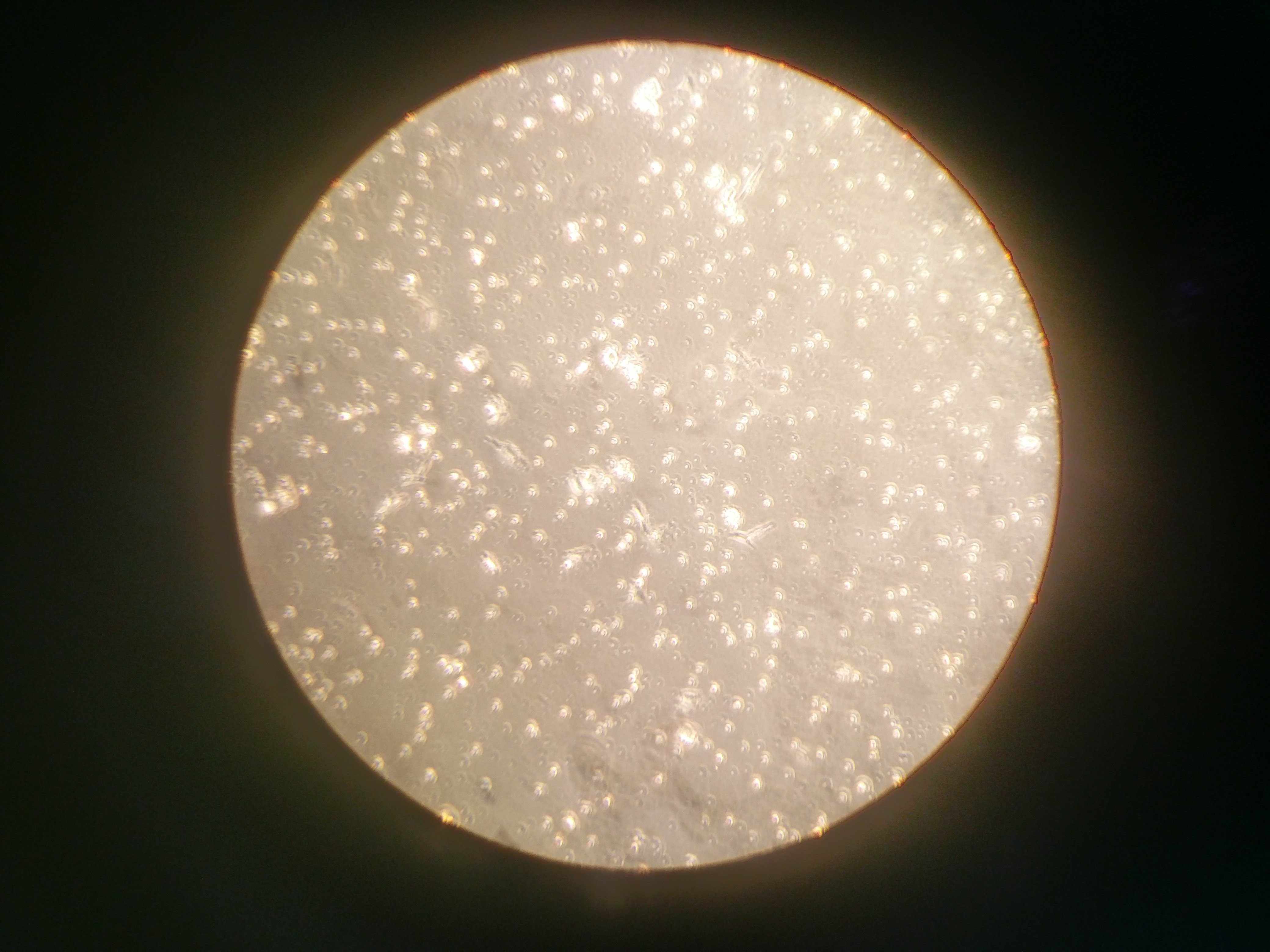

Supplement: Supplementary file 11 — Source data Fig. 3 [file 44321_2024_97_MOESM11_ESM.zip › Fig 3/Fig_3C/EW7_shDIPRO1/EW7-shDIPRO1-1.jpg]

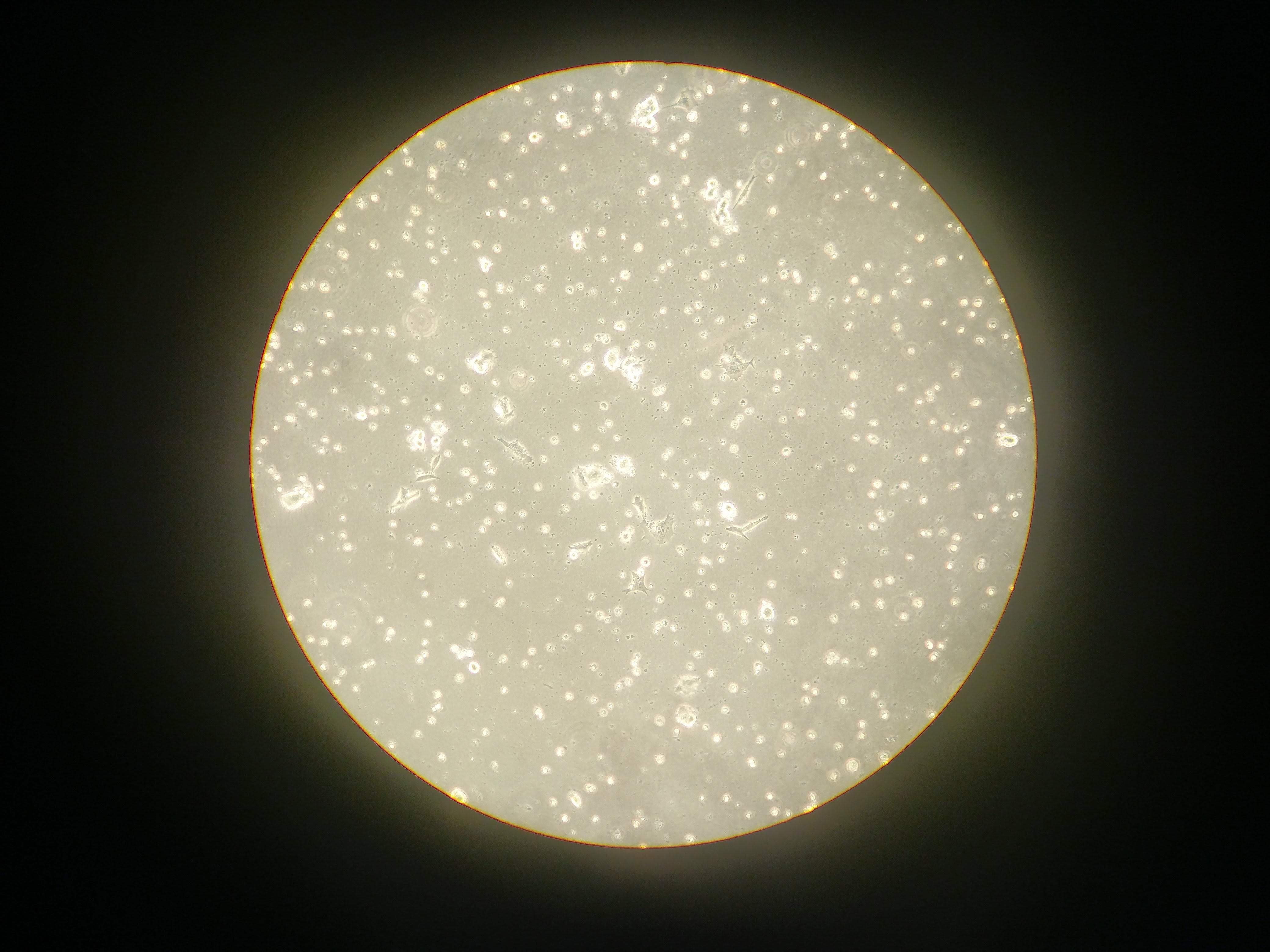

Supplement: Supplementary file 11 — Source data Fig. 3 [file 44321_2024_97_MOESM11_ESM.zip › Fig 3/Fig_3C/EW7_shDIPRO1/EW7-shDIPRO1-2.jpg]

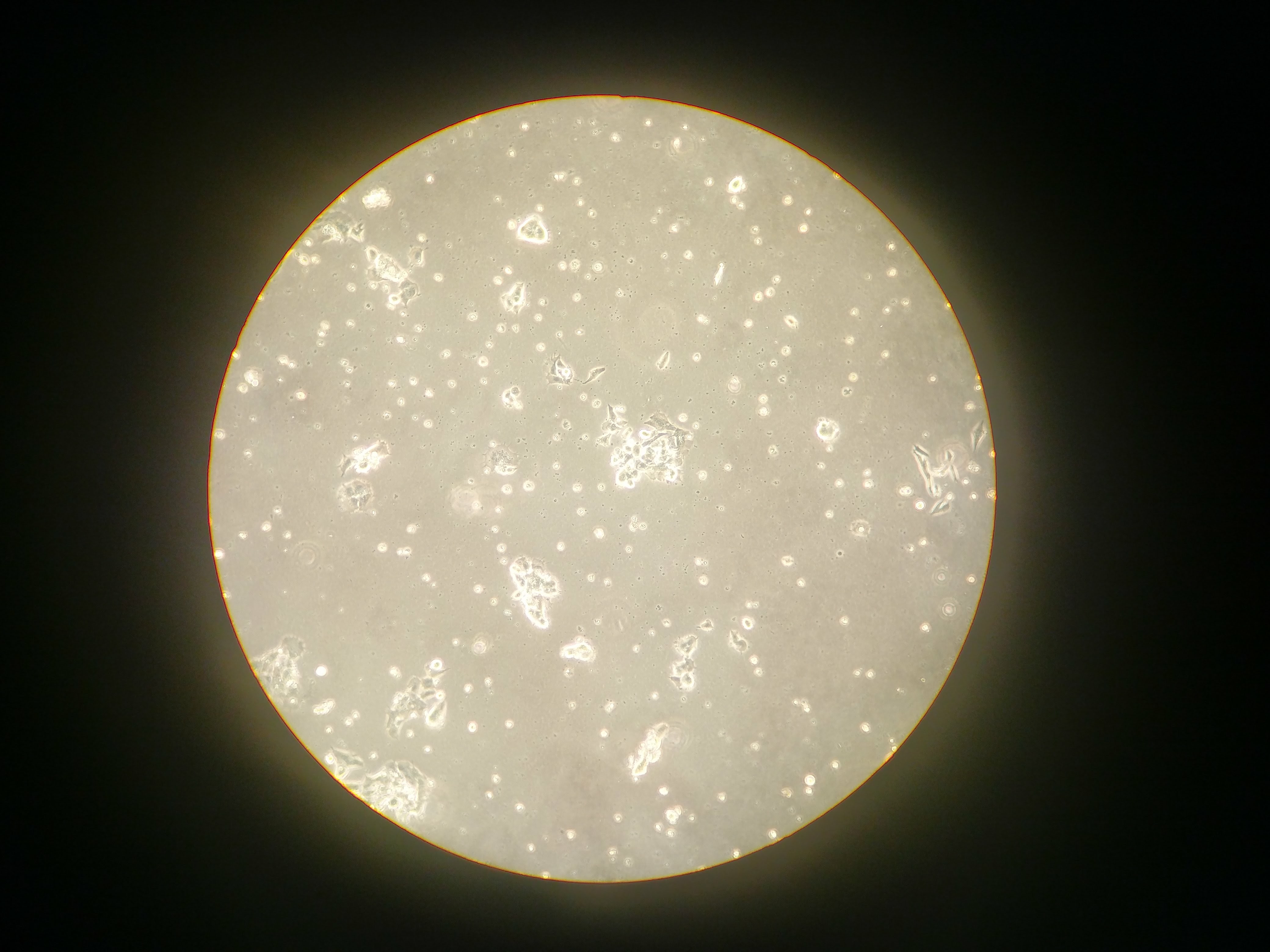

Supplement: Supplementary file 11 — Source data Fig. 3 [file 44321_2024_97_MOESM11_ESM.zip › Fig 3/Fig_3C/EW7_shDIPRO1/EW7-shDIPRO1-3.jpg]

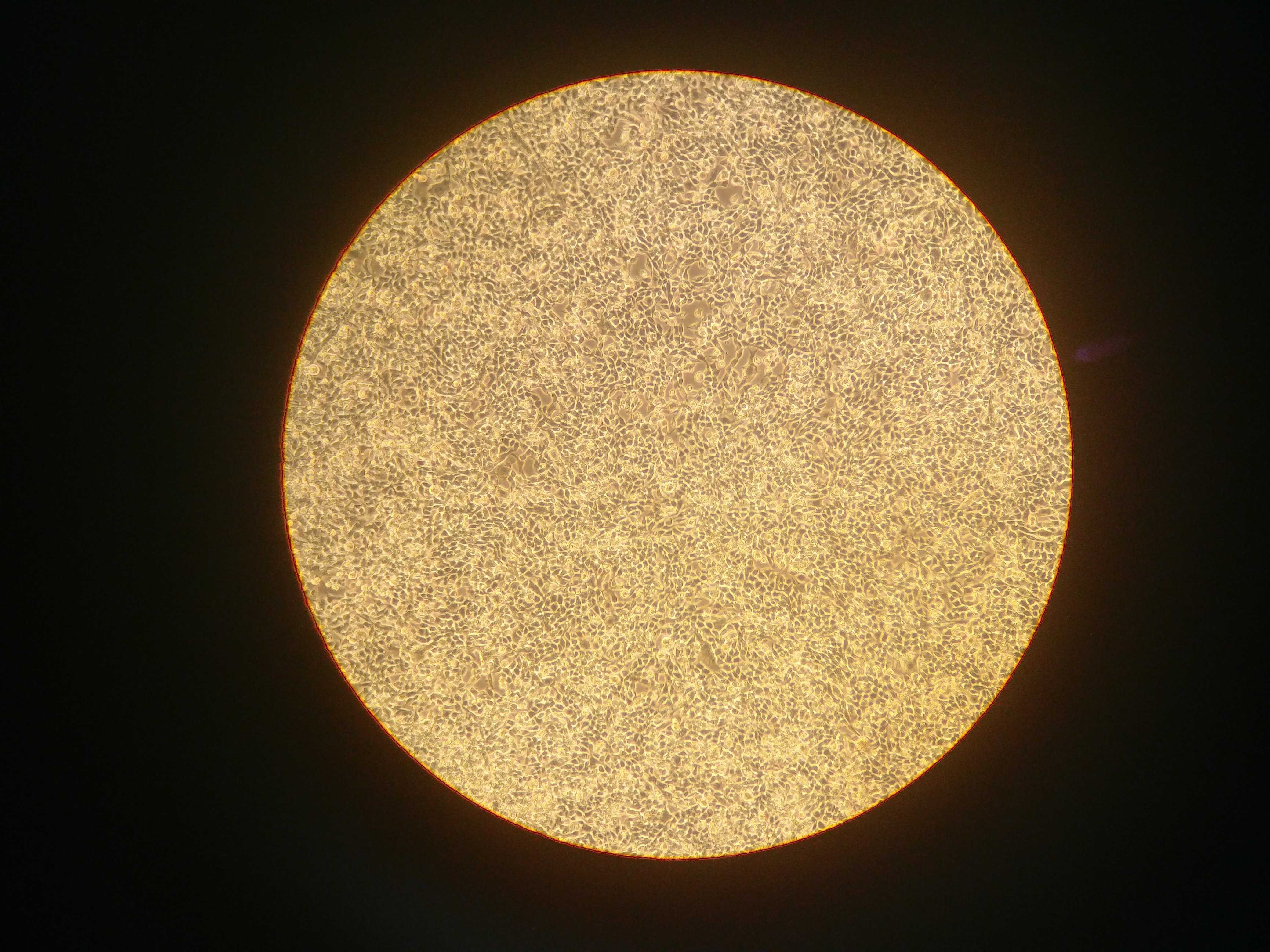

Supplement: Supplementary file 11 — Source data Fig. 3 [file 44321_2024_97_MOESM11_ESM.zip › Fig 3/Fig_3C/JR-shCTL/JR_pCTL.jpg]

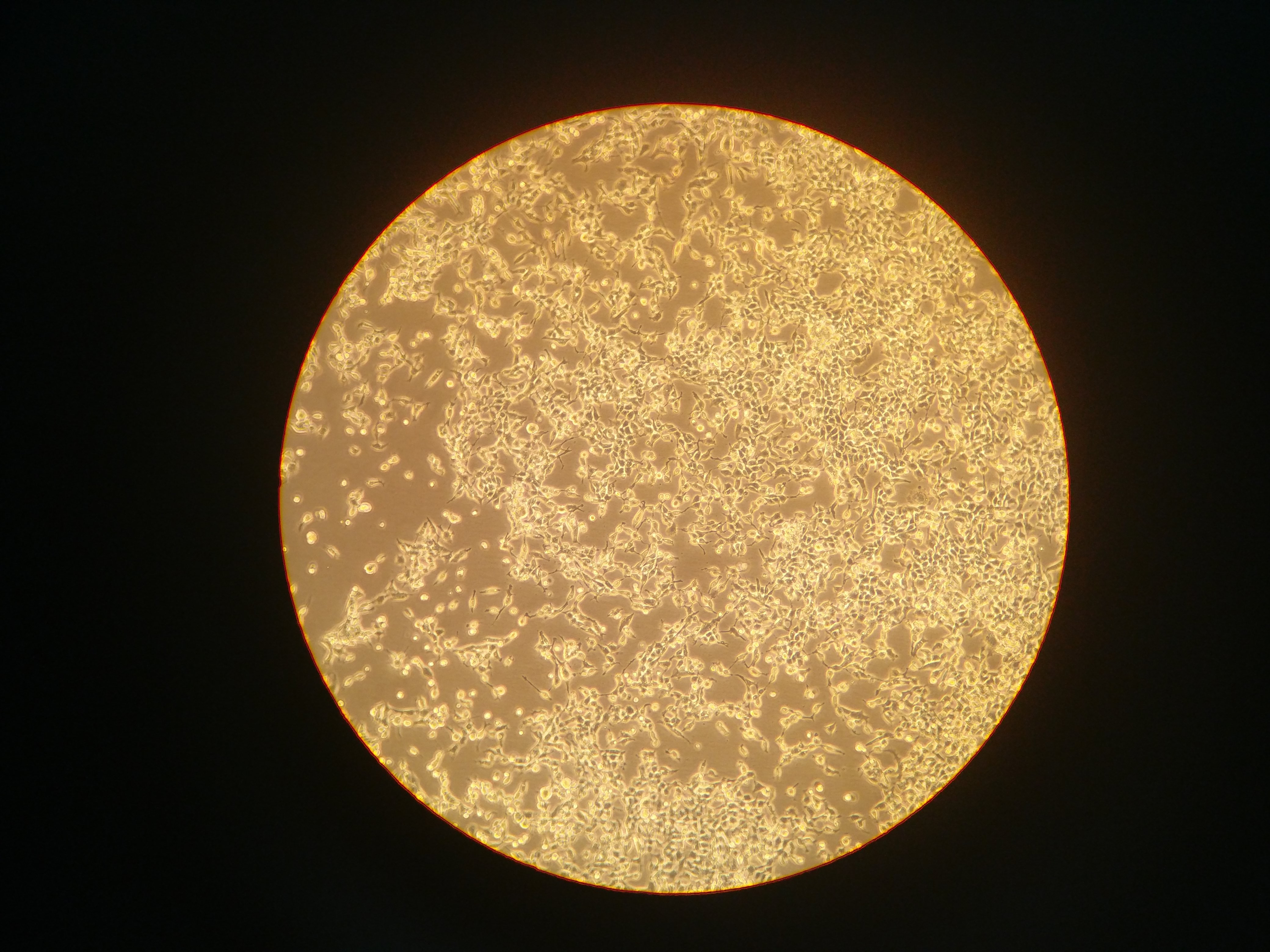

Supplement: Supplementary file 11 — Source data Fig. 3 [file 44321_2024_97_MOESM11_ESM.zip › Fig 3/Fig_3C/JR-shCTL/JR_shCTL-1.jpg]

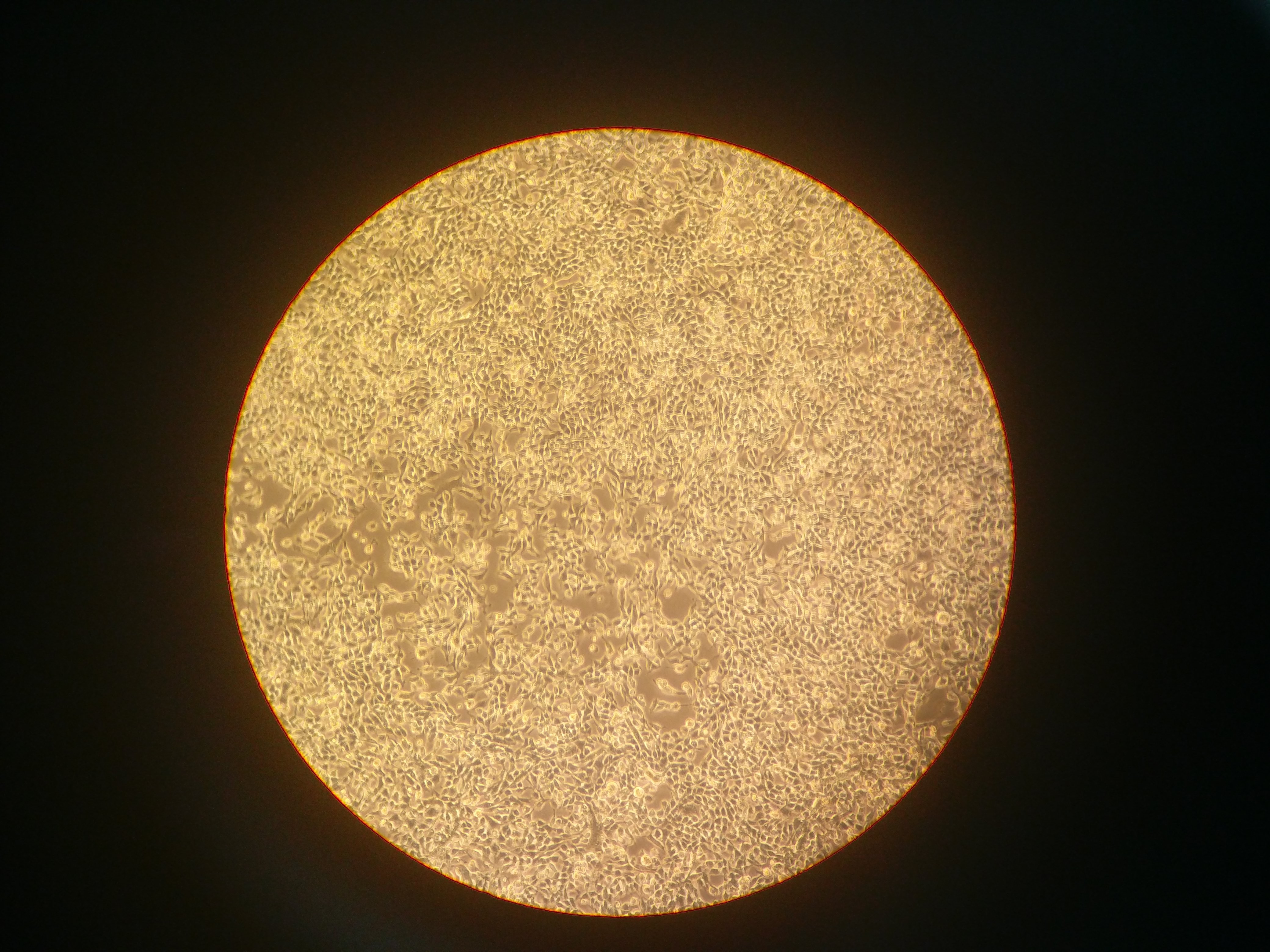

Supplement: Supplementary file 11 — Source data Fig. 3 [file 44321_2024_97_MOESM11_ESM.zip › Fig 3/Fig_3C/JR-shCTL/JR_shCTL-2.jpg]

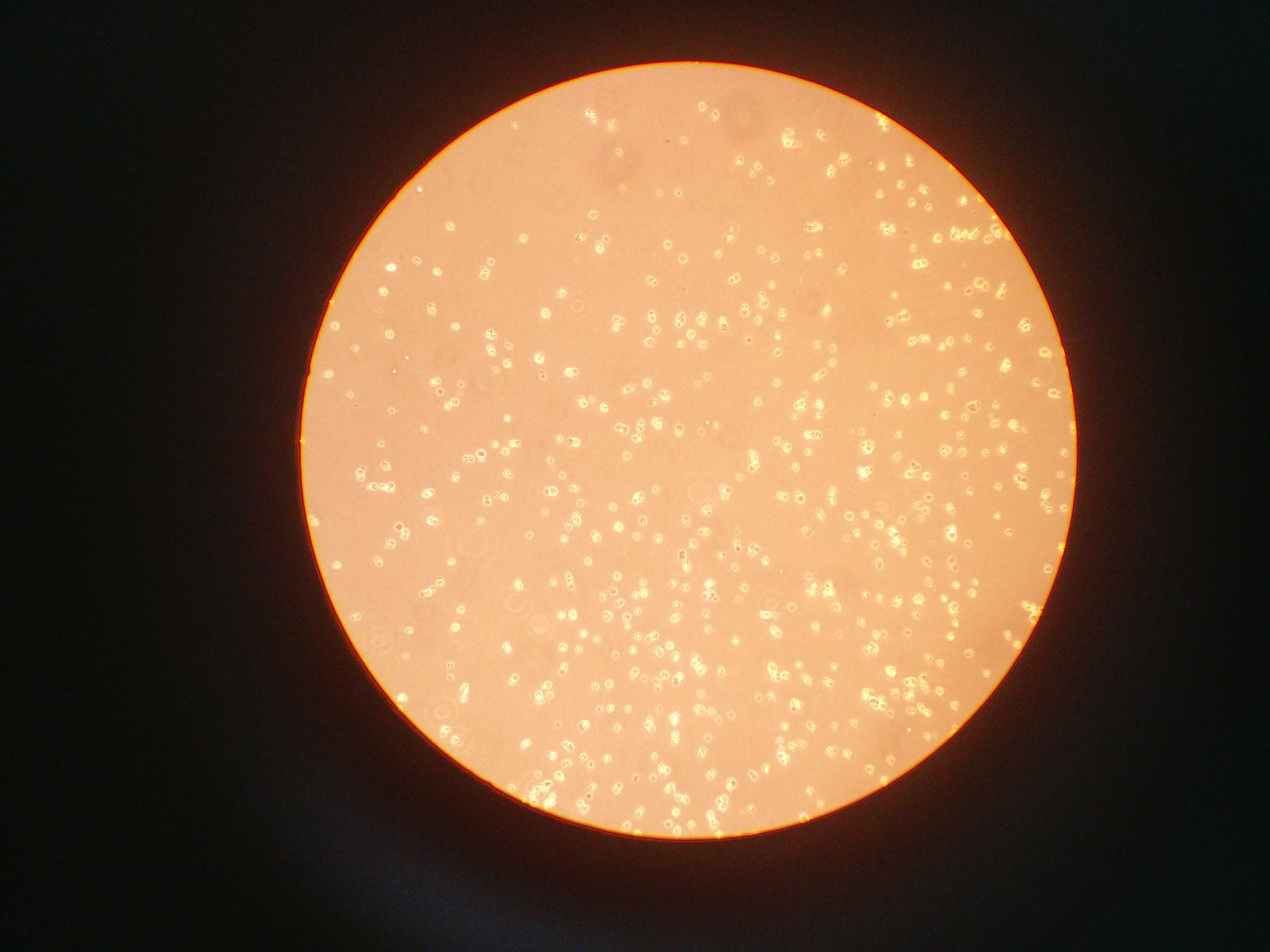

Supplement: Supplementary file 11 — Source data Fig. 3 [file 44321_2024_97_MOESM11_ESM.zip › Fig 3/Fig_3C/JR-shDIPRO1/JR-sh2/JR_sh2-1.jpg]

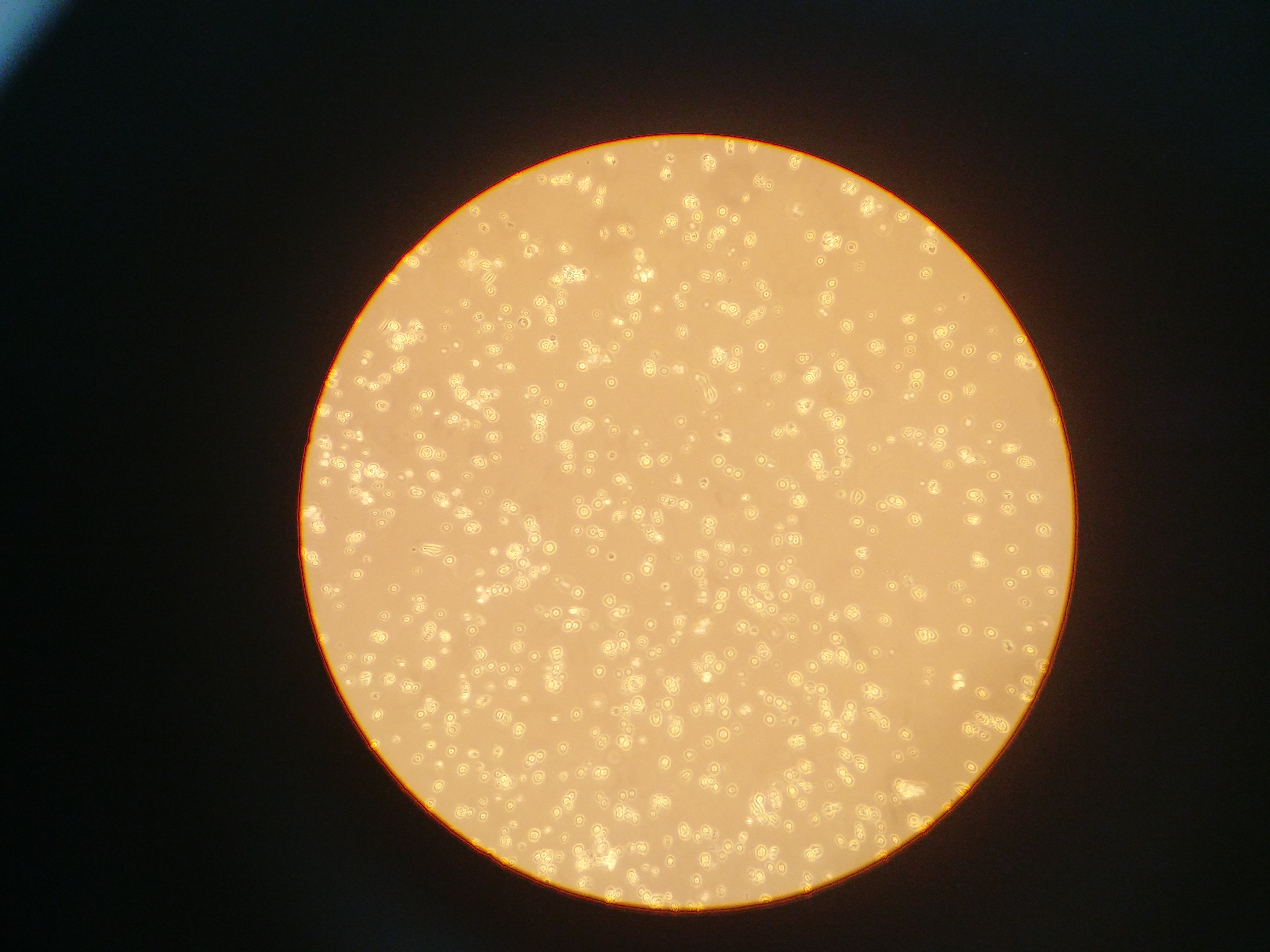

Supplement: Supplementary file 11 — Source data Fig. 3 [file 44321_2024_97_MOESM11_ESM.zip › Fig 3/Fig_3C/JR-shDIPRO1/JR-sh2/JR_sh2-2.jpg]

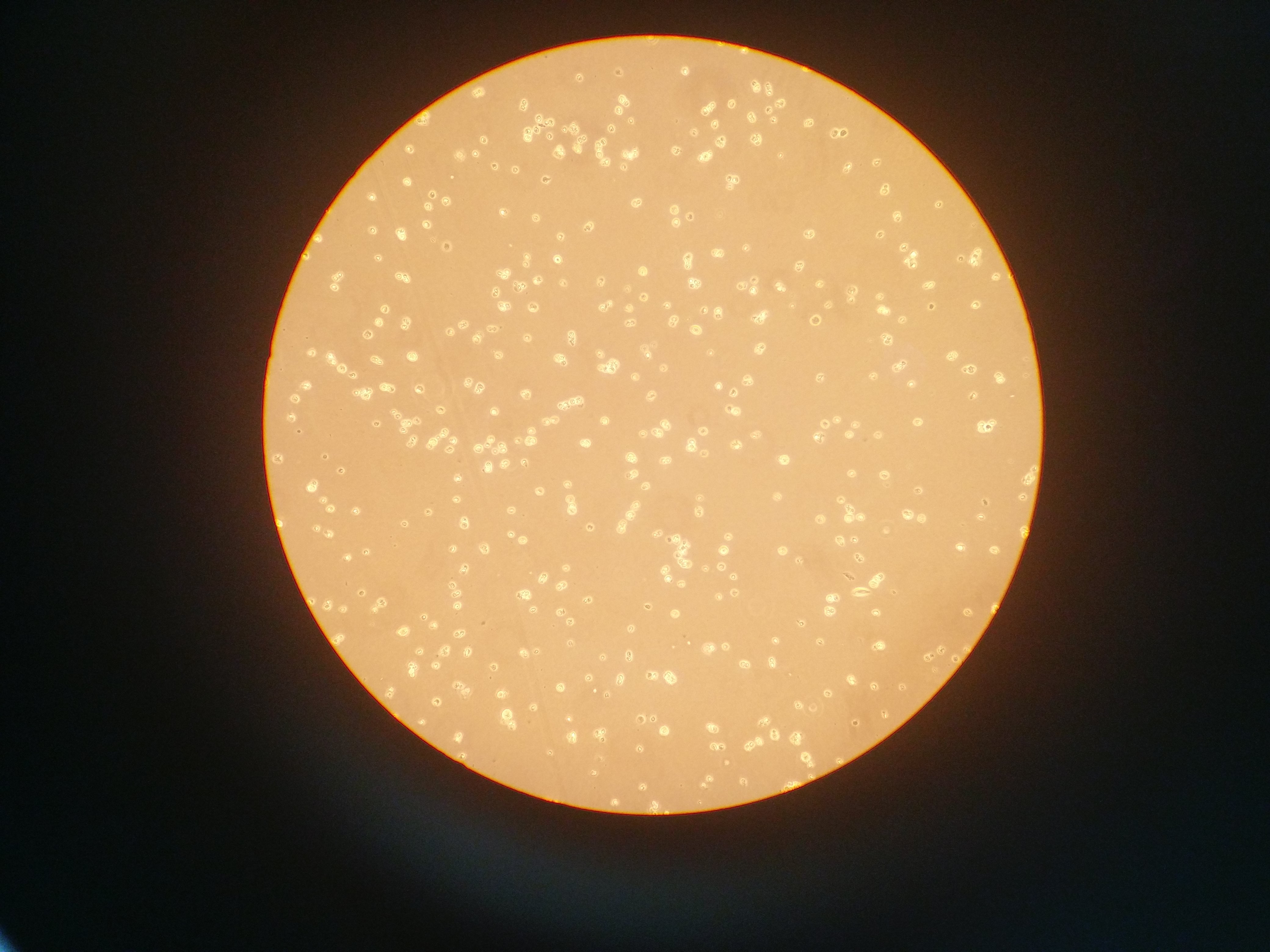

Supplement: Supplementary file 11 — Source data Fig. 3 [file 44321_2024_97_MOESM11_ESM.zip › Fig 3/Fig_3C/JR-shDIPRO1/JR-sh2/JR_sh2-3.jpg]

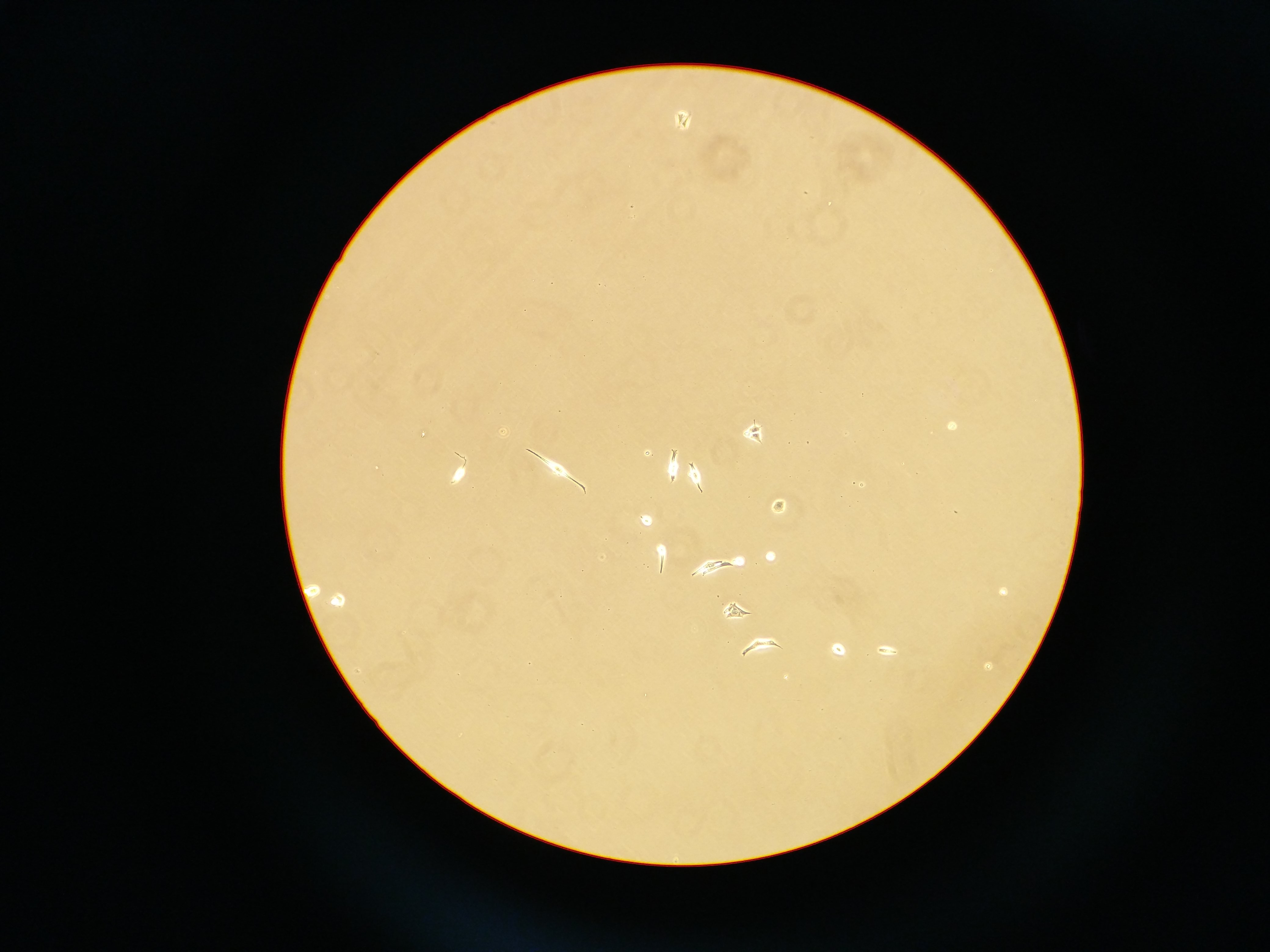

Supplement: Supplementary file 11 — Source data Fig. 3 [file 44321_2024_97_MOESM11_ESM.zip › Fig 3/Fig_3C/JR-shDIPRO1/JR_sh1/JR-sh1-1.jpg]

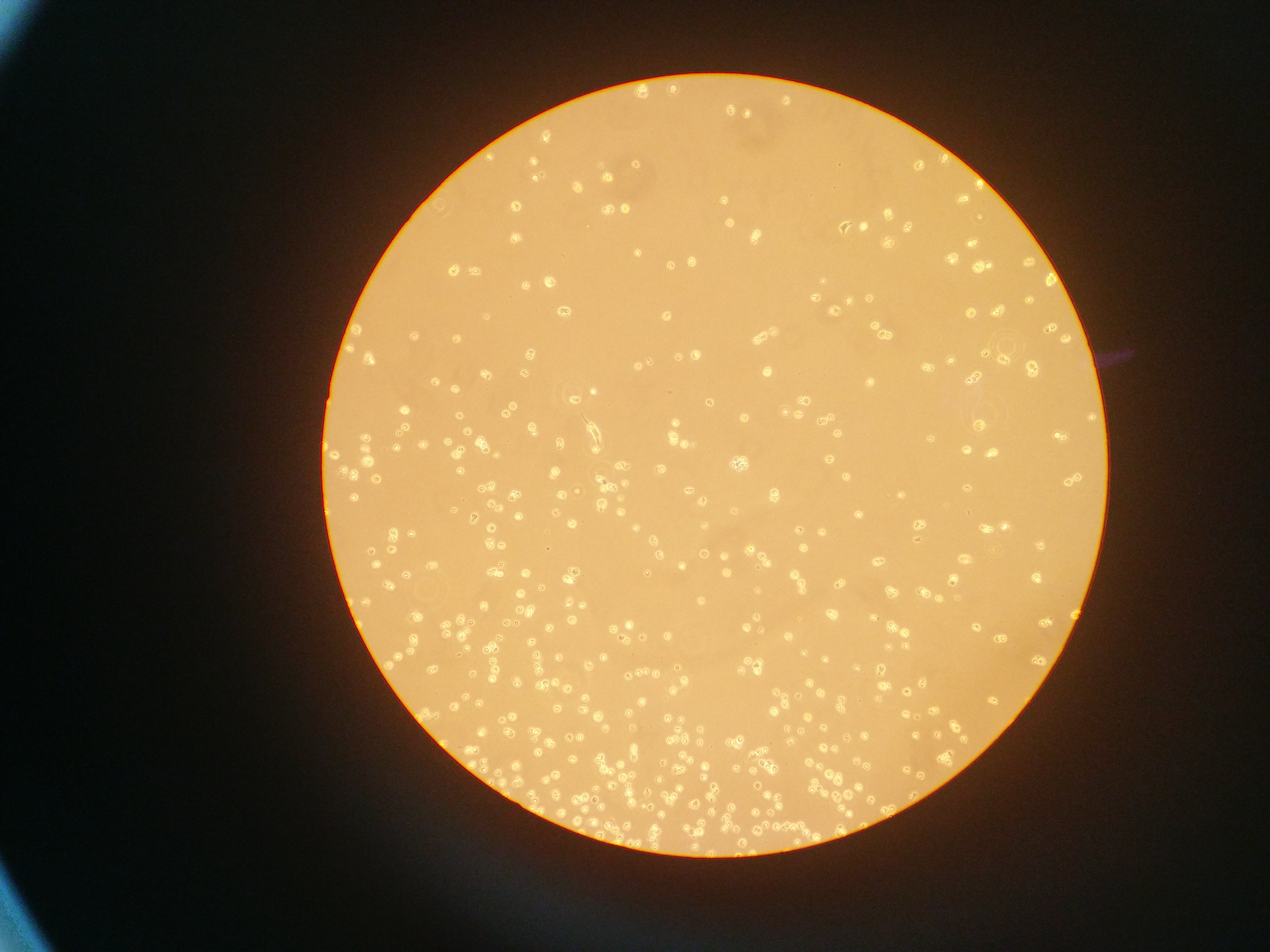

Supplement: Supplementary file 11 — Source data Fig. 3 [file 44321_2024_97_MOESM11_ESM.zip › Fig 3/Fig_3C/JR-shDIPRO1/JR_sh1/JR_sh1-2.jpg]

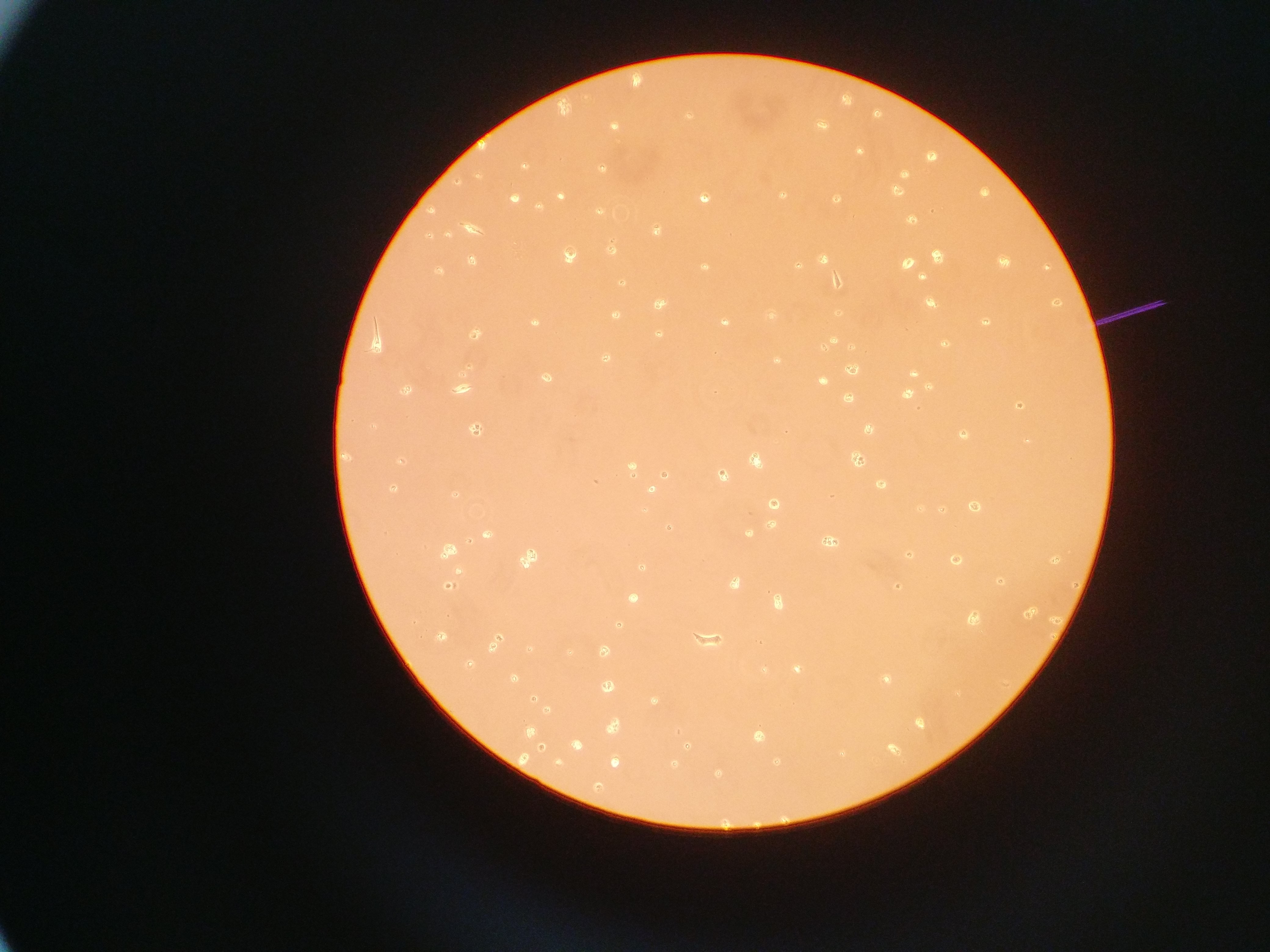

Supplement: Supplementary file 11 — Source data Fig. 3 [file 44321_2024_97_MOESM11_ESM.zip › Fig 3/Fig_3C/JR-shDIPRO1/JR_sh1/JR_sh1-3.jpg]

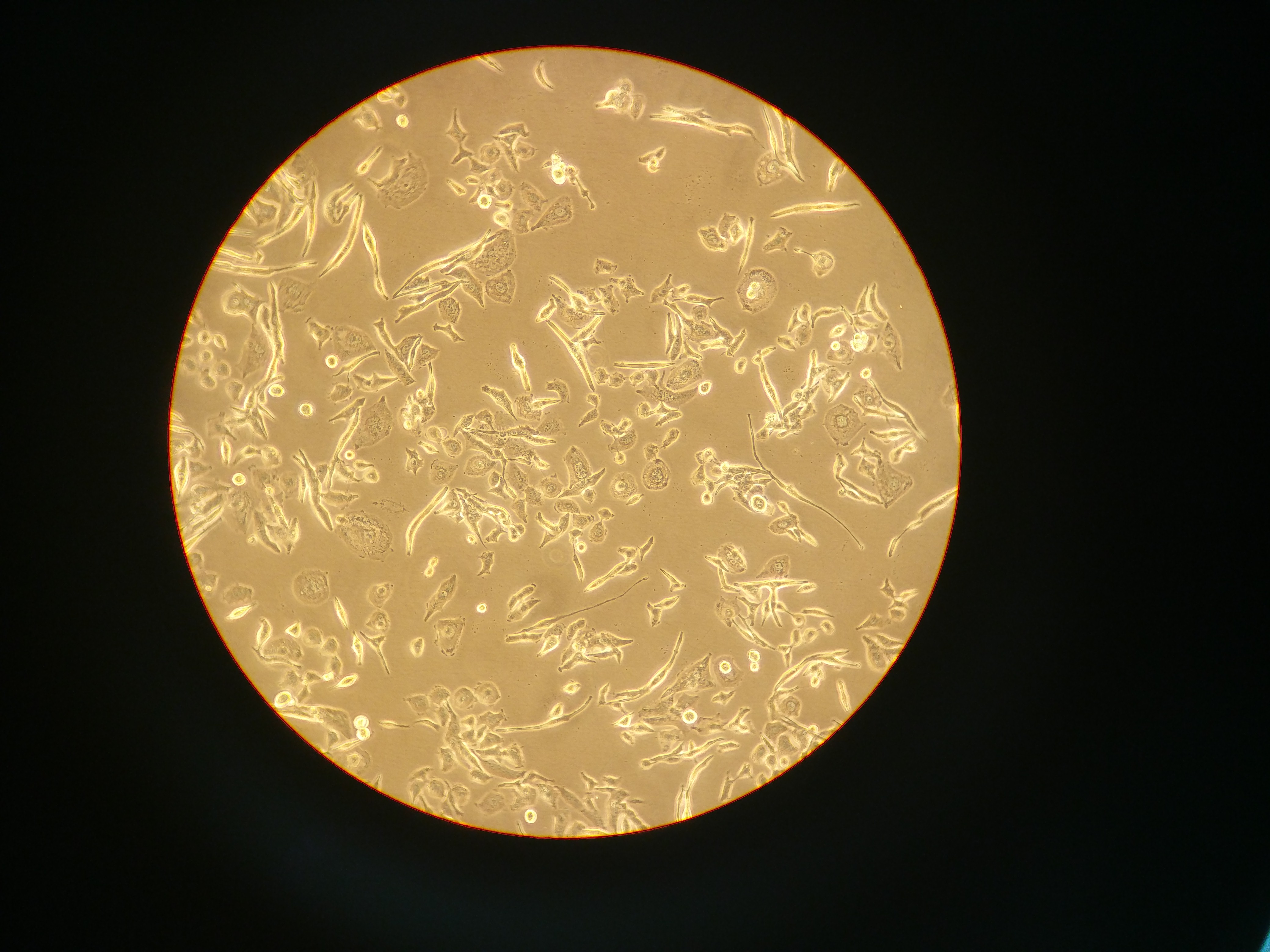

Supplement: Supplementary file 11 — Source data Fig. 3 [file 44321_2024_97_MOESM11_ESM.zip › Fig 3/Fig_3C/TE671_shCTL/TE671-shCTL-7.jpg]

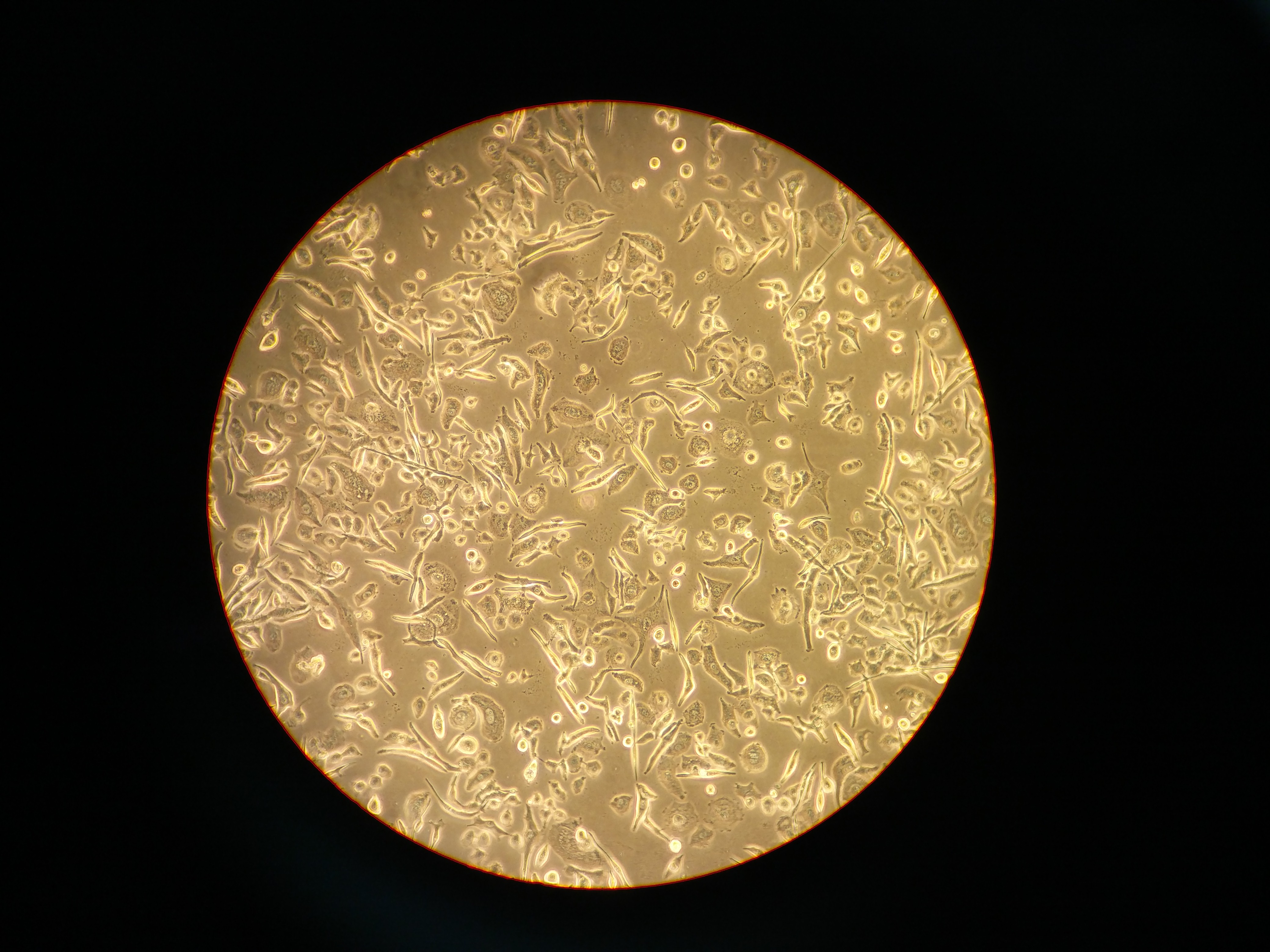

Supplement: Supplementary file 11 — Source data Fig. 3 [file 44321_2024_97_MOESM11_ESM.zip › Fig 3/Fig_3C/TE671_shCTL/TE671_pCTL.jpg]

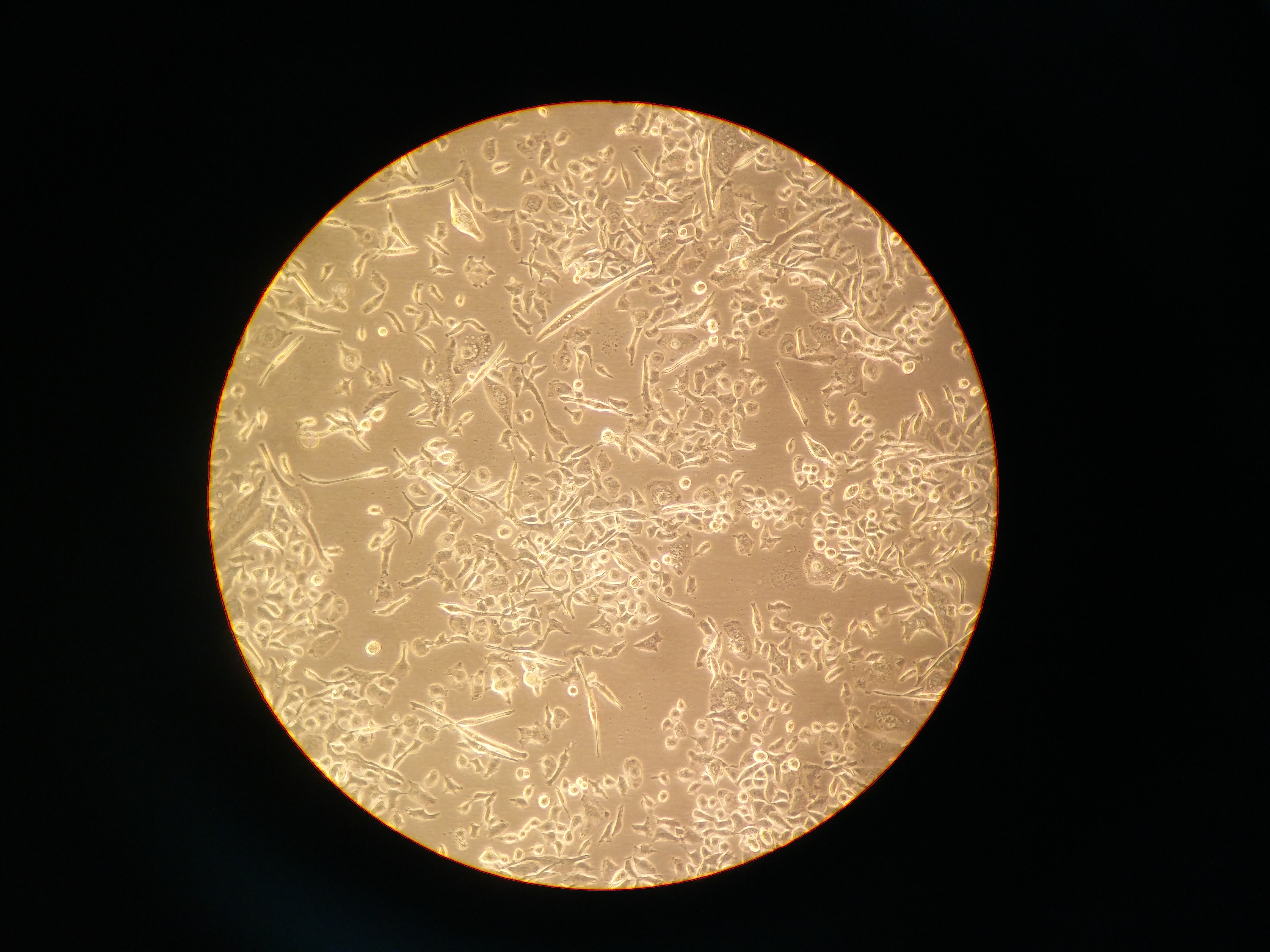

Supplement: Supplementary file 11 — Source data Fig. 3 [file 44321_2024_97_MOESM11_ESM.zip › Fig 3/Fig_3C/TE671_shCTL/TE671_shCtl-1.jpg]

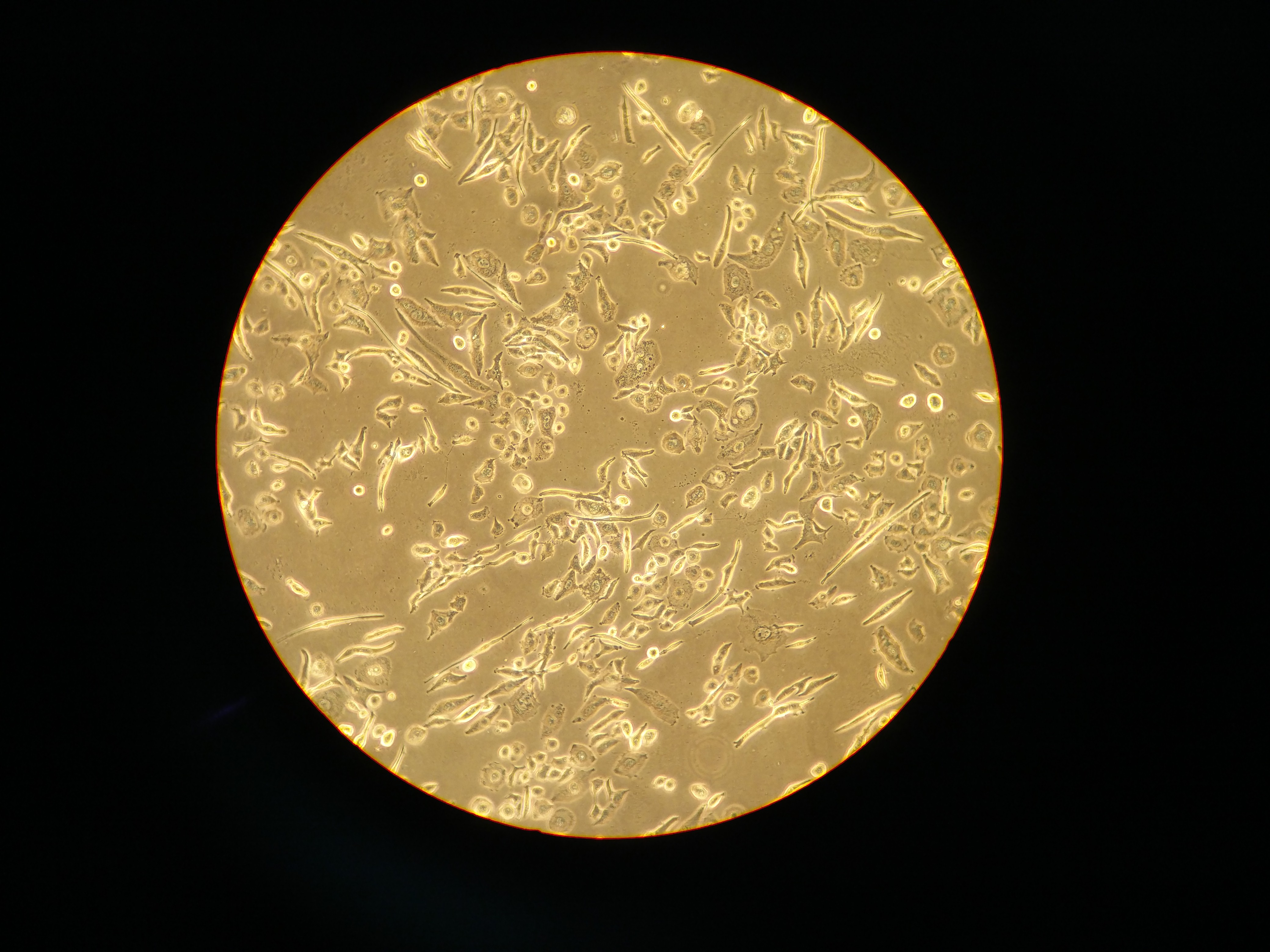

Supplement: Supplementary file 11 — Source data Fig. 3 [file 44321_2024_97_MOESM11_ESM.zip › Fig 3/Fig_3C/TE671_shCTL/TE671_shCtl-3.jpg]

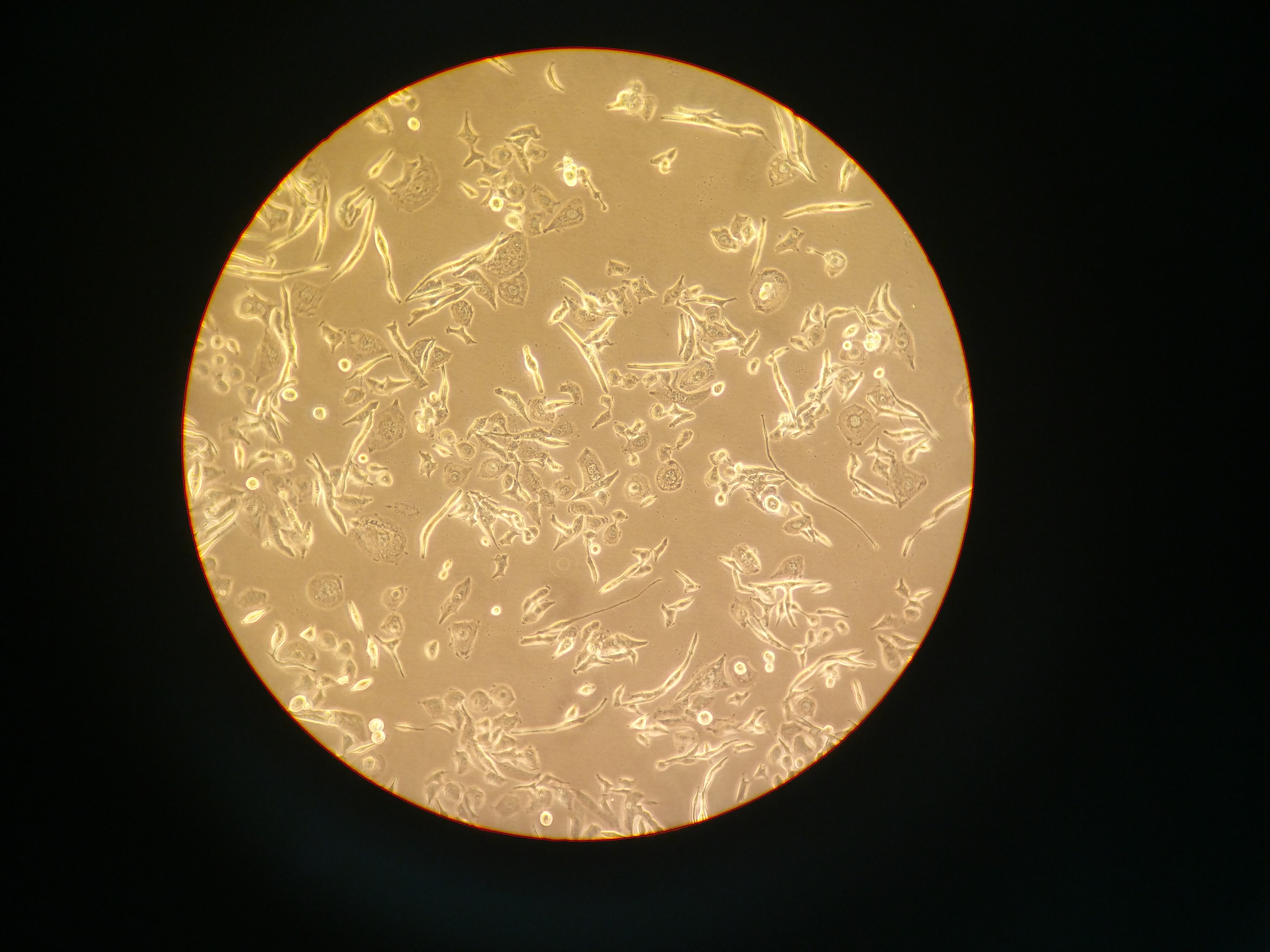

Supplement: Supplementary file 11 — Source data Fig. 3 [file 44321_2024_97_MOESM11_ESM.zip › Fig 3/Fig_3C/TE671_shCTL/TE671_shCTL-4.jpg]

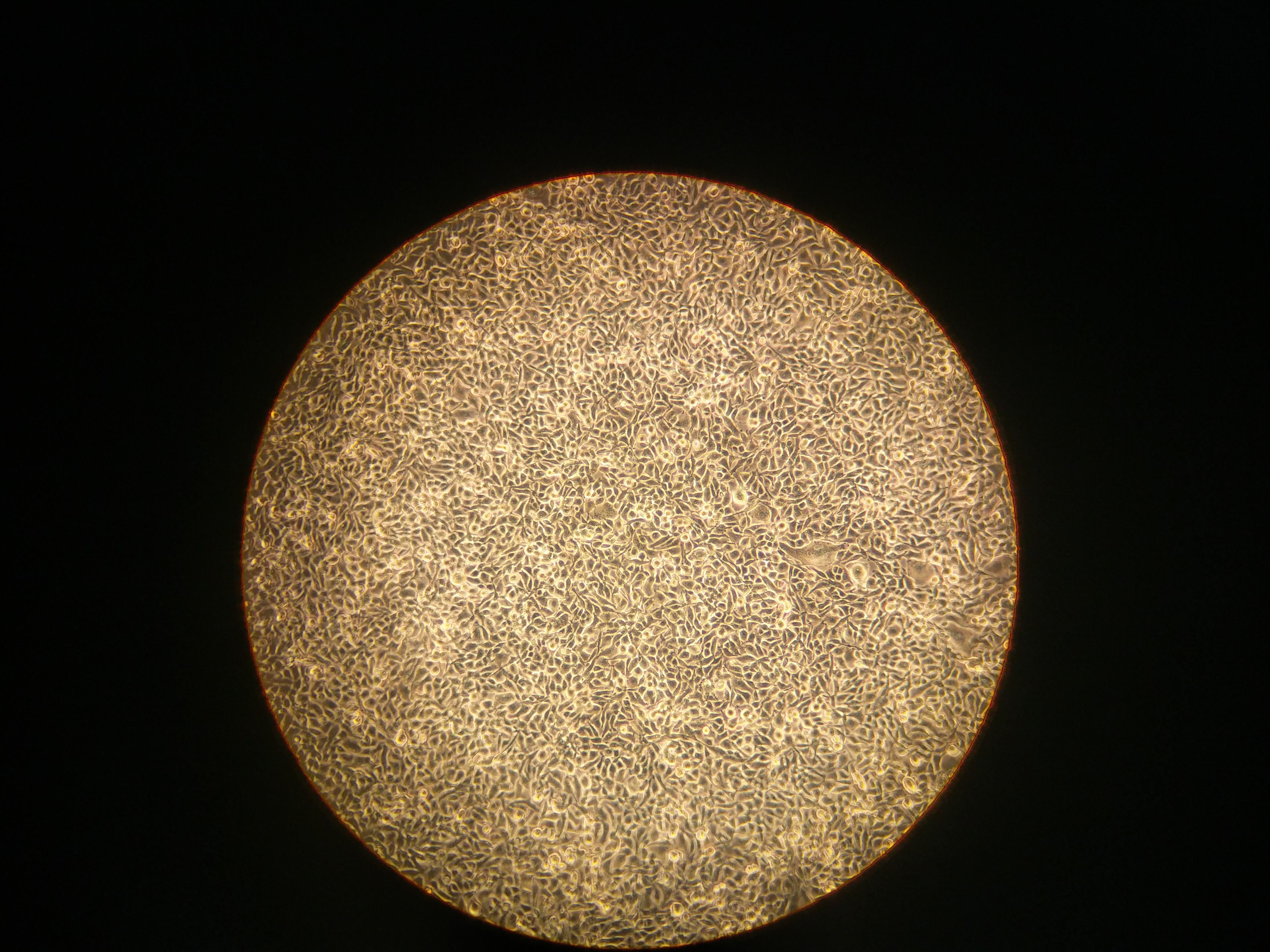

Supplement: Supplementary file 11 — Source data Fig. 3 [file 44321_2024_97_MOESM11_ESM.zip › Fig 3/Fig_3C/TE671_shCTL/TE671_shCTL-5.jpg]

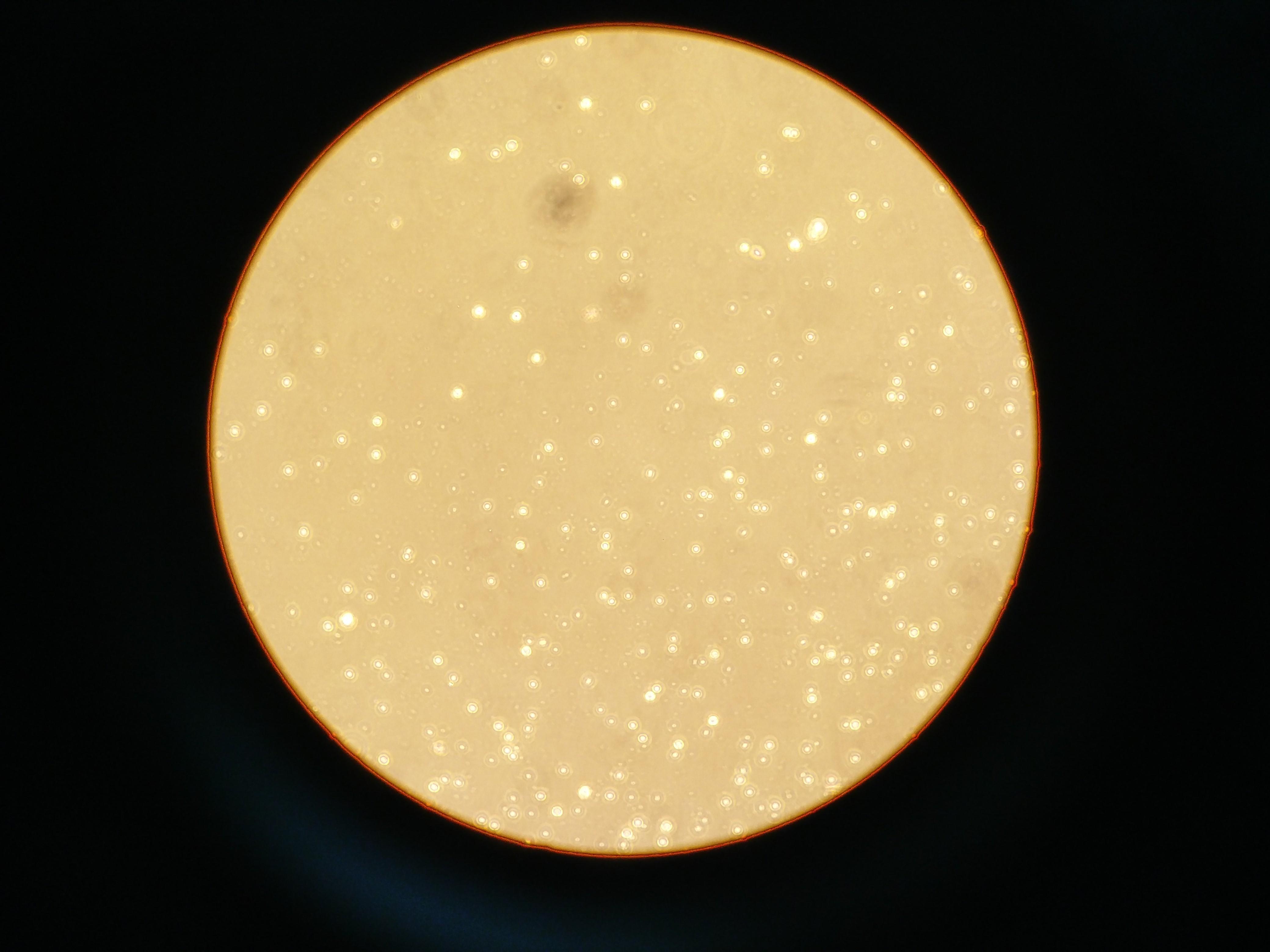

Supplement: Supplementary file 11 — Source data Fig. 3 [file 44321_2024_97_MOESM11_ESM.zip › Fig 3/Fig_3C/TE671_shDIPRO1/TE671_sh1.jpg]

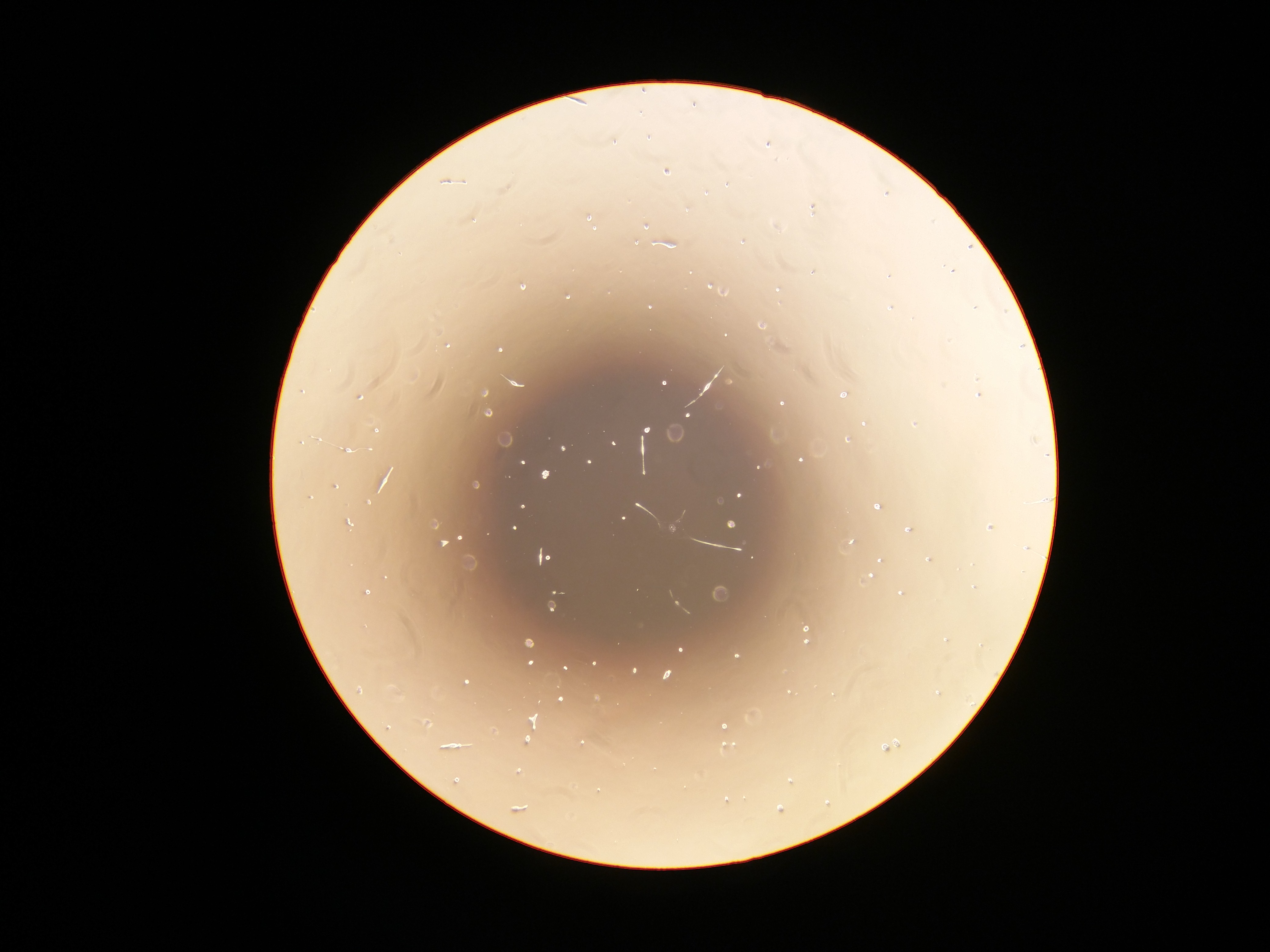

Supplement: Supplementary file 11 — Source data Fig. 3 [file 44321_2024_97_MOESM11_ESM.zip › Fig 3/Fig_3C/TE671_shDIPRO1/TE671_sh2-1.jpg]

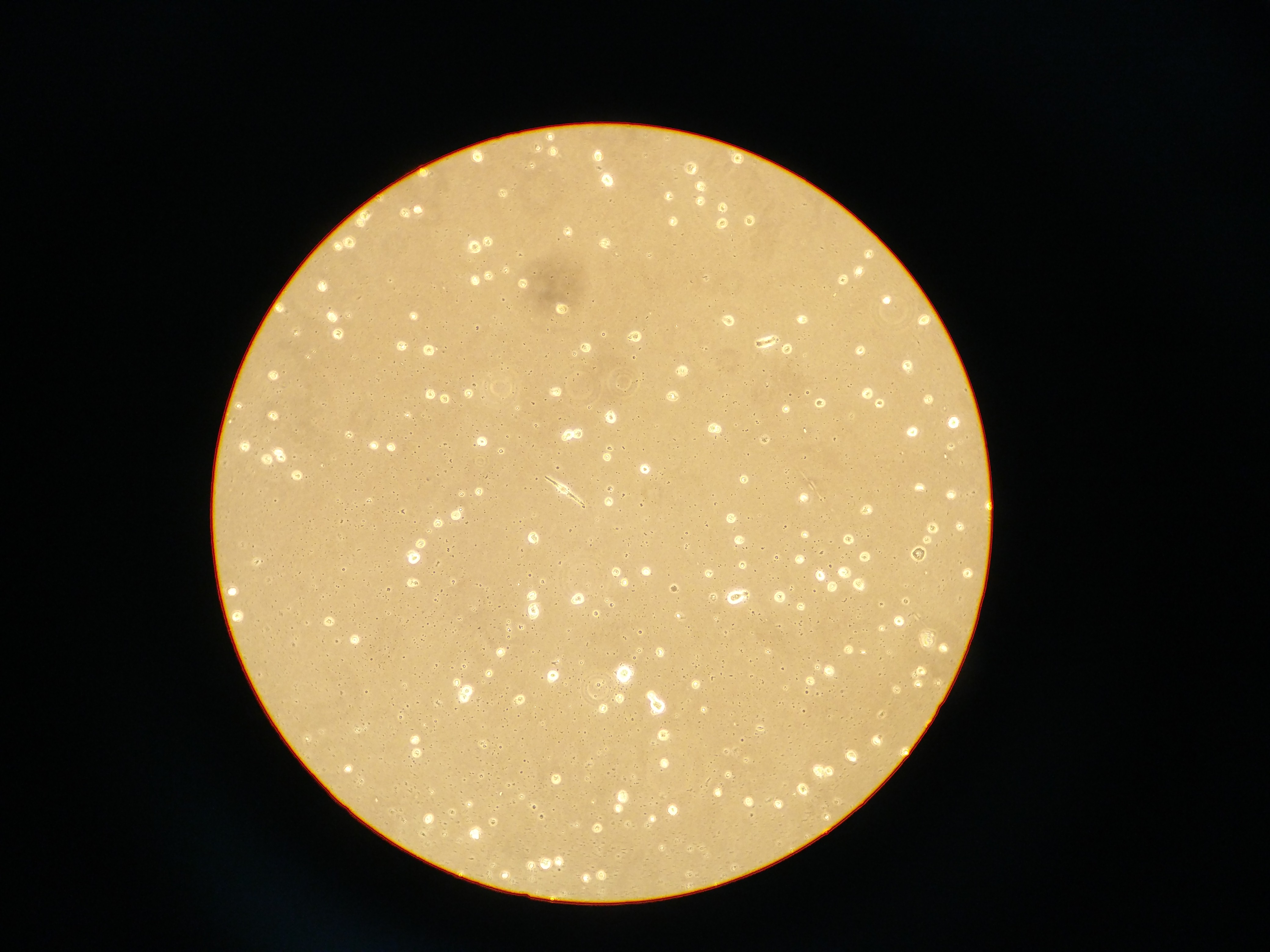

Supplement: Supplementary file 11 — Source data Fig. 3 [file 44321_2024_97_MOESM11_ESM.zip › Fig 3/Fig_3C/TE671_shDIPRO1/TE671_sh2.jpg]

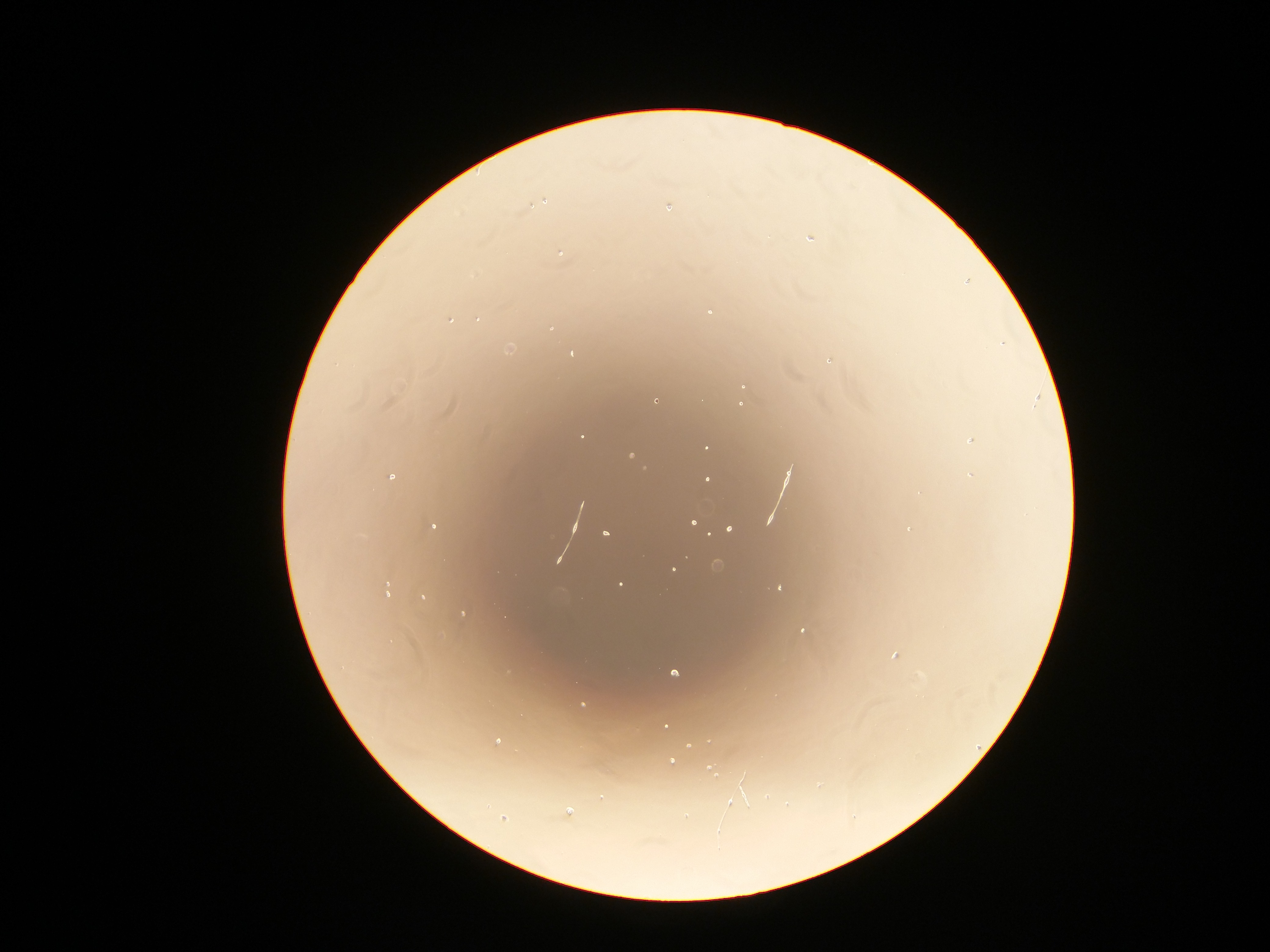

Supplement: Supplementary file 11 — Source data Fig. 3 [file 44321_2024_97_MOESM11_ESM.zip › Fig 3/Fig_3C/TE671_shDIPRO1/TE671_sh3-1.jpg]

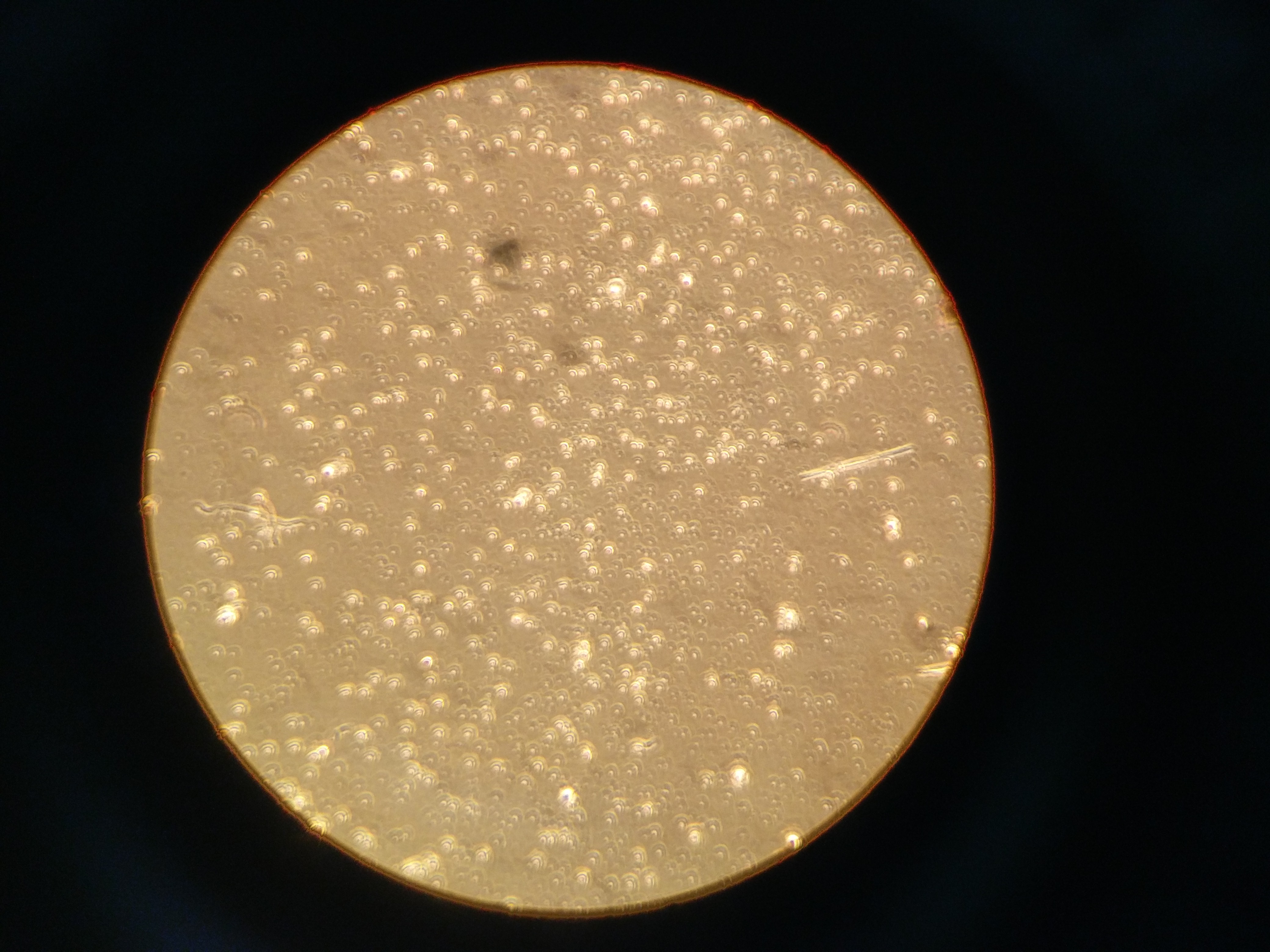

Supplement: Supplementary file 11 — Source data Fig. 3 [file 44321_2024_97_MOESM11_ESM.zip › Fig 3/Fig_3C/TE671_shDIPRO1/TE671_sh3.jpg]

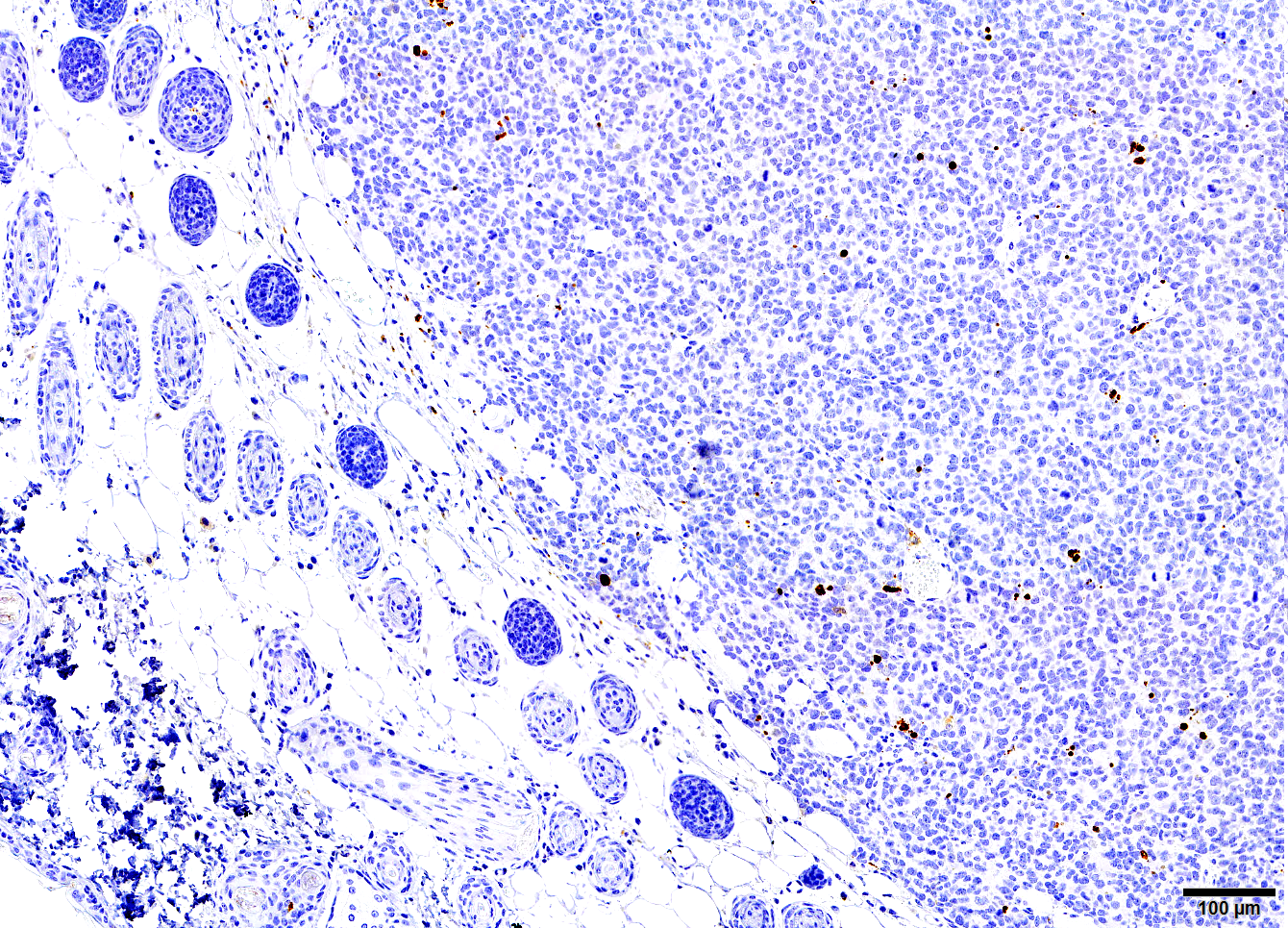

Supplement: Supplementary file 12 — Source data Fig. 4 [file 44321_2024_97_MOESM12_ESM.zip › Fig 4/Fig_4G/Caspase 3/Caspase_CTL_100.png]

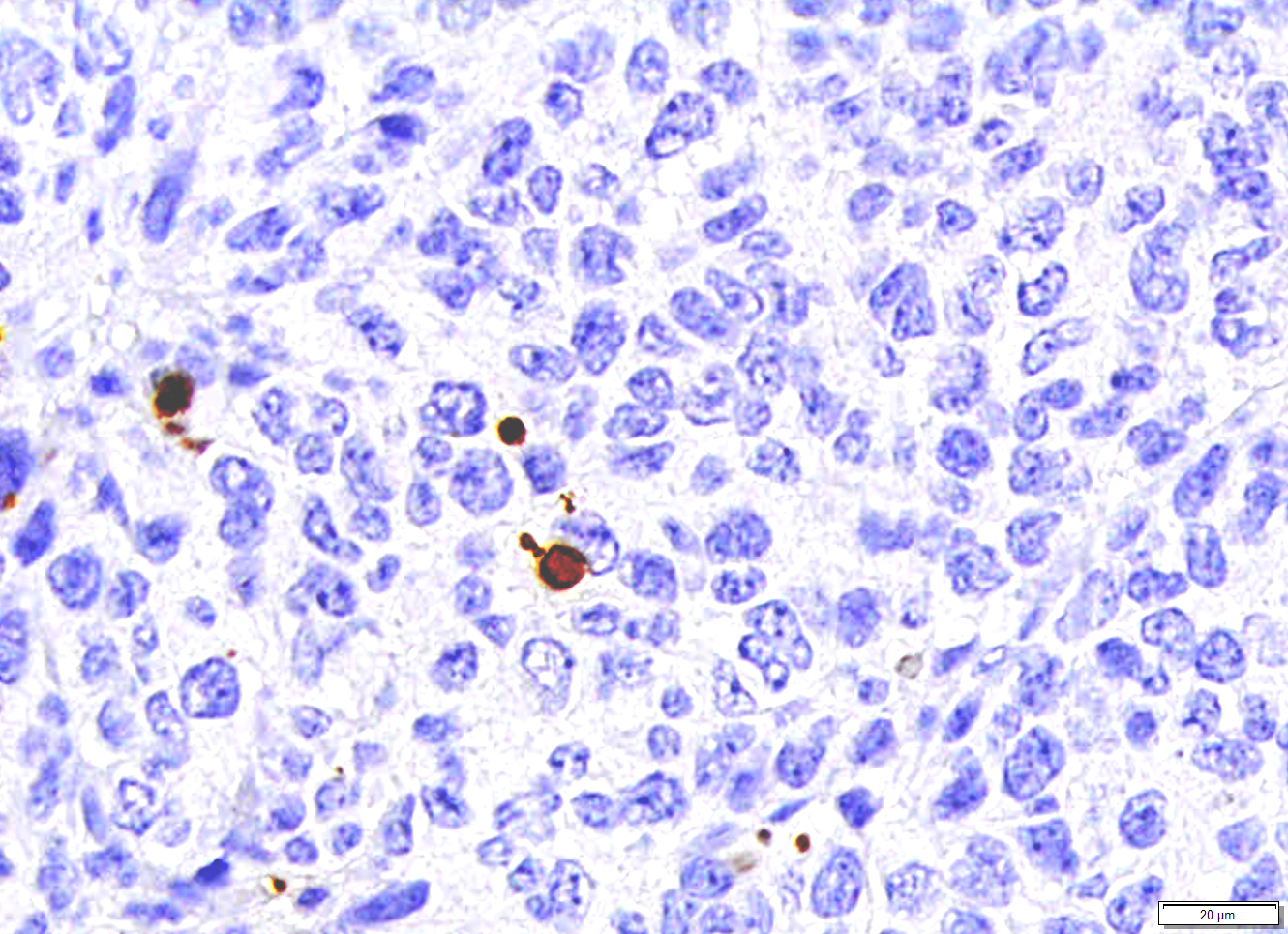

Supplement: Supplementary file 12 — Source data Fig. 4 [file 44321_2024_97_MOESM12_ESM.zip › Fig 4/Fig_4G/Caspase 3/Caspase_CTL_20.png]

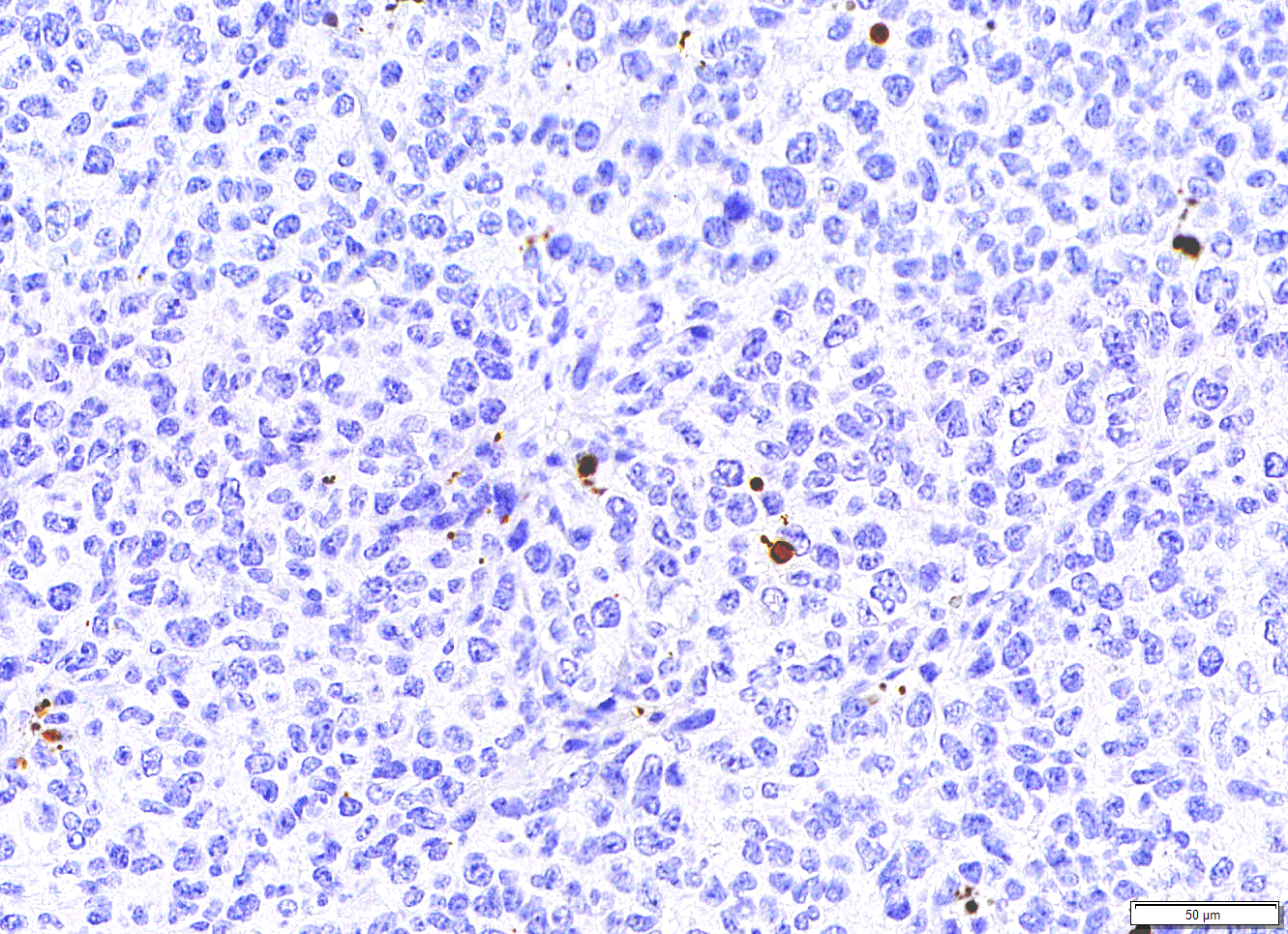

Supplement: Supplementary file 12 — Source data Fig. 4 [file 44321_2024_97_MOESM12_ESM.zip › Fig 4/Fig_4G/Caspase 3/Caspase_CTL_50.png]

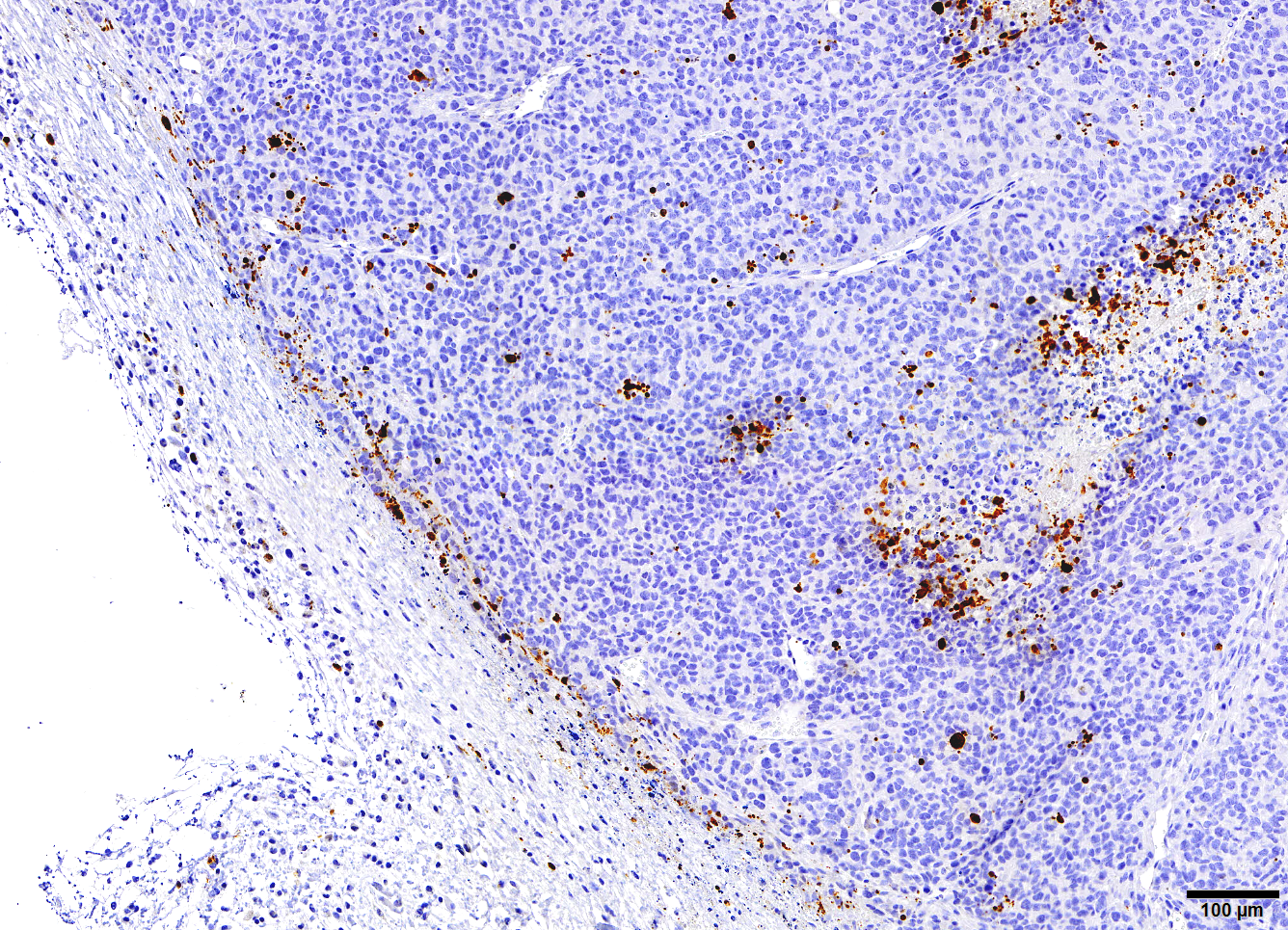

Supplement: Supplementary file 12 — Source data Fig. 4 [file 44321_2024_97_MOESM12_ESM.zip › Fig 4/Fig_4G/Caspase 3/Caspase_sh1_100.png]

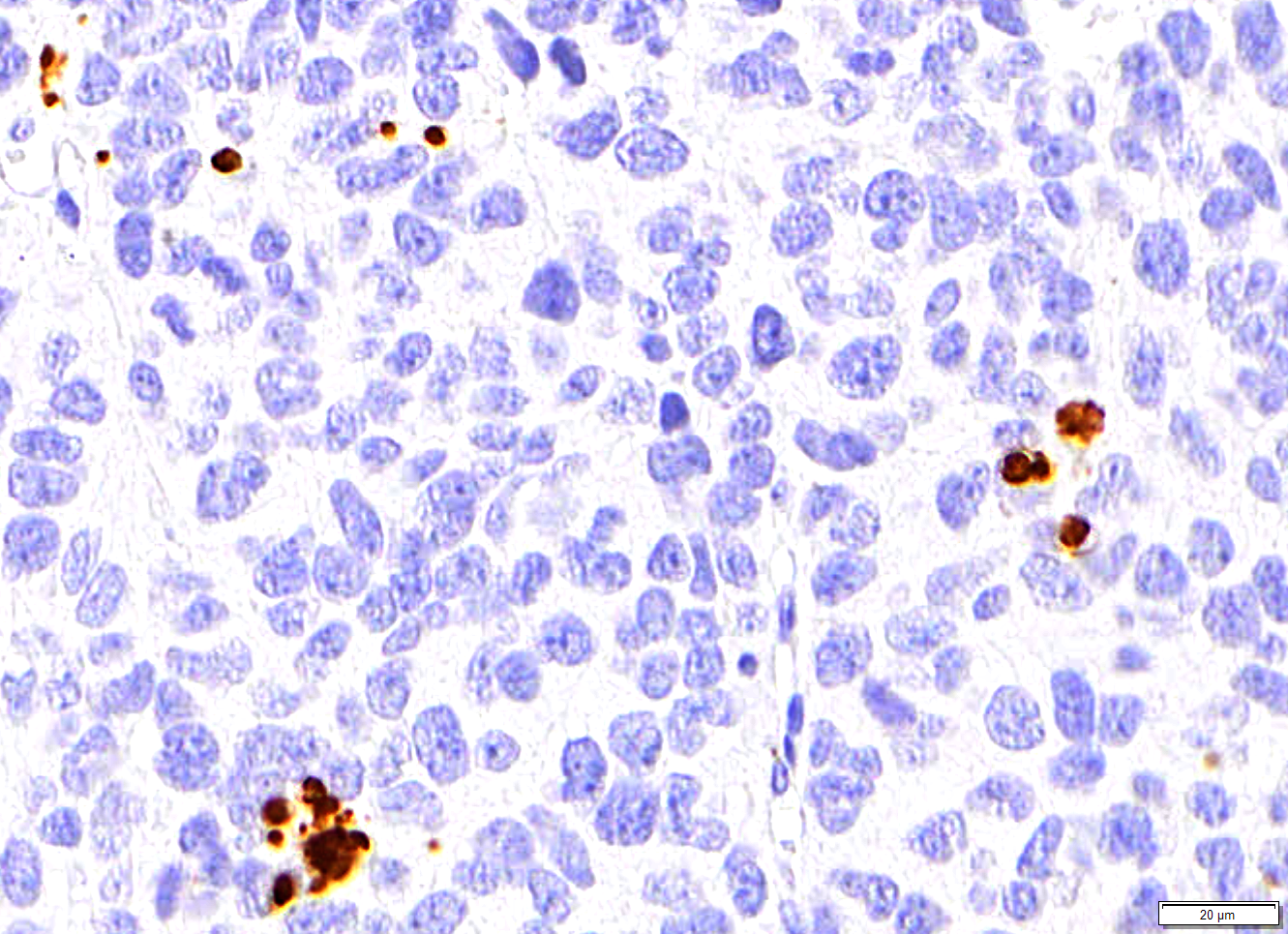

Supplement: Supplementary file 12 — Source data Fig. 4 [file 44321_2024_97_MOESM12_ESM.zip › Fig 4/Fig_4G/Caspase 3/Caspase_sh1_20.png]

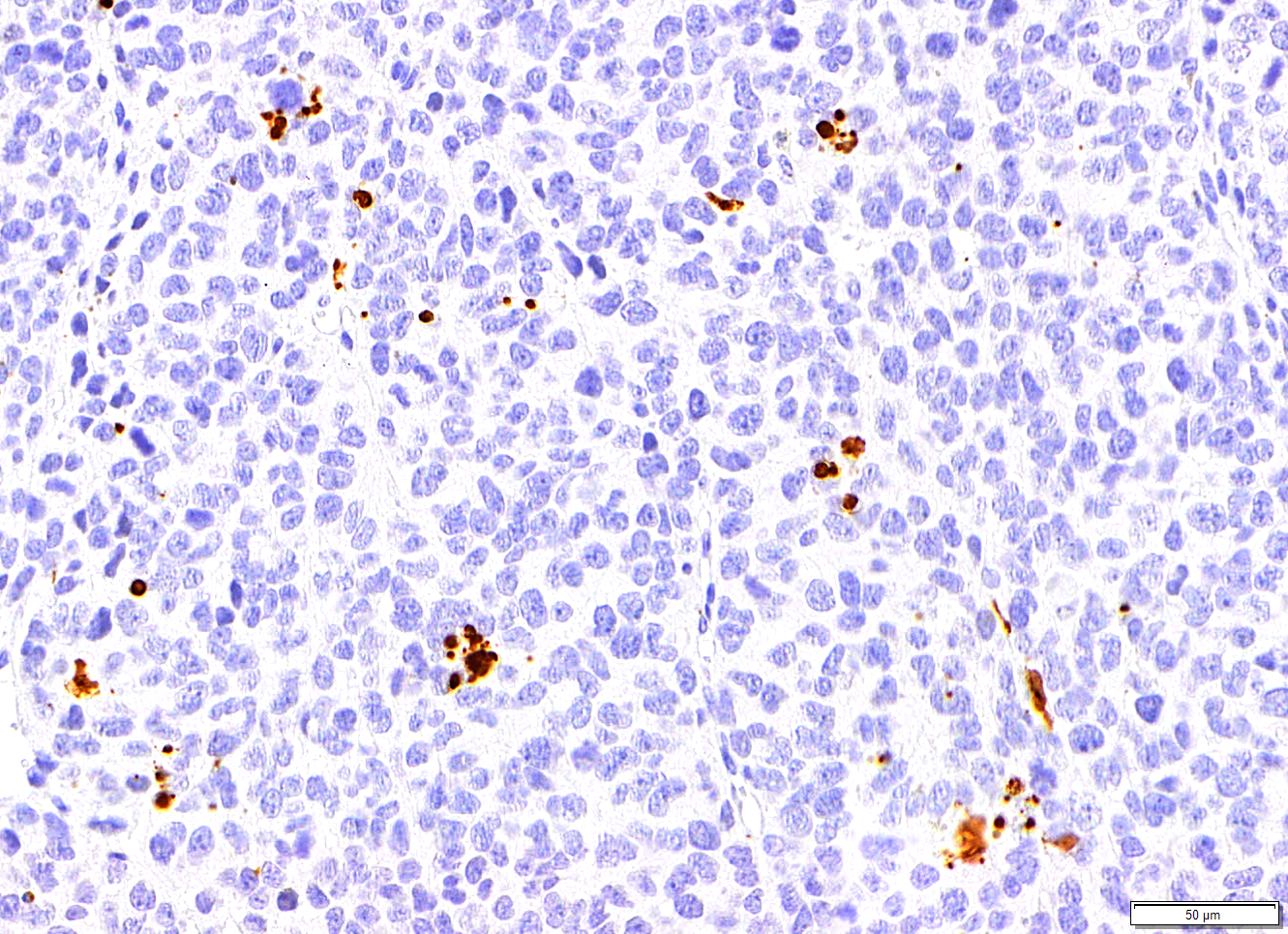

Supplement: Supplementary file 12 — Source data Fig. 4 [file 44321_2024_97_MOESM12_ESM.zip › Fig 4/Fig_4G/Caspase 3/Caspase_sh1_50.png]

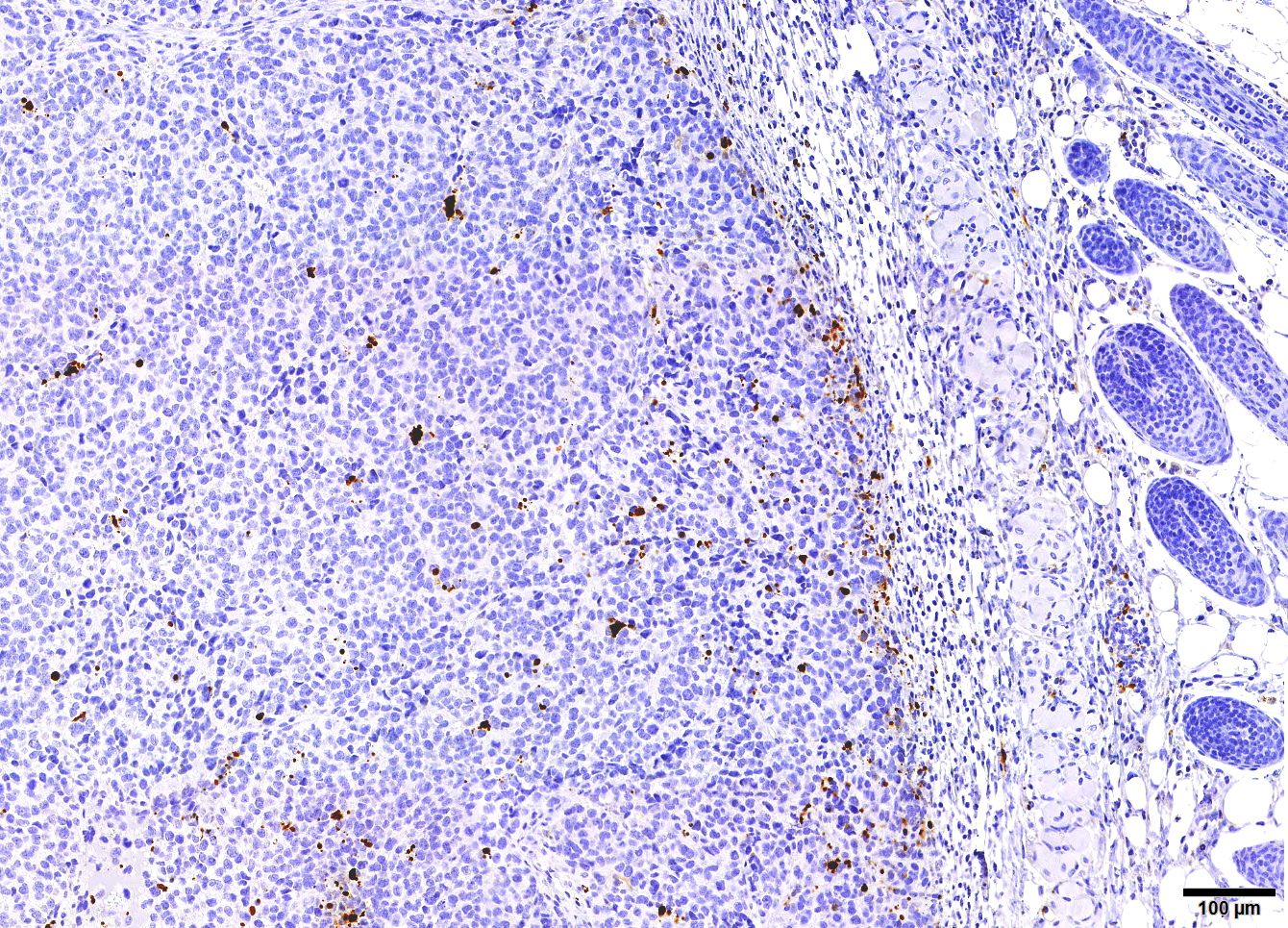

Supplement: Supplementary file 12 — Source data Fig. 4 [file 44321_2024_97_MOESM12_ESM.zip › Fig 4/Fig_4G/Caspase 3/Caspase_sh2_100.png]

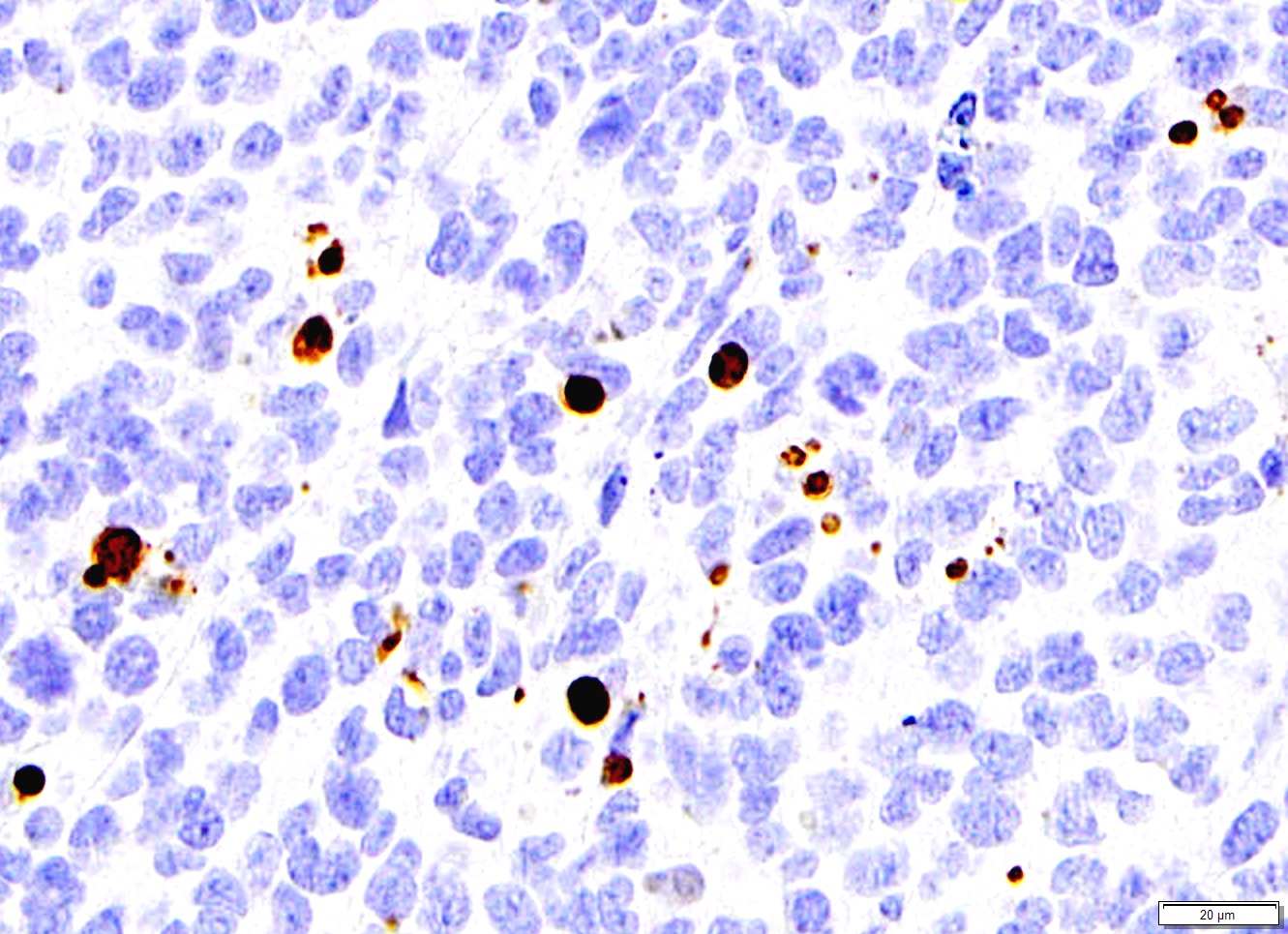

Supplement: Supplementary file 12 — Source data Fig. 4 [file 44321_2024_97_MOESM12_ESM.zip › Fig 4/Fig_4G/Caspase 3/Caspase_sh2_20.png]

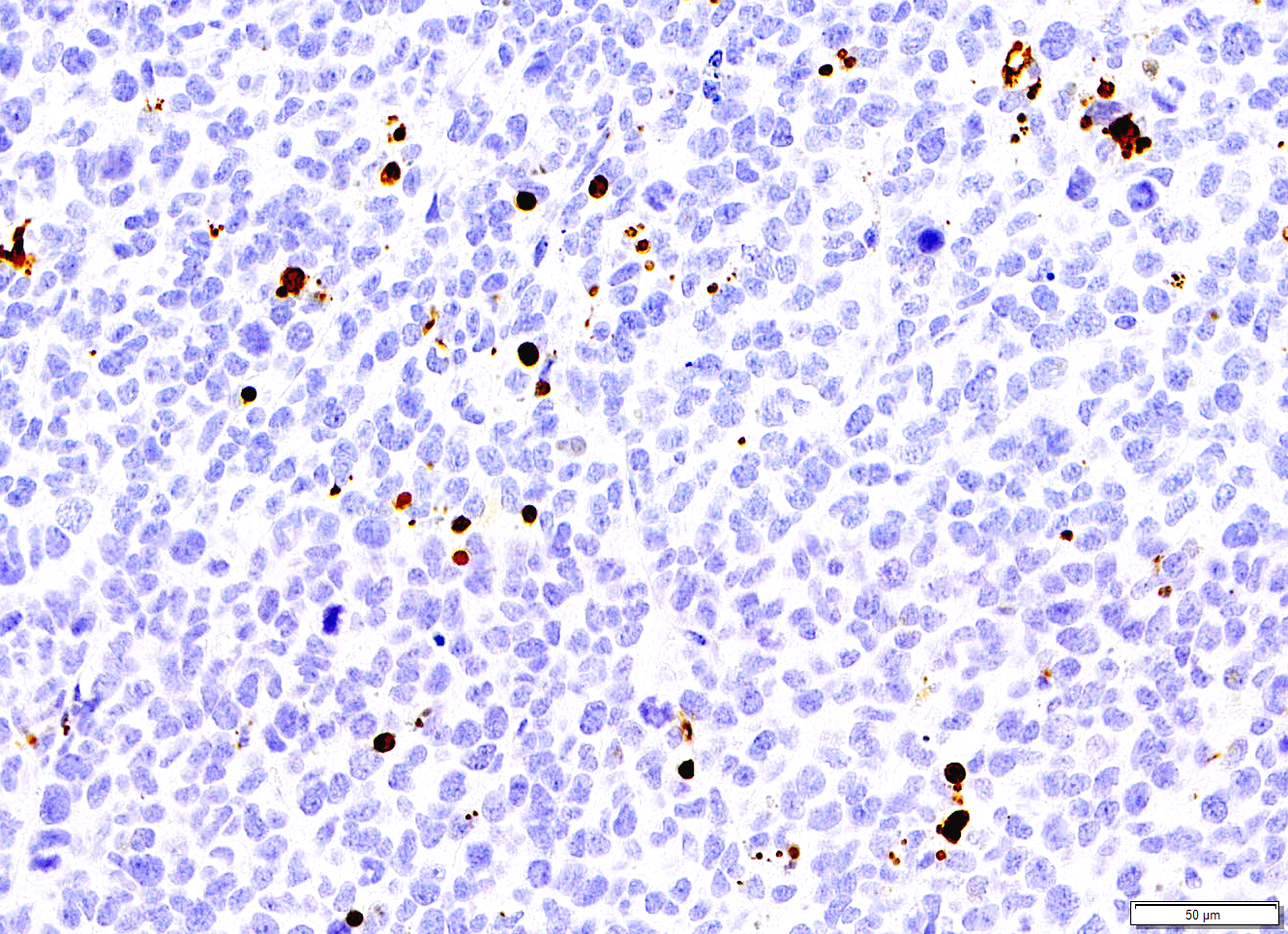

Supplement: Supplementary file 12 — Source data Fig. 4 [file 44321_2024_97_MOESM12_ESM.zip › Fig 4/Fig_4G/Caspase 3/Caspase_sh2_50.png]

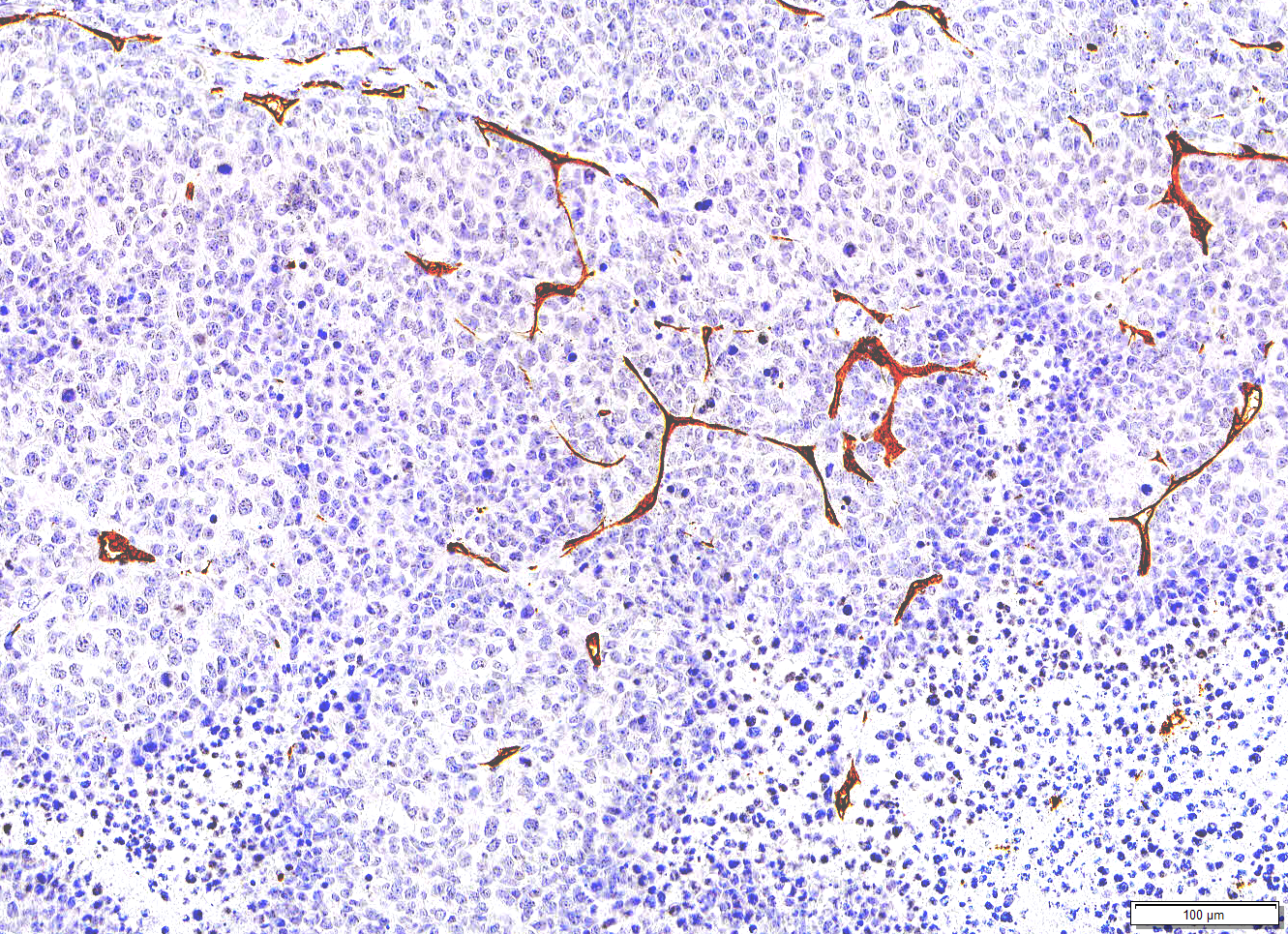

Supplement: Supplementary file 12 — Source data Fig. 4 [file 44321_2024_97_MOESM12_ESM.zip › Fig 4/Fig_4G/CD34/CD34 Ctl 100.png]

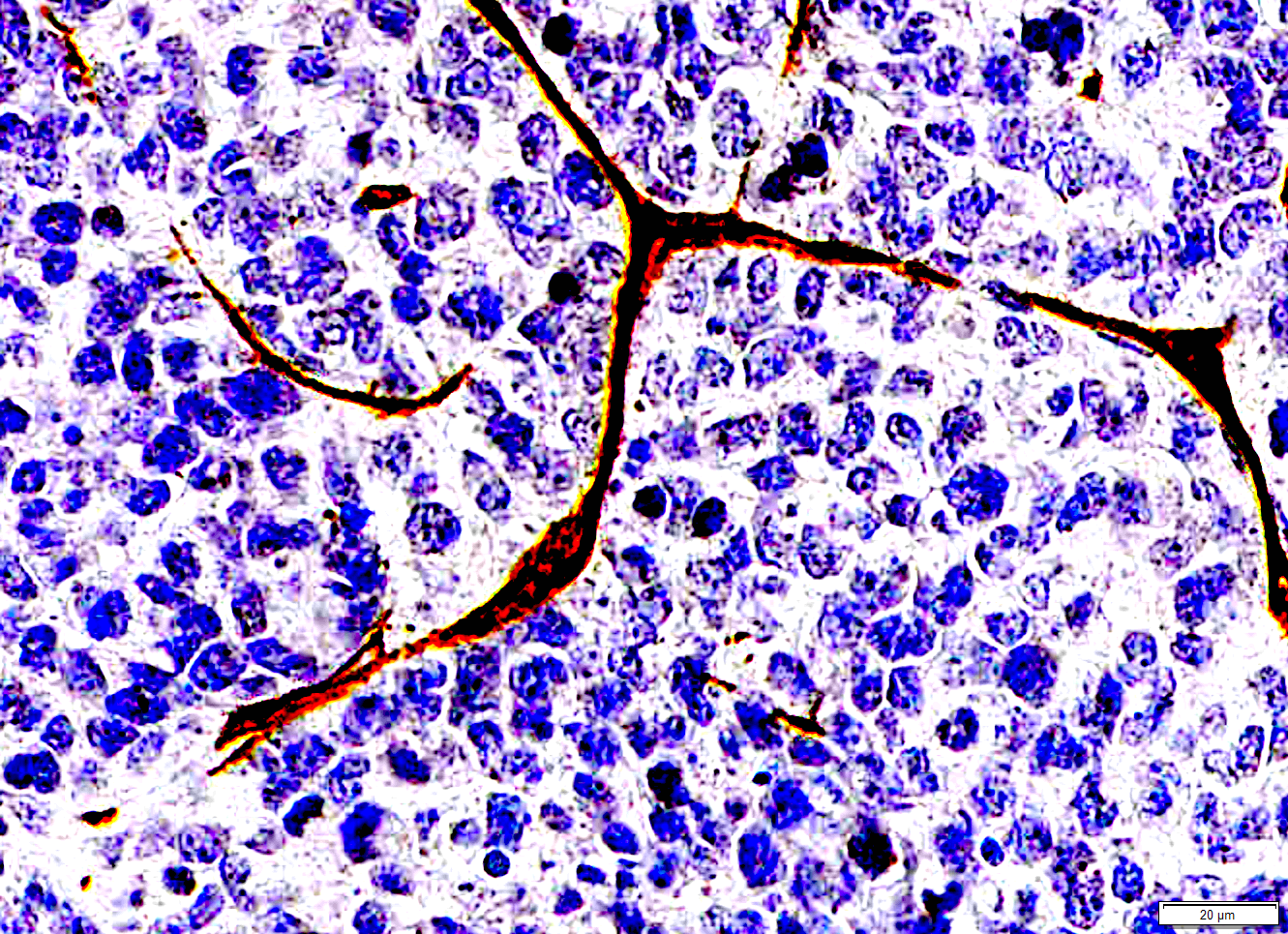

Supplement: Supplementary file 12 — Source data Fig. 4 [file 44321_2024_97_MOESM12_ESM.zip › Fig 4/Fig_4G/CD34/CD34 Ctl 20.png]

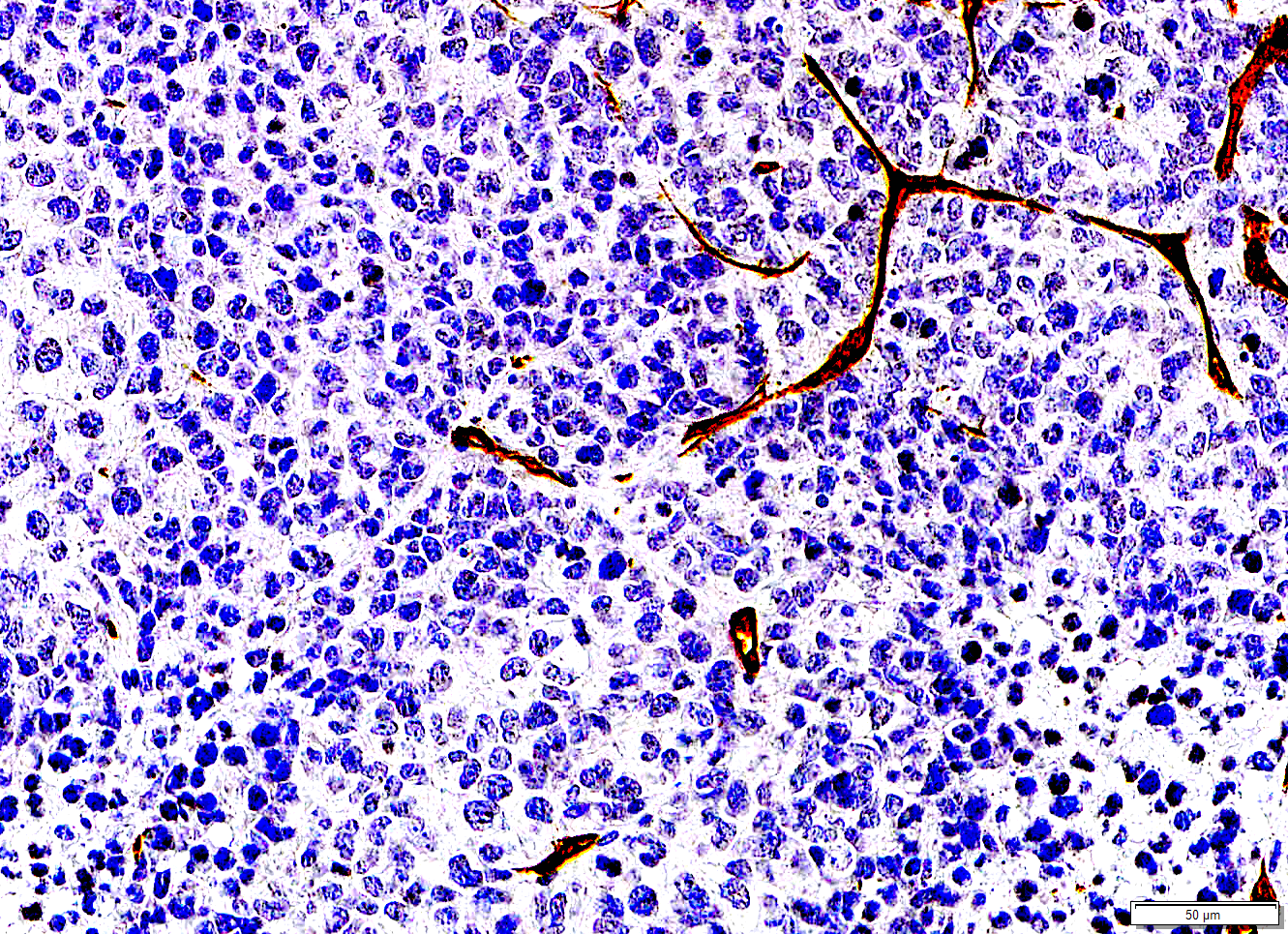

Supplement: Supplementary file 12 — Source data Fig. 4 [file 44321_2024_97_MOESM12_ESM.zip › Fig 4/Fig_4G/CD34/CD34 Ctl 50.png]

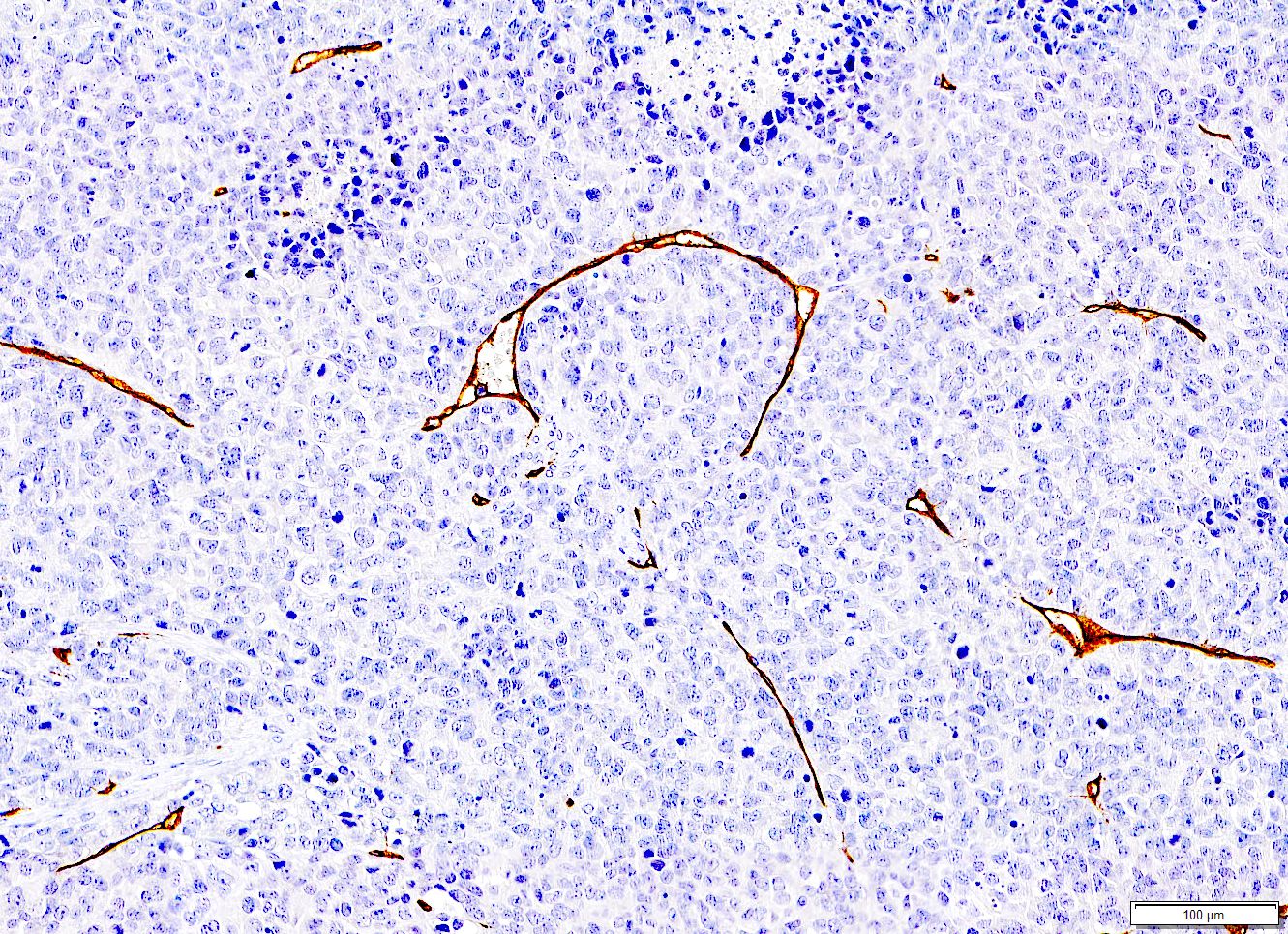

Supplement: Supplementary file 12 — Source data Fig. 4 [file 44321_2024_97_MOESM12_ESM.zip › Fig 4/Fig_4G/CD34/CD34 sh1 100.png]

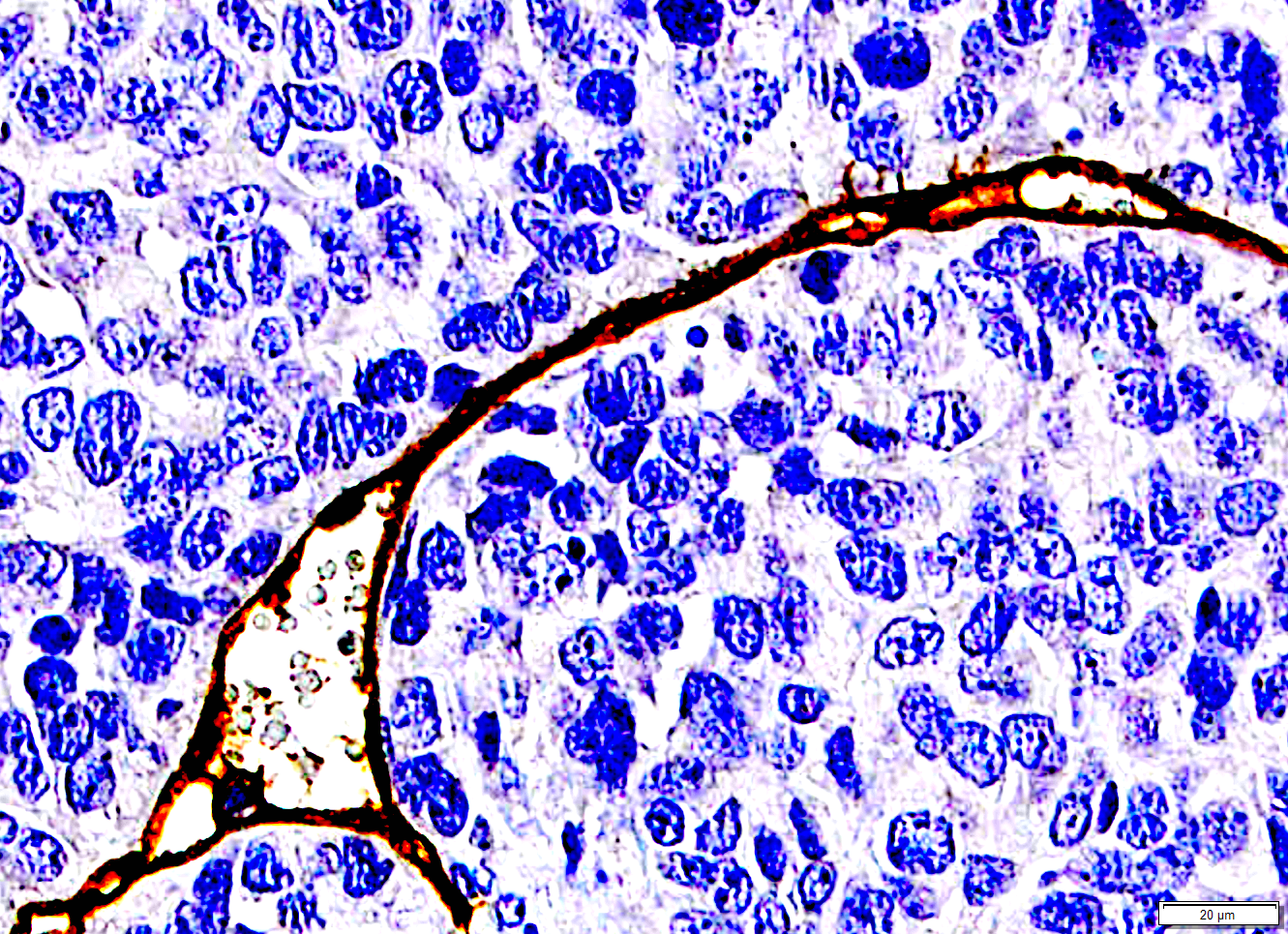

Supplement: Supplementary file 12 — Source data Fig. 4 [file 44321_2024_97_MOESM12_ESM.zip › Fig 4/Fig_4G/CD34/CD34 sh1 20.png]

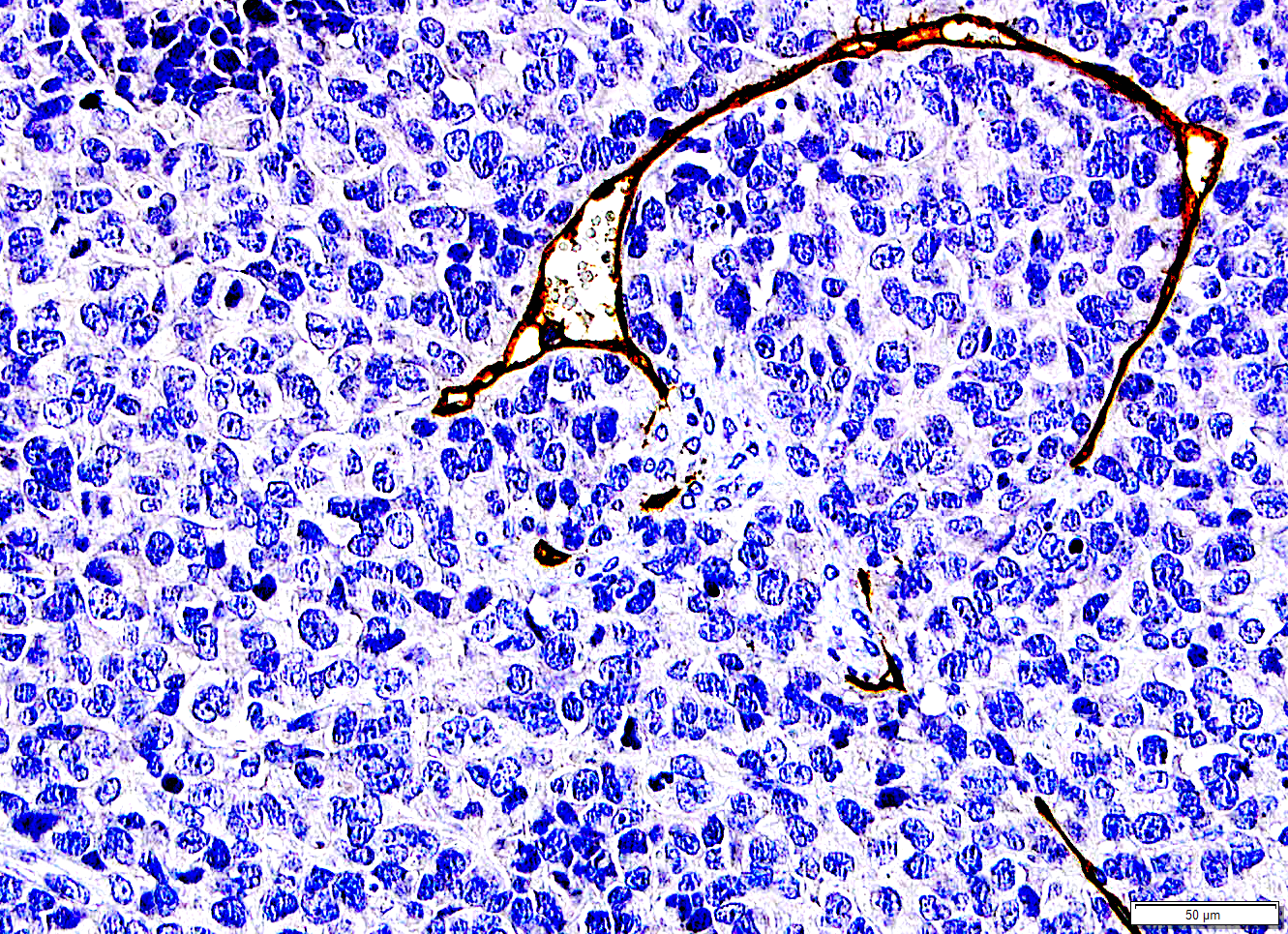

Supplement: Supplementary file 12 — Source data Fig. 4 [file 44321_2024_97_MOESM12_ESM.zip › Fig 4/Fig_4G/CD34/CD34 sh1 50.png]

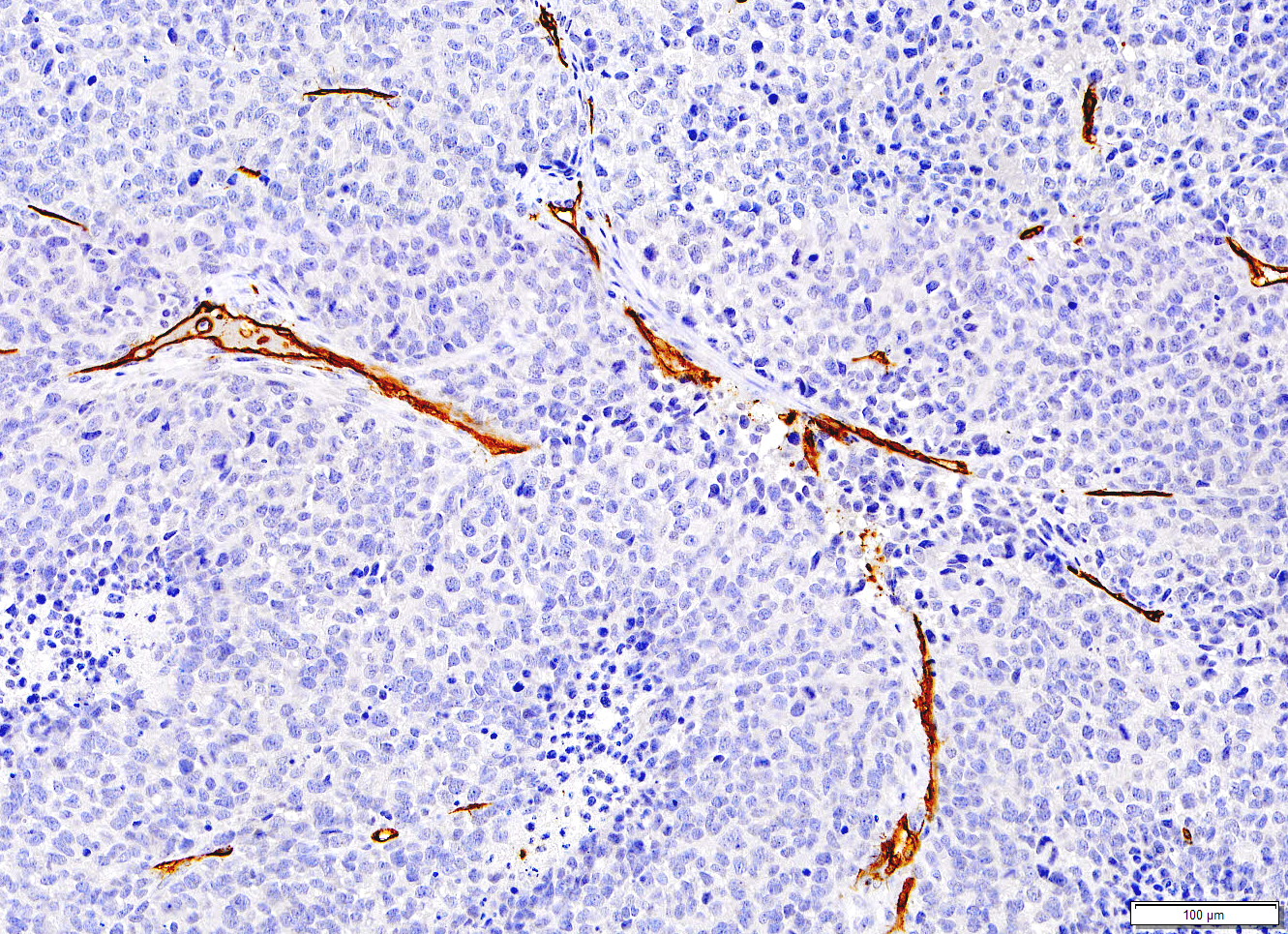

Supplement: Supplementary file 12 — Source data Fig. 4 [file 44321_2024_97_MOESM12_ESM.zip › Fig 4/Fig_4G/CD34/CD34 sh2 100.png]

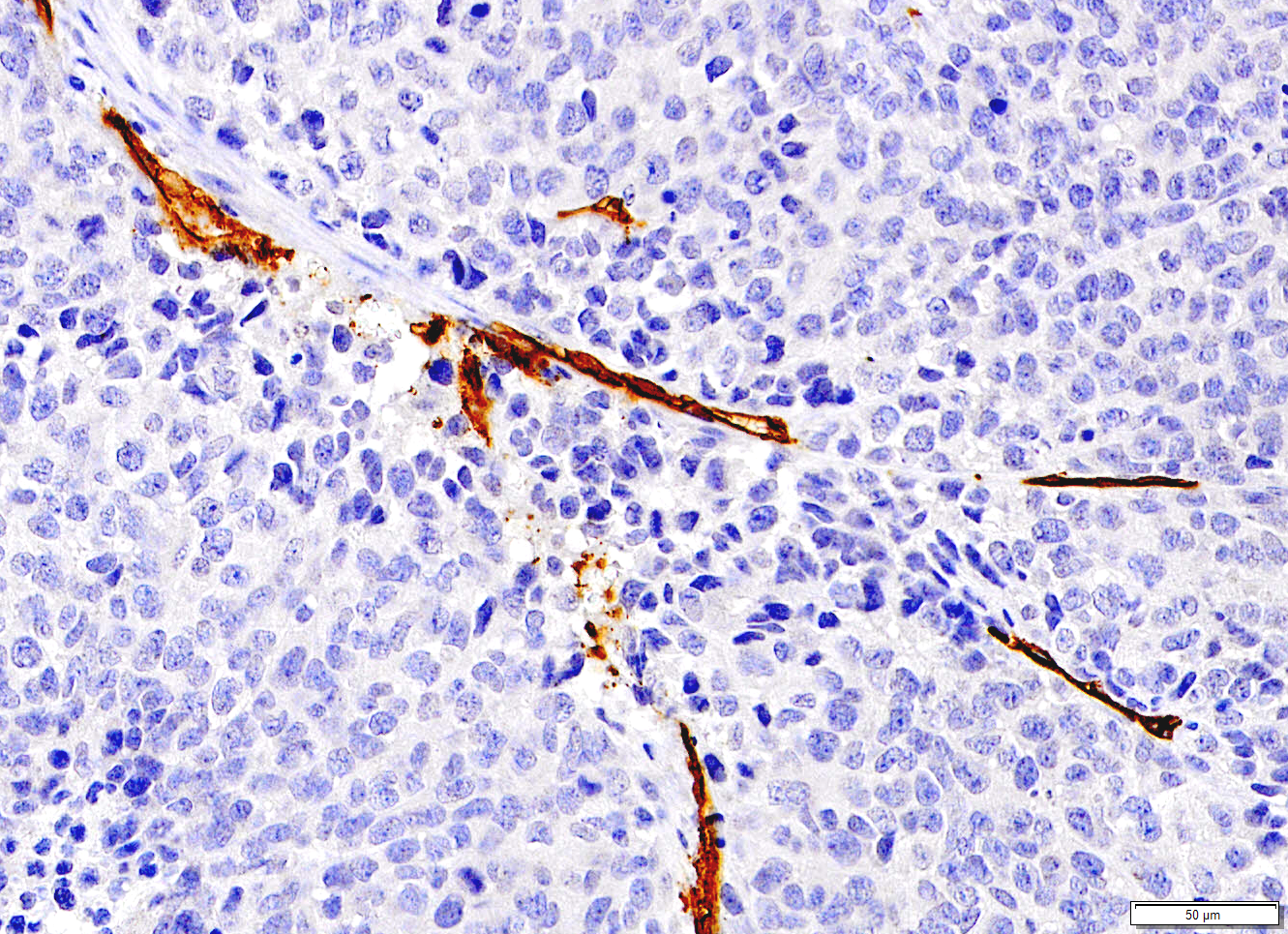

Supplement: Supplementary file 12 — Source data Fig. 4 [file 44321_2024_97_MOESM12_ESM.zip › Fig 4/Fig_4G/CD34/CD34 sh2 20.png]

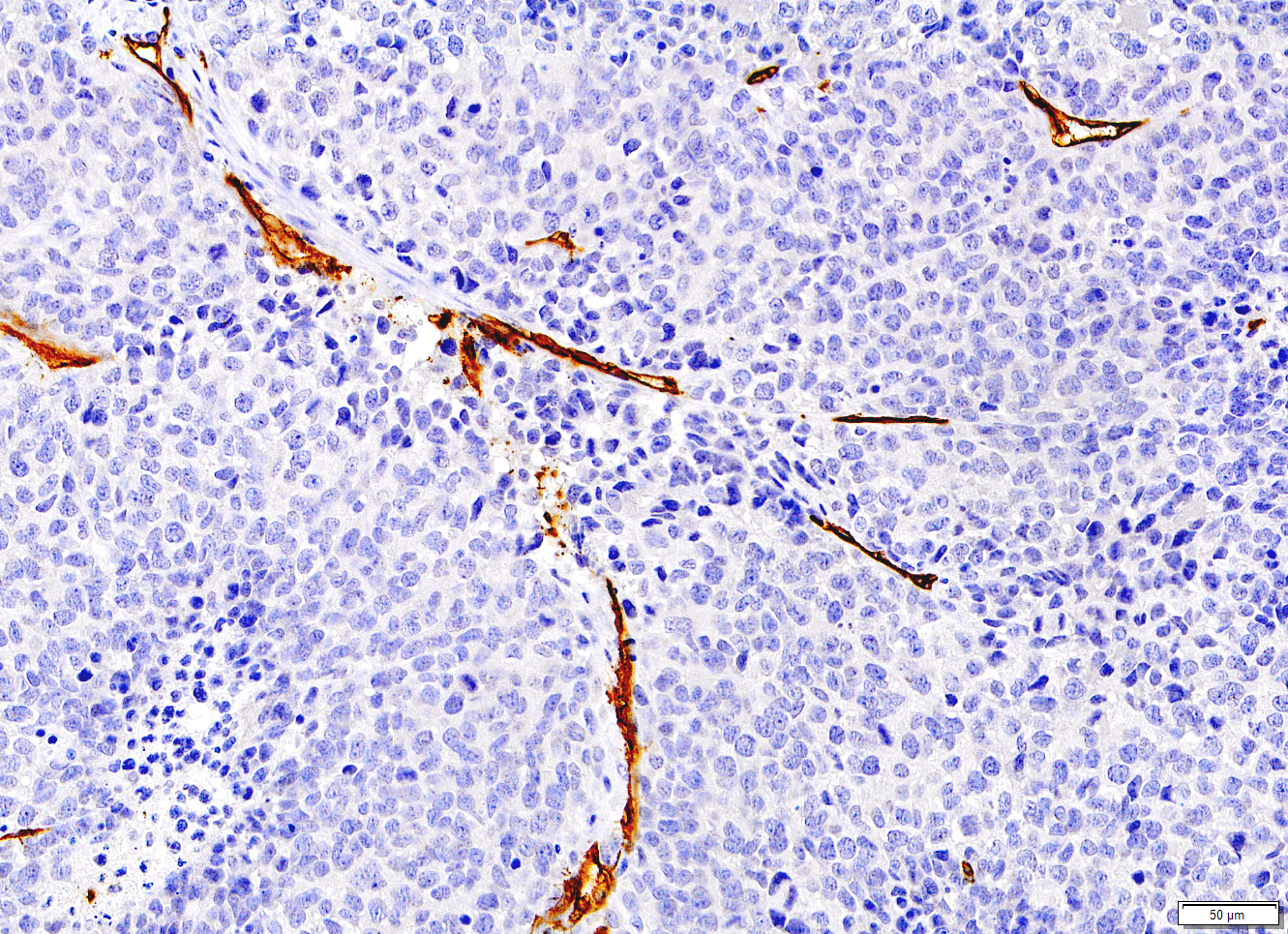

Supplement: Supplementary file 12 — Source data Fig. 4 [file 44321_2024_97_MOESM12_ESM.zip › Fig 4/Fig_4G/CD34/CD34 sh2 50.png]

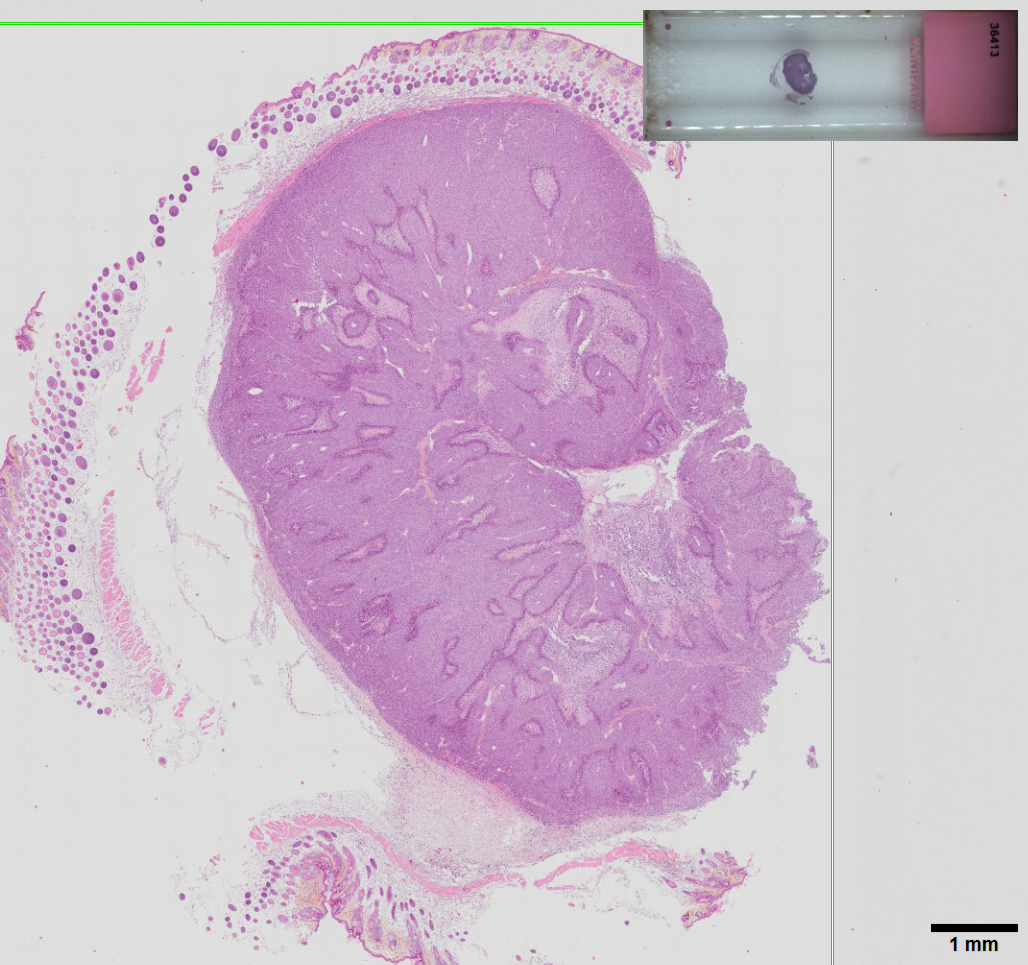

Supplement: Supplementary file 12 — Source data Fig. 4 [file 44321_2024_97_MOESM12_ESM.zip › Fig 4/Fig_4G/HEC sh1_1 mm.tif]

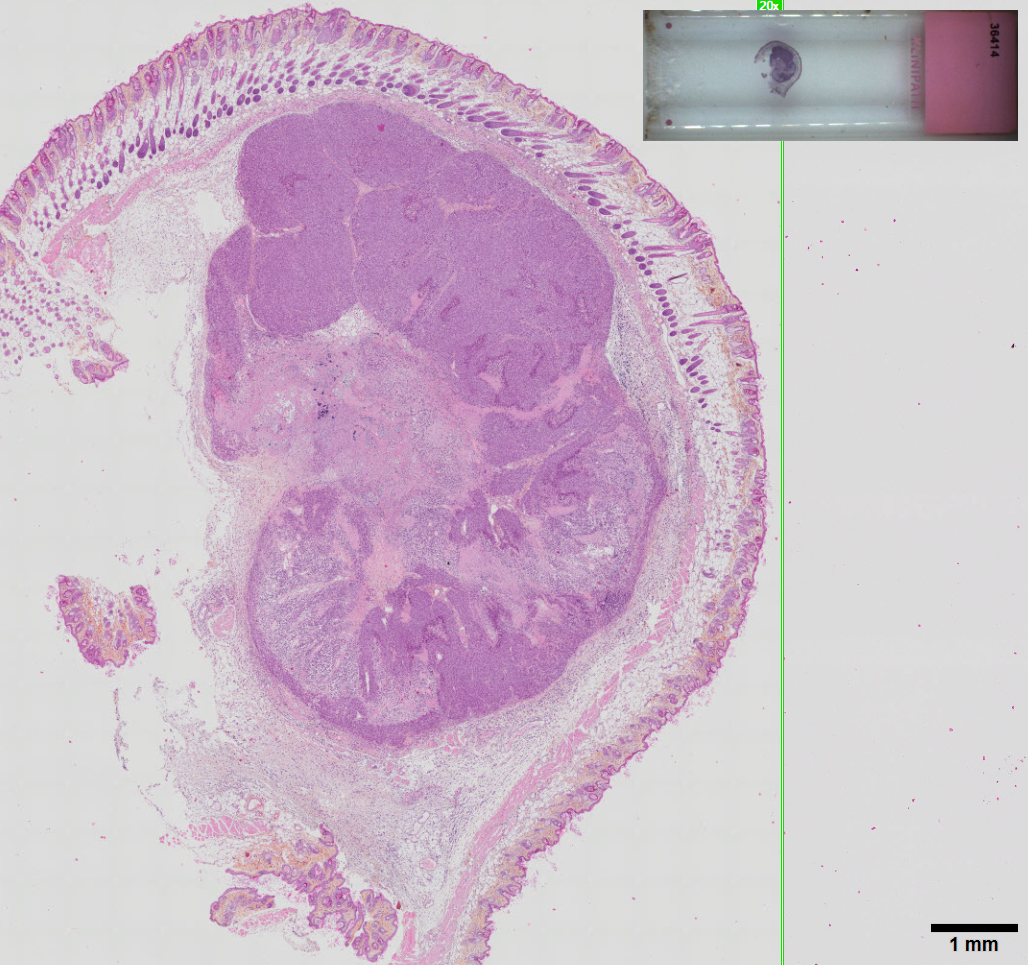

Supplement: Supplementary file 12 — Source data Fig. 4 [file 44321_2024_97_MOESM12_ESM.zip › Fig 4/Fig_4G/HEC_sh2_1 mm.tif]

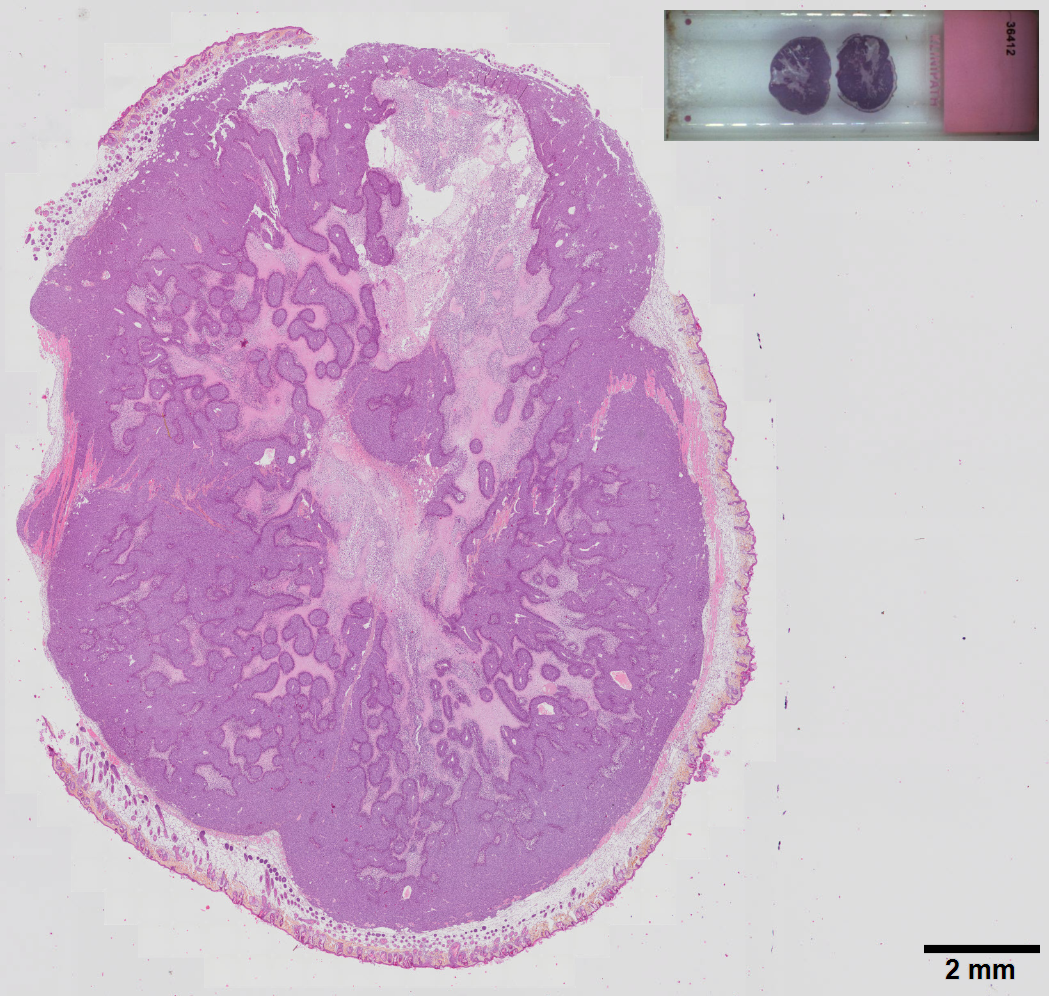

Supplement: Supplementary file 12 — Source data Fig. 4 [file 44321_2024_97_MOESM12_ESM.zip › Fig 4/Fig_4G/HES Ctl 2mm.png]

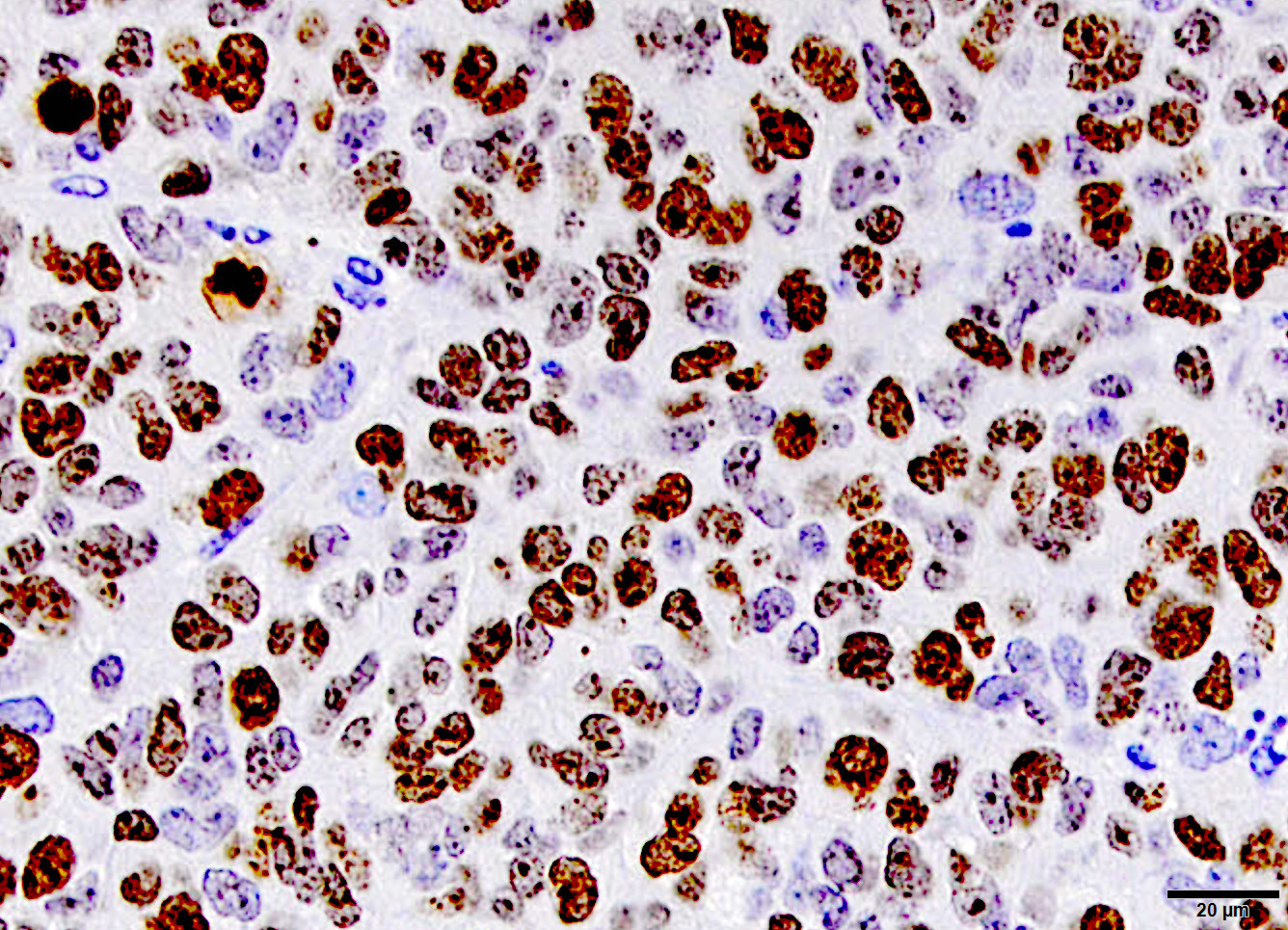

Supplement: Supplementary file 12 — Source data Fig. 4 [file 44321_2024_97_MOESM12_ESM.zip › Fig 4/Fig_4G/Ki67/Ki67 Ctl 20.png]

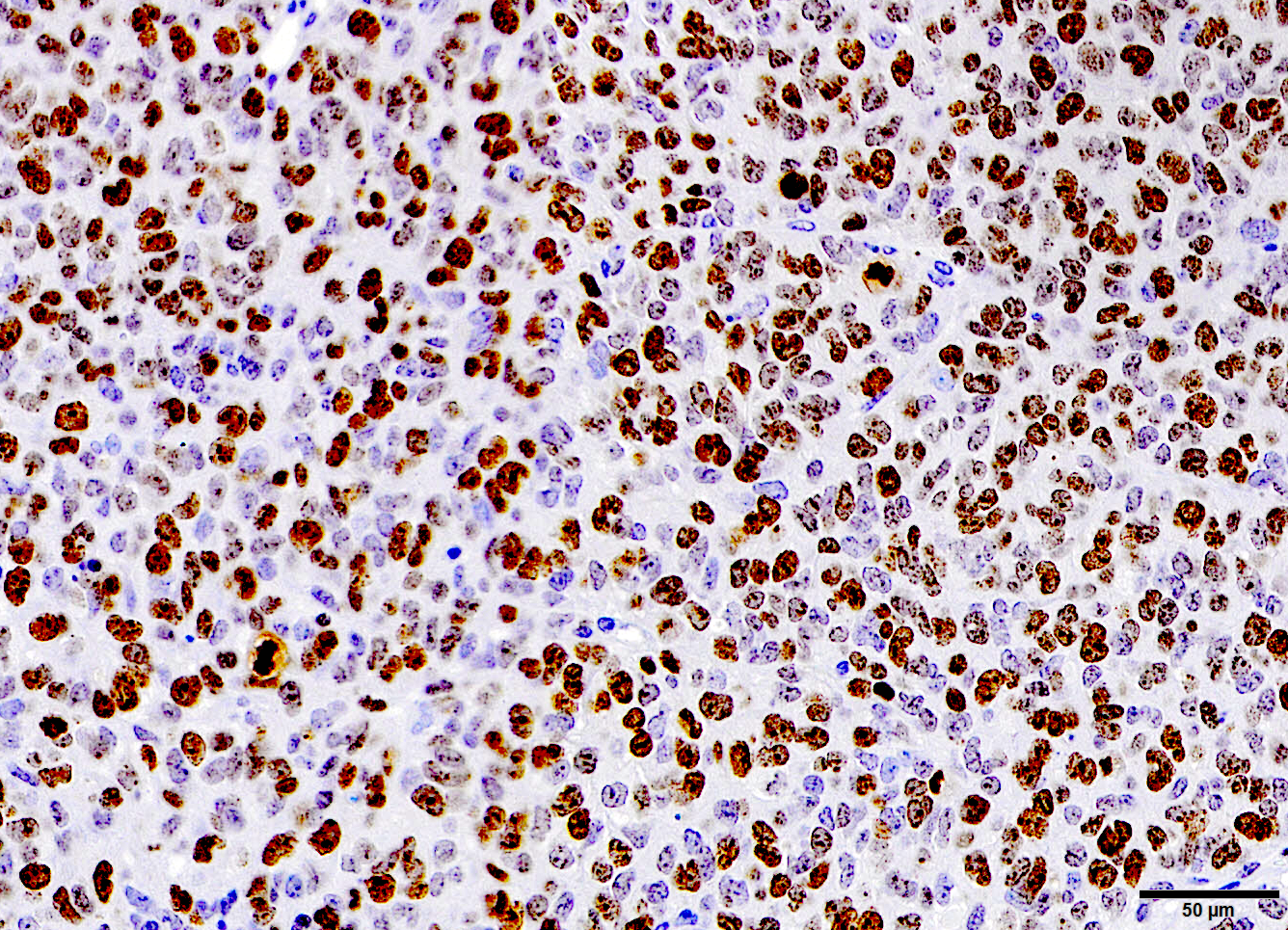

Supplement: Supplementary file 12 — Source data Fig. 4 [file 44321_2024_97_MOESM12_ESM.zip › Fig 4/Fig_4G/Ki67/Ki67 Ctl 50.png]

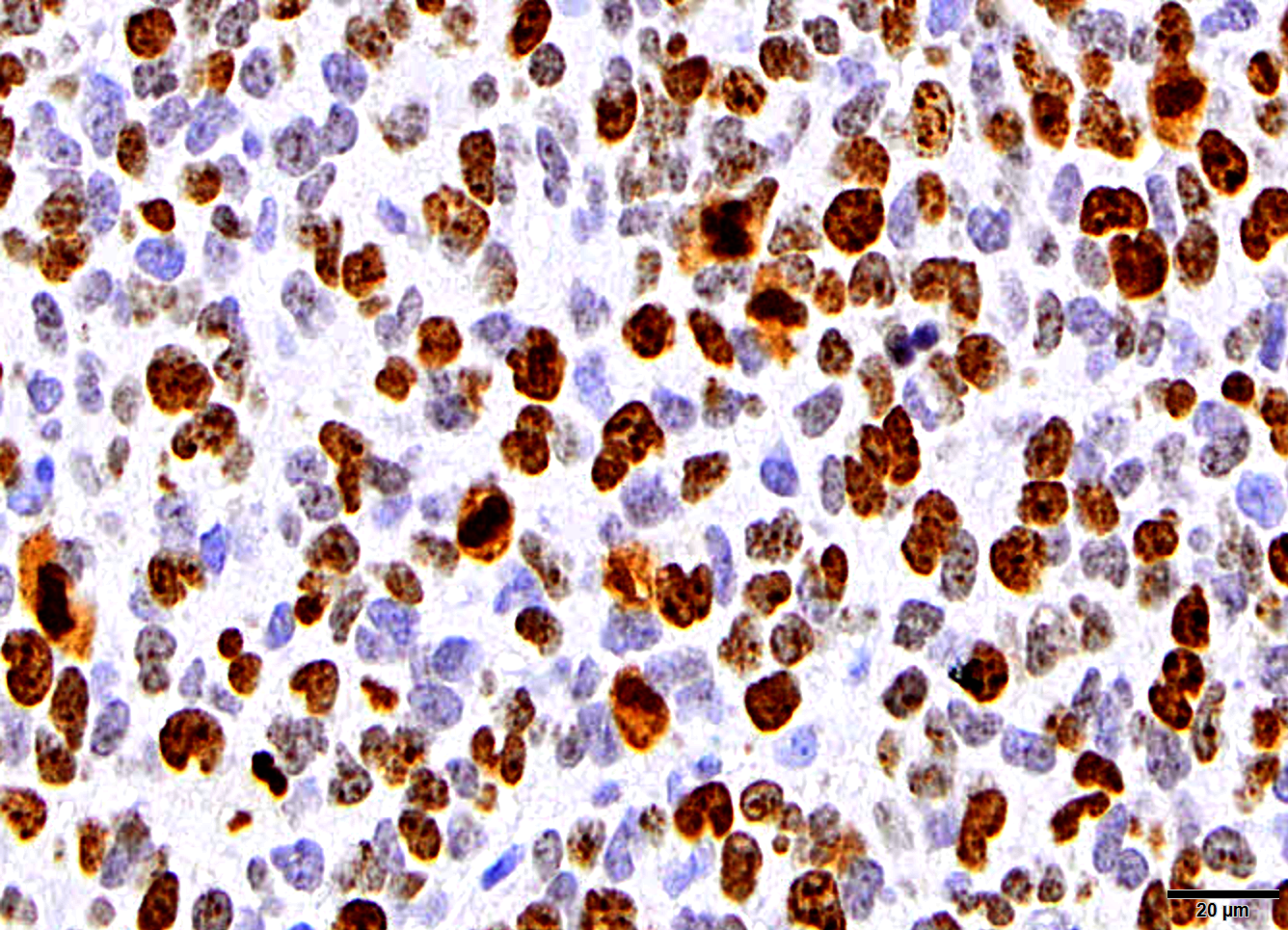

Supplement: Supplementary file 12 — Source data Fig. 4 [file 44321_2024_97_MOESM12_ESM.zip › Fig 4/Fig_4G/Ki67/Ki67 sh1 20.png]

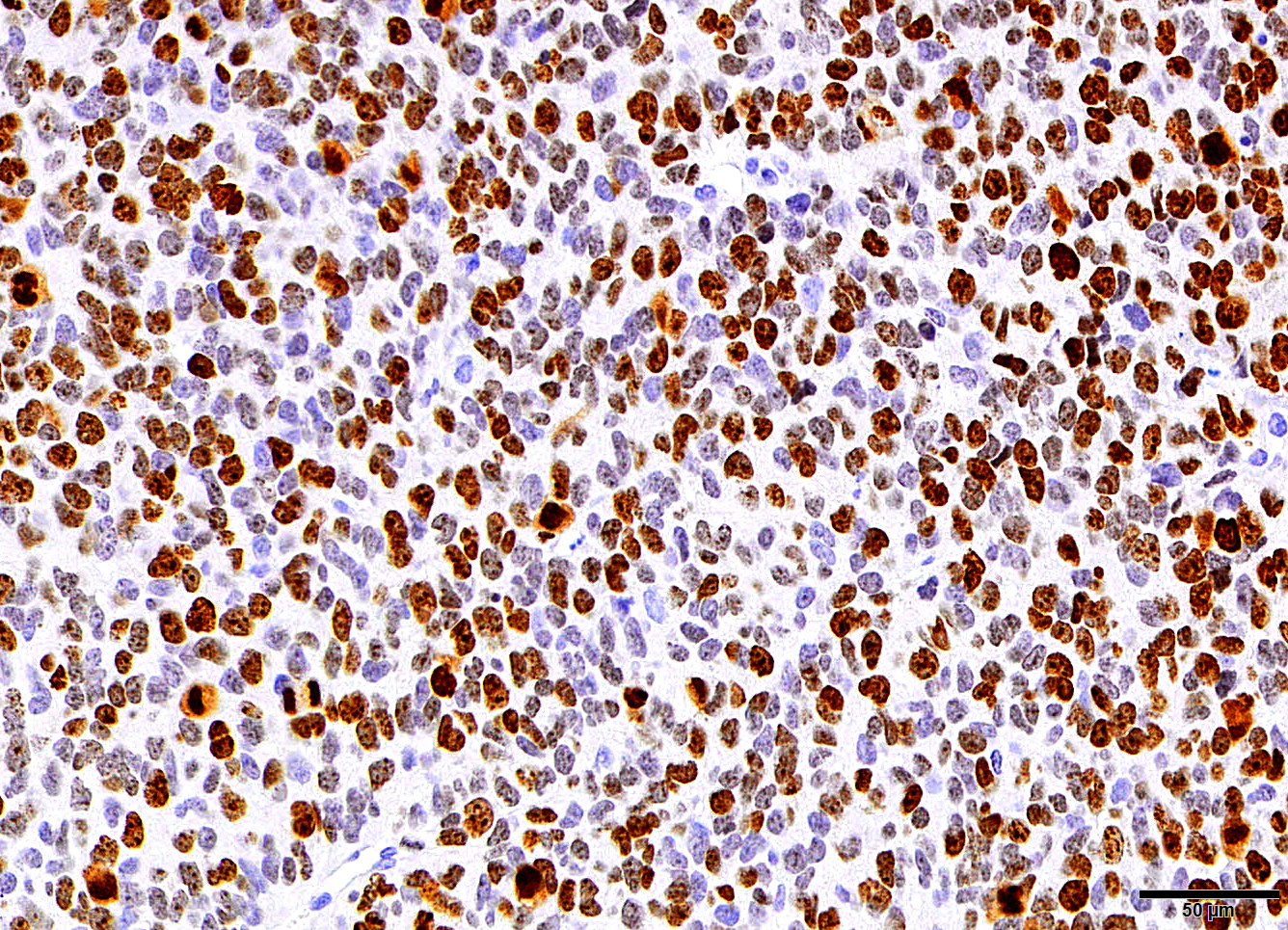

Supplement: Supplementary file 12 — Source data Fig. 4 [file 44321_2024_97_MOESM12_ESM.zip › Fig 4/Fig_4G/Ki67/Ki67 sh1 50.png]

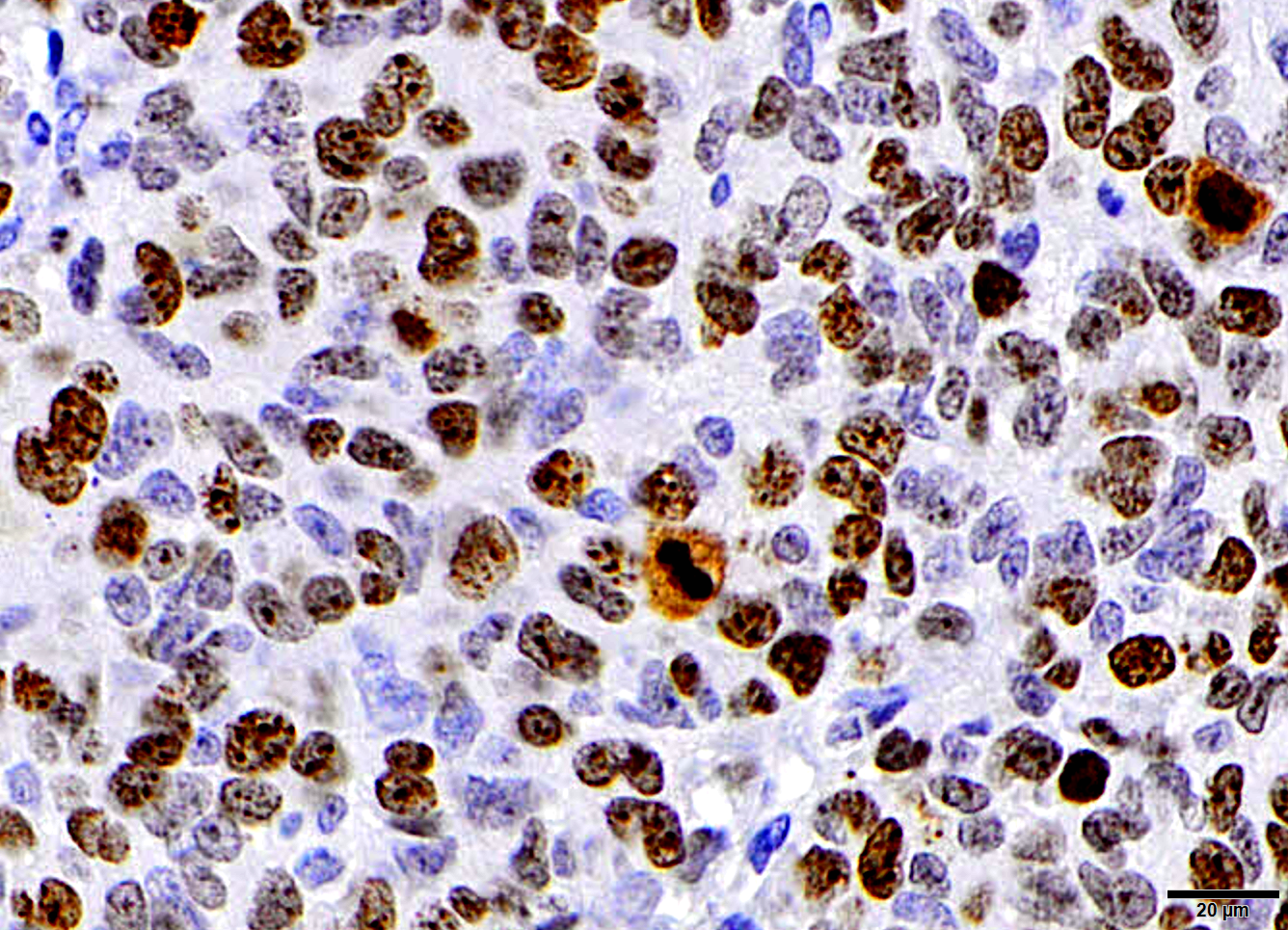

Supplement: Supplementary file 12 — Source data Fig. 4 [file 44321_2024_97_MOESM12_ESM.zip › Fig 4/Fig_4G/Ki67/Ki67 sh2 20.png]

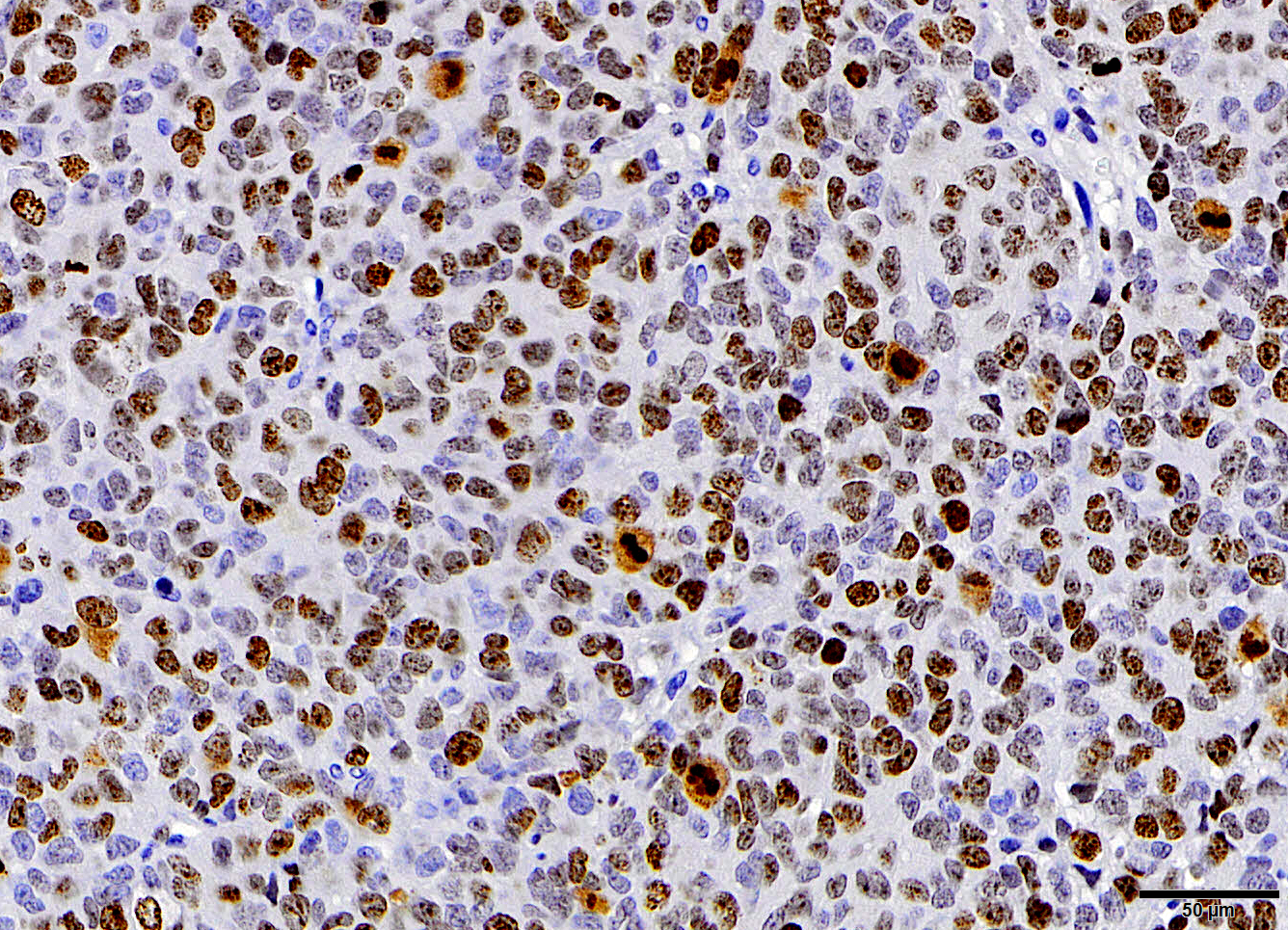

Supplement: Supplementary file 12 — Source data Fig. 4 [file 44321_2024_97_MOESM12_ESM.zip › Fig 4/Fig_4G/Ki67/Ki67 sh2 50.png]

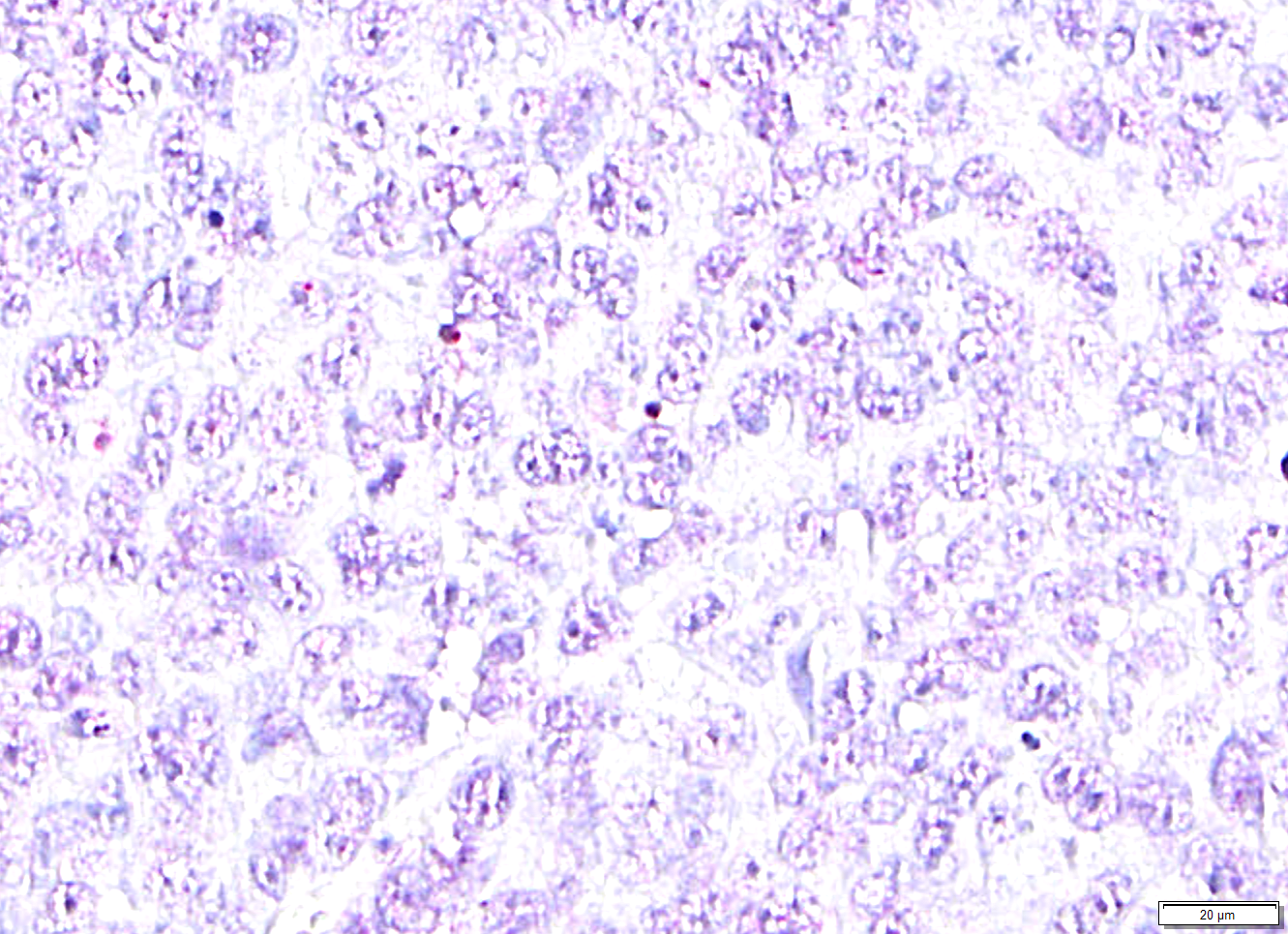

Supplement: Supplementary file 12 — Source data Fig. 4 [file 44321_2024_97_MOESM12_ESM.zip › Fig 4/Fig_4G/TUNEL/Tunel ctl 20 -6.png]

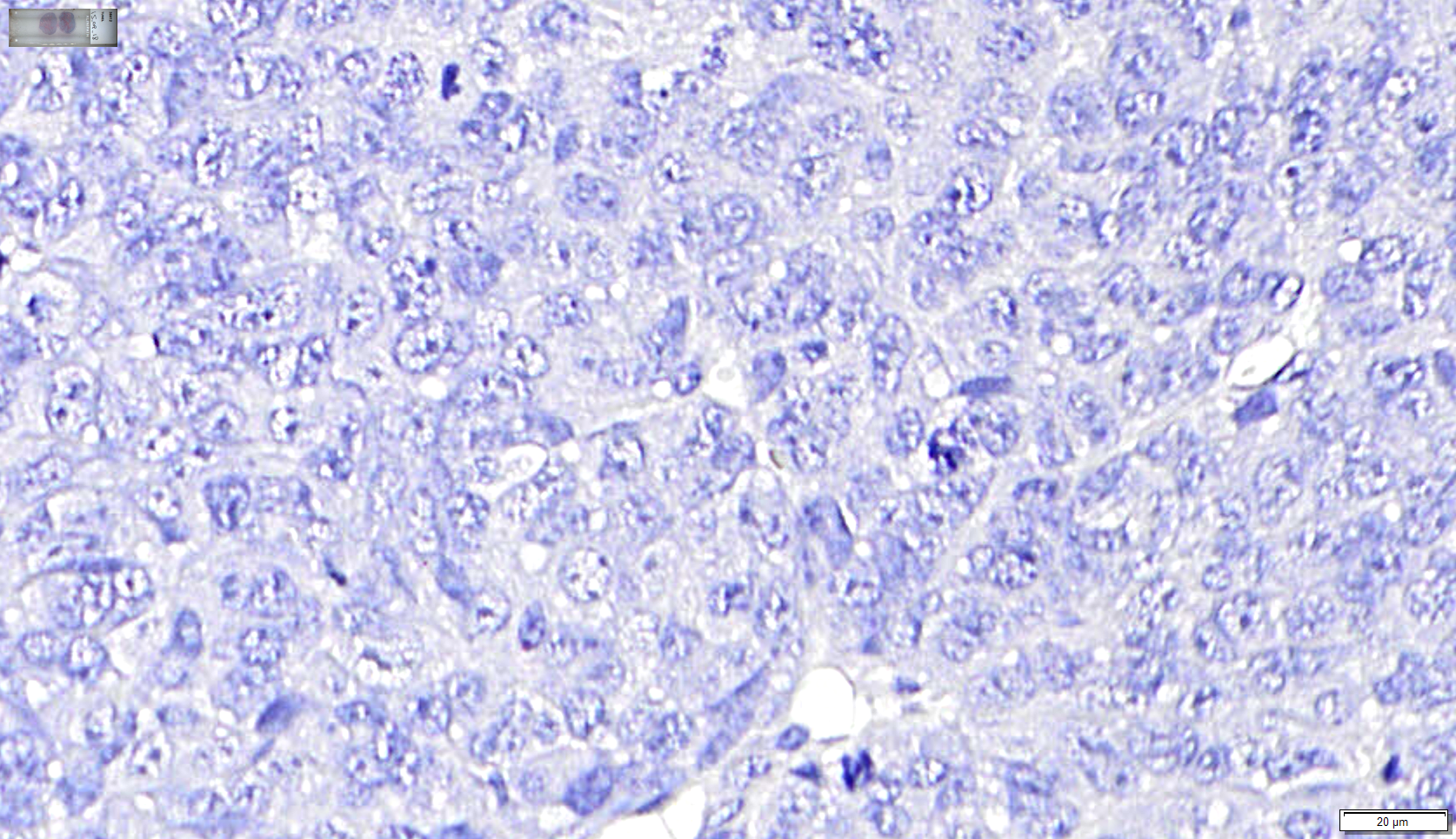

Supplement: Supplementary file 12 — Source data Fig. 4 [file 44321_2024_97_MOESM12_ESM.zip › Fig 4/Fig_4G/TUNEL/Tunel Ctl 20-1.png]

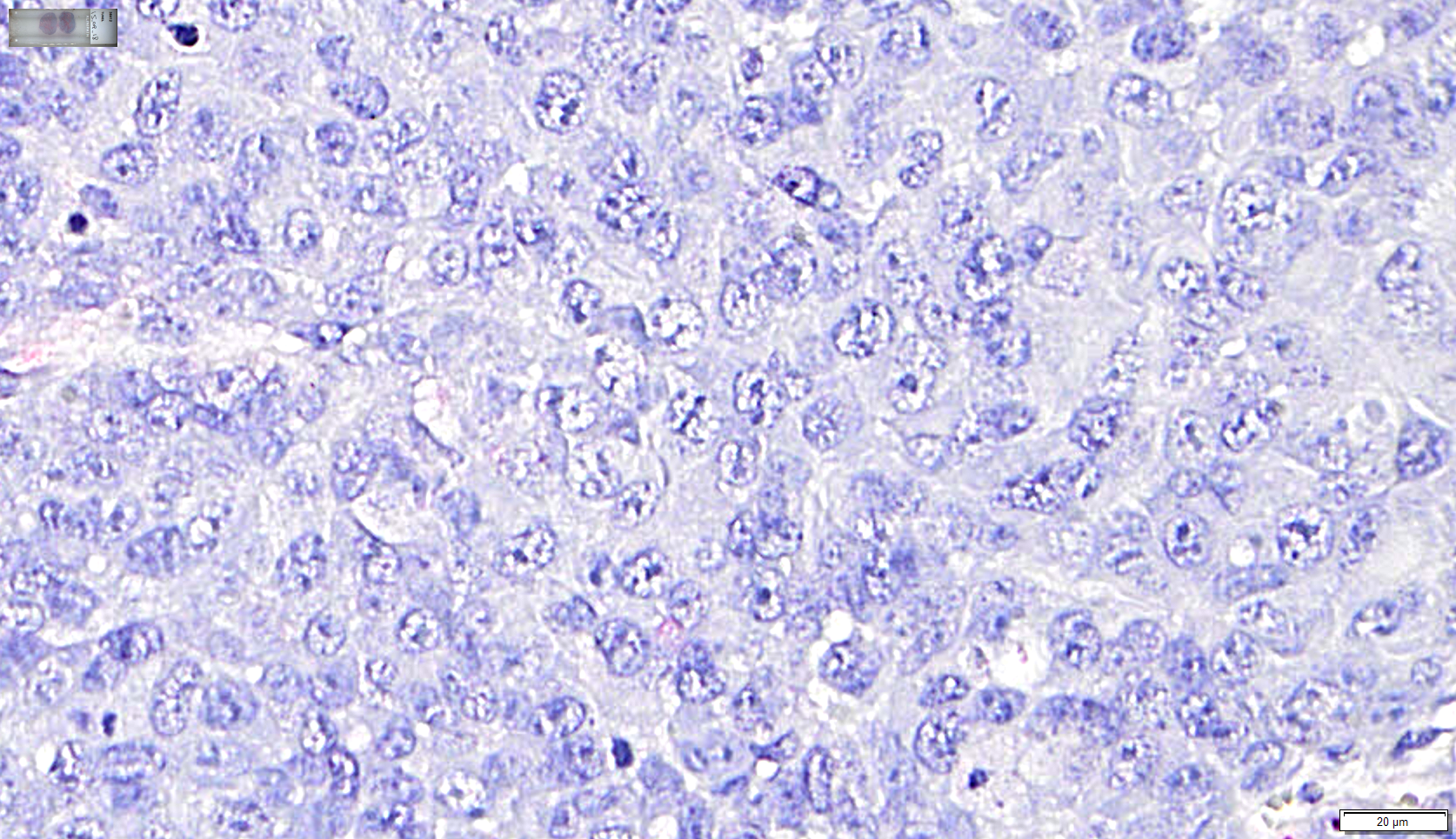

Supplement: Supplementary file 12 — Source data Fig. 4 [file 44321_2024_97_MOESM12_ESM.zip › Fig 4/Fig_4G/TUNEL/Tunel Ctl 20-3.png]

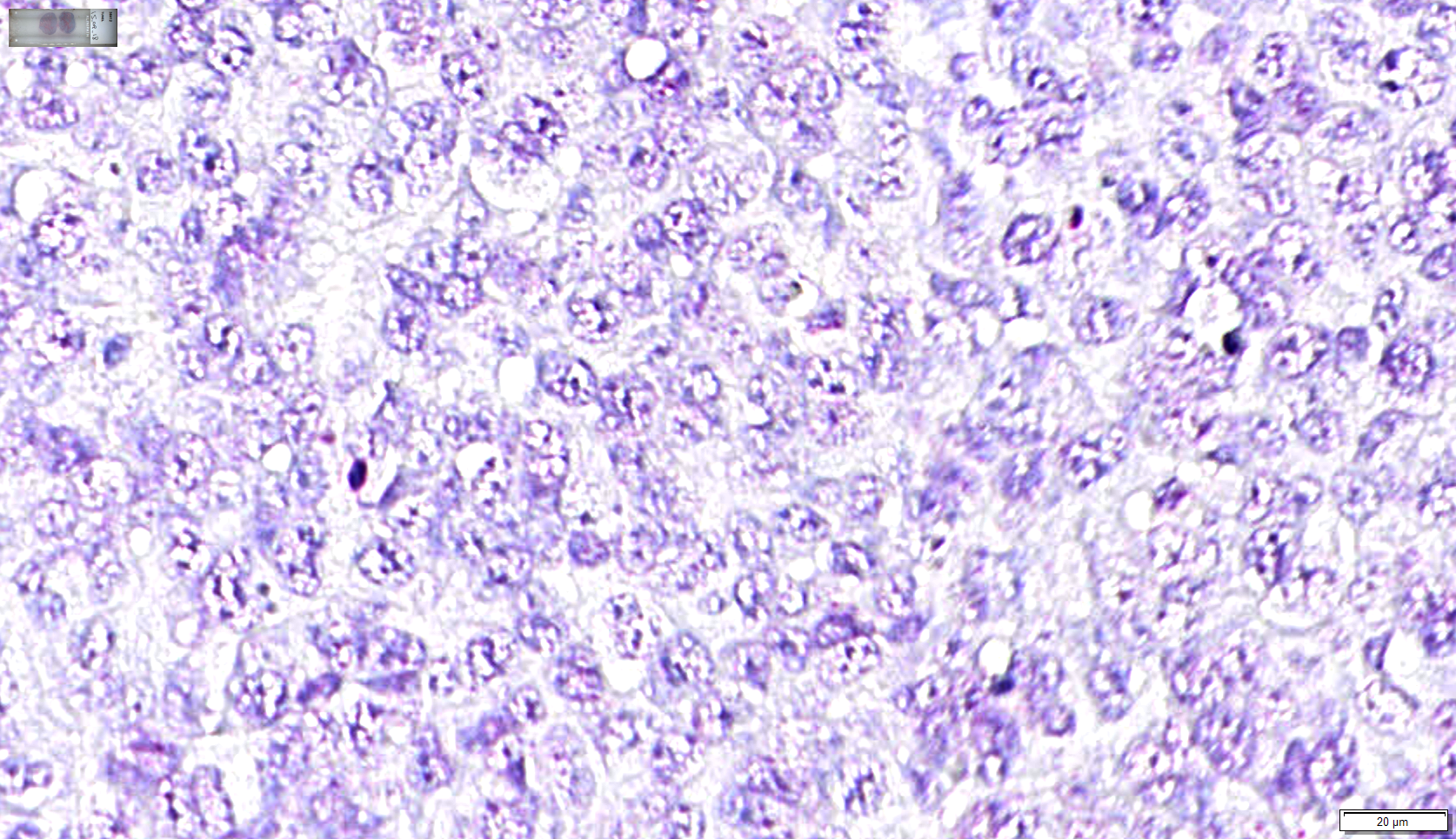

Supplement: Supplementary file 12 — Source data Fig. 4 [file 44321_2024_97_MOESM12_ESM.zip › Fig 4/Fig_4G/TUNEL/Tunel Ctl 20-4.png]

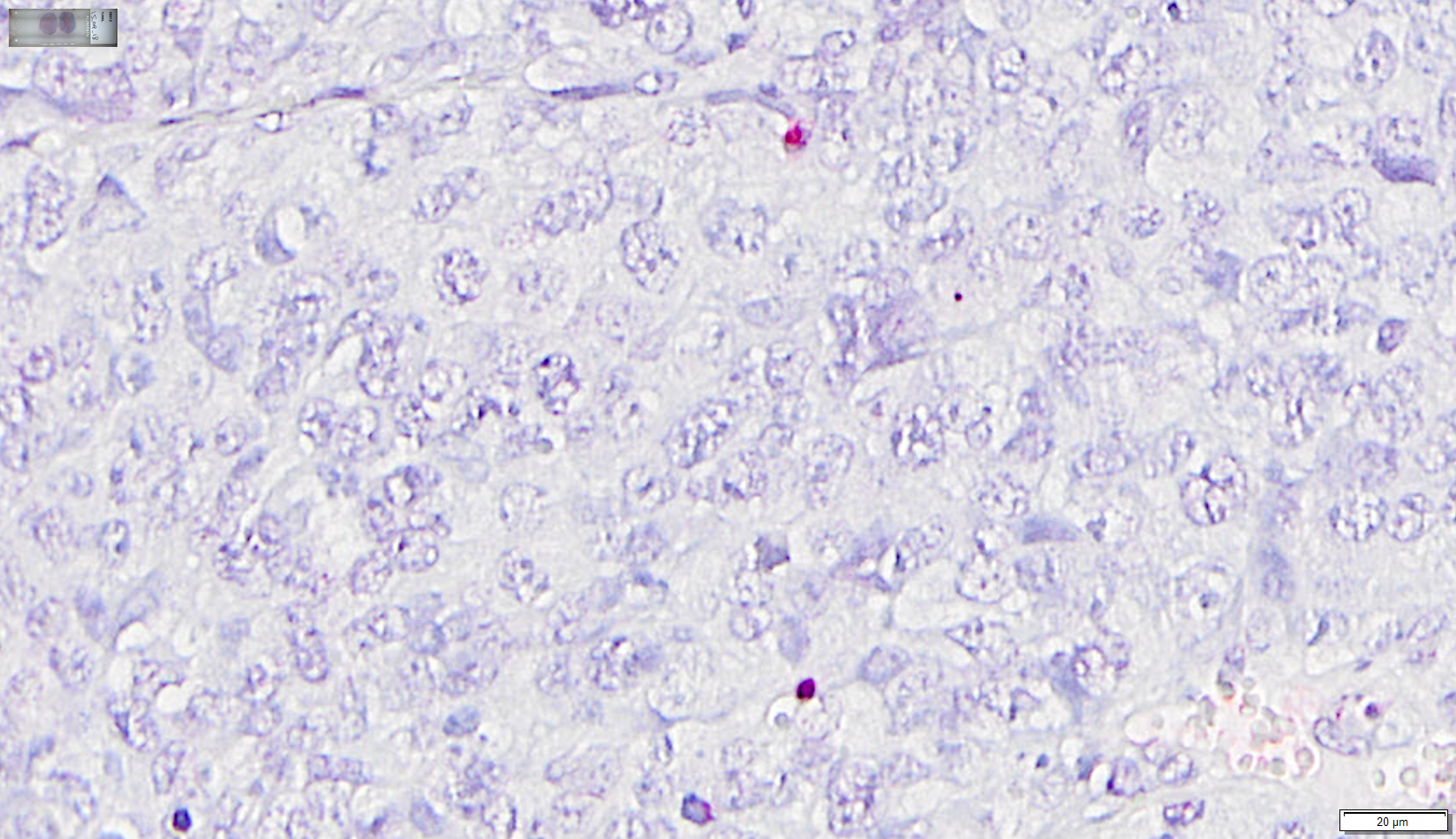

Supplement: Supplementary file 12 — Source data Fig. 4 [file 44321_2024_97_MOESM12_ESM.zip › Fig 4/Fig_4G/TUNEL/Tunel Ctl 20.png]

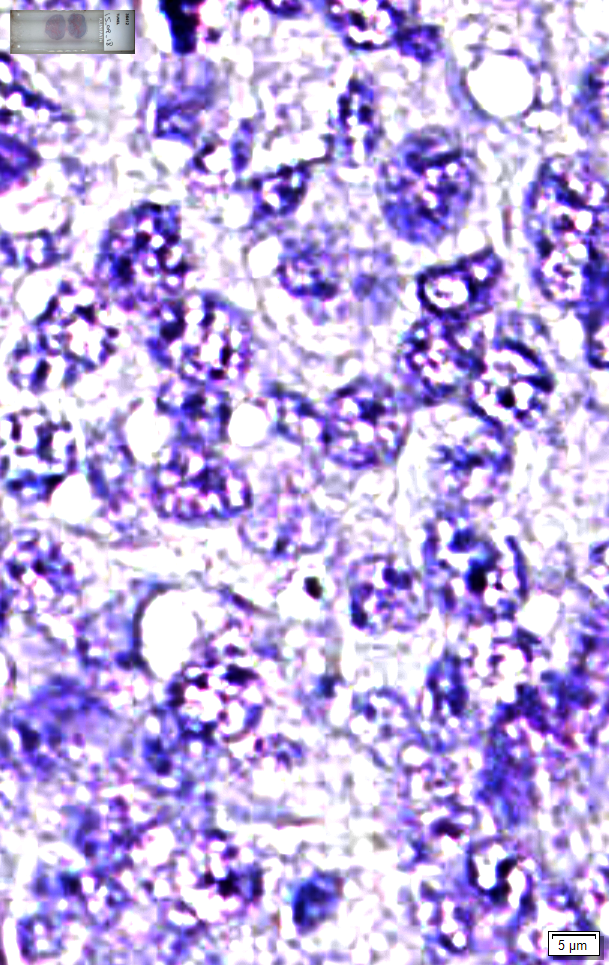

Supplement: Supplementary file 12 — Source data Fig. 4 [file 44321_2024_97_MOESM12_ESM.zip › Fig 4/Fig_4G/TUNEL/Tunel CTL 5 μm.png]

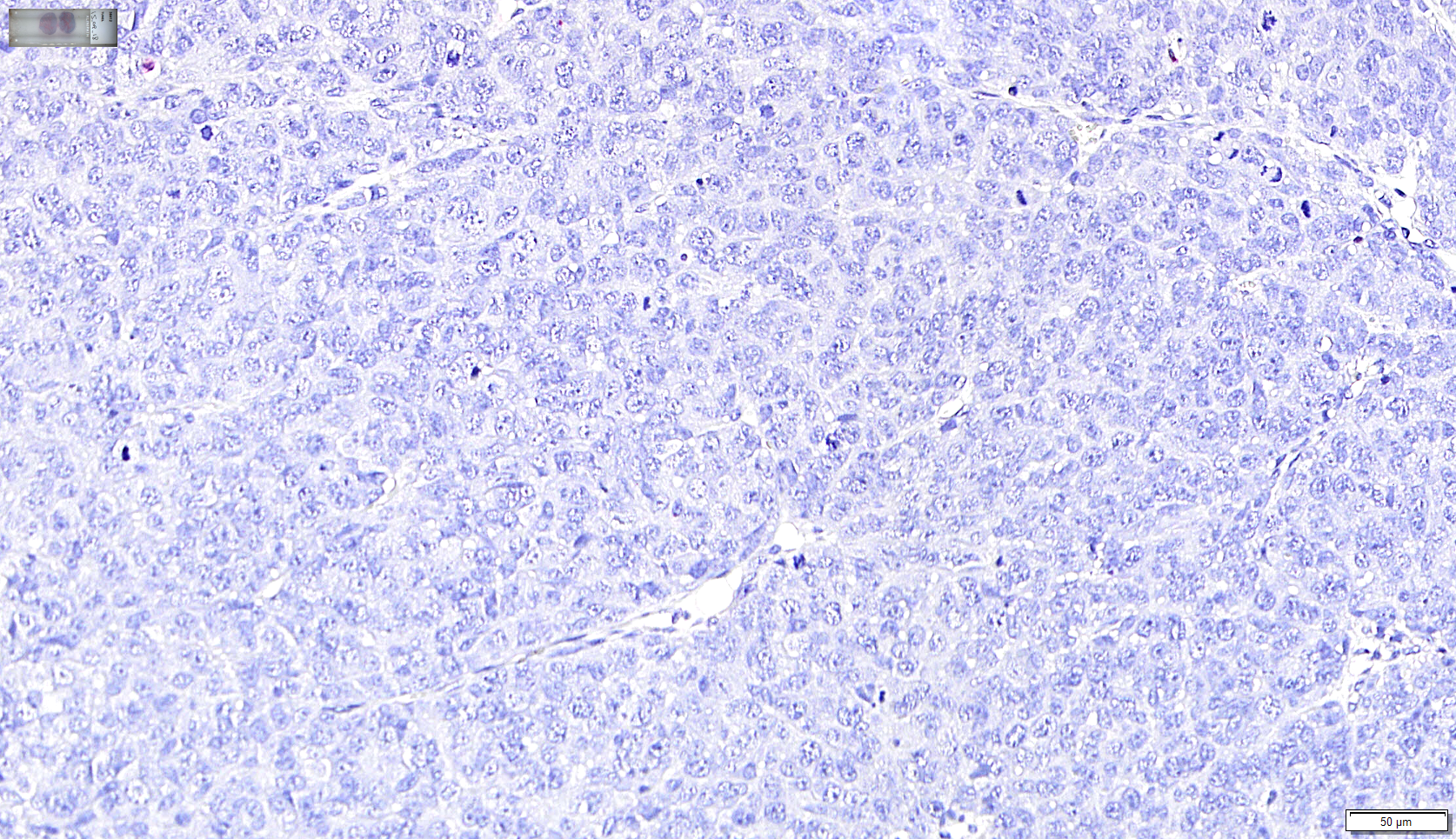

Supplement: Supplementary file 12 — Source data Fig. 4 [file 44321_2024_97_MOESM12_ESM.zip › Fig 4/Fig_4G/TUNEL/Tunel Ctl 50-1.png]

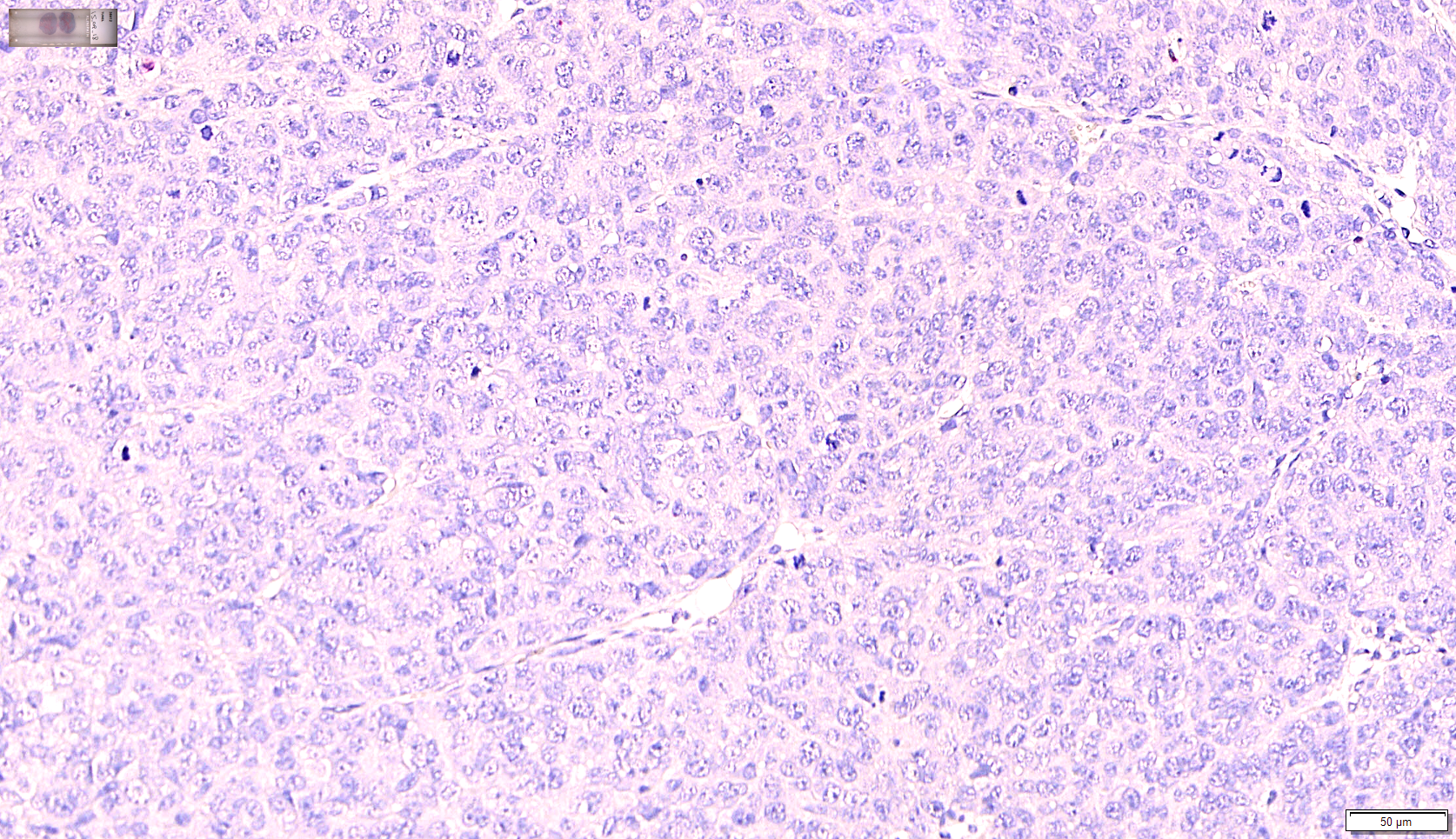

Supplement: Supplementary file 12 — Source data Fig. 4 [file 44321_2024_97_MOESM12_ESM.zip › Fig 4/Fig_4G/TUNEL/Tunel Ctl 50-2.png]

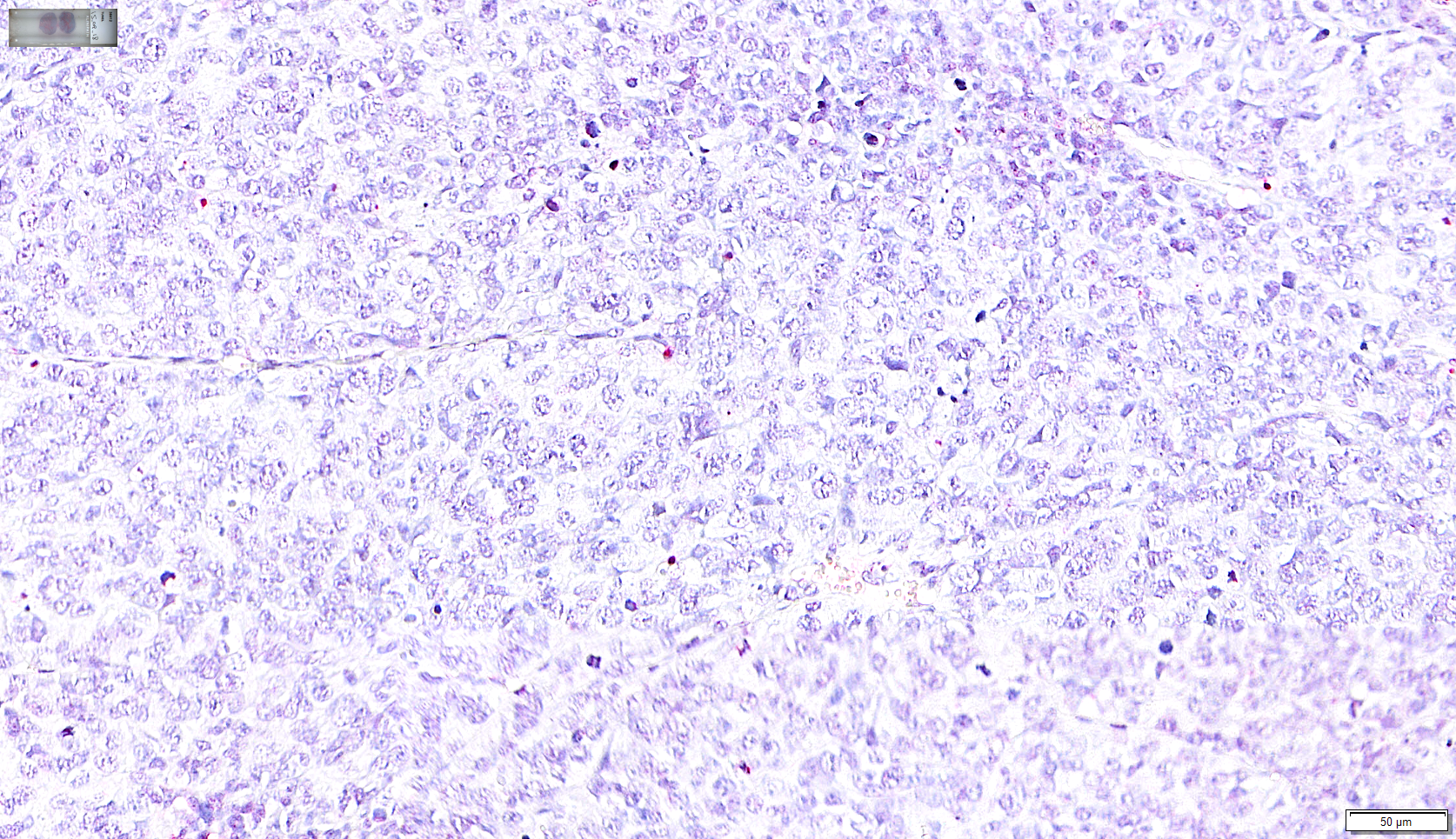

Supplement: Supplementary file 12 — Source data Fig. 4 [file 44321_2024_97_MOESM12_ESM.zip › Fig 4/Fig_4G/TUNEL/Tunel Ctl 50.png]

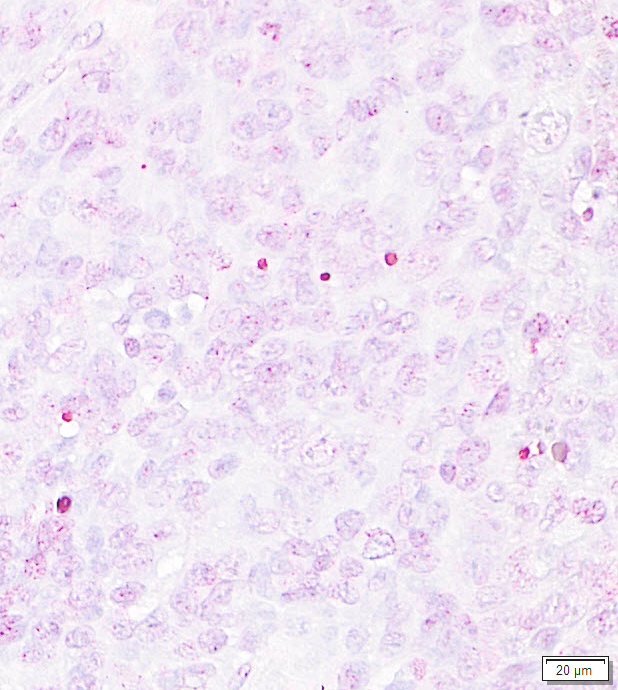

Supplement: Supplementary file 12 — Source data Fig. 4 [file 44321_2024_97_MOESM12_ESM.zip › Fig 4/Fig_4G/TUNEL/Tunel sh1 20-6.png]

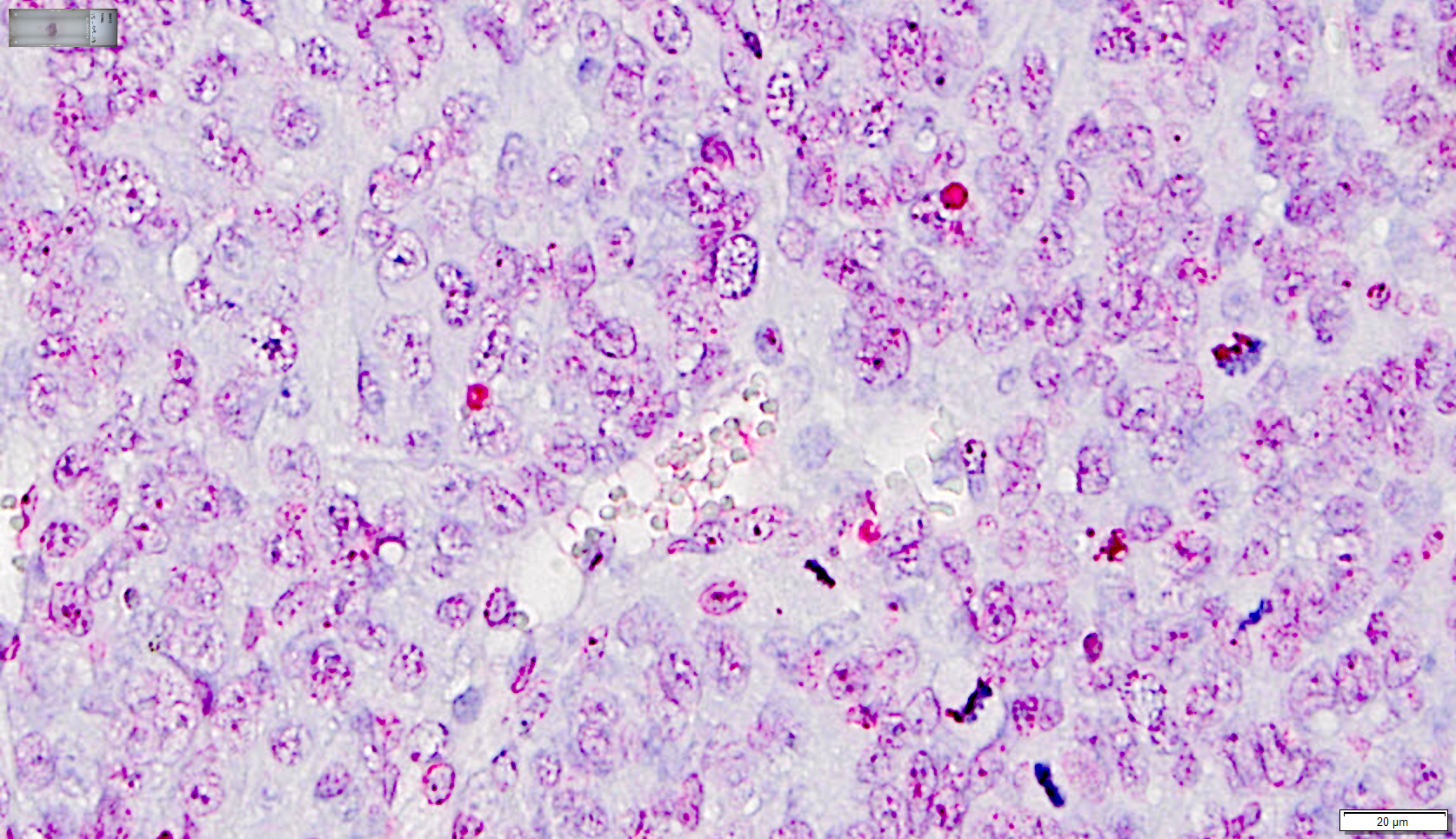

Supplement: Supplementary file 12 — Source data Fig. 4 [file 44321_2024_97_MOESM12_ESM.zip › Fig 4/Fig_4G/TUNEL/Tunel sh1-20 μm-3.png]

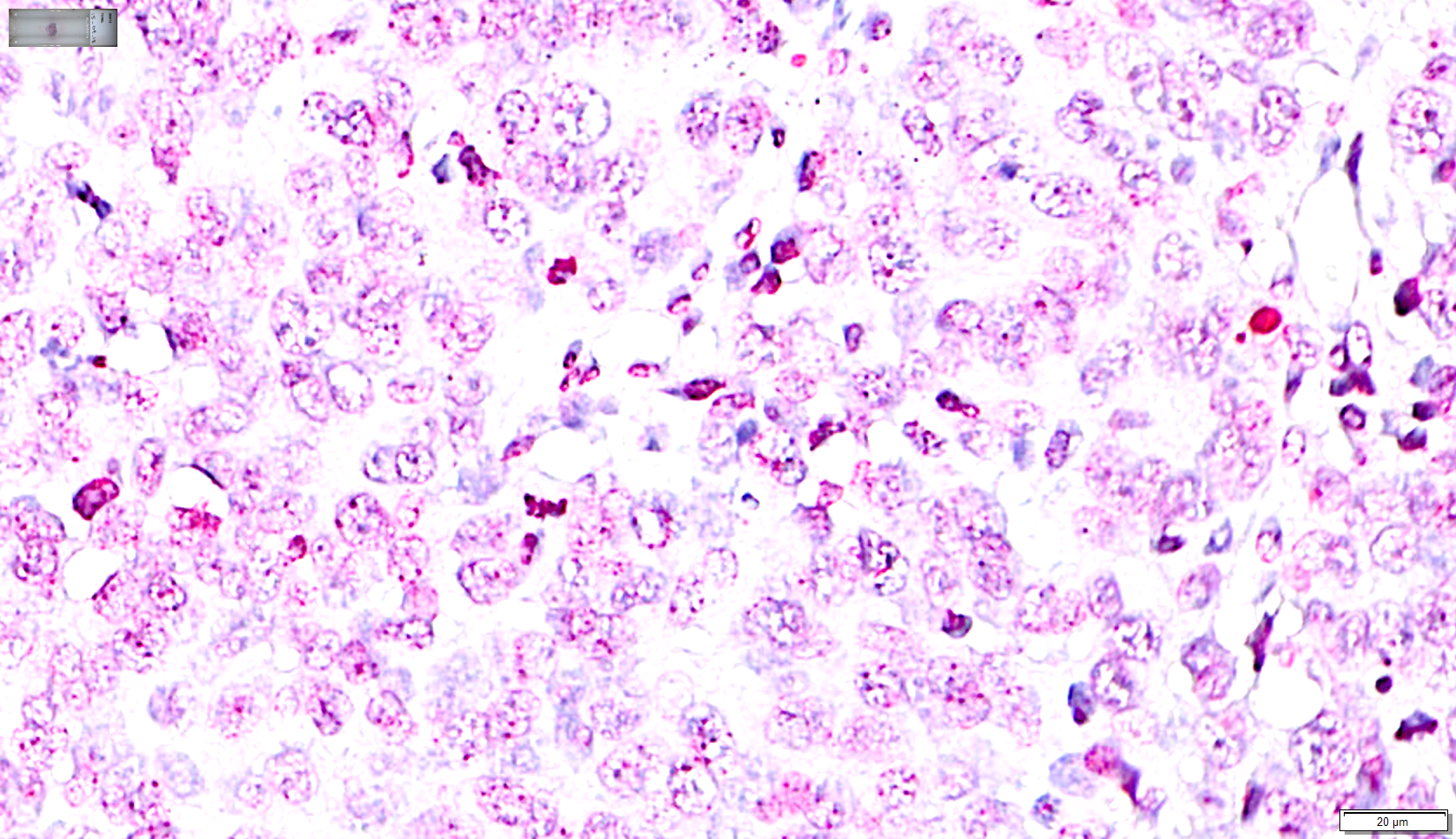

Supplement: Supplementary file 12 — Source data Fig. 4 [file 44321_2024_97_MOESM12_ESM.zip › Fig 4/Fig_4G/TUNEL/Tunel sh1-20 μm-4.png]

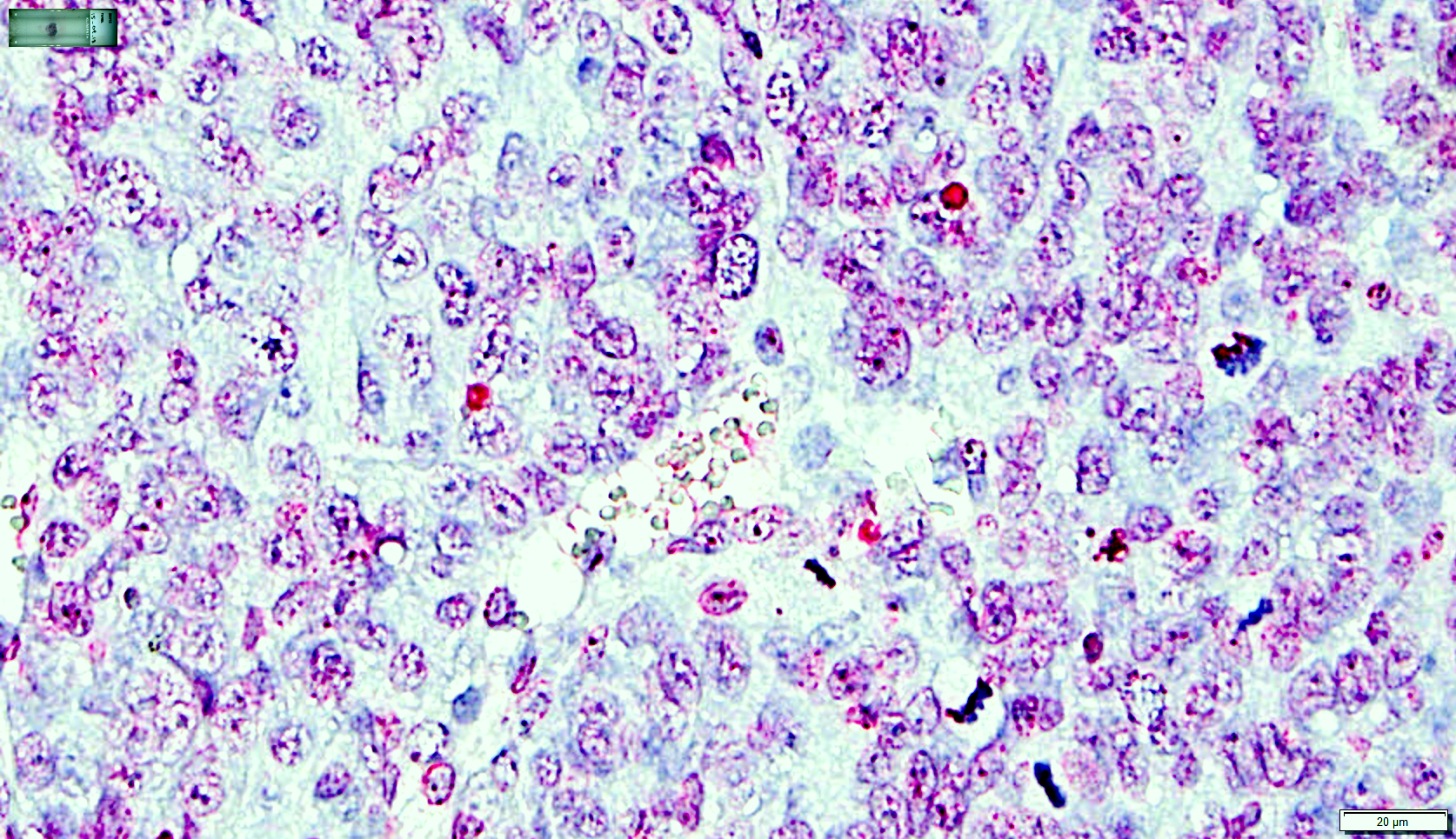

Supplement: Supplementary file 12 — Source data Fig. 4 [file 44321_2024_97_MOESM12_ESM.zip › Fig 4/Fig_4G/TUNEL/Tunel sh1-20 μm-5.png]

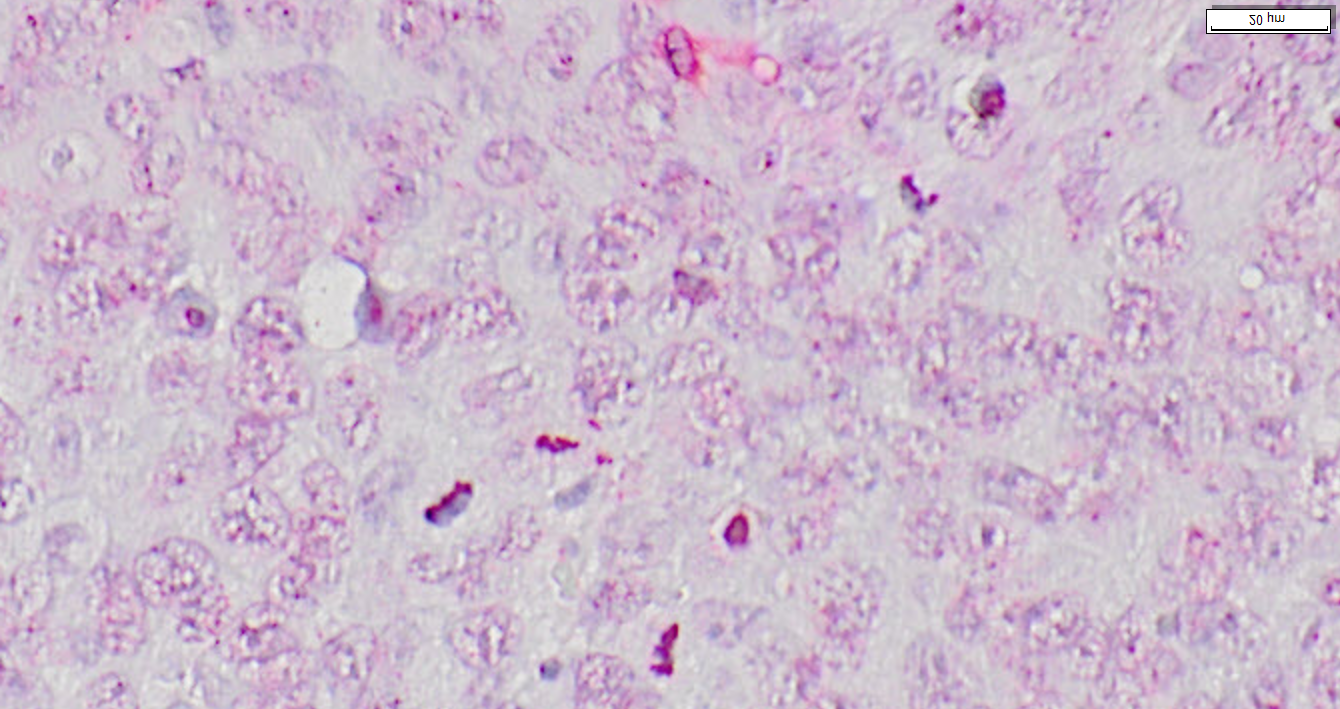

Supplement: Supplementary file 12 — Source data Fig. 4 [file 44321_2024_97_MOESM12_ESM.zip › Fig 4/Fig_4G/TUNEL/Tunel sh1-20 μm.png]

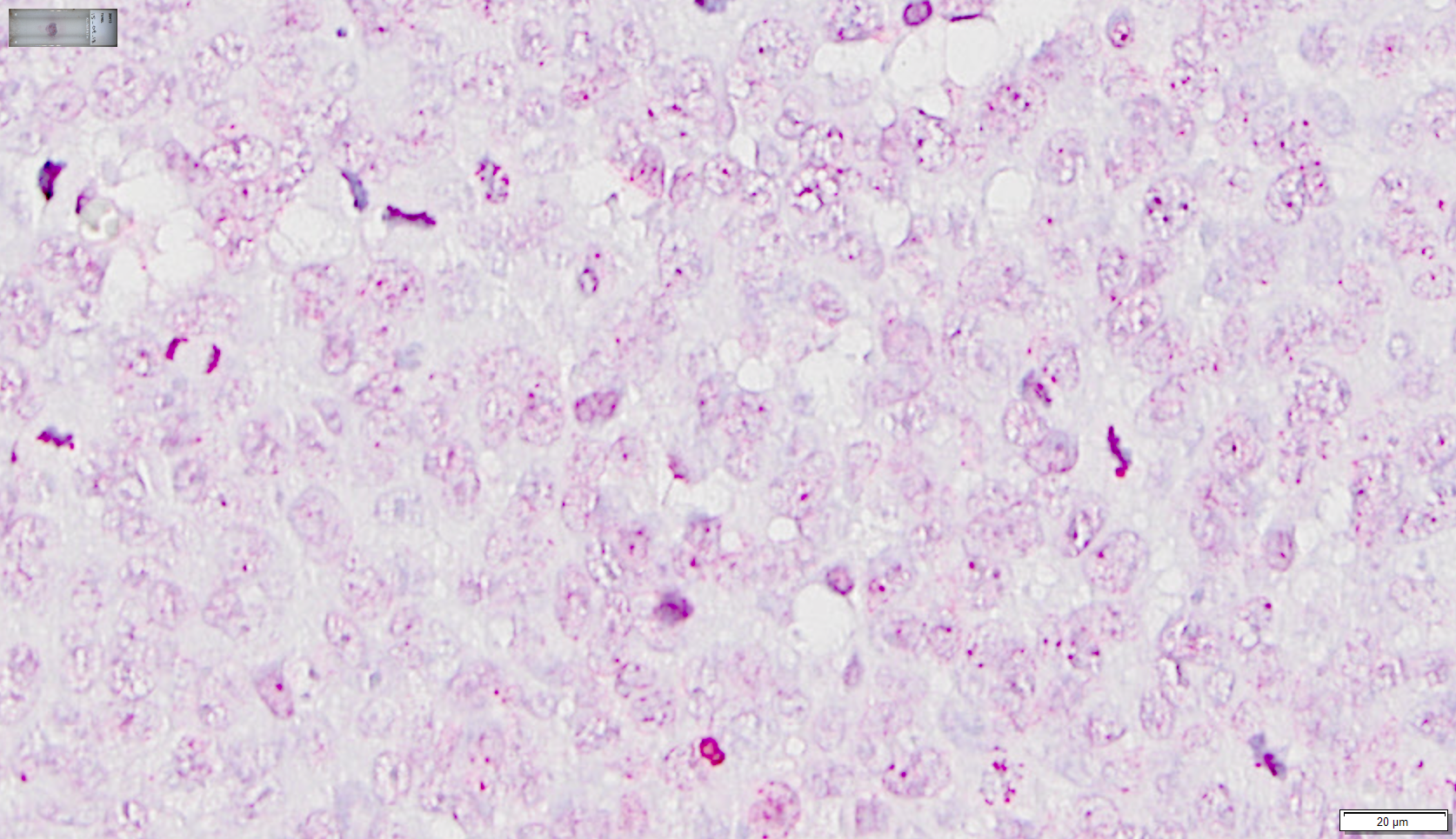

Supplement: Supplementary file 12 — Source data Fig. 4 [file 44321_2024_97_MOESM12_ESM.zip › Fig 4/Fig_4G/TUNEL/Tunel sh1-20-2 μm.png]

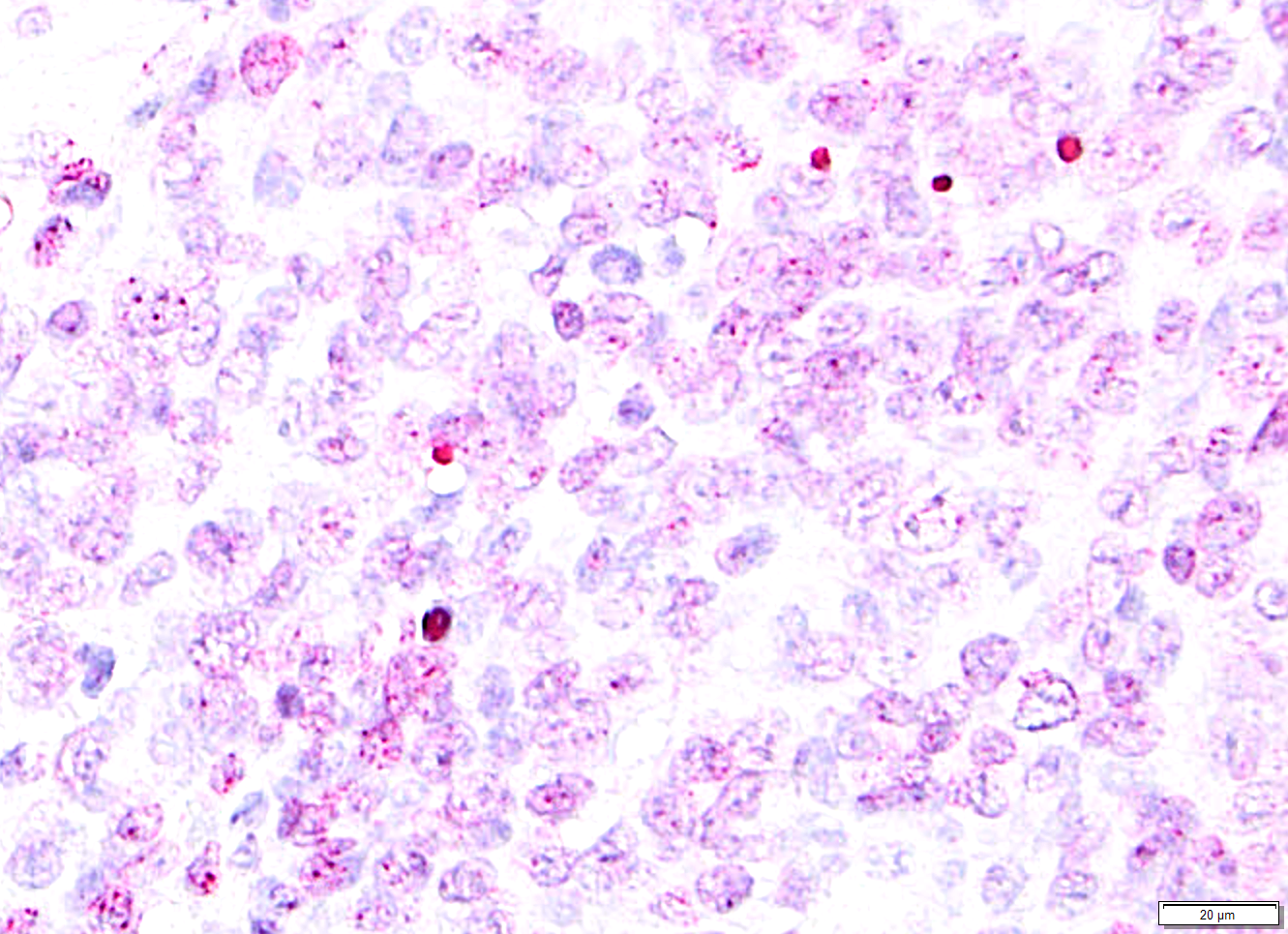

Supplement: Supplementary file 12 — Source data Fig. 4 [file 44321_2024_97_MOESM12_ESM.zip › Fig 4/Fig_4G/TUNEL/Tunel sh1-20-6.png]

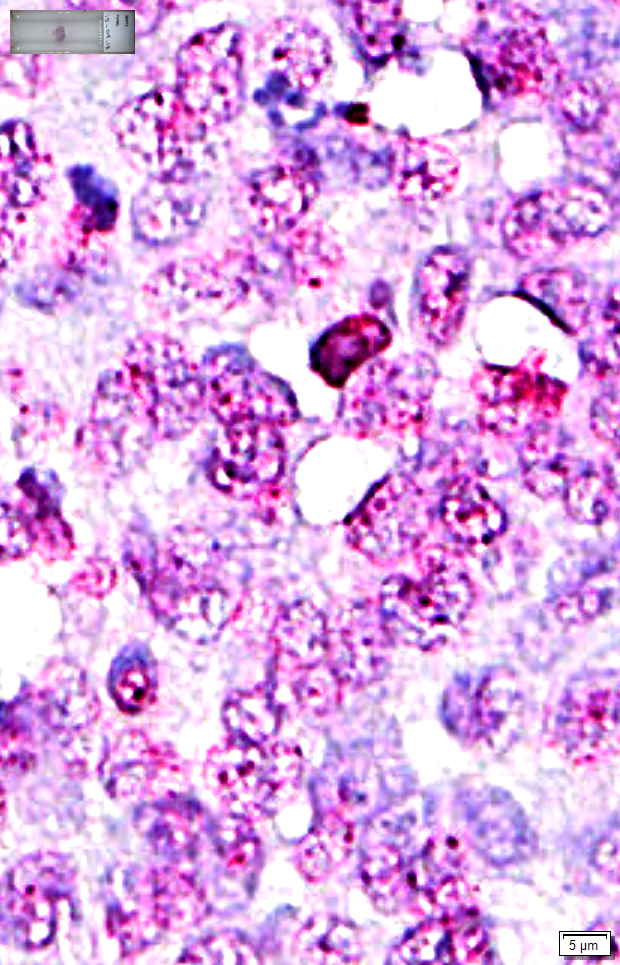

Supplement: Supplementary file 12 — Source data Fig. 4 [file 44321_2024_97_MOESM12_ESM.zip › Fig 4/Fig_4G/TUNEL/Tunel sh1-5 μm.png]
