# Supplementary material for: DiPRO1 distinctly reprograms muscle and mesenchymal cancer cells
Source: EMBO Mol Med. 2024 Jul 15;16(8):4. doi: 10.1038/s44321-024-00097-z (PMC11319797; doi:10.1038/s44321-024-00097-z)
Supplement: Supplementary file 12 — Source data Fig. 4 [file 44321_2024_97_MOESM12_ESM.zip › Fig 4/Fig_4D.pdf]

Survival analysis from Data EW Exp2_3-1700V

Generated on Mon Feb 08 09:27:37 2021

Overview

Response: **V**

Analysis type: **OS**

Parameters: **censoring on response=1800 and time=43**

Group	N	Event	MedSurv	MedFUP	Censoring
Ctl	12	12	16.00	NA	Animal26 (14+) Animal2 (15+) Animal6 (15+) Animal7 (15+) Anima23 (16+) Animal22 (16+) Animal24 (16+) Animal25 (16+) Animal3 (17+) Animal5 (20+) Animal1 (24+) Animal4 (27+)
siDiPRO1	12	11	23.50	43.00	Animal13 (15+) Animal14 (15+) Animal9 (17+) Animal30 (20+) Animal29 (22+) Animal8 (22+) Animal28 (25+) Animal10 (27+) Animal12 (27+) Animal27 (28+) Animal36 (28+) Animal11 (43-)
shDiPRO1	12	7	34.00	43.00	Animal16 (17+) Animal17 (17+) Animal18 (17+) Animal20 (17+) Animal31 (32+) Animal35 (32+) Animal15 (36+) Animal19 (43-) Animal21 (43-) Animal32 (43-) Animal33 (43-) Animal34 (43-)

Num. of animals: **36**, num. of events: **30**

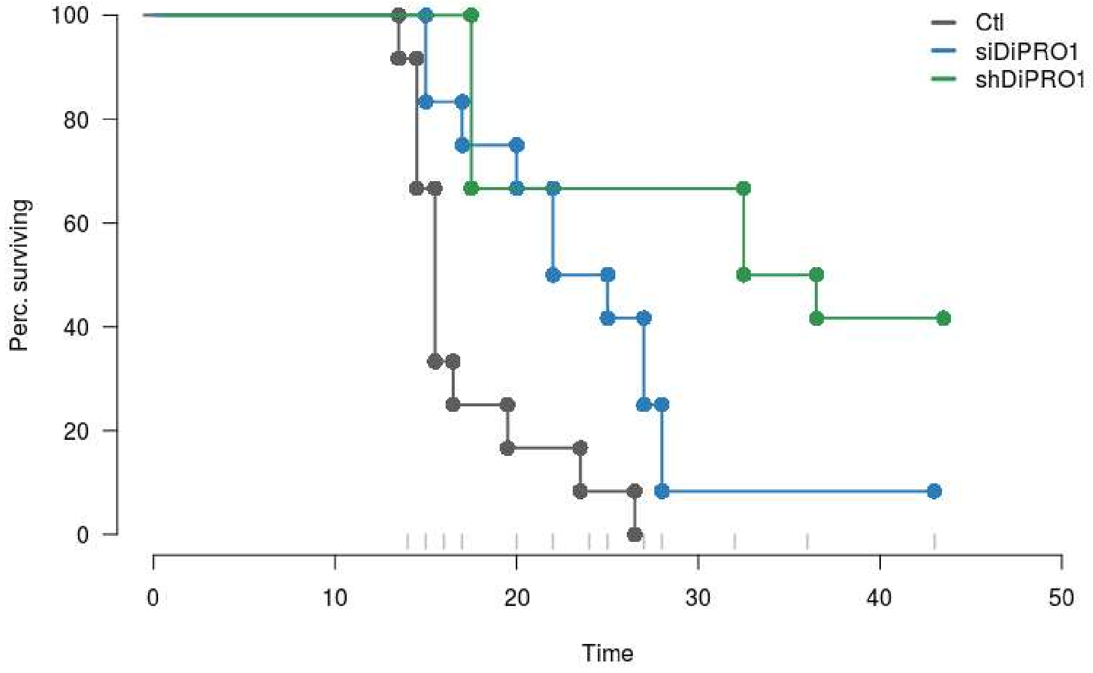

Stats

ANOVA

	Likelihood ratio test	Wald test	LogRank test
**Treat**	15.51 (d.f.=2), p<0.0004	13.86 (d.f.=2), p<0.0010	19.35 (d.f.=2), p<<0.0001

Hazard ratios

P-value adjustement: **holm**

Covariate	Hazard ratio	Pvalue	PvalueAdj	LogRPval	LogRPvalAdj
siDiPRO1	0.360 [0.151;0.861]	0.0195	0.0195	0.0085	0.0085
shDiPRO1	0.131 [0.045;0.387]	<0.0001	0.0002	0.0002	0.0003

Modelling

Cox regression

```
## coxphf(formula = Surv(Time, Event) ~ Grp, data = cdf)
## Model fitted by Penalized ML
## Confidence intervals and p-values by Profile Likelihood
##
##                  coef  se(coef) exp(coef) lower 0.95 upper 0.95    Chisq
## GrpsiDiPRO1 -1.021788 0.4447540 0.3599508 0.15098414  0.8459716  5.45897
## GrpshDiPRO1 -2.028880 0.5509642 0.1314826 0.04269593  0.3660381 15.43223
```

```
##                         p
## GrpsiDiPRO1 1.946812e-02
## GrpshDiPRO1 8.551743e-05
##
## Likelihood ratio test=15.51247 on 2 df, p=0.0004280646, n=36
```

Data

Id	Time	Resp	Event	Grp
Anima23	16	1688	TRUE	Ctl
Animal1	24	1693	TRUE	Ctl
Animal2	15	1690	TRUE	Ctl
Animal22	16	1688	TRUE	Ctl
Animal24	16	1700	TRUE	Ctl
Animal25	16	1688	TRUE	Ctl
Animal26	14	1700	TRUE	Ctl
Animal3	17	1319	TRUE	Ctl
Animal4	27	1383	TRUE	Ctl
Animal5	20	1587	TRUE	Ctl
Animal6	15	1429	TRUE	Ctl
Animal7	15	1743	TRUE	Ctl
Animal10	27	1793	TRUE	siDiPRO1
Animal11	43	18	FALSE	siDiPRO1
Animal12	27	1733	TRUE	siDiPRO1
Animal13	15	1312	TRUE	siDiPRO1
Animal14	15	1535	TRUE	siDiPRO1
Animal27	28	1701	TRUE	siDiPRO1
Animal28	25	1700	TRUE	siDiPRO1
Animal29	22	1689	TRUE	siDiPRO1
Animal30	20	1700	TRUE	siDiPRO1
Animal36	28	1701	TRUE	siDiPRO1
Animal8	22	1341	TRUE	siDiPRO1
Animal9	17	1343	TRUE	siDiPRO1
Animal15	36	1310	TRUE	shDiPRO1
Animal16	17	1538	TRUE	shDiPRO1
Animal17	17	1736	TRUE	shDiPRO1
Animal18	17	1717	TRUE	shDiPRO1
Animal19	43	1279	FALSE	shDiPRO1

Animal20	17	1270	TRUE	shDiPRO1
Animal21	43	74	FALSE	shDiPRO1
Animal31	32	1622	TRUE	shDiPRO1
Animal32	43	510.1	FALSE	shDiPRO1
Animal33	43	493	FALSE	shDiPRO1
Animal34	43	105.3	FALSE	shDiPRO1
Animal35	32	228.5	TRUE	shDiPRO1
