# Supplementary figures and images for: DiPRO1 distinctly reprograms muscle and mesenchymal cancer cells (part 2 of 2)
Source: EMBO Mol Med. 2024 Jul 15;16(8):4. doi: 10.1038/s44321-024-00097-z (PMC11319797; doi:10.1038/s44321-024-00097-z)

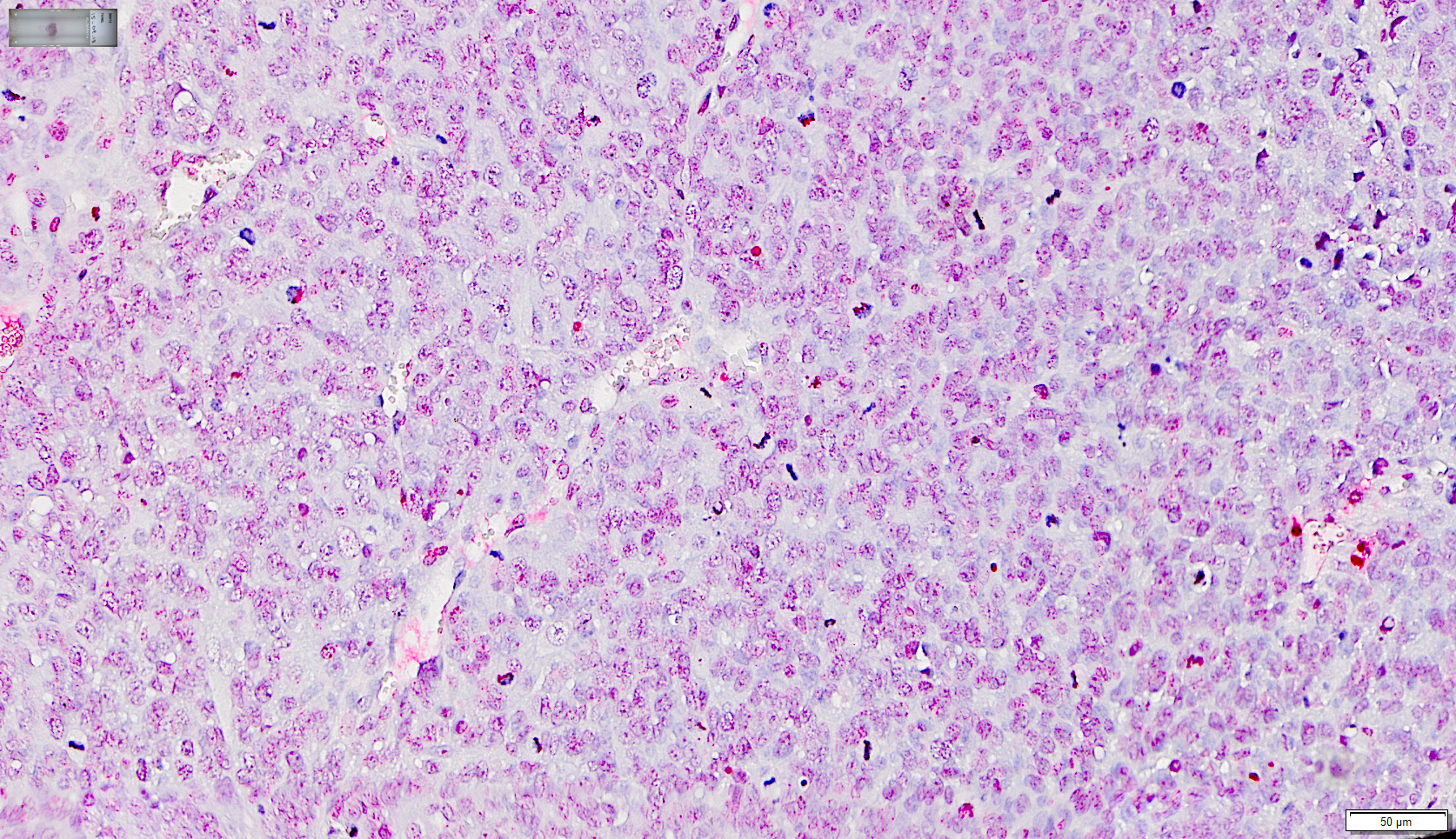

Supplement: Supplementary file 12 — Source data Fig. 4 [file 44321_2024_97_MOESM12_ESM.zip › Fig 4/Fig_4G/TUNEL/Tunel sh1-50-1.png]

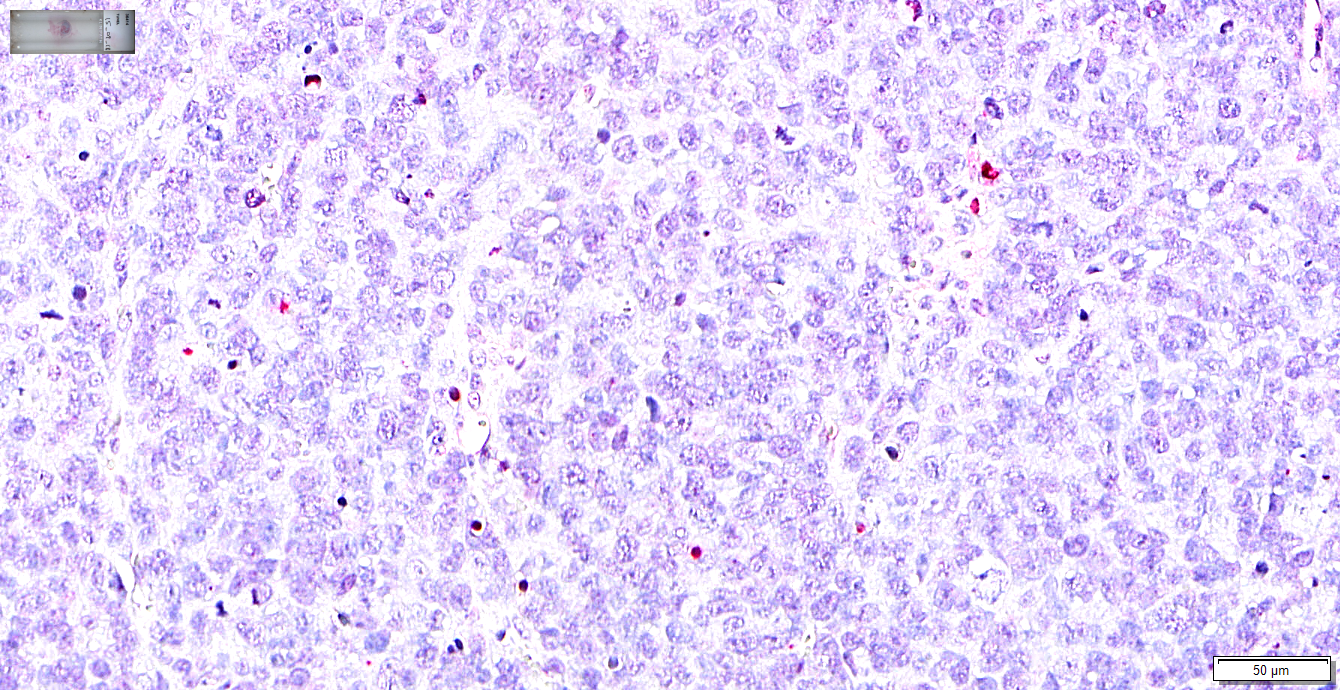

Supplement: Supplementary file 12 — Source data Fig. 4 [file 44321_2024_97_MOESM12_ESM.zip › Fig 4/Fig_4G/TUNEL/Tunel sh1-50-3.png]

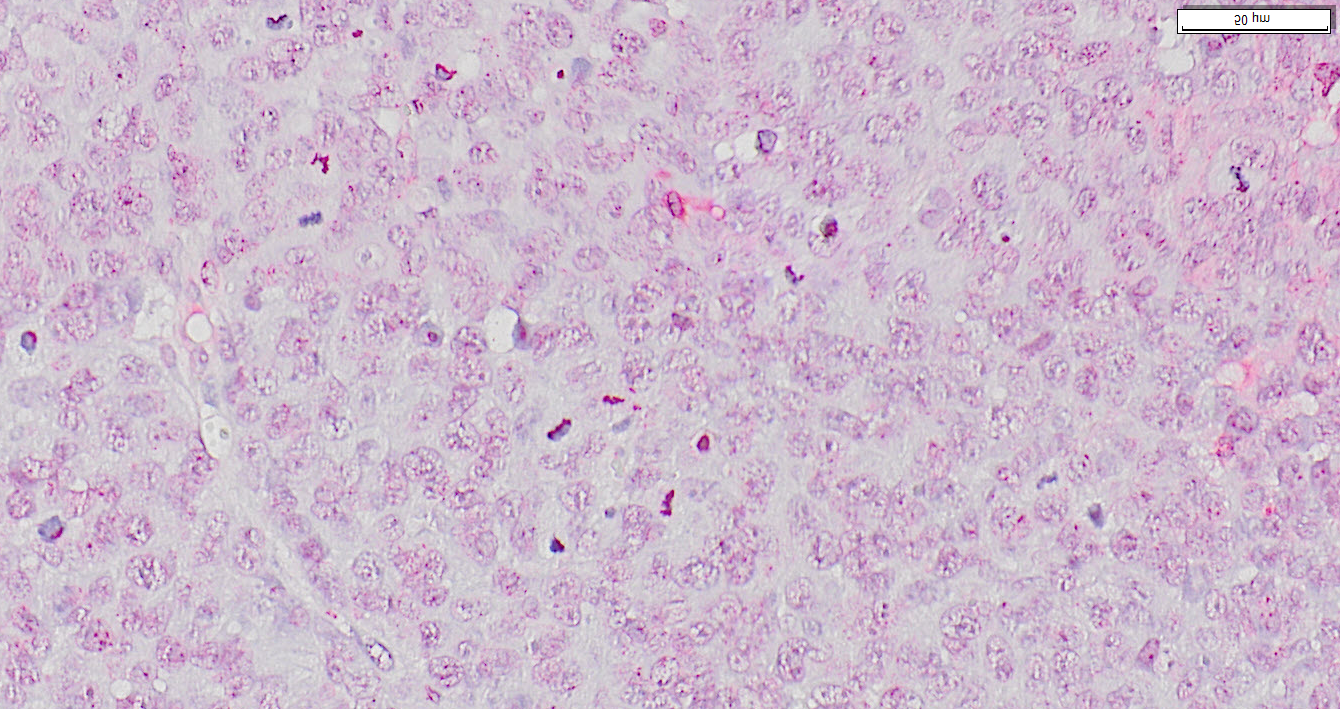

Supplement: Supplementary file 12 — Source data Fig. 4 [file 44321_2024_97_MOESM12_ESM.zip › Fig 4/Fig_4G/TUNEL/Tunel sh1.png]

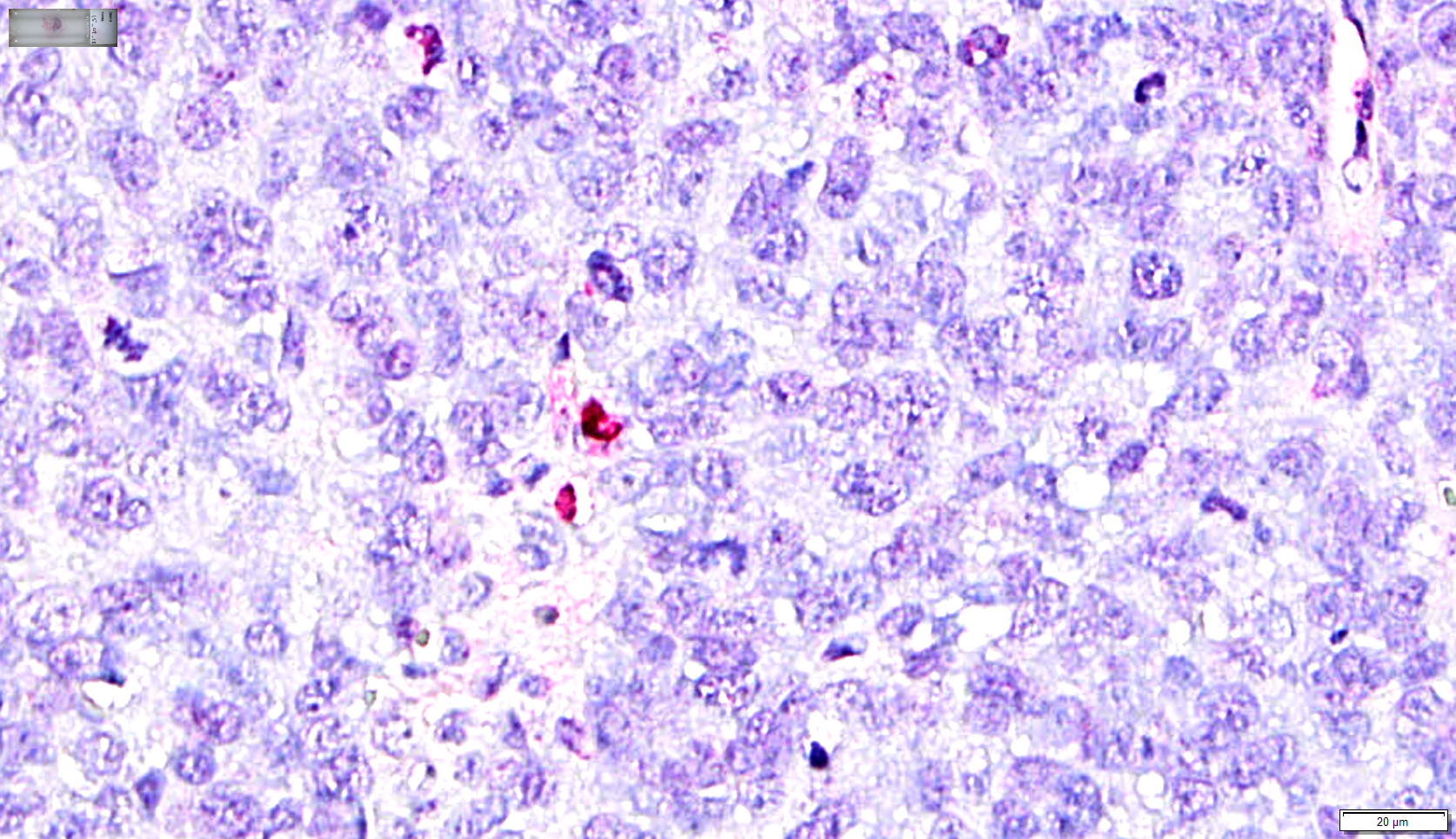

Supplement: Supplementary file 12 — Source data Fig. 4 [file 44321_2024_97_MOESM12_ESM.zip › Fig 4/Fig_4G/TUNEL/Tunel sh2 20-2.png]

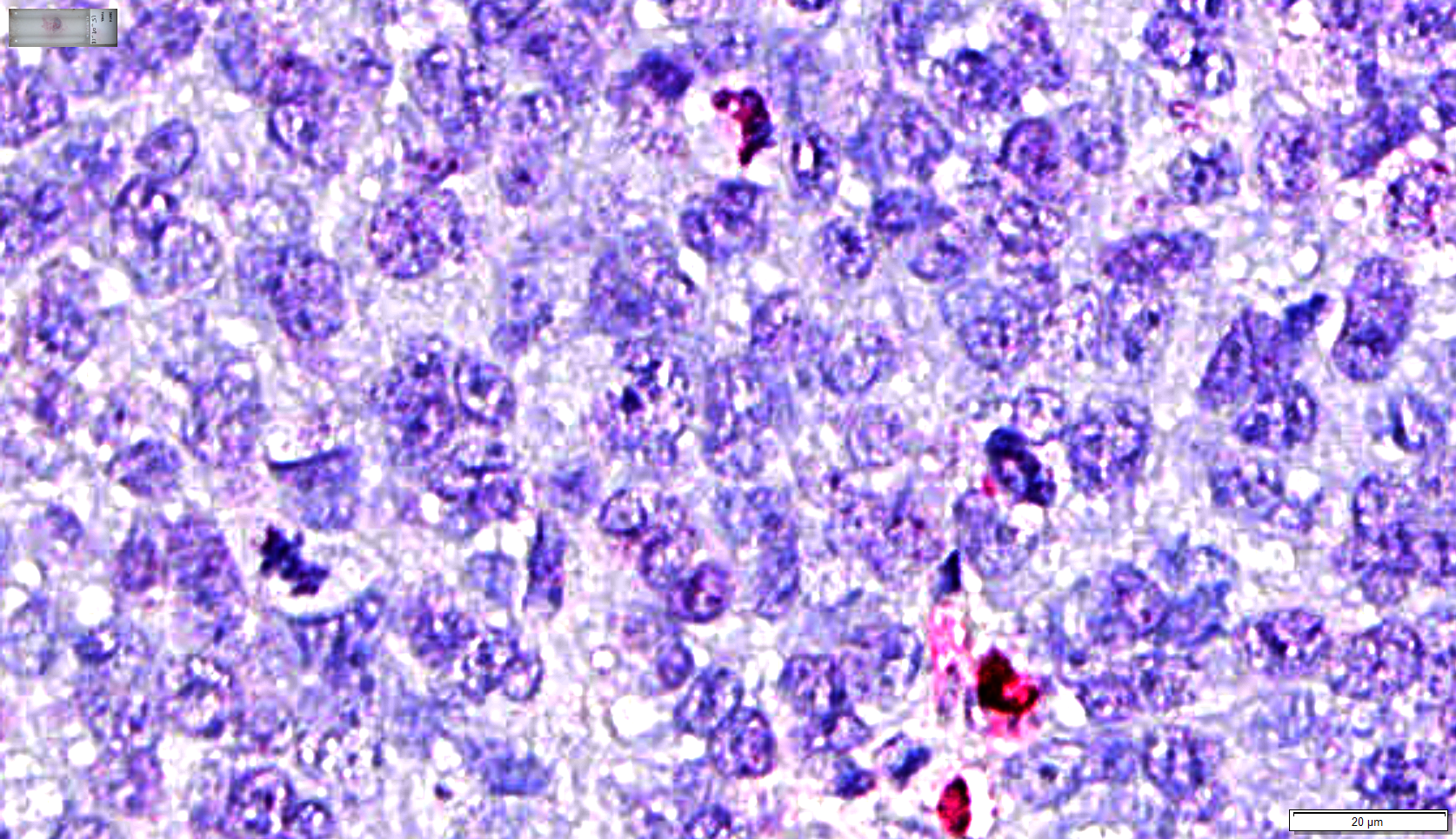

Supplement: Supplementary file 12 — Source data Fig. 4 [file 44321_2024_97_MOESM12_ESM.zip › Fig 4/Fig_4G/TUNEL/Tunel sh2 20-5.png]

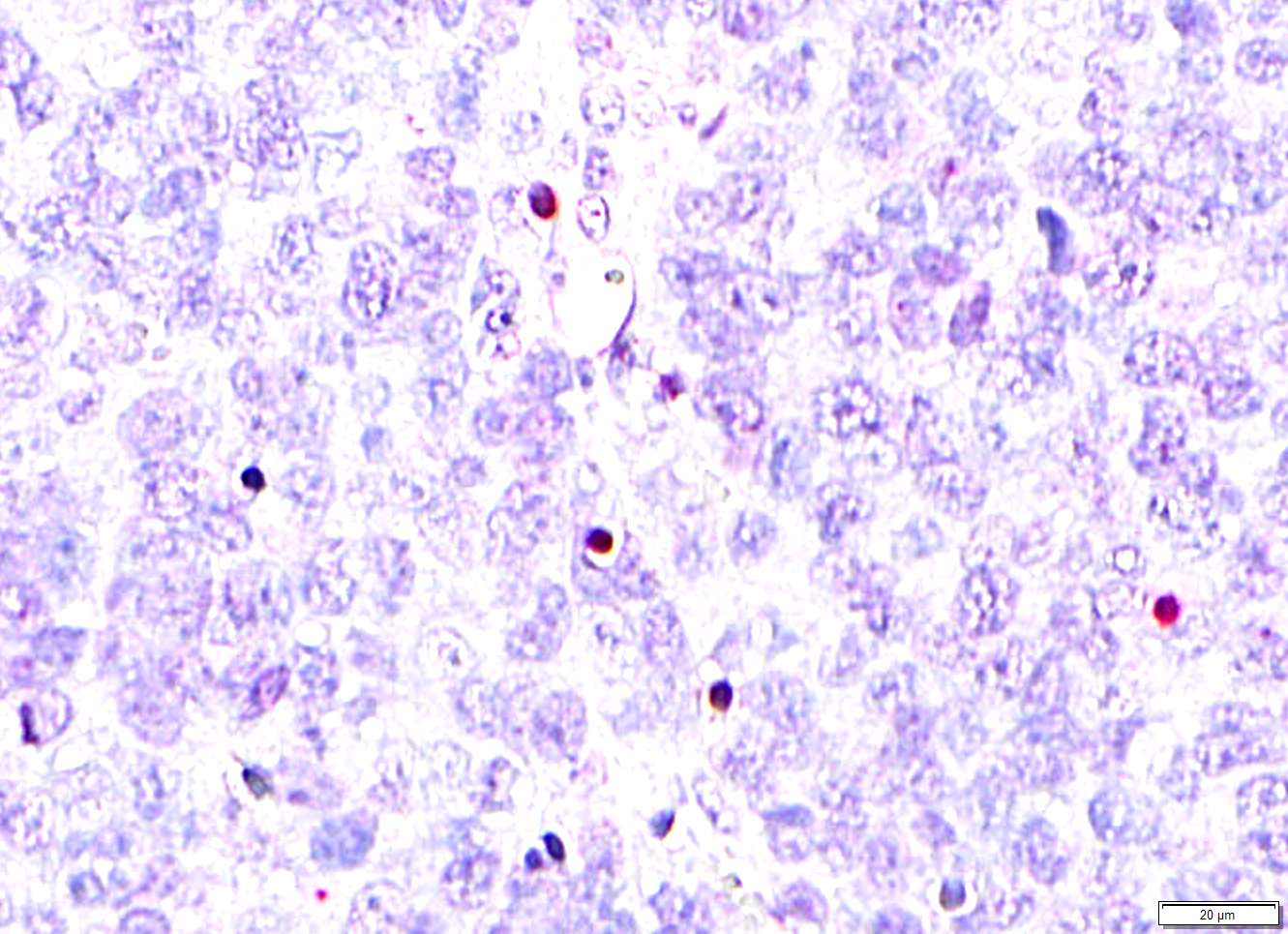

Supplement: Supplementary file 12 — Source data Fig. 4 [file 44321_2024_97_MOESM12_ESM.zip › Fig 4/Fig_4G/TUNEL/Tunel sh2 20-6.png]

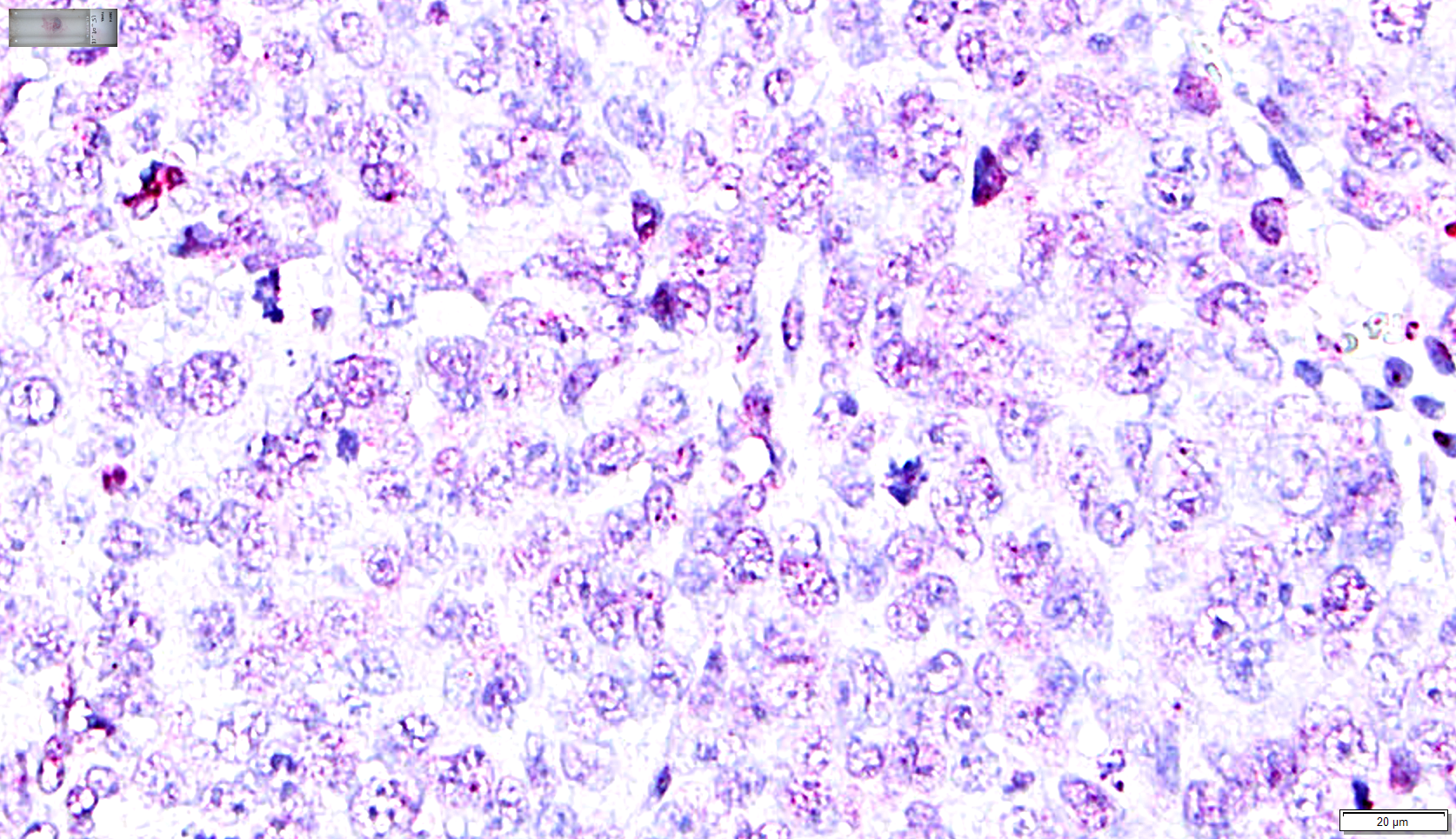

Supplement: Supplementary file 12 — Source data Fig. 4 [file 44321_2024_97_MOESM12_ESM.zip › Fig 4/Fig_4G/TUNEL/Tunel sh2 20.png]

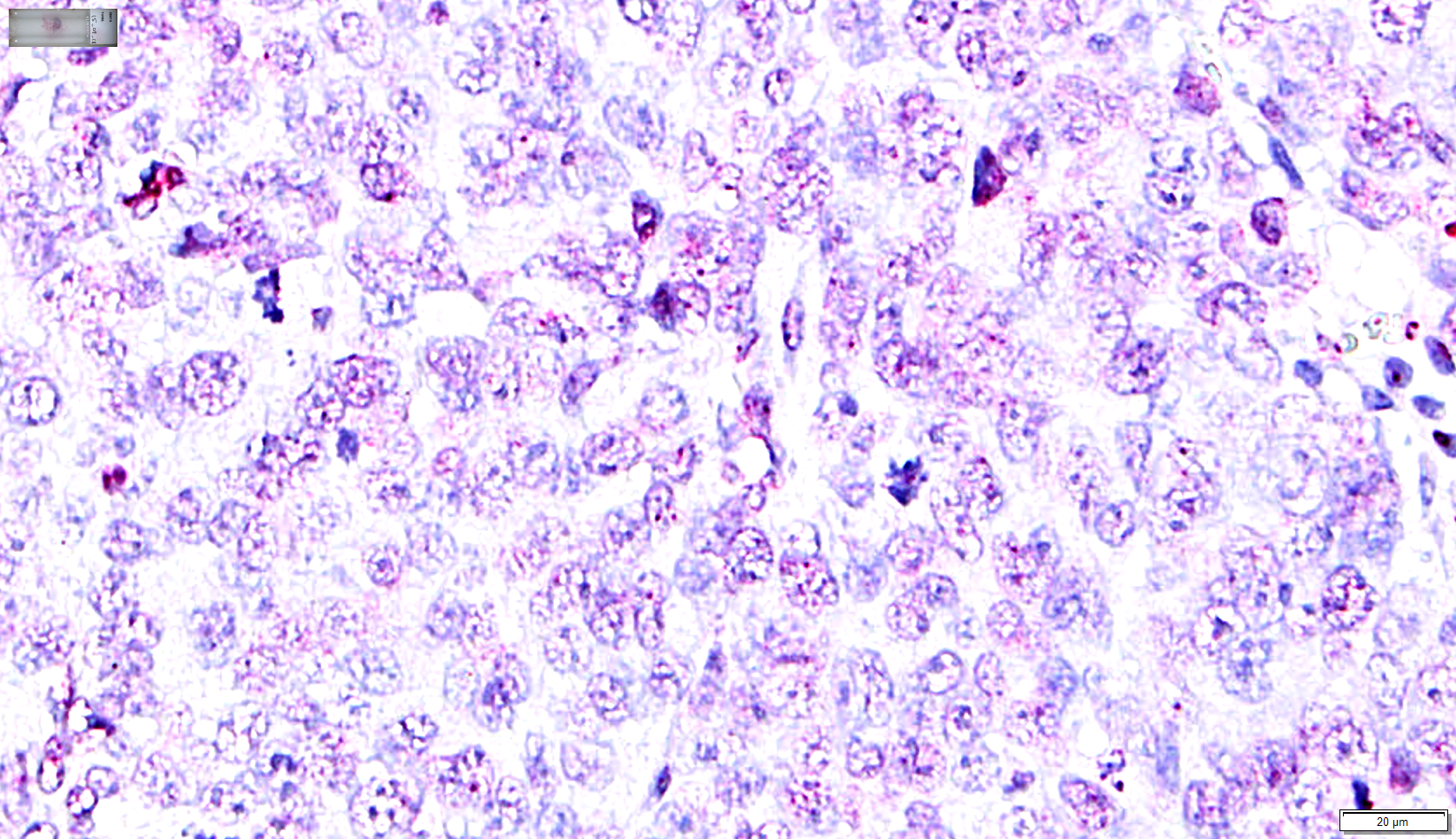

Supplement: Supplementary file 12 — Source data Fig. 4 [file 44321_2024_97_MOESM12_ESM.zip › Fig 4/Fig_4G/TUNEL/Tunel sh2 20.tif]

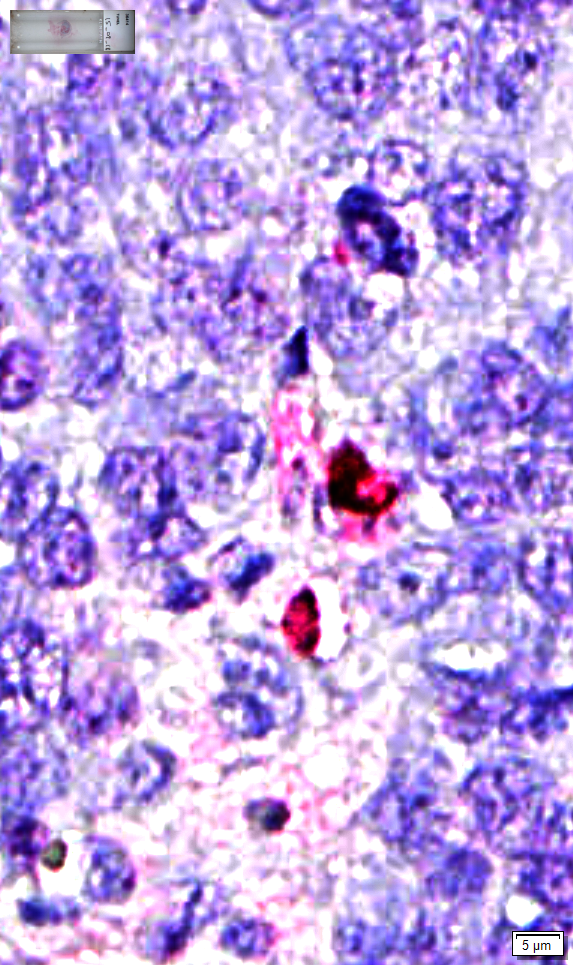

Supplement: Supplementary file 12 — Source data Fig. 4 [file 44321_2024_97_MOESM12_ESM.zip › Fig 4/Fig_4G/TUNEL/Tunel sh2-5 μm.png]

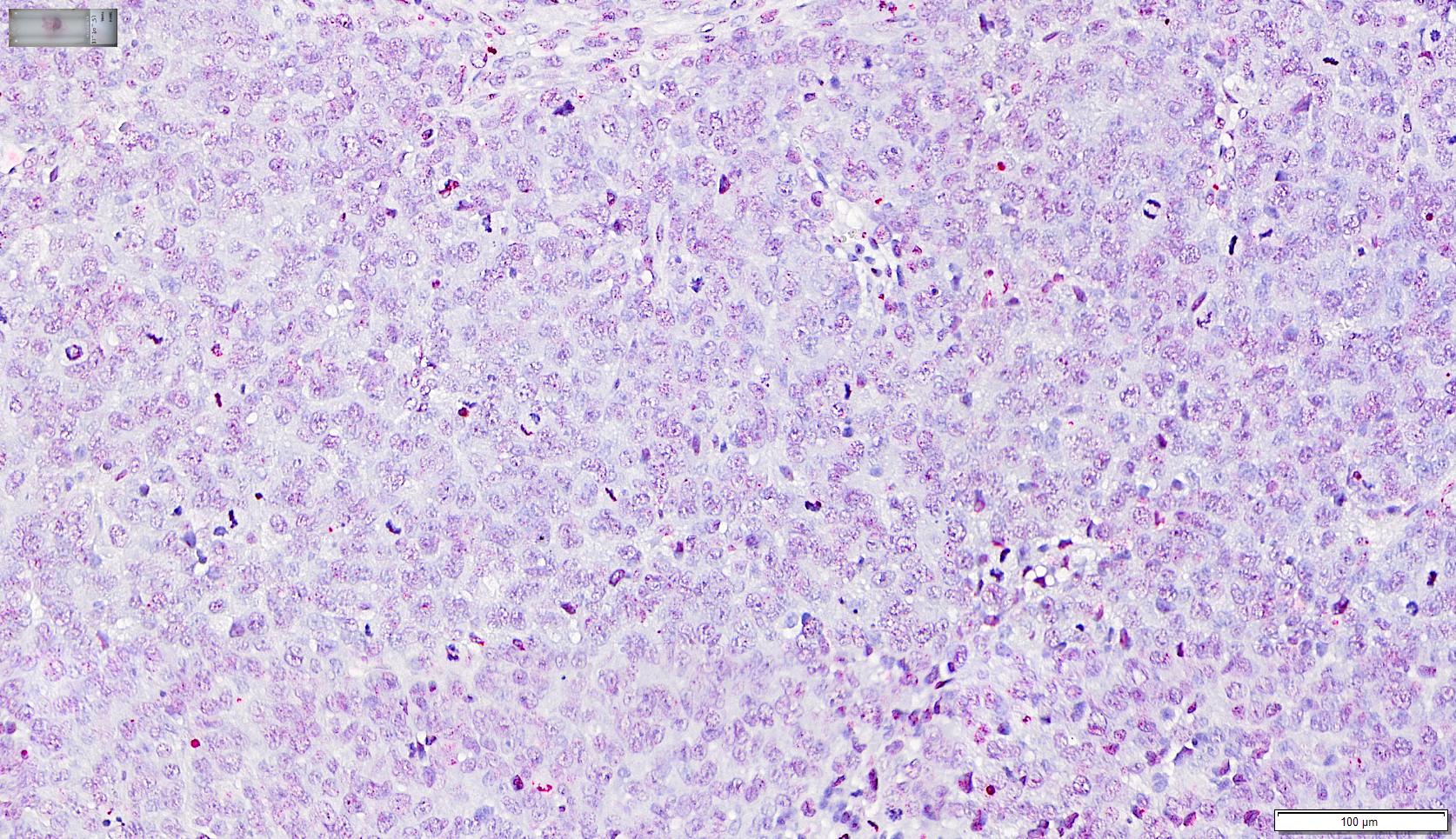

Supplement: Supplementary file 12 — Source data Fig. 4 [file 44321_2024_97_MOESM12_ESM.zip › Fig 4/Fig_4G/TUNEL/Tunel sh2-50-1.png]

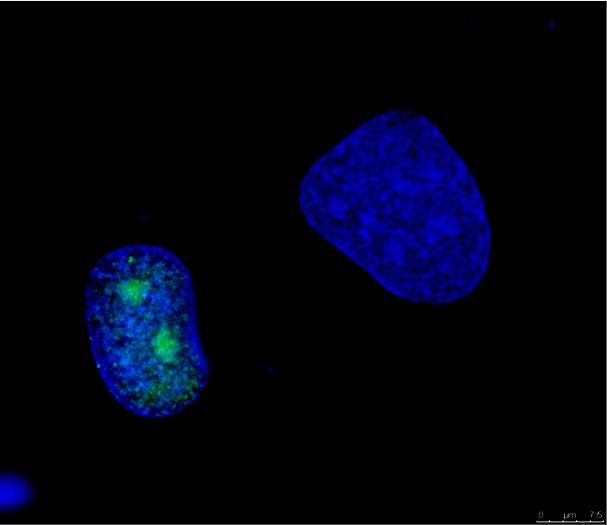

Supplement: Supplementary file 14 — Source data Fig. 6 [file 44321_2024_97_MOESM14_ESM.zip › Fig 6/Fig_6C/DiPRO1_YFP-1.jpg]

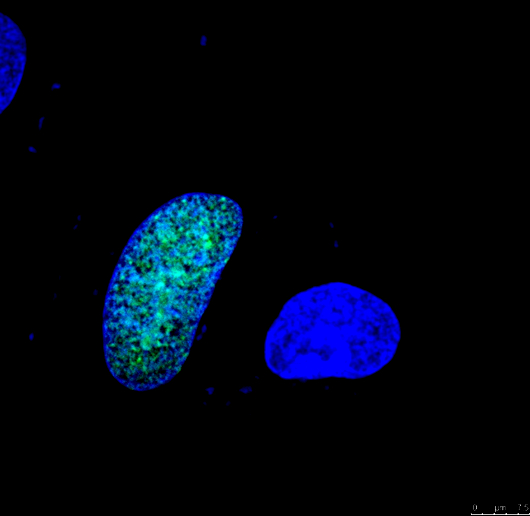

Supplement: Supplementary file 14 — Source data Fig. 6 [file 44321_2024_97_MOESM14_ESM.zip › Fig 6/Fig_6C/DiPRO1_YFP-2.jpg]

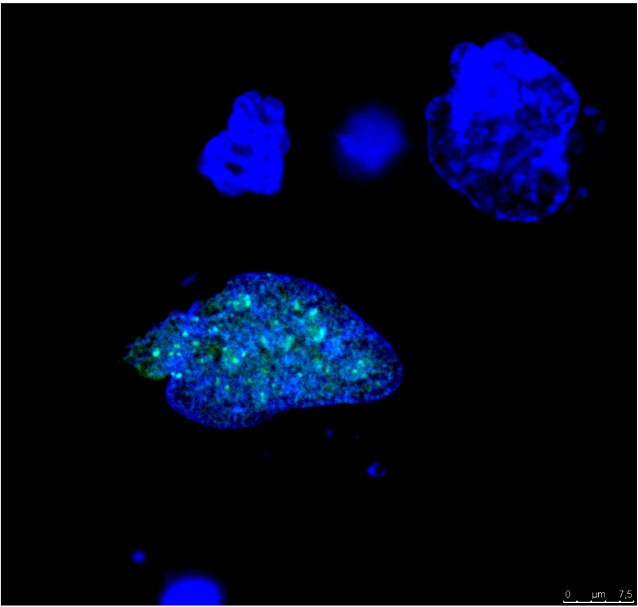

Supplement: Supplementary file 14 — Source data Fig. 6 [file 44321_2024_97_MOESM14_ESM.zip › Fig 6/Fig_6C/DiPRO1_YFP-3.jpg]

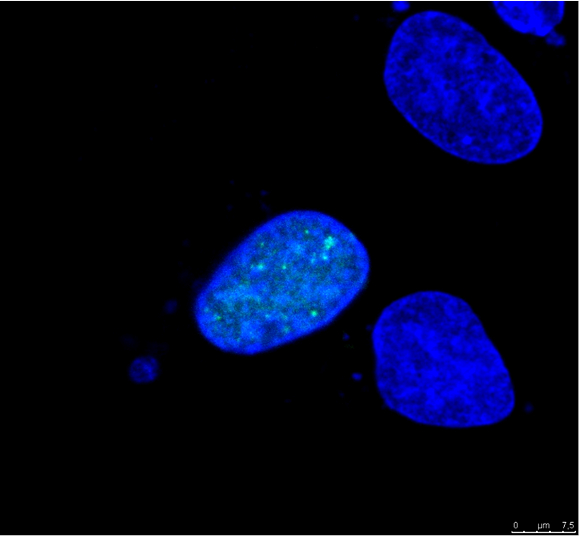

Supplement: Supplementary file 14 — Source data Fig. 6 [file 44321_2024_97_MOESM14_ESM.zip › Fig 6/Fig_6C/DiPRO1_YFP-4.jpg]

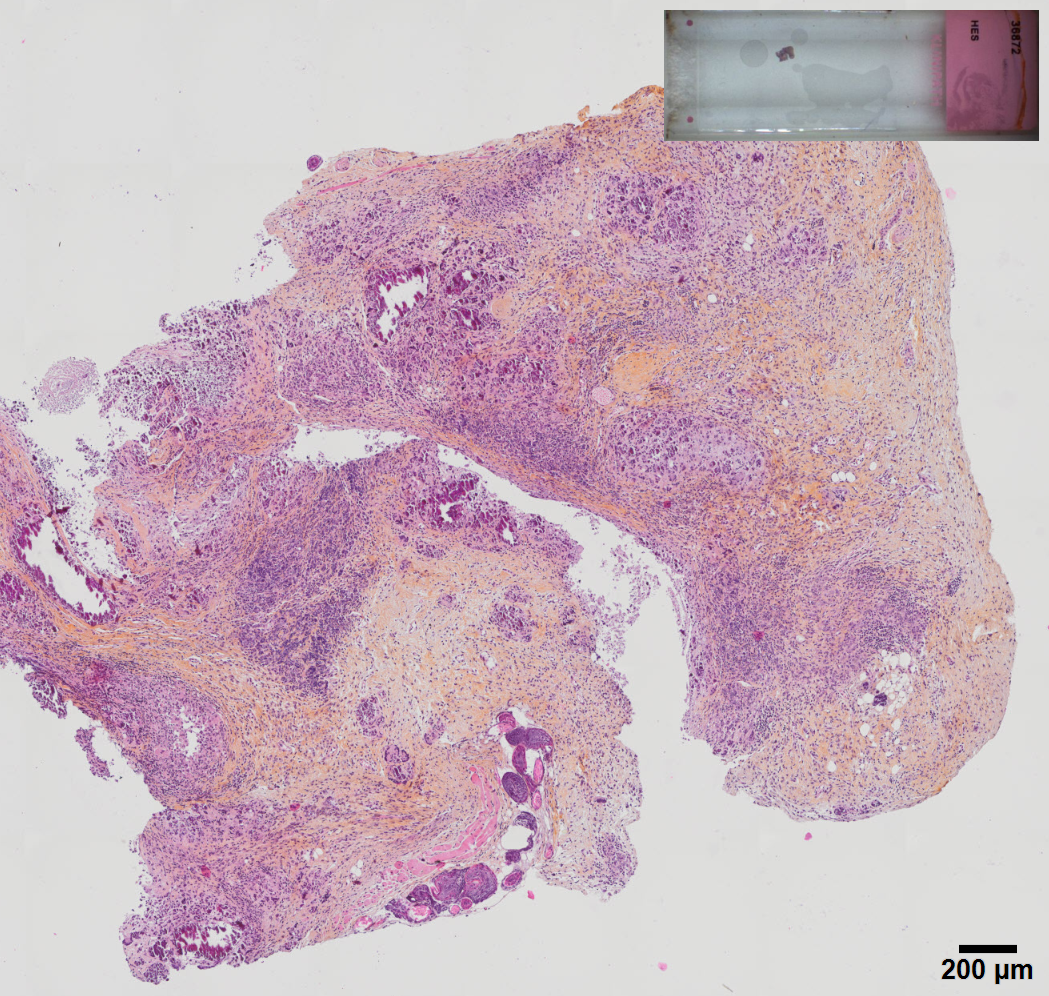

Supplement: Supplementary file 17 — EV Figure Source Data [file 44321_2024_97_MOESM17_ESM.zip › SourceData FigEV2/Fig_EV2/EV2L/HES sh2 200μm.png]
