# Supplementary material for: DiPRO1 distinctly reprograms muscle and mesenchymal cancer cells
Source: EMBO Mol Med. 2024 Jul 15;16(8):4. doi: 10.1038/s44321-024-00097-z (PMC11319797; doi:10.1038/s44321-024-00097-z)
Supplement: Supplementary file 15 — Source data Fig. 7 [file 44321_2024_97_MOESM15_ESM.zip › Fig 7/Fig_7C/Myo_UP_PGE.pdf]

PGE

Positional Gene Enrichment analysis of gene sets for high resolution identification of overrepresented chromosomal regions. Please cite De Preter, K., Barriot, R., Speleman, F., Vandesompele, J., Moreau, Y., 2008, *Nucleic Acids Res.*

This site is a backup mirror of the original Web site http://homes.esat.kuleuven.be/~bioiuser/pge/ which is undergoing maintenance.

Query	Unmapped IDs	Raw Results	BED	Chromosomes view	Make another query

RESULTS SUMMARY:

Mapped Ids: 140 **Unmapped Ids:** 28 **Regions found:** 32 ***p-value* adjustment:** FDR
Significance threshold: 0.05 **Reference dataset:** symbols
To view in a genome browser (*e.g.* Ensembl or UCSC), you can copy & paste the data in the BED tab in order to 'upload your data' in the genome browser. You can also access the same data at this address. Clicking on a region will direct you to Ensembl genome browser with a track PGE.

Scale factor: 1 Axis width: 200 (in pixels) Plot what: -10.log p-values
Refresh

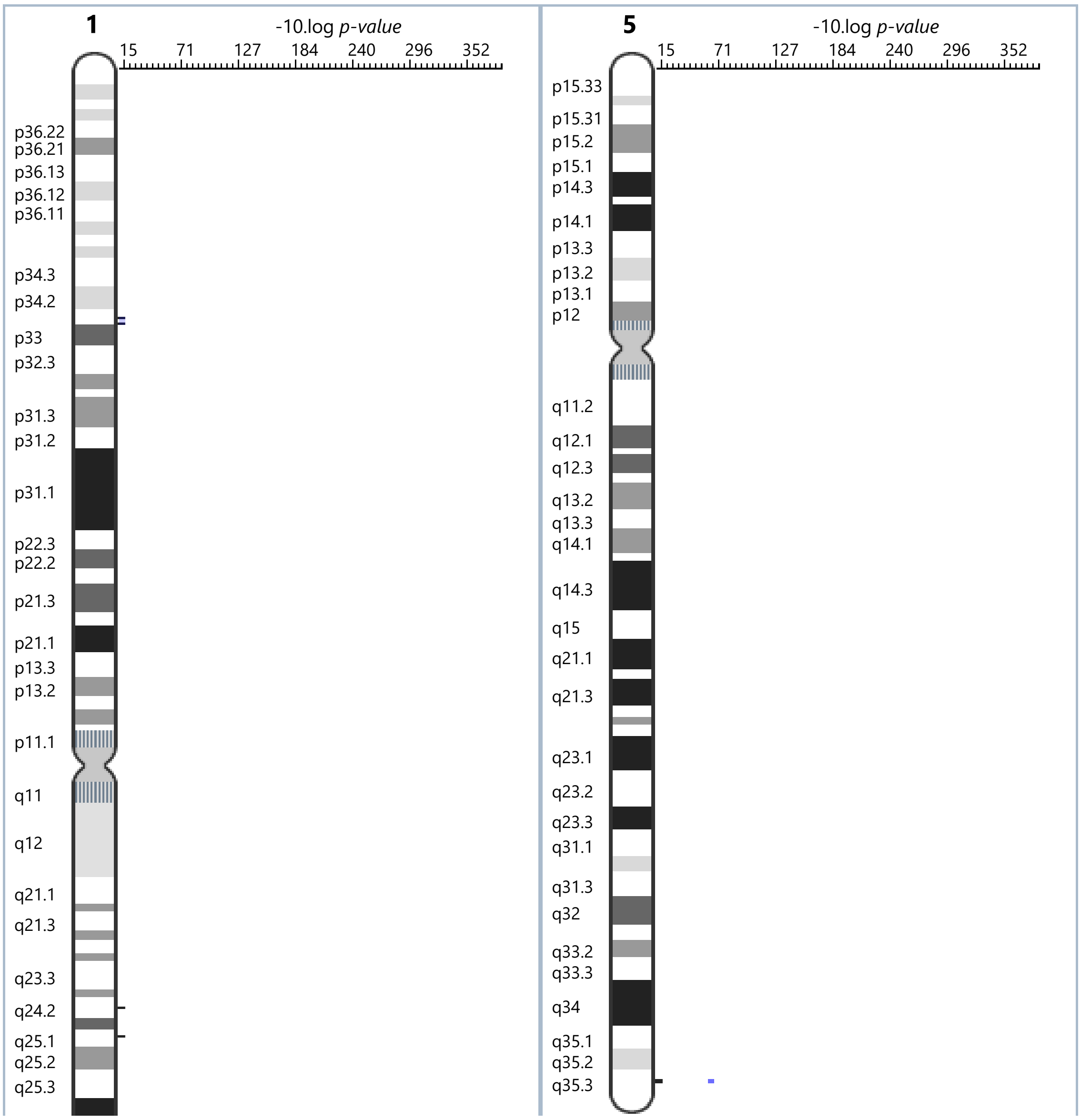

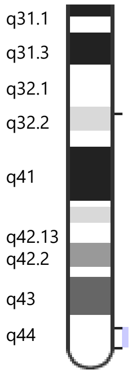
q31.1
q31.3
q32.1
q32.2
q41
q42.13
q42.2
q43
q44

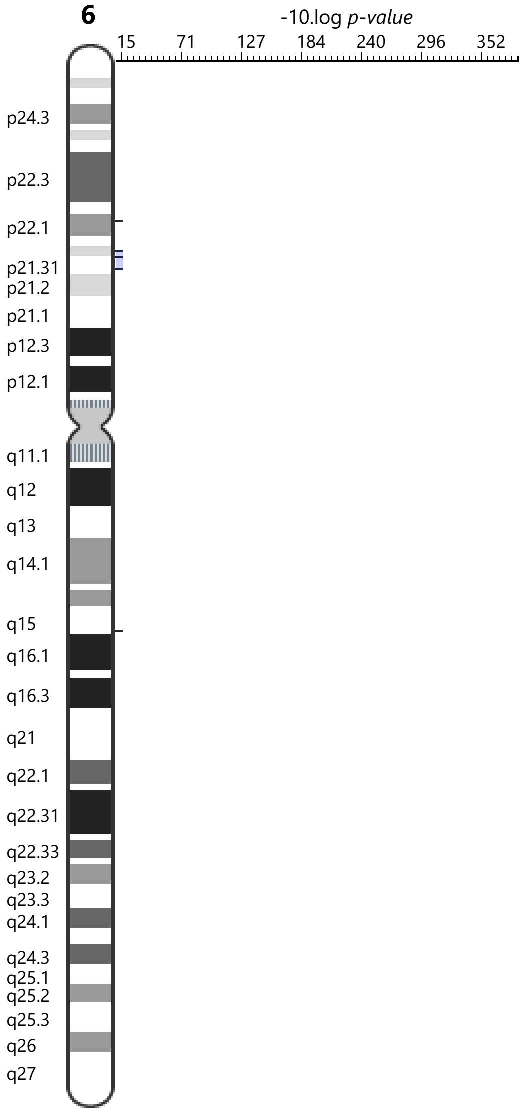
6
-10.log p-value
15 71 127 184 240 296 352
p24.3
p22.3
p22.1
p21.31
p21.2
p21.1
p12.3
p12.1
q11.1
q12
q13
q14.1
q15
q16.1
q16.3
q21
q22.1
q22.31
q22.33
q23.2
q23.3
q24.1
q24.3
q25.1
q25.2
q25.3
q26
q27

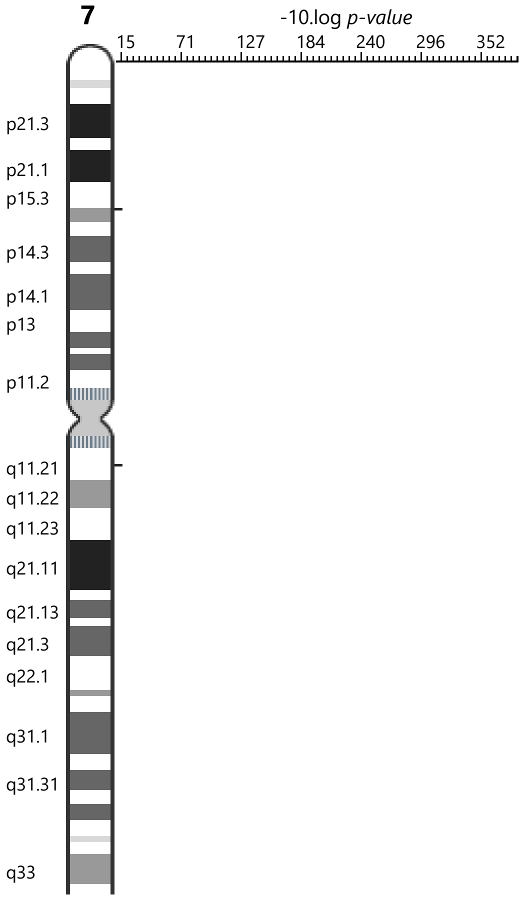
7
-10.log p-value
15 71 127 184 240 296 352
p21.3
p21.1
p15.3
p14.3
p14.1
p13
p11.2
q11.21
q11.22
q11.23
q21.11
q21.13
q21.3
q22.1
q31.1
q31.31
q33

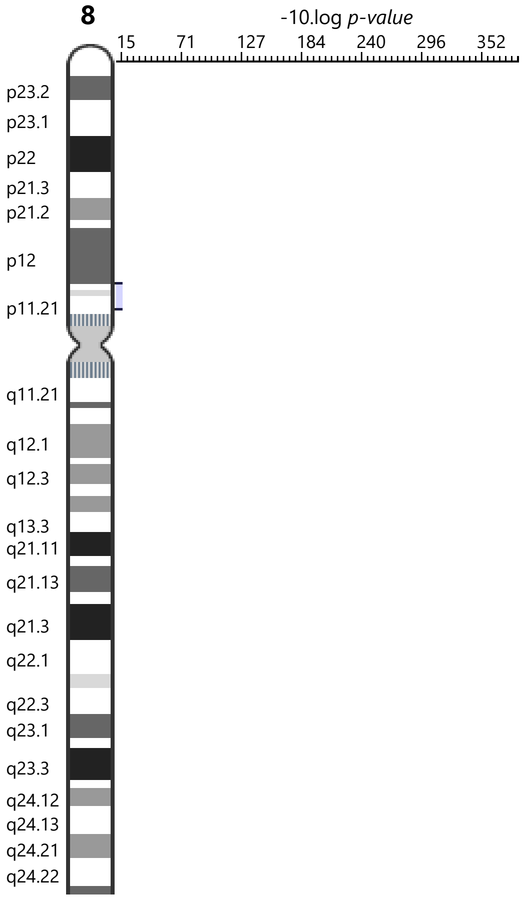
8
-10.log p-value
15 71 127 184 240 296 352
p23.2
p23.1
p22
p21.3
p21.2
p12
p11.21
q11.21
q12.1
q12.3
q13.3
q21.11
q21.13
q21.3
q22.1
q22.3
q23.1
q23.3
q24.12
q24.13
q24.21
q24.22

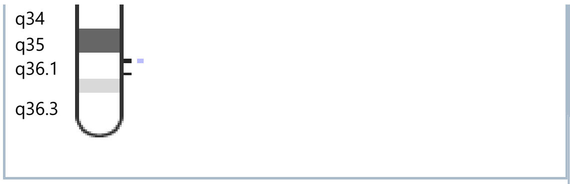
q34
q35
q36.1
q36.3

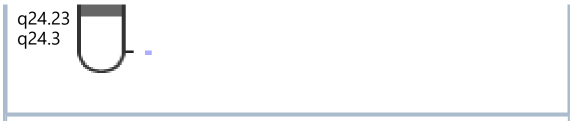
q24.23
q24.3

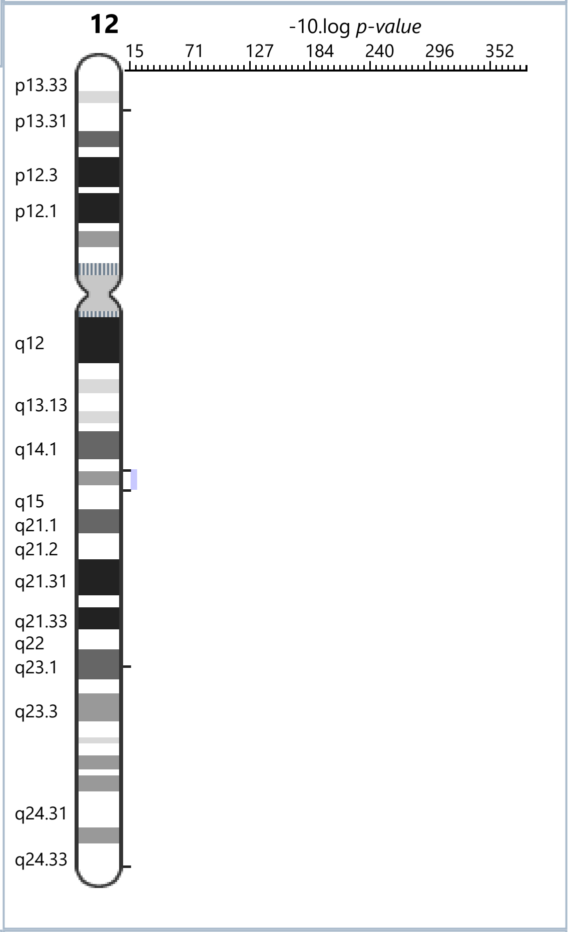
12
-10.log p-value
15 71 127 184 240 296 352
p13.33
p13.31
p12.3
p12.1
q12
q13.13
q14.1
q15
q21.1
q21.2
q21.31
q21.33
q22
q23.1
q23.3
q24.31
q24.33

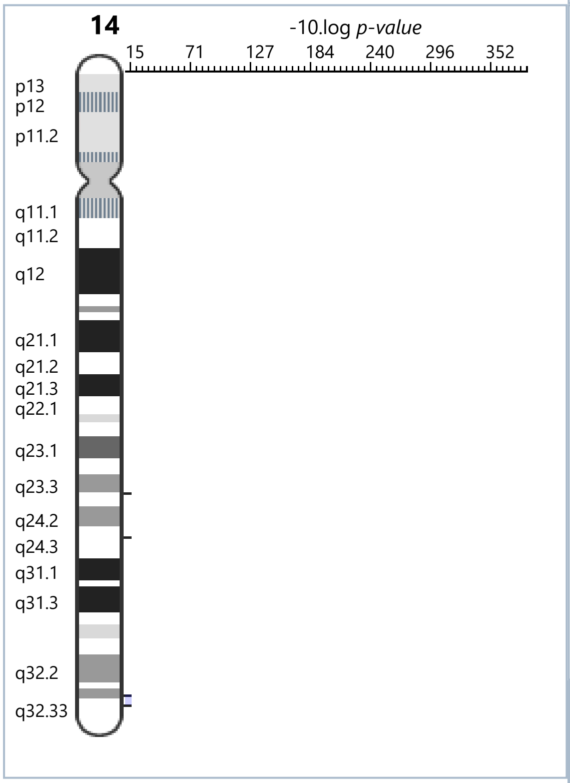
14
-10.log p-value
15 71 127 184 240 296 352
p13
p12
p11.2
q11.1
q11.2
q12
q21.1
q21.2
q21.3
q22.1
q23.1
q23.3
q24.2
q24.3
q31.1
q31.3
q32.2
q32.33

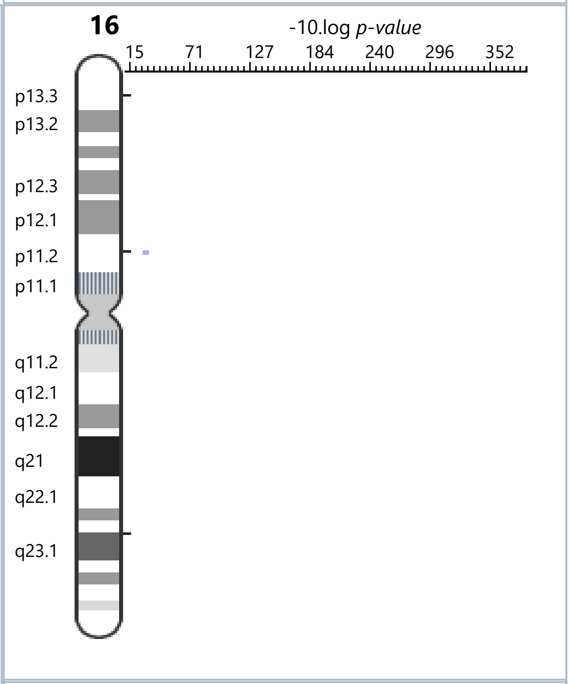
16
-10.log p-value
15 71 127 184 240 296 352
p13.3
p13.2
p12.3
p12.1
p11.2
p11.1
q11.2
q12.1
q12.2
q21
q22.1
q23.1

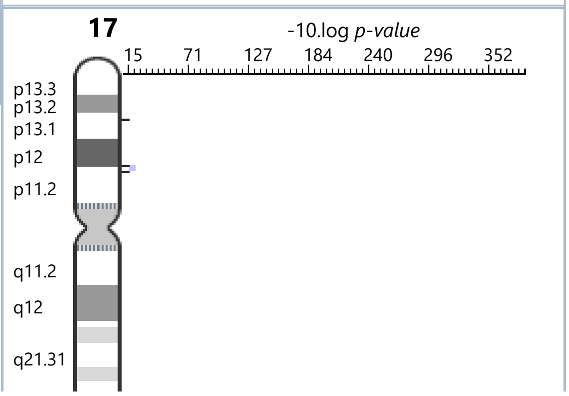
17
-10.log p-value
15 71 127 184 240 296 352
p13.3
p13.2
p13.1
p12
p11.2
q11.2
q12
q21.31

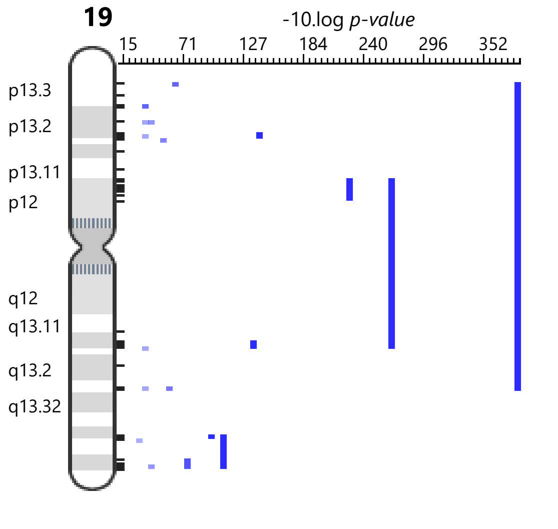

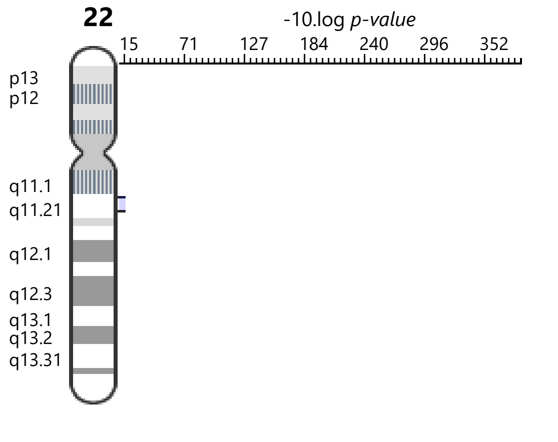

Report a problem | Download PGE Perl script
