# Supplementary material for: DiPRO1 distinctly reprograms muscle and mesenchymal cancer cells
Source: EMBO Mol Med. 2024 Jul 15;16(8):4. doi: 10.1038/s44321-024-00097-z (PMC11319797; doi:10.1038/s44321-024-00097-z)
Supplement: Supplementary file 15 — Source data Fig. 7 [file 44321_2024_97_MOESM15_ESM.zip › Fig 7/Fig_7C/RMS_UP_PGE.pdf]

PGE

Positional Gene Enrichment analysis of gene sets for high resolution identification of overrepresented chromosomal regions. Please cite De Preter, K., Barriot, R., Speleman, F., Vandesompele, J., Moreau, Y., 2008, *Nucleic Acids Res.*

This site is a backup mirror of the original Web site http://homes.esat.kuleuven.be/~bioiuser/pge/ which is undergoing maintenance.

Query	Unmapped IDs	Raw Results	BED	Chromosomes view	Make another query

RESULTS SUMMARY:

Mapped Ids: 5841 **Unmapped Ids:** 5141 **Regions found:** 10 ***p-value*** **adjustment:** FDR **Significance threshold:** 0.05 **Reference dataset:** symbols

To view in a genome browser (*e.g.* Ensembl or UCSC), you can copy & paste the data in the BED tab in order to 'upload your data' in the genome browser. You can also access the same data at this address. Clicking on a region will direct you to Ensembl genome browser with a track PGE.

Scale factor: 1 Axis width: 300 (in pixels) Plot what: -10.log p-values

Refresh

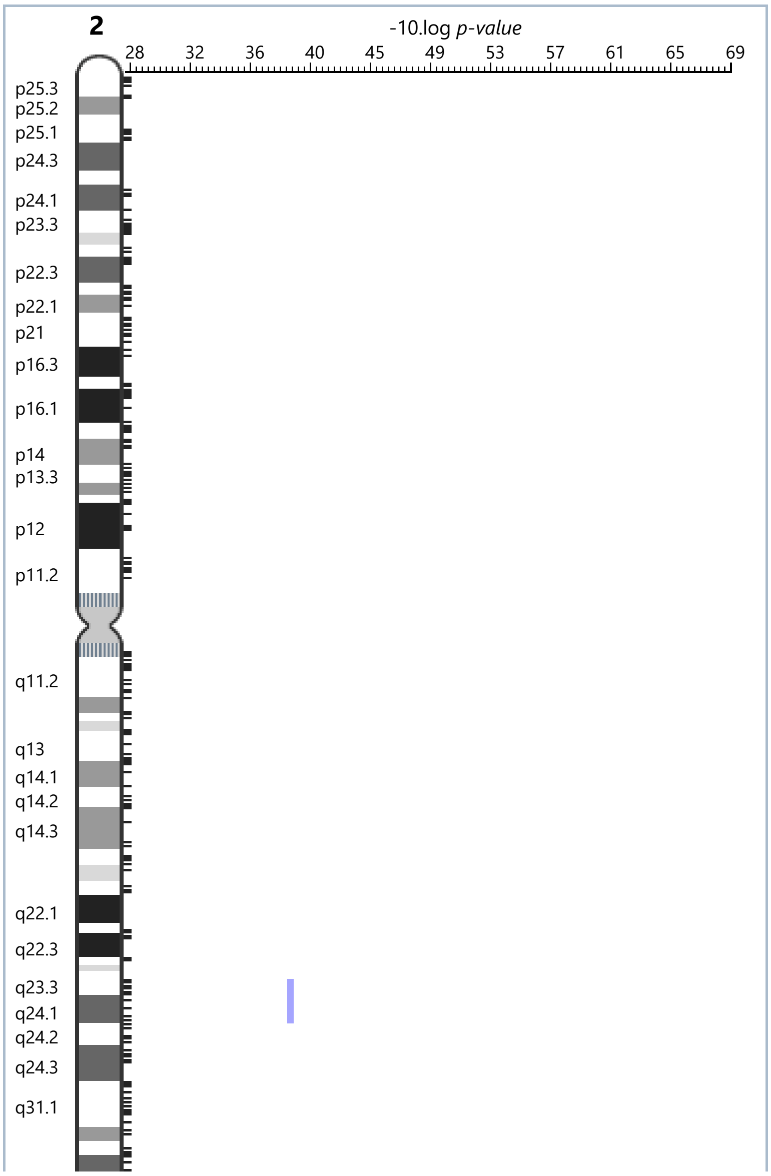

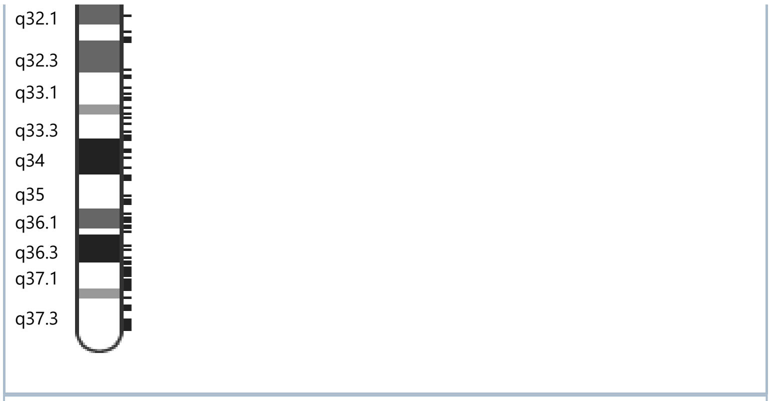
q32.1
q32.3
q33.1
q33.3
q34
q35
q36.1
q36.3
q37.1
q37.3

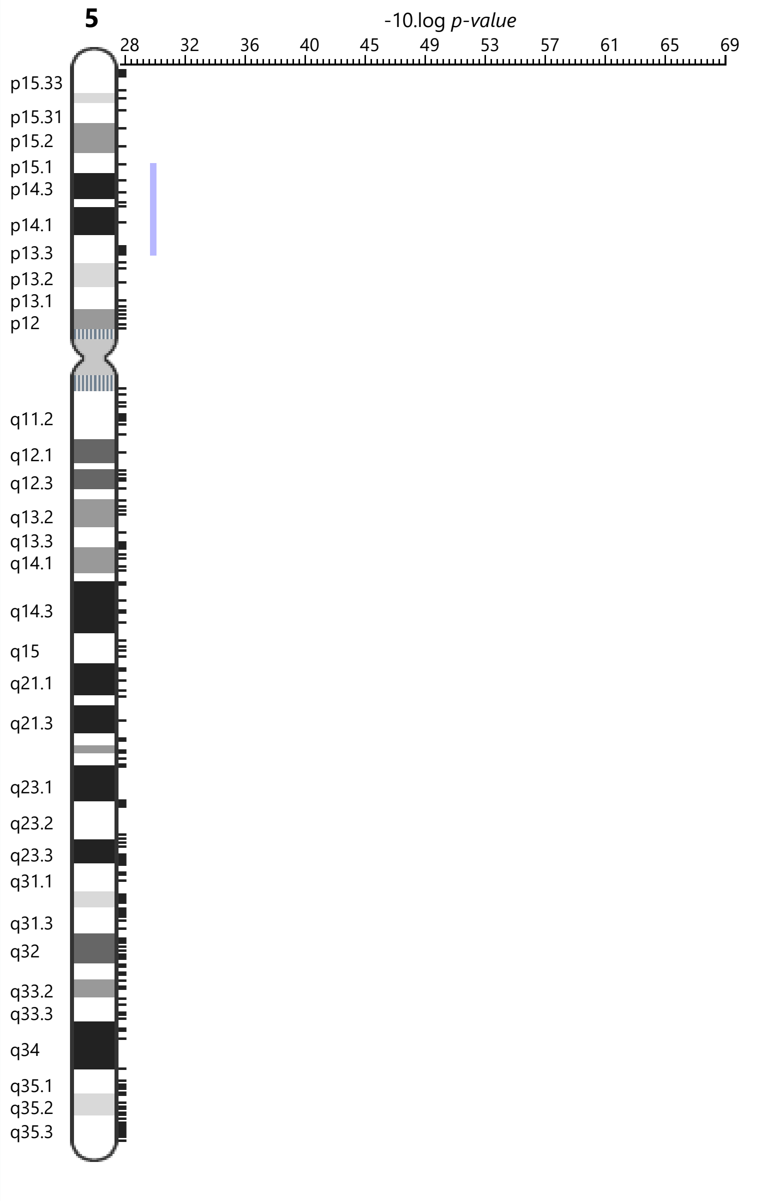
5
-10.log p-value
28 32 36 40 45 49 53 57 61 65 69
p15.33
p15.31
p15.2
p15.1
p14.3
p14.1
p13.3
p13.2
p13.1
p12
q11.2
q12.1
q12.3
q13.2
q13.3
q14.1
q14.3
q15
q21.1
q21.3
q23.1
q23.2
q23.3
q31.1
q31.3
q32
q33.2
q33.3
q34
q35.1
q35.2
q35.3

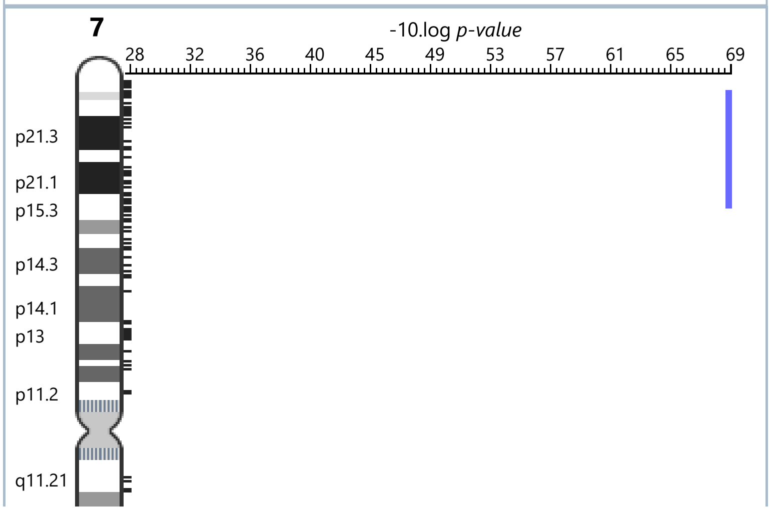
7
-10.log p-value
28 32 36 40 45 49 53 57 61 65 69
p21.3
p21.1
p15.3
p14.3
p14.1
p13
p11.2
q11.21

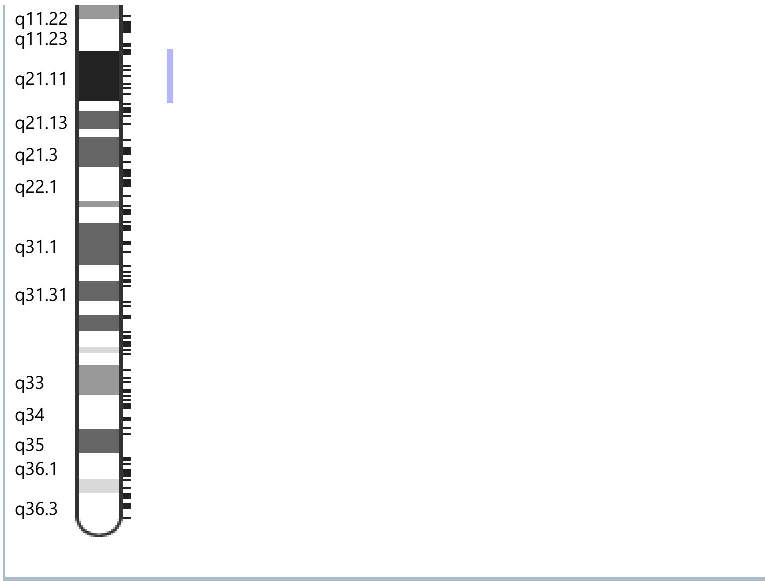
q11.22
q11.23
q21.11
q21.13
q21.3
q22.1
q31.1
q31.31
q33
q34
q35
q36.1
q36.3

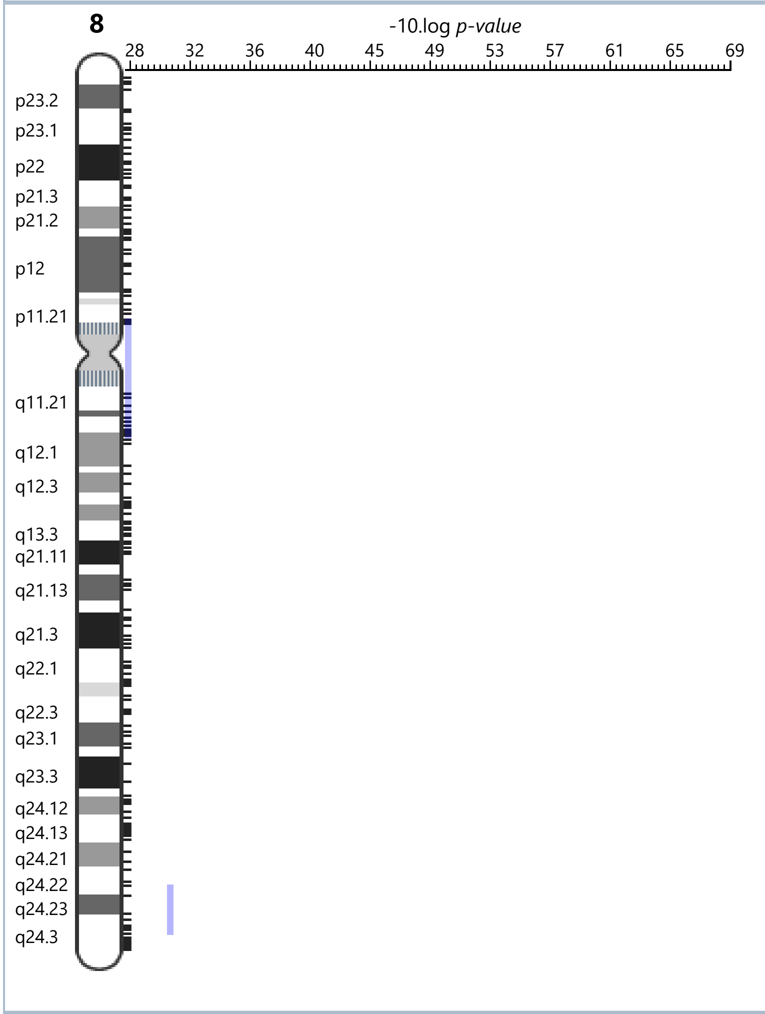
8
-10.log *p-value*
28 32 36 40 45 49 53 57 61 65 69
p23.2
p23.1
p22
p21.3
p21.2
p12
p11.21
q11.21
q12.1
q12.3
q13.3
q21.11
q21.13
q21.3
q22.1
q22.3
q23.1
q23.3
q24.12
q24.13
q24.21
q24.22
q24.23
q24.3

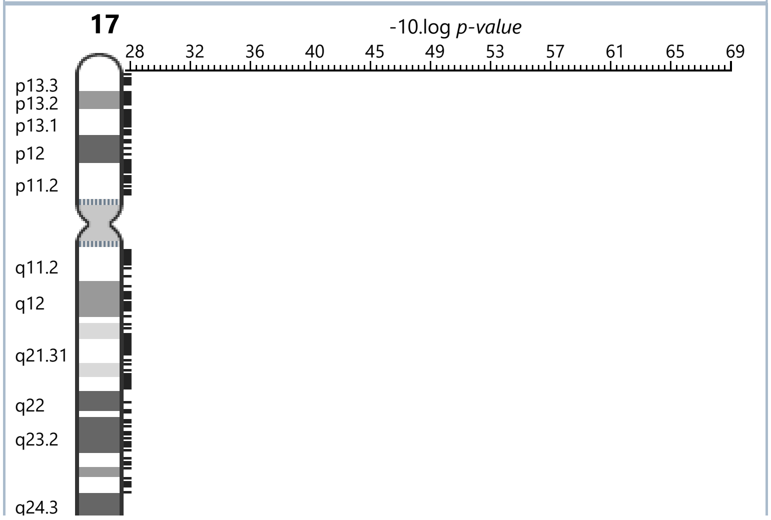
17
-10.log *p-value*
28 32 36 40 45 49 53 57 61 65 69
p13.3
p13.2
p13.1
p12
p11.2
q11.2
q12
q21.31
q22
q23.2
q24.3

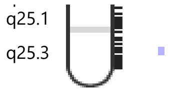

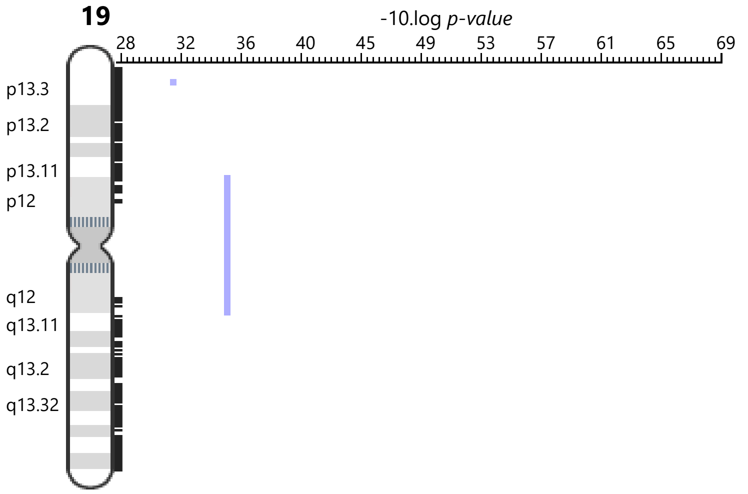

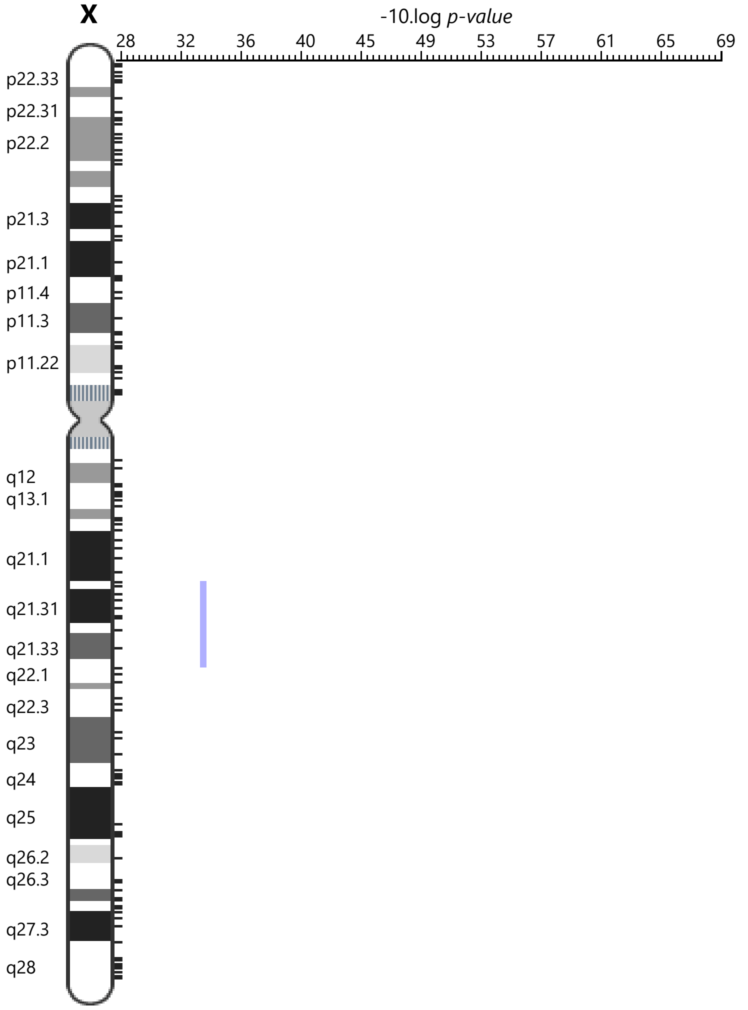

Report a problem | Download PGE Perl script
