# Supplementary material for: DiPRO1 distinctly reprograms muscle and mesenchymal cancer cells
Source: EMBO Mol Med. 2024 Jul 15;16(8):4. doi: 10.1038/s44321-024-00097-z (PMC11319797; doi:10.1038/s44321-024-00097-z)
Supplement: Supplementary file 18 — Expanded View Figures [file 44321_2024_97_MOESM18_ESM.pdf]

Expanded View Figures

Figure EV1. DiPRO1 regulates myogenic genes of human myoblasts.

(**A–C**) Myoblasts were stably transduced with a retroviral vector expressing the DiPRO1 ORF (pDiPRO1, $n = 6$) and compared with their parental counterparts (Ctl, $n = 6$). (**A**) Heatmap revealing differential expression of a set of genes in control (blue) and pDiPRO1 (red) samples. Upregulation of DiPRO1 gene expression (**B**) stimulates expression of PAX-family genes (**C**). (**D**) Cell death analysis of shDiPRO1 myoblasts. The cells transduced with lentiviral vector expressing shDiPRO1 or nontargeting shCtl, were stained with propidium iodide (PI) and analyzed by flow cytometry. The percentage of dead cells according to DNA content was compared to control cells, proportions (%) ± SD, $n = 3$ corresponding to three independent experiments. (**E–G**) Human myoblasts were transduced with a lentiviral vector expressing a DiPRO1-targeting shRNA (shDiPRO1, $n = 7$) and compared to myoblasts transduced with the equivalent vector expressing a nontargeting shRNA (shCTL, $n = 6$). The analysis was performed one week post-transduction. (**E**) Heatmap revealing changes in gene expression profile related to DiPRO1 knockdown in myoblasts. (**F**) DiPRO1 expression was significantly inhibited by shDiPRO1 (red) relative to shCtl (blue). (**G**) Clustered functional network of 185 upregulated (UP) and 136 downregulated (DW) genes encompassing muscle-related functions, negatively correlated with DiPRO1 expression in myoblats. Muscle GO terms are represented by nodes. Two networks were confronted. Green clusters correspond to upregulated myogenic genes, while lilac clusters correspond to downregulated genes in shDiPRO1 versus shCtl. Cytoscape software with ClueGO plug-in was used, kappa score = 0.4. (**H**) PCA analysis of differentially expressed genes separates pDiPRO1 ($n = 6$) and control according to the first principal component and shDiPRO1 ($n = 7$) and control ($n = 6$) according to the second principal component. (**I**) DiPRO1 upregulation in RMS and Ewing's sarcoma (ES) cell lines. RNA-seq data were extracted from the DepMap portal (Broad Institute). The whisker plot shows median (Q2) ± Q3-Q1 ± 1.5x IQR of ZNF555 expression by cancer type or in all cancer types (ALL). *** ($P < 0.001$) indicates a significant difference with ALL. (**J**) Stable knockdown of RMS cells was achieved by transduction of lentiviral vectors producing nontargeting shRNA (shCtl) or DiPRO1-targeting shRNAs (shDiPRO1). Cell cycle analysis of TE671/RMS cells lacking DiPRO1 was performed 48 and 72 h after transduction with vectors expressing DiPRO1-targeting shRNA. Transduced RMS cells were fixed in ethanol and stained with propidium iodide (PI). DNA content and percentage of cells in each cell cycle phase were determined by flow cytometry. The results represent two independent experiments. (**K**) RMS and myoblast (Myo) cells transfected with shCtl and shDiPRO1 were assayed for caspase-3 activity. The results were normalized to the protein quantity and appropriate shCtl was referenced as 100%. All conditions represent three or six transfections (RMS $n = 6$, Myo $n = 3$), proportions (%) ± SD. (**L**) DiPRO1 induces RMS cell proliferation. RMS cells were stably transduced with a retroviral vector expressing the DiPRO1 ORF (pDiPRO1) and compared with their parental counterparts (Ctl). The proliferation rate was estimated for DiPRO1-overexpressing RMS (pDiPRO1) and their control (Ctl) by Counting Kit 8 (Sigma-Aldrich). Cell number was determined using a titration curve performed at different cell dilutions from 0 to 25,000 cells/well. Cells were then seeded at 2.0×10^3, 5.0×10^3, 1.0×10^4, and 1.5×10^4 cells/well and absorbance was measured at 460 nm after 72 h of proliferation. Viable cells were compared to the initial number of cells. Data were expressed as mean % relative to initial cell number ± SD and represent four independent experiments ($n = 4$). The titration curve for RMS-Ctl is presented in Fig. 2E. Data information: In (**A–C**, **E–H**), Global transcriptome analysis of microarray was implemented using total mRNA. The Limma R package was used for statistical analysis. In (**B**, **C**, **F**, **I**), boxplots represent median (line) ± dispersion, box length IQR = Q3-Q1, whisker length = 1.5 * IQR. In (**D**, **I–L**), Welch's two-sample *t*-test. In (**B–D**, **I**, **K**, **L**), *,**, and *** indicate significant differences with the corresponding control, $P < 0.05$, 0.01, and 0.001, respectively. Source data are available online for this figure.

© The Author(s)

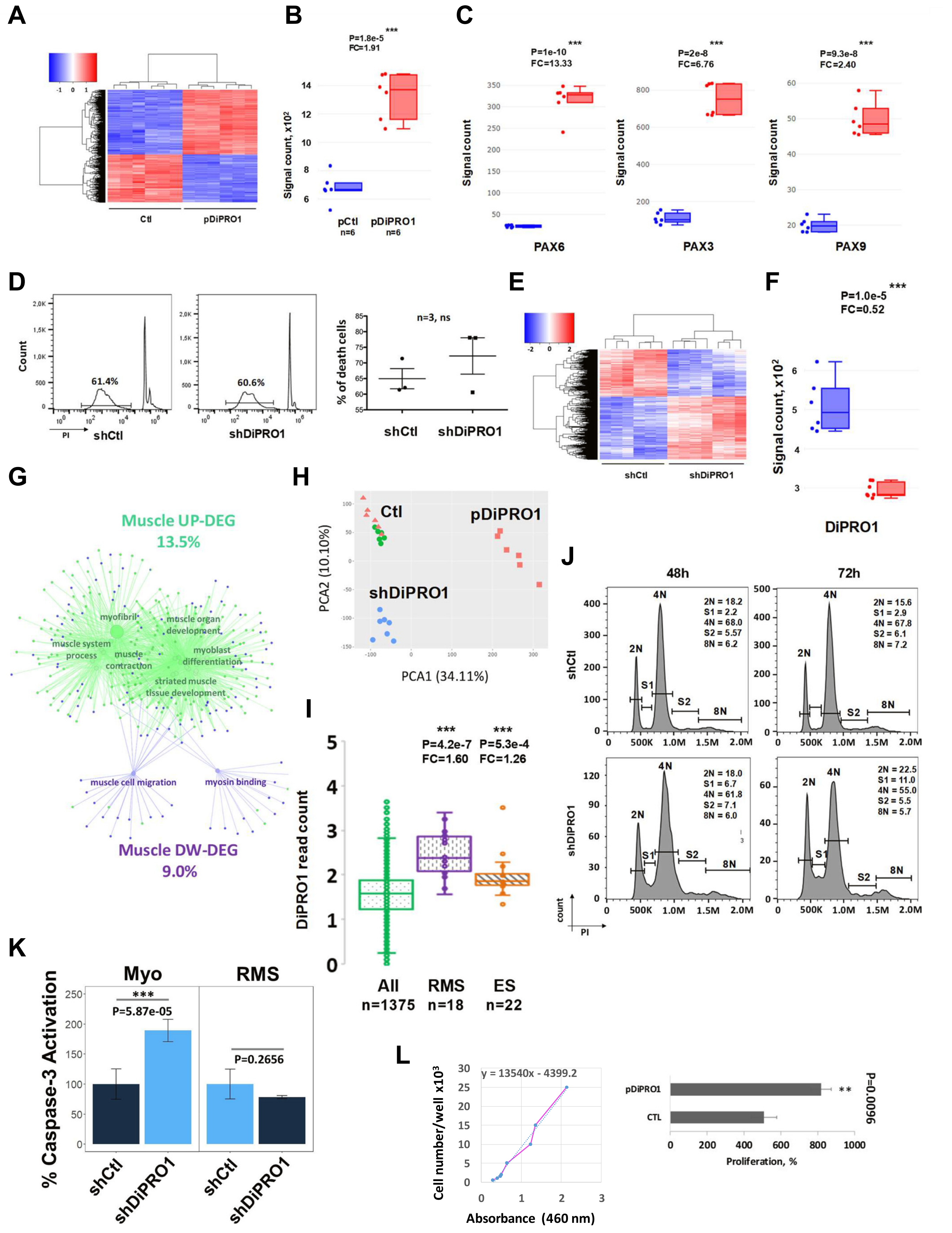
A
Ctl
pDiPRO1
B
P=1.8e-5
FC=1.91
***
Signal count, x10²
pCtl
n=6
pDiPRO1
n=6
C
P=1e-10
FC=13.33
P=2e-8
FC=6.76
P=9.3e-8
FC=2.40
Signal count
PAX6
PAX3
PAX9
D
Count
61.4%
60.6%
PI
shCtl
shDiPRO1
n=3, ns
% of death cells
E
shCtl
shDiPRO1
F
P=1.0e-5
FC=0.52
Signal count, x10²
DiPRO1
G
Muscle UP-DEG
13.5%
myofibril
muscle organ development
muscle system process
muscle contraction
myoblast differentiation
striated muscle tissue development
muscle cell migration
myosin binding
Muscle DW-DEG
9.0%
H
Ctl
pDiPRO1
shDiPRO1
PCA2 (10.10%)
PCA1 (34.11%)
I
P=4.2e-7
FC=1.60
P=5.3e-4
FC=1.26
DiPRO1 read count
All
n=1375
RMS
n=18
ES
n=22
J
48h
72h
shCtl
shDiPRO1
2N = 18.2
S1 = 2.2
4N = 68.0
S2 = 5.57
8N = 6.2
2N = 15.6
S1 = 2.9
4N = 67.8
S2 = 6.1
8N = 7.2
2N = 18.0
S1 = 6.7
4N = 61.8
S2 = 7.1
8N = 6.0
2N = 22.5
S1 = 11.0
4N = 55.0
S2 = 5.5
8N = 5.7
count
PI
K
Myo
RMS
% Caspase-3 Activation
P=5.87e-05
P=0.2656
shCtl
shDiPRO1
L
y = 13540x - 4399.2
Cell number/well x10³
Absorbance (460 nm)
pDiPRO1
CTL
Proliferation, %
P=0.0096

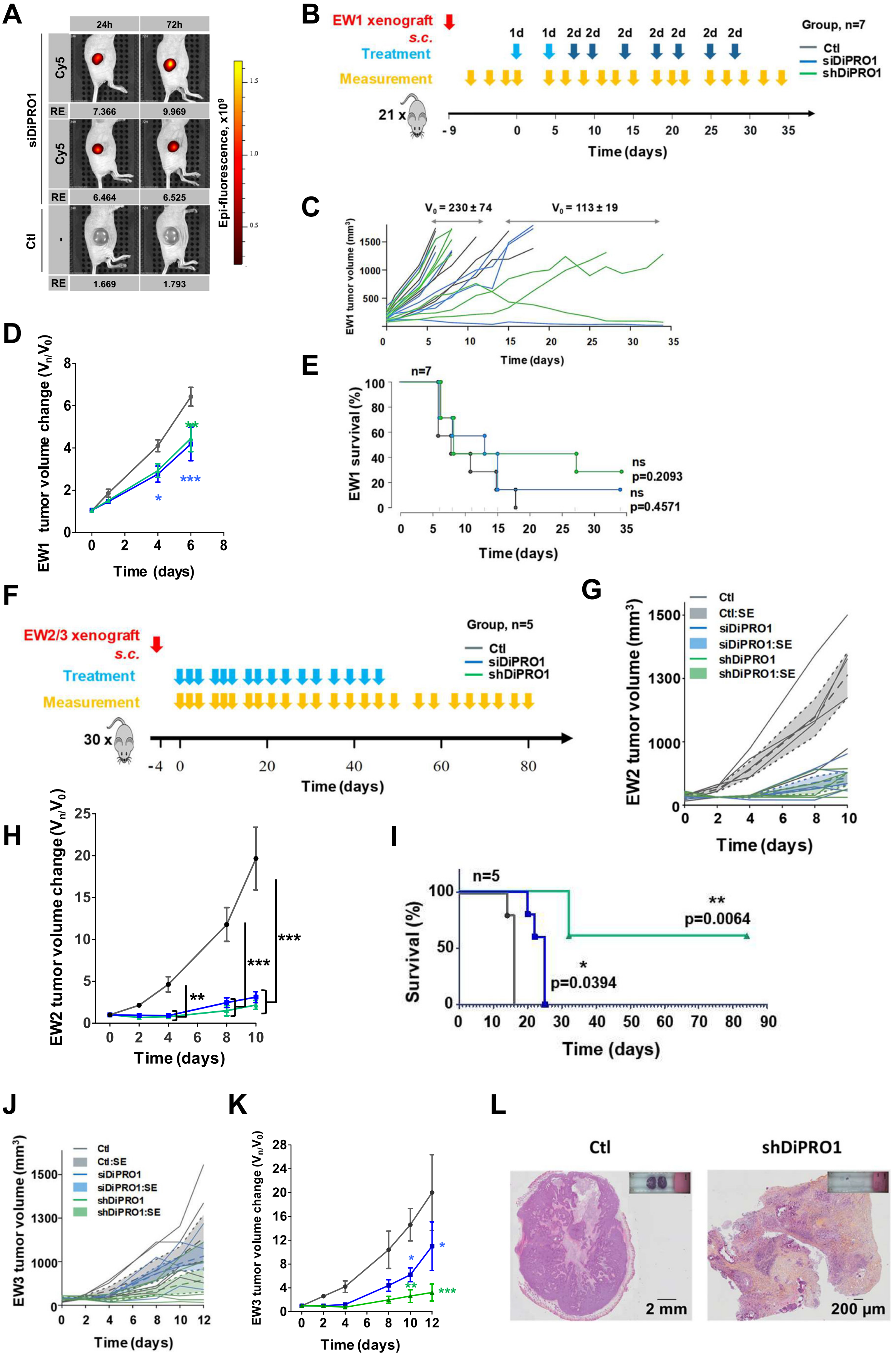
A
24h
72h
siDiPRO1
Cy5
RE
7.366
9.969
6.464
6.525
Ctl
1.669
1.793
Epi-fluorescence, x10⁹
B
EW1 xenograft
s.c.
Treatment
Measurement
1d 1d 2d 2d 2d 2d 2d 2d 2d
Group, n=7
Ctl
siDiPRO1
shDiPRO1
21 x
Time (days)
C
V₀ = 230 ± 74
V₀ = 113 ± 19
EW1 tumor volume (mm³)
Time (days)
D
EW1 tumor volume change (Vn/V0)
Time (days)
E
n=7
EW1 survival (%)
ns
p=0.2093
ns
p=0.4571
Time (days)
F
EW2/3 xenograft
s.c.
Treatment
Measurement
Group, n=5
Ctl
siDiPRO1
shDiPRO1
30 x
Time (days)
G
Ctl
Ctl:SE
siDiPRO1
siDiPRO1:SE
shDiPRO1
shDiPRO1:SE
EW2 tumor volume (mm³)
Time (days)
H
EW2 tumor volume change (Vn/V0)
Time (days)
I
n=5
Survival (%)
p=0.0064
p=0.0394
Time (days)
J
Ctl
Ctl:SE
siDiPRO1
siDiPRO1:SE
shDiPRO1
shDiPRO1:SE
EW3 tumor volume (mm³)
Time (days)
K
EW3 tumor volume change (Vn/V0)
Time (days)
L
Ctl
shDiPRO1
2 mm
200 µm

© The Author(s)

◀ **Figure EV2. DiPRO1 inhibitors compromise tumor growth in Ewing's sarcoma xenograft models.**

Nude mice received s.c. inoculations of A673 Ewing's sarcoma (EW) cells and were treated with siDiPRO1/jetPEI®, shDiPRO1/jetPEI®, and siCtl/jetPEI® scramble (Ctl) nanocomposites at 0.5 or 1 mg/kg/d. Three independent experiments were performed. (**A**) Tumor cell uptake of siDiPRO1/jetPEI®/Cy5 nanocomposites. Specific anti-DiPRO1 siRNA complexes coupled to Cy5 were delivered at a single dose (0.5 mg/kg/) by intratumoral injection into two nude mice bearing Ewing sarcoma tumors. Internalization was monitored in live mice 24 and 72 h post-treatment and radiant efficacy (RE) was measured. Control mice were treated with an equimolar dose of nontargeting siCtl/jetPEI® free of Cy5. (**B–E**) EW1 xenograft model. (**B**) Schematic showing the timing of tumor injection and treatment with DiPRO1 inhibitors. Mice were treated for 25 days at a dose of 0.5 mg/kg/inj (1d) twice-weekly for the first week, and then the dose was doubled (2d) for the rest of the treatment. (**C**) Growth curves of individual EW1 tumors (V, mm3). (**D**) Tumor progression was compared between the treated and control groups ($n = 7$). (**E**) Kaplan–Meier survival curves for treated mice. Overall survival was followed for 34 days ($n = 7$). The log-rank test (Mantel-Cox) was applied for each pairwise comparison, proportion (%) ± SEM. The *p* value was adjusted by Holm method. (**F–L**) EW2 and EW3 xenograft models. (**F**) Schematic showing the timing of tumor injection and treatment with DiPRO1 inhibitors. Mice were treated with siDiPRO1/jetPEI®, shDiPRO1/jetPEI®, and scrambled siRNA/jetPEI® (Ctl) nanocomposites for 80 days at a dose of 0.5 mg/kg/d (1d). The intensive three-dose-per-week regimen was used for the first 2 weeks, followed by a two-dose-per-week regimen until the end of treatment. (**G**) Growth curves of individual EW2 tumors (V, mm^3). (**H**) EW2 tumor progression was compared between the treated and control groups ($n = 5$). (**I**) Kaplan–Meier survival curves for treated mice bearing EW2 tumor xenografts. Overall survival was followed for 84 days. The log-rank test (Mantel-Cox) for pairwise comparison with control was applied for analysis, proportion (%) ± SEM. The *p* value was adjusted by the Holm method. (**J**) Growth curves of individual EW3 tumors (V, mm3). (**K**) EW3 tumor progression was compared between treatment and control groups ($n = 5$). (**L**) Representative HES staining of tumor sections from a scrambled control and an Ewing's sarcoma tumor (EW2) treated with shDiPRO1/jetPEI® shows the absence of viable tumor cells in the treated residual tumor 84 days after xenografting. Data information: In (**D**, **H**, **K**), a two-way ANOVA test was used for statistical analysis, V_n/V_0 ratio ± SEM. In (**D**, **E**, **H**, **I**, **K**), *$P < 0.05$, **$P < 0.01$, ***$P < 0.001$. Source data are available online for this figure.

 © The Author(s)

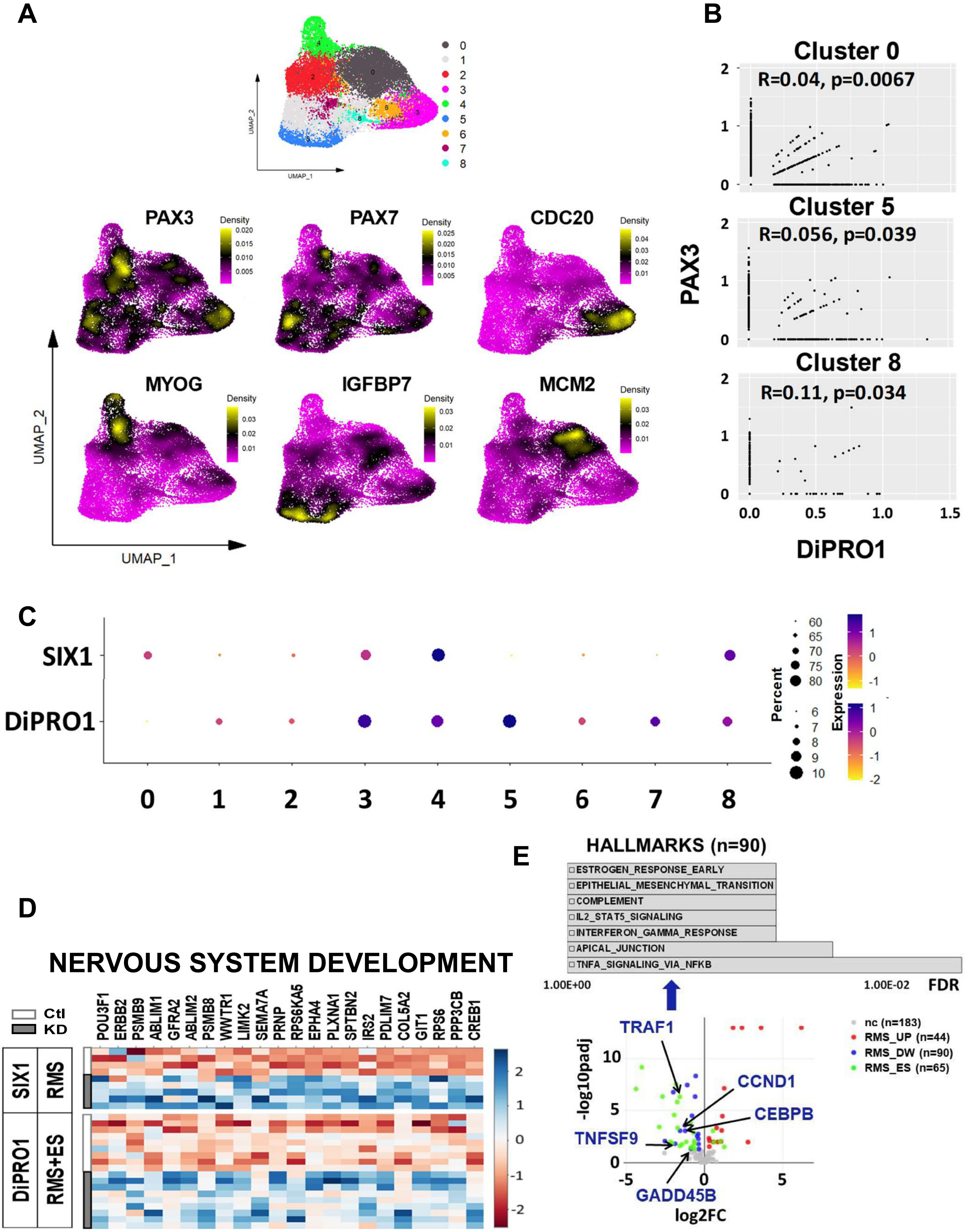
A
UMAP_1
UMAP_2
0
1
2
3
4
5
6
7
8
PAX3
PAX7
CDC20
MYOG
IGFBP7
MCM2
Density
B
Cluster 0
R=0.04, p=0.0067
Cluster 5
R=0.056, p=0.039
Cluster 8
R=0.11, p=0.034
PAX3
DiPRO1
C
SIX1
DiPRO1
Percent
Expression
D
NERVOUS SYSTEM DEVELOPMENT
Ctl
KD
SIX1
RMS
DiPRO1
RMS+ES
POU3F1
ERBB2
PSMB9
ABLIM1
GFRA2
ABLIM2
PSMB8
WWTR1
LIMK2
SEMA7A
PRNP
RPS6KA5
EPHA4
PLXNA1
SPTBN2
IRS2
PDLIM7
COL5A2
GIT1
RPS6
PPP3CB
CREB1
E
HALLMARKS (n=90)
ESTROGEN_RESPONSE_EARLY
EPITHELIAL_MESENCHYMAL_TRANSITION
COMPLEMENT
IL2_STAT5_SIGNALING
INTERFERON_GAMMA_RESPONSE
APICAL_JUNCTION
TNFA_SIGNALING_VIA_NFKB
1.00E+00
1.00E-02
FDR
TRAF1
CCND1
CEBPB
TNFSF9
GADD45B
-log10padj
log2FC
nc (n=183)
RMS_UP (n=44)
RMS_DW (n=90)
RMS_ES (n=65)

Figure EV3. Expression profiling of DiPRO1, SIX1, and their target genes in PDX tumors and tumor biopsies of RMS and Ewing sarcoma patients.

(**A–C**) scRNAseq datasets (GSE218974) derived from PDX primary RMS cultures ($n = 3$ eRMS and $n = 3$ aRMS) were reanalyzed using Seurat R toolkit. The individual data were normalized, feature-selected, and integrated into the individual data samples using the SCTransform function. (**A**) Integrated UMAP plot showing RMS cell populations expressing lineage-specific marker genes. Expression density analysis was performed using the R package scCustomize. (**B**) Coexpression of DiPRO1 and PAX3 genes in muscle/mesenchymal progenitors and S-phase cells using stat_cor(method = "pearson") R function. Pearson correlation coefficient (R) and *p* value represents the statistical significance of the linear relationship between two gene expression, $n = 6$. (**C**) Dot plot showing DiPRO1 and SIX1 gene expression in different RMS cell populations. (**D**) Heatmap of common expression gene set of the nervous system development KEGG pathway across DiPRO1 KD ($n = 9$) and SIX1 KD ($n = 4$) samples and appropriate controls of RMS and Ewing sarcoma (ES) cells. (**E**) Volcano plot representing differently upregulated (red) and downregulated (blue) DiPRO1 *cis*-target genes in RMS ($n = 37$) compared to other pediatric tumors (Other, $n = 265$), marked in blue. Common DEGs with Ewing sarcoma (ES, $n = 25$) are highlighted in green. Hallmark enrichment analysis of downregulated DEGs ($n = 90$) (blue arrow) in RMS was performed using the MSigDB database (FDR <0.05). Data information: In (**E**), RNA-seq data of primary tumor biopsies were processed using DEBrowser, DESeq2 (TMM normalization, local fit, LRT test), *p*Vadj <0.05.

 © The Author(s)

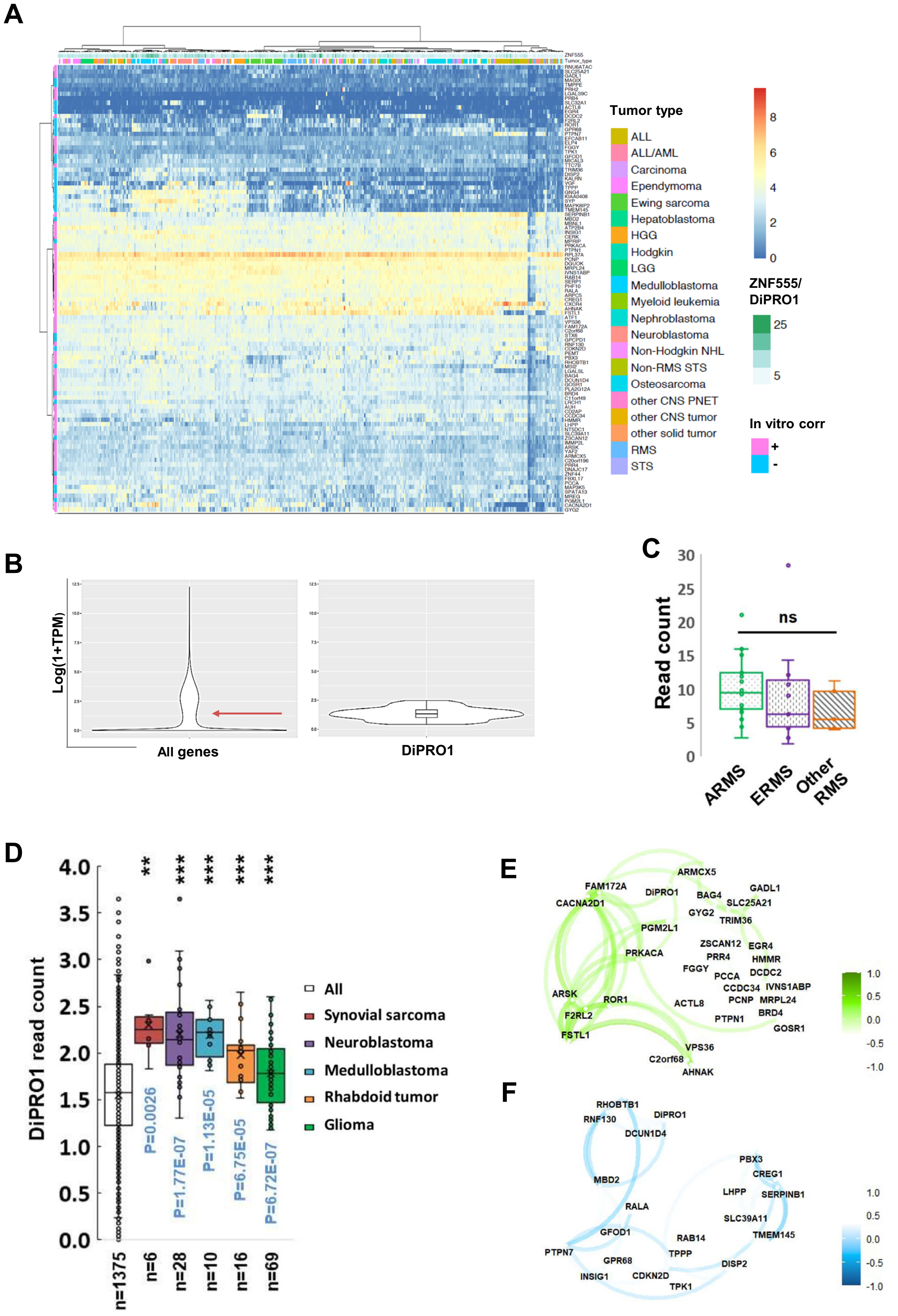
A
Tumor type
ALL
ALL/AML
Carcinoma
Ependymoma
Ewing sarcoma
Hepatoblastoma
HGG
Hodgkin
LGG
Medulloblastoma
Myeloid leukemia
Nephroblastoma
Neuroblastoma
Non-Hodgkin NHL
Non-RMS STS
Osteosarcoma
other CNS PNET
other CNS tumor
other solid tumor
RMS
STS
8
6
4
2
0
ZNF555/ DiPRO1
25
5
In vitro corr
+
-
B
Log(1+TPM)
All genes
DiPRO1
C
Read count
ns
ARMS
ERMS
Other RMS
D
DiPRO1 read count
All
Synovial sarcoma
Neuroblastoma
Medulloblastoma
Rhabdoid tumor
Glioma
P=0.0026
P=1.77E-07
P=1.13E-05
P=6.75E-05
P=6.72E-07
n=1375
n=6
n=28
n=10
n=16
n=69
E
ARMCX5
FAM172A
DiPRO1
BAG4
GADL1
CACNA2D1
SLC25A21
GYG2
TRIM36
PGM2L1
ZSCAN12
EGR4
PRKACA
PRR4
HMMR
FGGY
PCCA
DCDC2
CCDC34
IVNS1ABP
ARSK
PCNP
MRPL24
ROR1
ACTL8
BRD4
F2RL2
PTPN1
GOSR1
FSTL1
VPS36
C2orf68
AHNAK
F
RHOBTB1
RNF130
DiPRO1
DCUN1D4
PBX3
MBD2
CREG1
LHPP
SERPINB1
RALA
SLC39A11
GFOD1
RAB14
TMEM145
PTPN7
TPPP
GPR68
DISP2
INSIG1
CDKN2D
TPK1

© The Author(s)

Figure EV4. Clinical relevance of the DiPRO1 gene and its downstream targets in cancer.

(**A**) Unsupervised clustering analysis shows upregulation of the ZNF555/DiPRO1 gene in several tumor types. Genes positively (+) and negatively (−) correlated with DiPRO1 expression in in vitro DiPRO1 knockdown experiments are shown on the left side. (**B**) The variation in DiPRO1 expression levels among other genes in pediatric tumors ($n = 327$) Violin plots show the distribution of gene expression for all genes (left) and specifically for DiPRO1 (right). The median of the DiPRO1 expression is denoted by the red arrow. Additionally, the box plot presents a median (Q2) ± Q3-Q1 ± 1.5x IQR. (**C**) Boxplots of DiPRO1 expression in RMS subtypes; ARMS: alveolar RMS ($n = 21$), ERMS: embryonic RMS ($n = 11$) and other RMS ($n = 5$). The data are presented as median (Q2) ± Q3-Q1 ± 1.5x IQR. Statistical analysis using the *t*-test showed no significant (ns) difference between RMS subtypes ($P > 0.1591$). (**D**, **E**) RNA-seq data of cancer cell lines were retrieved from the DepMap portal (Broad Institute). (**D**) The whisker plot displays DiPRO1 expression across different cancer types: synovial sarcoma ($n = 6$), neuroblastoma ($n = 28$), medulloblastoma ($n = 10$), rhabdoid tumor ($n = 16$), and glioma ($n = 69$), as well as in all cancer types combined ($n = 1375$) from Dataset EV2. The results depict mean (cross) and median (Q2) ± Q3-Q1 ± 1.5x IQR. *t*-test was used for analysis. $^{**}P < 0.01$, $^{***}P < 0.001$. *P* values are shown between samples of the indicated cancer type and all cancer samples. Network plot of positive (**E**) and negative (**F**) expression correlation of DiPRO1 downstream targets across cancer cell lines ($n = 1375$). Variables that are more highly correlated appear closer together and are linked by paths (green for positive and blue for negative). The affected DEGs in RMS and Ewing's sarcoma tumor samples were analysed across cancer cell lines. Data information: In (**A**–**E**), RNA-seq data were processed using DEBrowser, the R package DESeq2 (TMM normalization, parametric adjustment, LRT test), FC >1.4, $pV < 0.05$. In (**C**, **D**), *t*-test was used. In (**A**, **E**, **F**), analysis was performed using the Corrr R package (default parameters).

 © The Author(s)

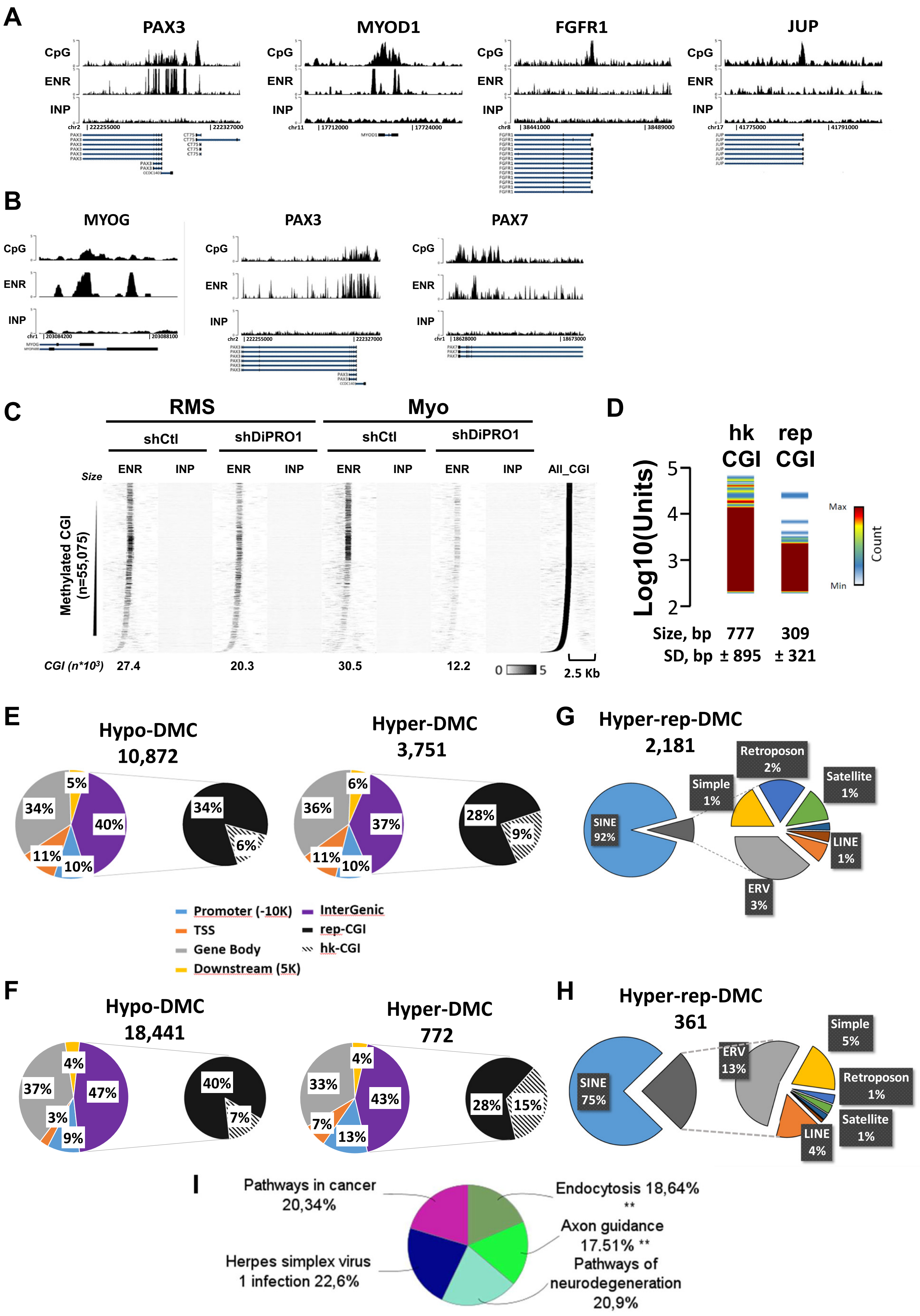
A
PAX3
MYOD1
FGFR1
JUP
CpG
ENR
INP
B
MYOG
PAX3
PAX7
C
RMS
Myo
shCtl
shDiPRO1
ENR
INP
All_CGI
Size
Methylated CGI (n=55,075)
CGI (n*10³) 27.4 20.3 30.5 12.2
0 5
2.5 Kb
D
hk CGI
rep CGI
Log10(Units)
Max
Min
Count
Size, bp 777 309
SD, bp ± 895 ± 321
E
Hypo-DMC 10,872
Hyper-DMC 3,751
Promoter (-10K)
TSS
Gene Body
Downstream (5K)
InterGenic
rep-CGI
hk-CGI
G
Hyper-rep-DMC 2,181
SINE 92%
Simple 1%
Retroposon 2%
Satellite 1%
LINE 1%
ERV 3%
F
Hypo-DMC 18,441
Hyper-DMC 772
H
Hyper-rep-DMC 361
SINE 75%
ERV 13%
Simple 5%
Retroposon 1%
Satellite 1%
LINE 4%
I
Pathways in cancer 20,34%
Herpes simplex virus 1 infection 22,6%
Endocytosis 18,64% **
Axon guidance 17.51% **
Pathways of neurodegeneration 20,9%

© The Author(s)

Figure EV5. Methylation changes in different CGI populations related to DiPRO1 inhibition in myoblasts and RMS cells.

Methylation by MIRA-seq was analyzed in DNA samples from TE671 RMS cells and human myoblasts (Myo). Cells expressing a DiPRO1-targeting shRNA (shDiPRO1) were compared to control cells expressing a nontargeting shRNA (shCtl). Signals from methylation-enriched DNA (ENR) were normalized to the unenriched input DNA (INP). CGI, CpG islands according to Gardiner-Garden and Frommer criteria; DMC, differentially methylated CGI; hk-DMC, housekeeping DMC w/o repetitive elements; rep-DMC, DMC with repetitive elements. Analysis was performed using EaSeq software. (**A**) Tracking of signals on both strands corresponding to methylation signals within CGI regions of PAX3, MYOD1, FRGR1, and JUP genes in RMS control cells. The Y-axis represents signal intensity; the X-axis represents hg38 genomic coordinates. (**B**) Heatmaps displaying methylation levels in size-sorted CGIs in shDiPRO1 and shCtl and corresponding inputs. The bar reflects the signal intensity. Y-axis: DNA fragments per 1 M reads per 1 K. X-axis: 2.5 Kb surrounding each region. (**C**) Density plot of the log-transformed size of hk- and rep-CGI in the human genome. (**D**) Tracking of signals on both strands corresponding to methylation signals within CGI regions of MYOG, PAX3, and PAX7 genes in control myoblasts. The Y-axis represents signal intensity; the X-axis represents hg38 genomic coordinates. (**B**) Genomic distribution of hypo- (top) and hypermethylated (bottom) DMC is shown as % of DMC total number. (**E**, **F**) Genomic distributions of hypo- (left) and hypermethylated (right) DMC are shown as % of DMC total number, in RMS cells (**E**) and myoblasts (**F**). (**G**, **H**) DNA repeat class distribution within DiPRO1-linked hypermethylated DMC regions in RMS cells (**G**) and myoblasts (**H**). (**I**) KEGG-based functional analysis of genes associated with hypomethylated DMC regions of RMS cells under DiPRO1 KD. *P* value of the group corrected with Bonferroni step-down, kappa score = 0.4. ClueGO, a Cytoscape plug-in was used.

 © The Author(s)
